# Supplementary material for: Interaction between Butyrate and Tumor Necrosis Factor α in Primary Rat Colonocytes
Source: Biomolecules. 2023 Jan 30;13(2):258. doi: 10.3390/biom13020258 (PMC9953264; doi:10.3390/biom13020258)
Supplement: Supplementary file 1 [file biomolecules-13-00258-s001.zip › biomolecules-2114585-supplementary.pdf]

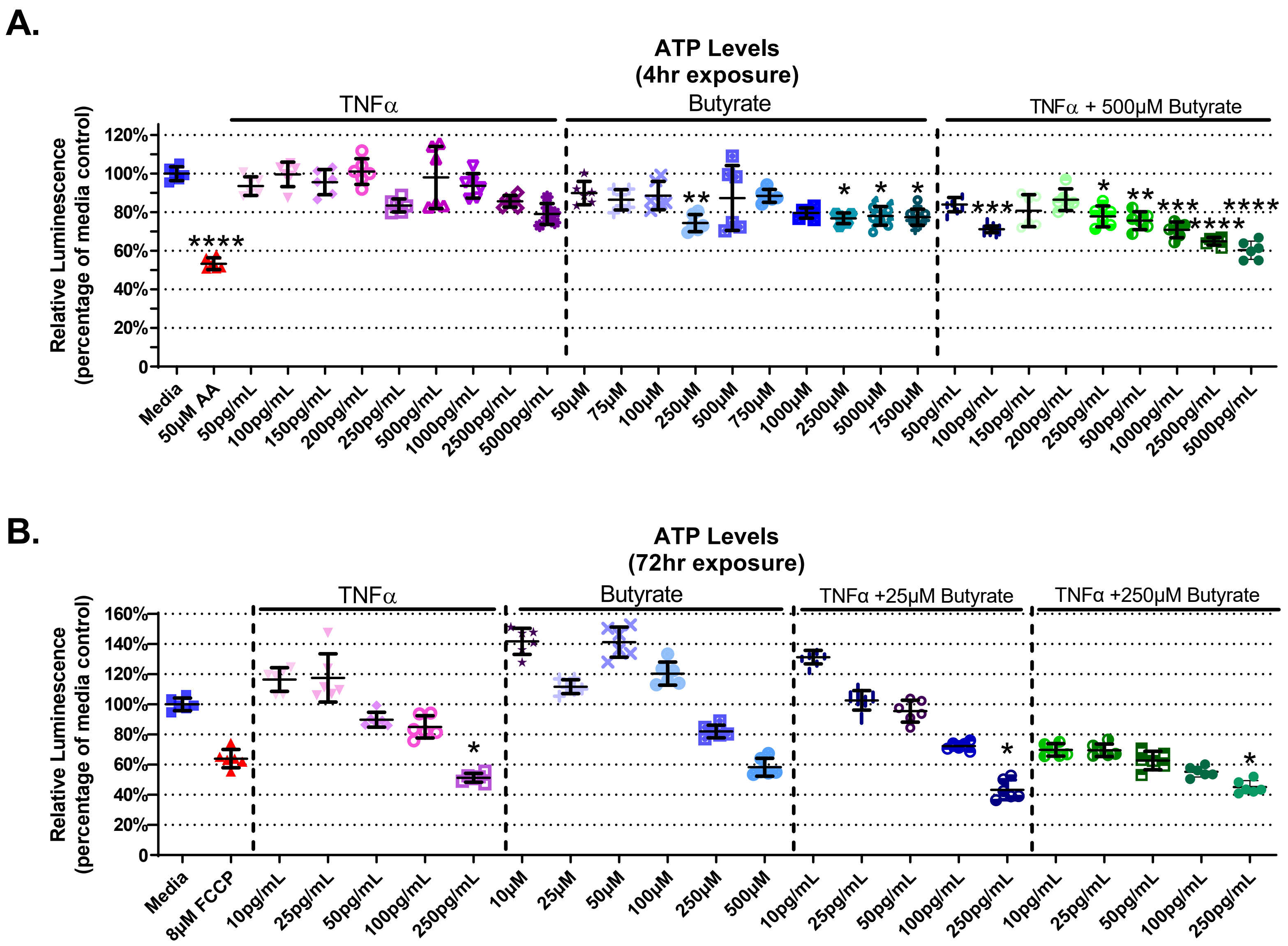

Supplemental Figure S1 Relative ATP Levels after exposure to butyrate and TNFα. (A.) 4 hours (B.) 72 hours. FCCP or Antimycin (AA) were used as positive controls. Relative Luminescence is graphed for each experimental group and is normalized to the media control (Horizonal bar represents mean relative fluorescence ± standard deviation). Asterisks indicate degree of significance from media control (Kruskal-Wallis test followed by a Dunn's multiple comparison test to media control, n= 3-4/experiment, two independent experiments were combined, *= p<0.05, **=p<0.01, ***=p<0.001, ****= p<0.0001). For simplicity, we only show differences from the control group on the figure, however, in Supplemental Data Table 3, we provide all comparisons among all groups.

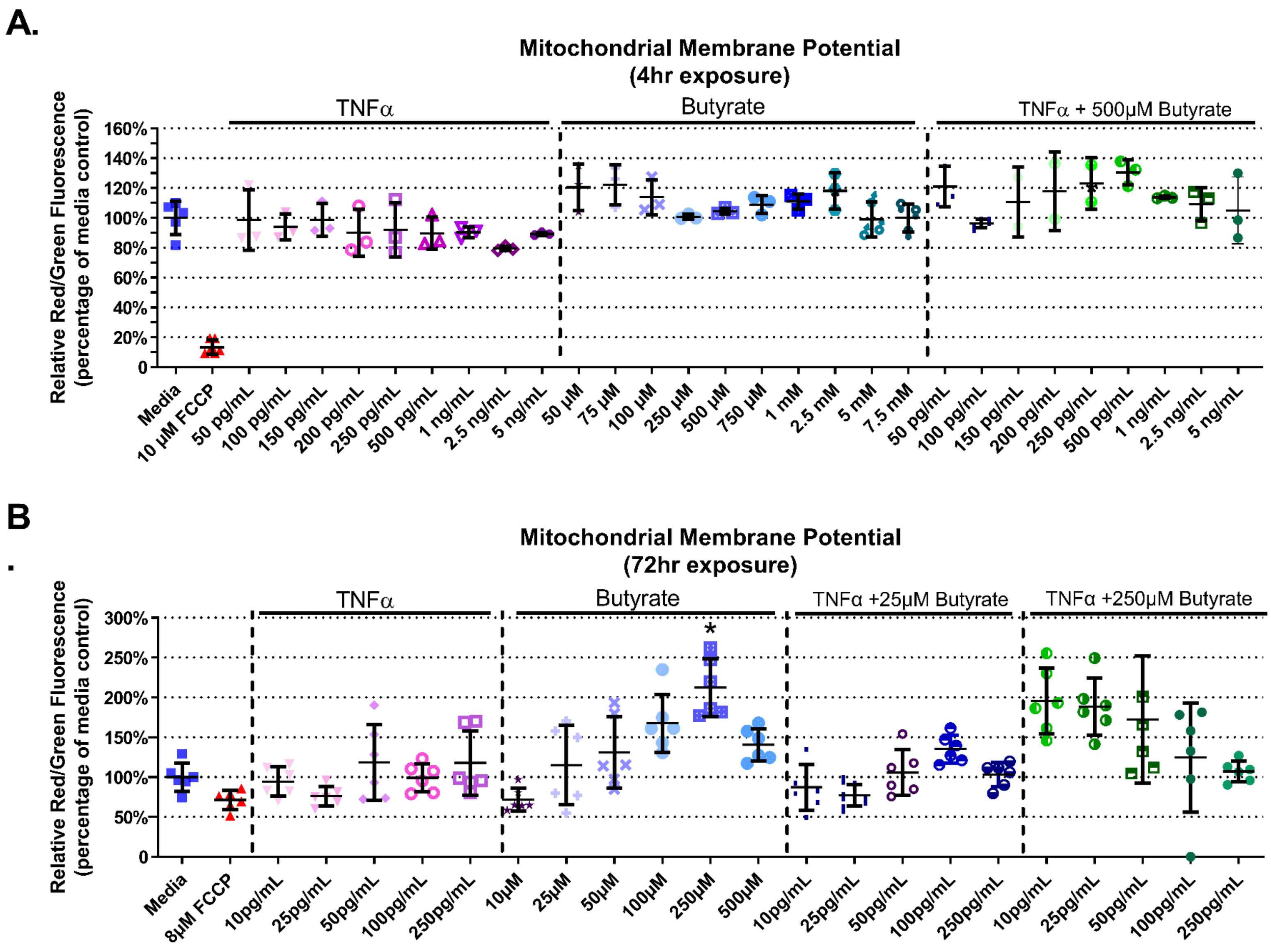

Supplemental Figure S2 Mitochondrial membrane potential after exposure to butyrate and TNFα for (A.) 4 hours (B.) 72 hours. FCCP was used as positive control. Relative red/green fluorescence ratio is graphed for each experimental group and is normalized to the media control (Mean relative fluorescence ± standard deviation). Asterisks indicate degree of significance from media control (Kruskal-Wallis test followed by a Dunn's multiple comparison, n=3/experiment, significance determined at *= p<0.05, **=p<0.01, ***=p<0.001, ****= p<0.0001). For simplicity, we only show differences from the control group on the figure, however, in Supplemental Data Table 3, we provide all comparisons among all groups.

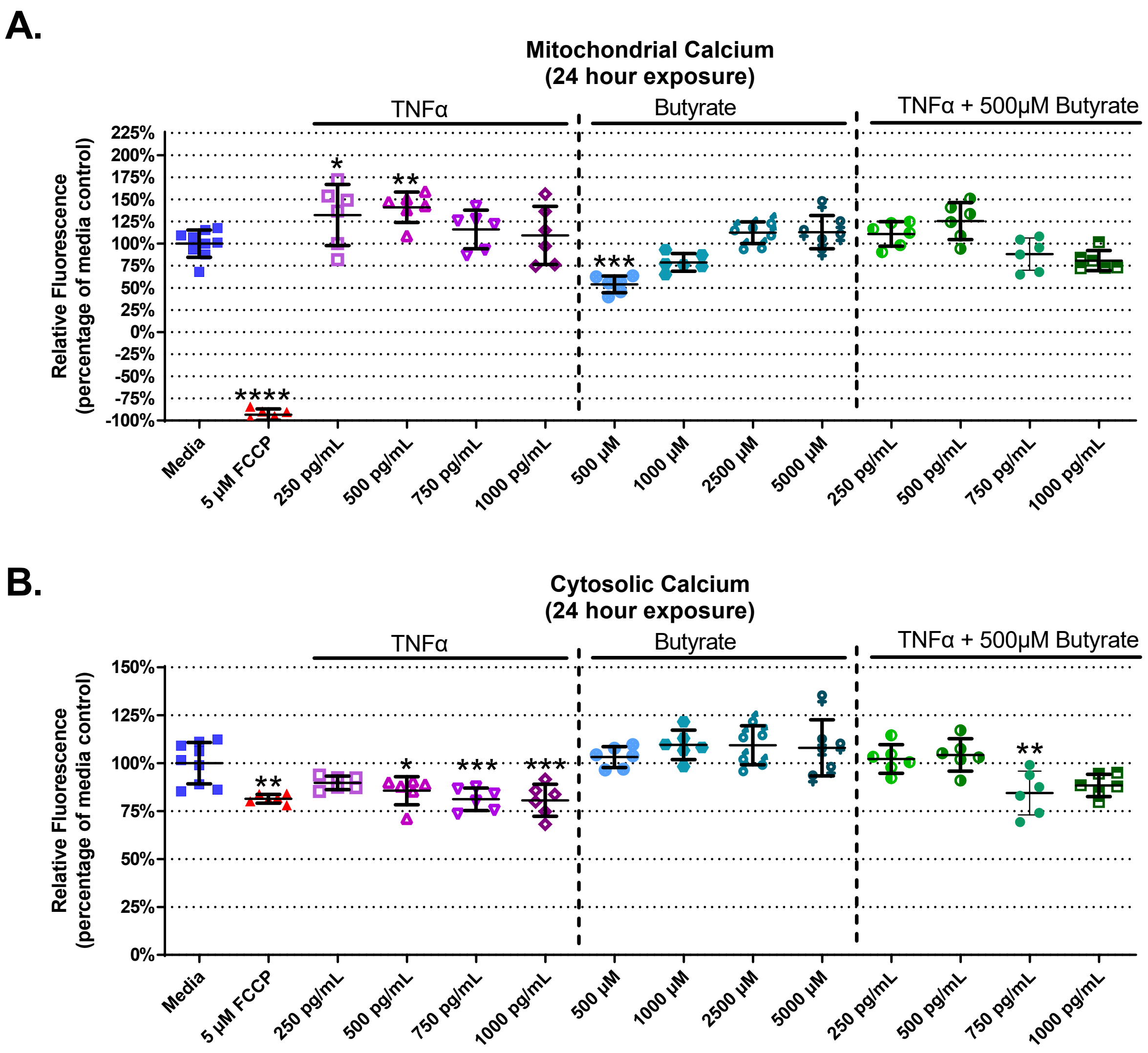

Supplemental Figure S3 (A) Mitochondrial and (B) Cytosolic calcium of primary rat colon epithelial cells after exposure to butyrate and TNFα for 24 hours. FCCP was used as positive control as it inhibits the uptake of the mitochondrial dye Rhod-2 AM. Asterisks indicate degree of significance from media control (One-Way ANOVA followed by a Dunnett's multiple comparison, mean ± S.D., n=3, significance determined at *= p<0.05, **=p<0.01, ***=p<0.001, ****= p<0.0001). For simplicity, we only show differences from the control group on the figure, however, in Supplemental Data Table 3, we provide all comparisons among all groups.

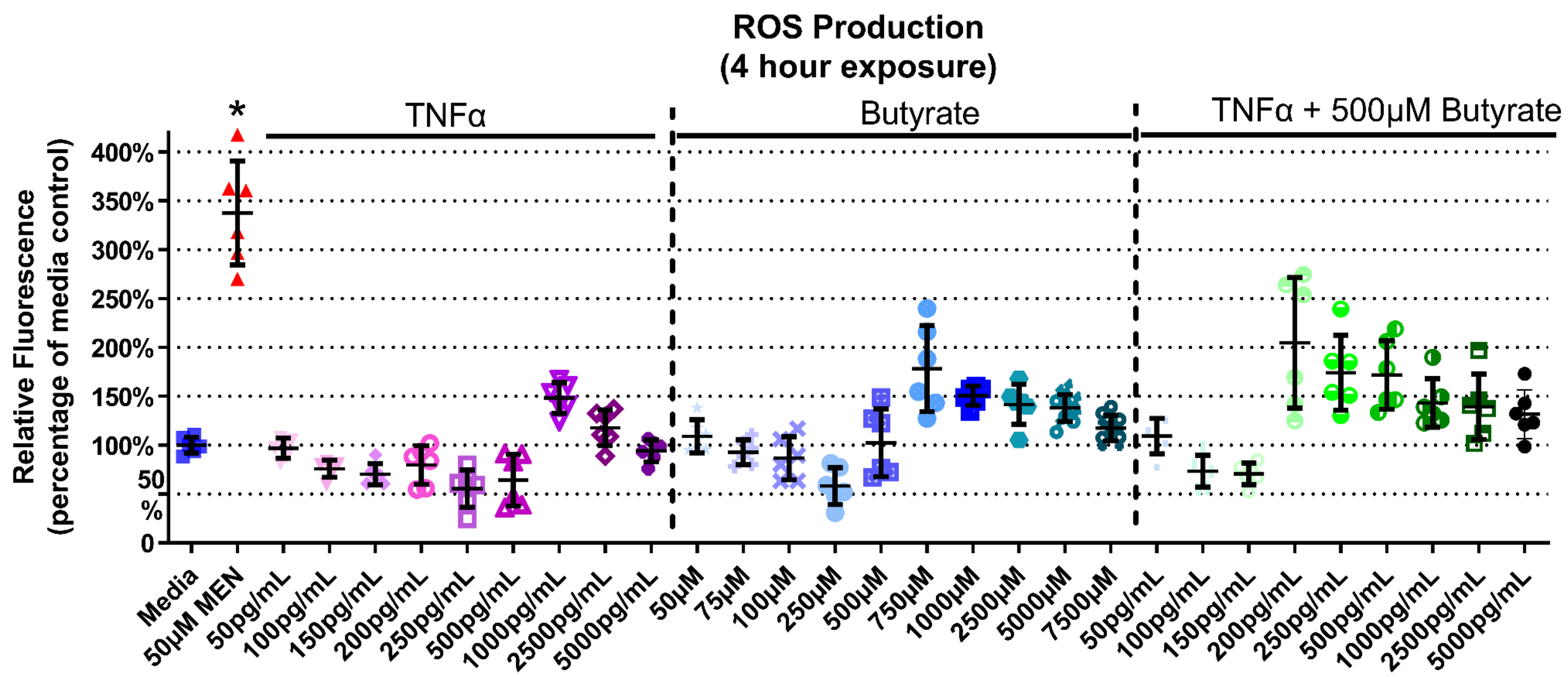

Supplemental Figure S4 Reactive oxygen species (ROS) production of primary rat colon epithelial cells after exposure to butyrate and TNFα for 4 hours. Menadione (MEN) was used as a positive control as a potent inducer of ROS. (Mean fluorescence ± standard deviation) (n=3). Asterisks indicate degree of significance from media control (Kruskal-Wallis test followed by a Dunn's multiple comparison, n=3, significance determined at *= p<0.05, **=p<0.01, ***=p<0.001, ****= p<0.0001). For simplicity, we only show differences from the control group on the figure, however, in Supplemental Data Table 3, we provide all comparisons among all groups.

Supplemental Table S1. Primers used for real-time PCR analysis.

Gene name	Gene Symbol	Forward (5' to 3')	Reverse (5' to 3')	Product length	Reference
Ribosomal 18s	Rps18	GTAAACCCGTTGAACCCCATT	CCATCCAATCGGTAGTAGCG	136	Han et al., 2020
Beta-actin	Bactin	GGACCTGACAGACTACCTCA	GTTGCCAATAGTGATGACCT	208	Han et al., 2020
Caspase 3	Casp3	AATTCAAGGGACGGGTCATG	TGACACAATACACGGGATCTG	180	Li et al., 2020
Catalase	Cat	ATGTGGTTTTCACCGACGAG	CCTTTGCCTTGGAGTATCTGG	135	
Leucine rich repeat containing G protein coupled receptor 5	Lgr5	AACATCAGTCAGCTACCCGC	GCATTTCCAGCAAGACGCAA	74	
Citrate synthase	Cs	CGGTTCTTGATCCTGATGAGGG	ACTGTTGAGGGCTGTGATGGC	284	Serdan et al., 2021
Pyruvate dehydrogenase kinase 1	Pdk1	GGATTGCCCATATCACGTCTTT	TCCCGTAACCCTCTAGGGAATA	2005	Xiang et al., 2014
Pyruvate dehydrogenase kinase 2	Pdk2	ATGAAAGAGATCAACCTGCTTCC	GGCTCTGGACATACCAGCTC	82	Yao et al., 2021
Pyruvate dehydrogenase kinase 4	Pdk4	GACCCAGTCACCAATCAAAATCT	GGTTCATCAGCATCCGAGTAGA	82	Yao et al., 2021
PPARG coactivator 1 alpha	Ppargc1a	CCCACAGAGAACAGAAACAG	GGGTCAGAGGAAGAGATAAAG	118	Hernández et al., 2012
Sepiapterin reductase	Spr	GTTCGGTGTTGCTTCTAAGC	ATAGCTCAGCACCCTCACAC	488	
Solute carrier family 16 member 1	Slc16a1	GGTGTCATTGGAGGTCTTGGG	GGCCAATGGTCGCTTCTTG	90	Enoki et al., 2006
Solute carrier family 16 member 3	Slc16a3	GGGTCATCACTGGCTTGGGT	GGAACACGGGACTGCCTGC	123	Enoki et al., 2006
BCL2 associated X	Bax	AGGATGATTGCTGATGTGGATAC	CACAAAGATGGTCACTGTCTGC	300	Van Der Hoeven et al., 2003
B-cell lymphoma 2	Bcl2	GCTACGAGTGGGATACTGGAGA	AGTCATCCACAGAGCGATGTT	446	Schoemaker et al., 2002
Superoxide dismutase 1	Sod1 (Cu/Zn SOD)	GCGTCATTCACTTCGAGCAG	ATTGATGGACATGGAACCCA	102	
Superoxide dismutase 2	Sod2 (Mn SOD)	AAGGTCGCTTACAGATTGCC	GCTAACATTCTCCCAGTTGATTAC	176	
TNF receptor superfamily member 1B	Tnfrsf1b	TCAGATGTGCTGTGCTAAGTGTCC	GCCAGGATGCTACAAATGCG	291	

1. Han, H., Liu, L., Chen, M., Liu, Y., Wang, H., & Chen, L. (2020). The optimal compound reference genes for qRT-PCR analysis in the developing rat long bones under physiological conditions and prenatal dexamethasone exposure model. Reproductive Toxicology, 98, 242-251.
2. Li, Xin, et al. "Effect of nicotine on placental inflammation and apoptosis in preeclampsia-like model." Life Sciences 261 (2020): 118314.
3. Serdan, T. D. A., Masi, L. N., Pereira, J. N. B., Rodrigues, L. E., Alecrim, A. L., Scervino, M. V. M., ... & Hirabara, S. M. (2021). Impaired brown adipose tissue is differentially modulated in insulin-resistant obese wistar and type 2 diabetic Goto-Kakizaki rats. Biomedicine & Pharmacotherapy, 142, 112019.
4. Xiang, L., Gilkes, D. M., Chaturvedi, P., Luo, W., Hu, H., Takano, N., ... & Semenza, G. L. (2014). Ganetespib blocks HIF-1 activity and inhibits tumor growth, vascularization, stem cell maintenance, invasion, and metastasis in orthotopic mouse models of triple-negative breast cancer. Journal of molecular medicine, 92(2), 151-164.
5. Yao, S., Shang, W., Huang, L., Xu, R., Wu, M., & Wang, F. (2021). The oncogenic and prognostic role of PDK1 in the progression and metastasis of ovarian cancer. Journal of Cancer, 12(3), 630.

6. Hernández, A., Curi, R., Salazar, L. A., HERNANDEZ, A., CURI, R., & SALAZAR, L. (2012). Repression of Ppargc1a Gene in Liver of Hyperglycemic Rats Induced with High Fat Diet Combined with Streptozotocin. Int. J. Morphol, 30(2), 643-650.
7. Enoki, T., Yoshida, Y., Lally, J., Hatta, H., & Bonen, A. (2006). Testosterone increases lactate transport, monocarboxylate transporter (MCT) 1 and MCT4 in rat skeletal muscle. The Journal of physiology, 577(1), 433-443.
8. Van Der Hoeven, J. A., Moshage, H., Schuurs, T., Nijboer, M., Van Schilfgaarde, R., & Ploeg, R. J. (2003). Brain death induces apoptosis in donor liver of the rat. Transplantation, 76(8), 1150-1154.
9. Schoemaker, M. H., Ros, J. E., Homan, M., Trautwein, C., Liston, P., Poelstra, K., ... & Moshage, H. (2002). Cytokine regulation of pro-and anti-apoptotic genes in rat hepatocytes: NF-κB-regulated inhibitor of apoptosis protein 2 (cIAP2) prevents apoptosis. Journal of hepatology, 36(6), 742-750.

Supplemental Data Table S2. Contains metabolomics data.

Concentration (µg/g cells)

Polarity	Name	Formula	Annot. DeltaI	Calc. MW	RT [min]
Negative	(+/-)13-HODE	C18 H32 O3	0.48	296.23529	16.216
Positive	(1R,9S)-11-[(Methylsulfanyl)	C18 H20 N2 (	-2.57	360.09569	5.322
Positive	(4-Fluorophenyl)[(3S)-3-(1-n	C19 H18 F N3	4.82	323.14495	9.989
Positive	[(3S)-3-(1H-Benzimidazol-2-	C18 H16 F N3	4.92	309.12926	8.611
Positive	[1-(4-fluorophenyl)-5-methy	C21 H20 F N3	4.28	349.16053	11.166
Positive	1-(4-chlorophenyl)-1-pheny	C19 H16 Cl N	2.31	309.09276	7.998
Positive	1-(4-Fluorophenyl)-3-(2-met	C18 H14 F N3	4.9	323.10859	8.04
Positive	1-allyl-4,5-diphenyl-2-(2-thi	C22 H18 N2 S	-2.03	342.11837	10.445
Positive	1,5-diphenyl-1H-1,2,4-triazo	C14 H11 N3 S	-1.96	253.06687	3.227
Positive	2-(2-amino-3-methylbutana	C14 H20 N2 (	-2.06	264.14685	9.284
Negative	2-(Acetylamino)hexanoic ac	C8 H15 N O3	0.63	173.1053	10.34
Positive	2-[(3S)-1-(2-Pyridinylmethyl	C17 H17 N3 S	-1.62	295.11384	8.238
Positive	2-[(3S)-1-(3,4-Difluorobenzy	C19 H18 F2 N	1.19	328.13911	12.861
Positive	2-Amino-N,N-diethylacetam	C6 H14 N2 O	-1.47	130.11042	2.615
Negative	2-Furoic acid	C5 H4 O3	0.26	112.01607	7.676
Negative	2-Naphthalenesulfonic acid	C10 H8 O3 S	0.19	208.01945	10.376
Positive	2,3,4,9-Tetrahydro-1H-β-ca	C12 H12 N2 (	-1.54	216.08954	9.279
Positive	2,4-Dimethylbenzaldehyde	C9 H10 O	-0.66	134.07308	11.3
Positive	2,5-Dimethylpyrazine	C6 H8 N2	-1.07	108.06863	2.858
Positive	3-(2-fluorophenyl)-2-[(2-fluo	C15 H10 F2 N	1.45	304.04863	2.488
Positive	3-(allylsulfanyl)-4-[4-(benzyl	C19 H19 N3 (	-1.28	337.12445	8.388
Positive	3-(benzylthio)-5-[4-(tert-but	C20 H23 N3 S	-2.33	337.16048	9.822
Positive	3-[4-(tert-butyl)anilino]-2-(3	C18 H18 N2 (	-2.6	310.11318	10.099
Positive	3-amino-2-phenyl-2H-pyraz	C12 H10 N4 (	-1.92	242.07991	11.373
Positive	3-Hydroxy-2-methylpyridine	C6 H7 N O	-1	109.05265	2.597
Negative	3-Hydroxy-3-(methoxycarbo	C7 H10 O7	-0.4	206.04257	7.687
Positive	3-Phenyl-2,3-dihydro-1,3-be	C13 H10 N2 S	-1.52	226.05613	3.707
Positive	3,5-Dimethoxyaniline	C8 H11 N O2	0.32	153.07903	8.302
Positive	4-(2-methoxyphenyl)-1-(3-p	C20 H21 N3 (	-3.34	351.13936	8.709
Positive	4-(4-methoxyphenyl)-2-(2-tl	C15 H12 N2 (	-2.01	268.0665	7.653
Positive	4-[2-(2-Thienyl)-4-quinolyl]r	C17 H16 N2 (	-1.74	296.09782	9.223
Positive	4-{[(3S)-3-(5-Fluoro-1H-ben	C19 H18 F N3	3.34	339.13944	8.501
Positive	4-Hydroxybenzaldehyde	C7 H6 O2	-1.62	122.03658	4.58
Positive	4-Methoxychalcone	C16 H14 O2	-4.54	238.0983	9.258
Positive	4-methyl-6-[(4-methylpheny	C17 H15 N3 S	-2.3	293.098	7.927
Negative	4-Methylphenol	C7 H8 O	0.3	108.05755	8.721
Positive	4-Morpholinepropanesulfor	C7 H15 N O4	-1.9	209.07178	1.949
Positive	4-morpholinobenzoic acid	C11 H13 N O3	-1.62	207.08921	11.456

Negative	4-Nitrophenol	C6 H5 N O3	0.67	139.02704	12.253
Negative	4-Oxoproline	C5 H7 N O3	0.04	129.0426	3.219
Positive	4,4'-Bis(diethylamino)benzo	C21 H28 N2 (	-3.25	324.21911	14.93
Positive	5-(2-Chloro-6-fluorobenzyl)-	C13 H12 Cl F	4.84	298.03573	1.614
Positive	5-Fluoro THJ	C22 H21 F N4	0.22	376.17002	10.013
Positive	5-Hydroxylysine	C6 H14 N2 O:	-1.44	162.10021	1.581
Positive	5(Z),8(Z),11(Z)-Eicosatrienoi	C22 H39 N O2	-2.5	349.29721	17.147
Positive	6-Methyl-2-pyridinemethan	C7 H9 N O	-1.13	123.06828	9.349
Positive	7-Methylguanine	C6 H7 N5 O	-1.16	165.06487	3.282
Negative	8-iso Prostaglandin F1?	C20 H36 O5	0.94	356.25661	13.465
Positive	8,8-dimethyl-2-phenyl-4H,8	C20 H16 O3	-4.35	304.10862	7.605
Negative	9-Phenanthrol	C14 H10 O	-3.05	194.07257	1.56
Positive	Acetyl-L-carnitine	C9 H17 N O4	-1.34	203.11549	2.746
Positive	Acetylcholine	C7 H15 N O2	-1.31	145.11009	2.021
Positive	Adenine	C5 H5 N5	-0.55	135.05442	2.736
Positive	Adenosine 5'-monophospha	C10 H14 N5 (	-2.43	347.06224	2.738
Negative	Adipic acid	C6 H10 O4	0.71	146.05801	8.53
Positive	Aniline	C6 H7 N	-1.49	93.05771	4.125
Positive	Benperidol	C22 H24 F N3	3.62	381.18663	11.676
Negative	Benzoic acid	C7 H6 O2	0.31	122.03682	10.297
Positive	Betaine	C5 H11 N O2	-1.66	117.07878	1.734
Positive	Biopterin	C9 H11 N5 O:	-2.08	237.0857	5.571
Positive	Bis(4-ethylbenzylidene)sorb	C24 H30 O6	-2.46	414.20322	14.186
Positive	Chlormequat	C5 H12 Cl N	-1.46	121.06565	2.131
Positive	Choline	C5 H13 N O	-1.91	103.09952	1.887
Negative	Citric acid	C6 H8 O7	0.51	192.0271	2.867
Positive	Creatine	C4 H9 N3 O2	-1.56	131.06927	1.975
Positive	Creatinine	C4 H7 N3 O	-1.13	113.05878	1.89
Positive	Cyproheptadine	C21 H21 N	-2.81	287.16659	14.857
Negative	Cystathionine	C7 H14 N2 O	0.16	222.06746	1.707
Positive	Cysteinylglycine	C5 H10 N2 O:	-1.53	178.04094	2.787
Negative	Cytarabine	C9 H13 N3 O!	0.61	243.08567	2.68
Positive	Cytidine	C9 H13 N3 O!	-2.05	243.08502	2.736
Positive	Cytidine 5'-monophosphate	C9 H14 N3 O	-2.46	323.05106	2.342
Positive	Cytosine	C4 H5 N3 O	-1.35	111.04311	2.736
Negative	D-(+)-Malic acid	C4 H6 O5	0.67	134.02161	2.903
Positive	D-(+)-Proline	C5 H9 N O2	-1.51	115.06315	2.007
Positive	D-Panthenol	C9 H19 N O4	-1.6	205.13108	8.092
Positive	D-Serine	C3 H7 N O3	-0.86	105.0425	2.551
Positive	delta8-THC-d9	C21 H21 [2]H	1.73	323.28163	16.988
Positive	Desmethylclozapine	C17 H17 Cl N	-1.59	312.11368	8.526
Positive	Di(2-ethylhexyl) phthalate	C24 H38 O4	-2.31	390.27611	18.108
Positive	Didemethylisoproturon	C10 H14 N2 (	-1.24	178.11039	7.562

Positive	Diphenylamine	C12 H11 N	-1.69	169.08886	14.344
Positive	Disperse blue 7	C18 H18 N2 (	0.06	358.11651	11.401
Negative	DL-Alanine	C3 H7 N O2	-0.48	89.04764	3.131
Positive	DL-Arginine	C6 H14 N4 O2	-0.67	174.11156	2.843
Negative	DL-Lactic Acid	C3 H6 O3	-0.71	90.03163	2.85
Negative	DL-Malic acid	C4 H6 O5	0.31	134.02156	2.294
Positive	DL-Serine	C3 H7 N O3	-0.85	105.0425	8.719
Positive	DL-Tryptophan	C11 H12 N2 (	-1.79	204.08951	8.619
Negative	DL-β-Leucine	C6 H13 N O2	-0.02	131.09463	5.151
Negative	Docosatrienoic acid	C22 H38 O2	0.91	334.28748	17.33
Negative	Dodecyl sulfate	C12 H26 O4 S	0.84	266.1554	17.812
Positive	Ethyl 2-(4-toluidino)-4-(triflu	C15 H14 F3 N	-2.46	325.10301	11.532
Negative	Ethyl myristate	C16 H32 O2	0.76	256.24042	16.67
Positive	Ethyl violet	C31 H41 N3	-2.55	455.32889	13.94
Negative	Ethylmalonic acid	C5 H8 O4	0.16	132.04228	2.698
Positive	Fingolimod	C19 H33 N O2	-2.54	307.25035	17.828
Positive	Flurandrenolide	C24 H33 F O6	3.13	436.22748	9.614
Negative	Formononetin	C16 H12 O4	-1.96	268.07303	2.645
Negative	Fumaric acid	C4 H4 O4	0.19	116.01098	2.29
Negative	Gluconic acid	C6 H12 O7	-0.08	196.05829	1.617
Negative	Glutaric acid	C5 H8 O4	0.34	132.0423	7.575
Negative	Glycine	C2 H5 N O2	0.02	75.03203	2.125
Negative	Glycolic acid	C2 H4 O3	-0.62	76.016	2.754
Positive	Glycyl-L-leucine	C8 H16 N2 O3	-1.21	188.11587	8.185
Positive	Guanine	C5 H5 N5 O	-1.24	151.04922	2.736
Positive	Hexanoylcarnitine	C13 H25 N O4	-2.18	259.17779	10.536
Positive	Hypoxanthine	C5 H4 N4 O	-1.21	136.03835	2.994
Negative	Indole-3-acetic acid	C10 H9 N O2	0.23	175.06337	12.26
Negative	Isobutyric acid	C4 H8 O2	-0.49	88.05239	8.29
Positive	Isoleucine	C6 H13 N O2	-1.3	131.09446	5.145
Negative	Isophthalic acid	C8 H6 O4	0.22	166.02664	9.621
Positive	L-(-)-Methionine	C5 H11 N O2	-1.28	149.05086	2.778
Negative	L-(+)-Lactic acid	C3 H6 O3	-0.39	90.03166	2.741
Positive	L-Alanyl-L-proline	C8 H14 N2 O3	-1.34	186.10019	3.009
Positive	L-Dopa	C9 H11 N O4	-1.25	197.06856	8.374
Positive	L-Glutamic acid	C5 H9 N O4	-1.32	147.05296	1.743
Positive	L-Glutathione (reduced)	C10 H17 N3 (	-2.56	307.08302	7.216
Positive	L-Histidine	C6 H9 N3 O2	-1.29	155.06928	1.607
Positive	L-Isoleucine	C6 H13 N O2	-1.3	131.09446	4.656
Positive	L-Methionine sulfoxide	C5 H11 N O3	-1.39	165.04574	1.76
Positive	L-Phenylalanine	C9 H11 N O2	-1.31	165.07876	7.843
Positive	L-Pyroglutamic acid	C5 H7 N O3	-1	129.04246	3.251
Positive	L-Threonine	C4 H9 N O3	-1.41	119.05807	1.648

Negative	L-Tyrosine	C9 H11 N O3	0.23	181.07393	4.587
Positive	L-Tyrosine methyl ester	C10 H13 N O3	-1.14	195.08932	9.606
Positive	L(-)-Carnitine	C7 H15 N O3	-1.57	161.10494	1.772
Negative	Methylmalonic acid	C4 H6 O4	0.09	118.02662	4.067
Negative	Methylsuccinic acid	C5 H8 O4	0.43	132.04231	8.022
Positive	N-[5-(tert-butyl)-3-isoxazoly	C15 H16 F3 N	-2.18	343.11363	10.368
Positive	N-{[(1S,4S,6S)-4-{[5-(2-Fluor	C26 H29 F N4	2.95	448.22878	11.117
Negative	N-Ethylglycine	C4 H9 N O2	0.01	103.06333	1.691
Positive	N,N-Bis(2-pyridinylmethyl)-1	C21 H17 N3 C	-2.23	359.10843	9.39
Positive	N,N-Diethyldodecanamide	C16 H33 N O	-2.26	255.25564	16.071
Positive	N,N-Diethylethanolamine	C6 H15 N O	-1.67	117.11517	2.561
Positive	N,N-Dimethylsphingosine	C20 H41 N O2	-2.51	327.31291	13.914
Positive	N,N'-Dicyclohexylurea	C13 H24 N2 C	-2.04	224.1884	13.949
Positive	N'-{6-[(5-chloro-3-pyridyl)ox	C13 H13 Cl N4	-0.85	276.07756	8.113
Positive	N2-(2,4-dimethylphenyl)-1,3	C15 H14 N2 S	-2.11	254.08723	8.086
Positive	N2-(3-Pyridyl)-4-(4-methoxy	C15 H13 N3 C	-2	283.07737	3.227
Positive	N2,1-Diphenyl-6-imino-1,6-c	C15 H14 N6	4.77	278.12932	7.171
Positive	N6,N6,N6-Trimethyl-L-lysine	C9 H20 N2 O2	-1.56	188.15218	1.596
Positive	N8-Acetylspermidine	C9 H21 N3 O	-1.52	187.16818	1.764
Positive	Nicotinamide	C6 H6 N2 O	-1.63	122.04781	2.876
Positive	Nicotinamide 1-oxide	C6 H6 N2 O2	-1.01	138.04279	2.742
Positive	Nicotinamide adenine dinuc	C21 H27 N7 C	-1.97	663.10782	2.739
Positive	Niflumic acid	C13 H9 F3 N2	0.66	282.0618	1.617
Positive	NP-001346	C11 H15 N5 C	-1.92	297.08899	1.839
Negative	NP-008147	C19 H18 O5	-1.21	326.11503	7.471
Positive	NP-011220	C11 H18 N2 C	-1.71	210.13647	10.576
Negative	NP-016455	C11 H18 N2 C	0.26	242.12672	9.848
Negative	Orotidine	C10 H12 N2 C	0.89	288.05962	1.899
Positive	Palmitoyl sphingomyelin	C39 H79 N2 C	-2.62	702.56573	13.916
Negative	Perfluoro-1-butanesulfonic	C4 H F9 O3 S	0.88	299.95053	13.446
Positive	Periciazine	C21 H23 N3 C	-1.52	365.15563	9.595
Positive	Perillartine	C10 H15 N O	-1.13	165.11518	11.598
Positive	Prolylleucine	C11 H20 N2 C	-1.7	228.147	9.292
Positive	Propionylcarnitine	C10 H19 N O4	-1.69	217.13104	7.439
Positive	Pyridoxamine	C8 H12 N2 O2	-1.38	168.08965	7.609
Positive	Pyrimethamine	C12 H13 Cl N4	-1.27	248.08256	2.693
Negative	Pyruvic acid	C3 H4 O3	-0.17	88.01603	2.697
Positive	Rhodamine 6G	C28 H30 N2 C	-2.17	442.22468	13.67
Positive	Ricinine	C8 H8 N2 O2	-1.02	164.05841	8.837
Positive	S-Adenosylhomocysteine	C14 H20 N6 C	-1.96	384.12084	7.143
Positive	S-Adenosylmethionine	C15 H22 N6 C	-2.57	398.13621	1.839
Positive	Spermidine	C7 H19 N3	-1.38	145.1577	1.446
Positive	Spermine	C10 H26 N4	-1.71	202.2154	1.417

Positive	Sphingosine (d18:1)	C18 H37 N O2	-2.26	299.28175	13.777
Positive	Spiperone	C23 H26 F N3	3.48	395.20228	11.966
Positive	Stearamide	C18 H37 N O	-2.18	283.2869	17.286
Positive	tert-Butyl N-[1-(aminocarbo	C11 H22 N2 C	-1.68	230.16266	8.328
Positive	Thiamine	C12 H16 N4 C	-2.55	264.10381	1.984
Positive	Thiosildenafil	C22 H30 N6 C	-0.73	490.18172	12.553
Positive	Thymine	C5 H6 N2 O2	-1.06	126.04279	7.791
Positive	Tolycaine	C15 H22 N2 C	-2.15	278.16245	9.9
Positive	Tyrosylalanine	C12 H16 N2 C	-1.99	252.1105	7.927
Negative	UDP-N-acetylglucosamine	C17 H27 N3 C	1.09	607.08223	1.971
Positive	Uracil	C4 H4 N2 O2	-1.56	112.0271	4.5
Positive	Valine	C5 H11 N O2	-1.66	117.07878	2.708
Negative	Xanthine	C5 H4 N4 O2	0.19	152.03345	3.78
Positive	α-Aspartylphenylalanine	C13 H16 N2 C	-2.13	280.10533	8.706
Negative	β-Alanine	C3 H7 N O2	-0.29	89.04765	8.068
Positive	β-Nicotinamide mononucle	C11 H15 N2 C	-2.76	334.05568	2.393
Positive	Y-Glutamylcysteine	C8 H14 N2 O5	-2.34	250.06176	2.769
Positive	Y-L-Glutamyl-L-glutamic aci	C10 H16 N2 C	-2.19	276.09515	2.694

Supplemental Data Table S3. Contains all statistical analysis output for figures and Supplemental figures.

JJAH_VAR	Media	Media	Media	Media	Media	Media
Tube Label	M1	M2	M3	M4	M5	M6
Tube identity	Media only c	Media only c	Media only c	Media only c	Media only c	Media only c
Collection Da	44032	44032	44032	44032	44032	44032
mzCloud Best	Sp01	Sp02	Sp03	Sp04	Sp05	Sp06
97.9	1.01846981	0.45209742	0.14275915	0.80757921	0.00964375	0.24379139
98.6	0.05988578	0.03686436	0.08345453	0.10055124	0.03438496	0.039792
99.7	0.11364555	0.05995915	0.10006208	0.12577368	0.05791658	0.0859783
99.4	0.17178302	0.13062564	0.12685862	0.09291917	0.19677073	0.12887366
98.3	0.1741959	0.08840949	0.14761929	0.15928773	0.10901186	0.142027
97.7	0.03481366	0.01934124	0.01934407	0.0784366	0.02615891	0.03451778
99.1	0.02582674	0.01513479	0.00587637	0.0337712	0.01883519	0.02282731
98.7	0.0170368	0.01052273	0.01627753	0.01354222	0.01082755	0.01991755
99.9	0.21579324	0.14048743	0.20508694	0.18385305	0.15192635	0.20465713
98.3	0.13636283	0.05940967	0.16499874	0.12866212	0.05180276	0.09656241
98.7	0.52293545	0.21233548	0.57394021	0.66131305	0.3006594	0.3185146
97.3	0.01535033	0.01822159	0.02159637	0.01700496	0.01900652	0.01444715
99.9	0.12225561	0.06869977	0.12633423	0.13342959	0.0561632	0.12234187
98.9	1.00938009	2.73098605	1.31844158	3.85875139	2.05730573	1.74305322
97.9	0.20113652	0.16419131	0.38325327	0.35131634	0.22849099	0.28181986
98.3	0.08011538	0.15206971	0.08912552	0.24168075	0.12997669	0.43182337
99.3	0.02447149	0.01651051	0.03651436	0.03916498	0.0186947	0.02498342
97.8	0.0501113	0.03281551	0.0477951	0.09731351	0.0592238	0.04082471
98	0.01531095	0.02170112	0.02025784	0.04959421	0.02497845	0.01466298
98.4	0.17934135	0.10138931	0.21432905	0.19545805	0.15465625	0.21120811
96.5	0.02807731	0.01753938	0.01654604	0.04390562	0.02290721	0.01870394
99.6	0.2522988	0.14652822	0.19557851	0.12529715	0.28402671	0.2654951
95.9	0.01117624	0.00531382	0.00959789	0.0147718	0.00679797	0.01224383
98.2	0.04235955	0.03824677	0.04150012	0.07682319	0.02413245	0.05257343
98.2	0.03739731	0.01922574	0.03384843	0.09425063	0.01409721	0.02076949
97.4	0.05962488	0.03937965	0.11337864	0.10854721	0.05695873	0.0733024
95.9	0.09198672	0.08401412	0.08472754	0.08936883	0.06408861	0.07329708
95.9	0.04730256	0.0335946	0.06003707	0.10658423	0.03890915	0.02839535
98.8	0.01137003	0.00739133	0.0086966	0.01192305	0.00876849	0.0097363
98.6	0.06068325	0.02738156	0.05873043	0.03895341	0.04638914	0.05414329
98.8	0.026755	0.01456725	0.02268663	0.02298999	0.0183361	0.02877014
97.9	0.01915087	0.01535629	0.01687804	0.01440897	0.02034545	0.01679841
98.7	2.8512133	1.58348733	3.05364173	2.58138364	1.50221118	2.27790758
95.9	1.10814028	0.7172617	0.72982695	1.0574734	1.01422333	1.78376941
100	0.01581806	0.01375304	0.01402834	0.0401867	0.01585439	0.01398767
99.7	1.0688617	0.56586835	0.92233196	1.74039286	0.65084429	0.56917228
100	0.0934129	0.12959411	0.138703	0.20729879	0.1061898	0.10319474
98.9	0.01860748	0.01438776	0.02124047	0.03989818	0.01199038	0.01333577

99.7	0.51842309	0.33862508	0.43210975	1.01774753	0.33491558	0.33262894
99.7	186.012129	107.435777	171.569516	351.930762	136.366279	124.96521
98.2	0.00038496	0.00092123	0.0002483	0.00062476	0.00023533	0.04519592
99	0.46743654	0.21028069	0.42461768	0.93986604	0.31488268	0.35422057
99.9	0.03305993	0.01721303	0.02565814	0.0378563	0.01582899	0.07364751
95.8	0.04756288	0.013925	0.01280959	0.05893134	0.01820493	0.00492479
97.1	0.03531427	0.01629442	0.00122367	0.0195956	0.00450448	0.00463971
98.1	0.07831592	0.06483557	0.07825359	0.16424116	0.04659644	0.05629475
97.9	0.05286766	0.02395998	0.05921329	0.05433206	0.0365513	0.04642974
95.9	0.1873418	0.11559901	0.13396049	0.59253188	0.0907095	0.09784334
98.2	0.01192691	0.00701797	0.00276392	0.00450638	0.00783851	0.00655785
98.5	0.36246653	0.18041959	0.20464258	0.27096542	0.21750512	0.19792679
98.2	0.41506614	0.22466382	0.70230415	0.50429994	0.29985144	0.5496215
95.6	0.27897514	0.17073975	0.36421561	0.39012634	0.20971438	0.2819783
99.7	0.38885902	0.25151494	0.40000145	0.46104037	0.3192108	0.40375473
99.8	3.64479091	3.57707264	4.13298708	6.64249939	3.7235451	3.76135688
98	0.18911324	0.11910665	0.13264705	0.26446741	0.12457046	0.24857443
98.4	0.00123699	0.00087624	0.00146514	0.00291502	0.0012185	0.00144121
99.2	0.01864413	0.01000765	0.0153333	0.01789475	0.0117894	0.01369015
98.5	0.47463395	0.25586225	0.37786977	0.71391641	0.29856454	0.32449301
98.5	0.59805824	0.20737962	0.60198621	1.01729189	0.26899892	0.39816455
97.2	0.07528209	0.0545243	0.10928305	0.11519969	0.06567767	0.09350569
99.7	0.0400213	0.05537551	0.03019664	0.09971714	0.0352134	0.0526898
100	2.23134982	1.3459524	3.50292259	4.91979582	1.7902187	2.27934193
97.9	0.93563141	0.6016	0.99826187	1.10380151	0.67034822	0.8125106
99.7	2.30089623	3.02711803	3.92142727	6.10627839	3.33625342	2.8861118
99.9	43.7767163	23.609652	47.4179057	58.903684	32.9818094	37.4351728
99.2	2.72674295	2.69405559	5.67544204	4.55502784	3.40572558	5.03244122
99.5	0.072784	0.07233831	0.06254521	0.10133269	0.03709444	0.0845146
97.9	7.42232822	4.49894054	5.71869316	12.9324875	5.87264276	4.30411167
97.1	0.54864495	0.39976483	0.65786076	0.78671118	0.45359617	0.5418095
95	1.96371249	1.84738699	2.01734452	4.95870263	2.21813362	1.95467852
99.6	4.24802263	2.25602845	3.68181113	4.89701694	2.86475186	3.53430596
99.6	0.06241159	0.05924553	0.04634854	0.06308363	0.0678824	0.06361066
100	10.6553441	5.73943827	10.5069458	14.507917	7.30603916	9.81931357
98.2	0.31286185	0.2195939	0.16874189	0.37563072	0.31618351	0.24350915
100	34.2601688	19.4387799	40.1205812	38.9720225	23.6491397	30.5334782
95.2	0.00220239	0.00243239	0.32935257	0.02391857	0.00169494	0.10518244
99.8	0.06774755	0.02586725	0.11129214	0.09063768	0.03756459	0.04005295
99.3	0.08461437	0.02297059	0.00608085	0.08410613	0.01425888	0.01101862
99.5	0.02328472	0.02715503	0.03953331	0.00356613	0.02048152	0.03151714
95.4	0.00981404	0.00325538	0.00157643	0.00998839	0.00140531	0.00093774
97.2	0.08540703	0.04363878	0.05052823	0.01322493	0.05359887	0.04969387

99.6	0.00712962	0.00548623	0.00333717	0.00934771	0.00249135	0.00639715
100	0.02170179	0.01351742	0.01835974	0.04658009	0.00918991	0.01563625
99.5	0.49759862	0.22562598	0.43732327	0.97789457	0.31351404	0.27030816
98.6	0.12608678	0.00307845	0.00540184	0.00622848	0.07535587	0.00380182
99.8	4.32989297	0.43478275	3.08402227	6.51867022	3.55288309	0.40912723
99.9	10.6822095	6.47147641	6.0521785	6.99764153	7.81878107	7.36394555
99.9	0.022268	0.01016802	0.01871335	0.04197283	0.0133737	0.01958712
99.8	2.11023824	1.34664362	2.99289654	1.70979476	1.45105729	2.1593395
99.2	12.5264443	7.03317387	13.1219959	11.3505757	6.87011768	8.28322722
99.8	9.0194325	2.49290493	0.43720157	3.60203812	0.77107275	1.33072199
99	1.73108299	0.05972798	0.02309731	0.11193393	1.53776934	0.06294192
98.3	0.00421738	0.00286119	0.00790735	0.00161434	0.003759	0.01724565
95.2	0.16402389	0.0463328	0.02693846	0.23493012	0.07657174	0.03703925
98.5	0.0035345	0.00155879	0.00394511	0.00620018	0.00131703	0.00087995
99.7	0.04496508	0.01877449	0.03727452	0.09945513	0.03006338	0.02738267
97.2	0.05476976	0.01039433	0.00485503	0.02419785	0.00763645	0.0051544
99.5	0.04696262	0.00755121	0.0153619	0.02498751	0.00842511	0.06596858
97.8	0.35555568	0.19702863	0.09980981	0.25227185	4.27693986	1.47637806
98.2	0.5443047	0.33499845	0.31768358	0.37873621	0.42066122	0.42615296
95.3	1.24815532	0.7057307	1.52018437	2.84155164	0.85738612	0.56829377
99.3	0.16796717	0.10540554	0.13584708	0.20359496	0.1298048	0.13367006
97.2	0.79456083	0.38151071	0.66389822	1.727642	0.57924572	0.50774794
98.6	0.24385296	0.15911749	0.19934805	0.59999104	0.22506617	0.13907958
98.6	0.41287756	0.24220572	0.38813307	0.34468457	0.20224094	0.29318178
99.9	1.08352206	0.74597133	1.73400017	2.02661605	0.73098018	0.91530531
98.1	0.00254701	0.00512213	0.00711969	0.00709013	0.00408038	0.00849921
100	84.1934999	49.1405569	94.572439	120.78008	56.6953921	66.0274048
99.5	0.3665349	0.17070937	0.35305364	0.47844032	0.22700184	0.19680105
99.1	1.02887177	0.02059219	0.90432734	2.44654865	0.79789933	0.02695565
99.8	37.2225151	17.3324041	47.9057649	49.8800176	19.905992	33.5370548
98.4	0.28558149	0.21360893	0.24696533	0.34462853	0.25273198	0.23435894
99.5	1.19166752	0.77288425	1.52320885	0.29093289	0.71045276	1.72448554
99.9	75.2965586	28.8205382	0.45026318	79.1942244	38.1421939	0.31464676
95.6	0.46691326	0.13371609	0.25256924	0.30231693	0.2714317	0.39503527
95.2	0.04249688	0.01257372	0.0471101	0.03151508	0.01754941	0.0274062
99.7	3.50381956	0.92263897	2.95795812	10.6692524	1.05145573	1.11884403
96.6	0.00362577	0.00217479	0.00423479	0.32165375	0.00325997	0.0033477
95.8	0.03458753	0.02030044	0.04567687	0.03130964	0.0232718	0.02286219
99.7	19.0363476	6.89307442	22.9273559	18.3770616	9.40242664	14.500447
98.3	2.42620367	0.80612521	2.13928476	3.679765	0.86839129	1.1158222
100	23.3354206	14.5960242	24.258572	24.3214693	14.658567	18.2131338
99.8	17.0641427	11.1048541	18.4801464	34.3805416	11.382952	13.1126454
96.2	0.24509942	0.12880473	0.32541335	0.26083725	0.18520564	0.25141232

99.9	29.4224203	15.4586928	24.0328196	31.1829826	15.0141058	16.8894902
98.8	0.00884928	0.00593518	0.01131923	0.00558273	0.00681655	0.01266667
99.9	1.47669131	0.69824539	1.40521519	1.65962429	0.86972107	1.21125059
95.8	67.6647071	31.5203054	79.953909	95.5122529	41.4288179	40.8099017
99	0.17225999	0.09800752	0.12489301	0.18930904	0.11622514	0.14910787
98.6	0.0382061	0.02710653	0.04109896	0.04624381	0.02633291	0.03340992
100	0.04268396	0.02759525	0.0458137	0.03819989	0.03287928	0.04112086
99.4	0.74369455	0.15978409	0.50166735	3.375761	0.20819199	0.18253324
98.4	0.02334878	0.01950685	0.02712416	0.02655589	0.01832573	0.0228999
99.2	0.16781153	0.08925579	0.02561975	0.25066398	0.04677779	0.04246452
98.8	1.72292234	1.15094663	1.43021101	2.79515213	1.23797695	1.26835269
95.2	0.0349973	0.01507703	0.02397777	0.09868029	0.01666912	0.01700848
99.6	0.12534091	0.11060584	0.12207978	0.15093709	0.03984467	0.10815903
95.2	0.00317943	0.00114404	0.00153709	0.00161663	0.00212681	0.00236838
97.8	0.08712772	0.06356315	0.06026618	0.09543807	0.06961726	0.07334181
97.7	0.05537458	0.03462039	0.04476746	0.05661205	0.0305761	0.04722064
96.8	0.00272559	0.00431009	0.00629539	0.00572952	0.004831	0.00685173
97.1	0.02902069	0.01571039	0.02829769	0.02031019	0.01741556	0.02580556
97.5	0.26892351	0.14375362	0.29725044	0.34187599	0.14974378	0.21831054
99.9	24.6935394	15.4529052	32.0189548	45.4733976	13.0515056	18.3267747
99.3	0.24573278	0.18057557	0.32879115	0.5543228	0.1272377	0.19270652
97.5	0.02611958	0.02881676	0.03711539	0.06284807	0.01666434	0.02485946
99.8	1.21496025	0.95234726	1.03679737	2.94459882	0.95916479	0.76386997
99.9	0.07279392	0.04580819	0.12025303	0.10398799	0.04784713	0.05653389
98.3	0.44382174	0.19501991	0.29075863	0.41002019	0.29184189	0.29638929
97.2	0.08871814	0.04552879	0.07624301	0.11034453	0.0415731	0.05977353
98.6	1.87413744	0.85664557	1.25128083	2.48428731	0.8358758	1.01536599
99.2	2.20068963	1.18618434	2.17063058	2.60755707	1.84321853	1.89618788
98.8	0.19322124	0.05380528	1.95574089	4.13219172	0.10144887	0.17338923
96.8	0.29838032	0.41186688	0.26402726	0.66937809	0.22449419	0.36208964
99.1	0.01314887	0.00920045	0.01767925	0.01256464	0.00901346	0.01439598
96.6	0.07105952	0.00141376	0.01983255	0.02271111	0.05805088	0.00862513
96.1	0.02022681	0.00979888	0.01513579	0.03066074	0.0073981	0.01159326
96	0.05277938	0.03035024	0.15521493	0.00164627	0.09879926	0.13021311
98.3	0.01427712	0.00923805	0.01090131	0.04780224	0.01109613	0.01077931
99.8	0.08931607	0.01463357	0.07395419	0.08671486	0.08854345	0.04402839
95.1	0.53376842	0.2316045	0.71972115	0.41345112	0.51273222	0.45778509
99.1	0.00442907	0.00145433	0.01665124	0.00302366	0.69247846	0.11873668
97.5	0.00988143	0.01106368	0.00929897	0.02774587	0.01031992	0.00756558
97.8	0.00027881	0.00020982	0.00920063	0.00448434	0.00060414	0.00044997
98.3	0.08375025	0.06423933	0.10512611	0.10862715	0.06032406	0.07313081
98.3	2.33196352	2.62502375	2.77141995	2.04814917	2.21412535	2.6178352
99.7	0.06236803	0.06422299	0.05638276	0.07483627	0.10275112	0.09100482

95.1	1.87966506	0.37078996	0.25600638	1.96164818	0.41692384	0.33432353
98.2	0.01702583	0.0101276	0.01692721	0.01596619	0.01167483	0.01519701
99.6	0.05903917	0.02945109	0.01028309	0.08898102	0.01816979	0.01453764
96.9	0.10617948	0.05732425	0.09459824	0.10297487	0.04843277	0.08633252
99.9	0.84281749	0.52302002	1.25866841	1.47905778	0.49487601	0.73532155
99.4	0.02366058	0.01825849	0.03169684	0.03420567	0.01565834	0.0170169
98.9	0.71009247	0.42370316	0.91130599	0.98831772	0.45041933	0.54932378
96.2	0.06530506	0.02837198	0.06206245	0.05949113	0.02699457	0.00164729
98.2	0.02785508	0.02363346	0.03394964	0.0395796	0.01679592	0.02694664
97.9	0.40285022	0.40213354	0.3844026	0.5075113	0.4253831	0.37680355
99.7	12.6500319	8.86433538	15.5723488	20.7889604	10.2245304	15.1005968
99.9	12.5042341	6.6091533	14.1265267	14.6884611	7.6478997	9.52568679
95.3	7.83983557	3.88337444	7.83794912	8.16701776	5.24988458	5.34241096
97.2	0.419435	0.26489268	0.419316	0.40241342	0.24403253	0.33006538
99.3	0.46524253	0.27951208	0.40721828	0.55215212	0.40539603	0.0468396
99.9	0.09914184	0.07394952	0.14228091	0.23455354	0.07331495	0.05662606
98	1.50719141	1.30847568	1.774807	2.66397614	1.44320626	1.40956147
96.5	0.52571195	0.36266878	0.60359988	0.32104713	0.3751086	0.51765453

Media	Media	TNFa500pg.n	TNFa500pg.n	TNFa500pg.n	TNFa500pg.n	TNFa500pg.n
M7	M8	T1	T2	T3	T4	T5
Media only c	Media only c	500pg/mL TN	500pg/mL TN	500pg/mL TN	500pg/mL TN	500pg/mL TN
44032	44032	44032	44032	44032	44032	44032
Sp07	Sp08	Sp09	Sp10	Sp11	Sp12	Sp13
0.55471733	0.00779111	0.31679318	0.4603339	0.14767778	0.66839094	0.01375298
0.02417192	0.00912647	0.03371037	0.06017197	0.06915097	0.04381135	0.04393371
0.06203305	0.03532171	0.05139373	0.10308051	0.07701794	0.06708699	0.05623945
0.05425022	0.03216419	0.09264326	0.22259458	0.09110683	0.0362307	0.089972
0.12004282	0.05287261	0.1182013	0.18450357	0.11440745	0.12005349	0.10273635
0.02141444	0.01212248	0.02716667	0.02613566	0.02851105	0.02595276	0.03020593
0.01997295	0.00869402	0.01910497	0.02218272	0.01846265	0.01697481	0.02364542
0.0154324	0.00783589	0.01336125	0.01711433	0.01338119	0.01678433	0.01119239
0.15350259	0.09054648	0.16568369	0.34358872	0.16189416	0.17339821	0.18366787
0.11443085	0.06598114	0.0737482	0.08601089	0.09215259	0.1172818	0.05122955
0.45471624	0.12051609	0.412411	0.32196304	0.3971169	0.32061851	0.22395133
0.00479044	0.006314	0.00741417	0.02957252	0.00722196	0.00473929	0.01676442
0.08310287	0.05694658	0.07083115	0.1010224	0.10604378	0.10874053	0.0556806
1.34783933	0.35915574	0.09540087	6.82560565	3.27228505	0.86183994	5.07303339
0.16521247	0.17955391	0.24067084	0.34922909	0.21893691	0.13976521	0.34355052
0.11484174	0.08601477	0.13663569	0.71359767	0.24948742	0.20097238	0.41950686
0.01138869	0.01209218	0.01669459	0.03894989	0.02966835	0.019	0.02081412
0.0255376	0.01379204	0.03692133	0.04282655	0.03250191	0.04290654	0.03380012
0.01460617	0.00379458	0.01071413	0.03872667	0.02305169	0.01666697	0.01813459
0.17616719	0.07240768	0.16975735	0.1994214	0.16732357	0.17215132	0.15611839
0.02559391	0.01120957	0.01868144	0.04580678	0.02712393	0.01505156	0.02406305
0.11249433	0.06565137	0.15299401	0.3078084	0.21352255	0.12043318	0.15266357
0.00682876	0.00519557	0.00809542	0.01378734	0.00863717	0.00770962	0.00743714
0.02653722	0.02730497	0.02251554	0.05522848	0.03161206	0.03936837	0.02621646
0.02157296	0.00477821	0.02210101	0.03779394	0.02943929	0.03556933	0.01526269
0.03484078	0.05334698	0.06124683	0.07842064	0.06161415	0.04258511	0.07422758
0.0682874	0.03851284	0.06805831	0.1160835	0.0650282	0.06241186	0.07691931
0.03063769	0.04198583	0.03866076	0.04762171	0.03724209	0.03845809	0.05444243
0.00814238	0.00371725	0.00907936	0.01278212	0.01126974	0.00738439	0.00797502
0.04735222	0.03906965	0.04927087	0.05851444	0.05510451	0.04958845	0.04774602
0.01456634	0.00964591	0.01584923	0.04234142	0.02196302	0.01710676	0.01883108
0.00671133	0.00604728	0.01451925	0.03442778	0.01154878	0.00577197	0.02090531
1.85997503	0.94847192	1.82252203	2.10464064	1.95110533	2.06755162	1.49636133
1.01923966	0.49655041	0.68468402	2.26930705	1.17185911	1.27253088	1.14239777
0.01263248	0.00594455	0.01757827	0.02114055	0.01290795	0.01612185	0.0235444
0.61579362	0.20446269	0.69670969	1.04468646	0.71470247	0.51081282	0.56808623
0.08405615	0.04690123	0.08500342	0.151804	0.12725252	0.15321954	0.12634241
0.01250806	0.00611754	0.01235428	0.02296578	0.01218385	0.01473526	0.01691283

0.3756302	0.13640745	0.35662849	0.56185331	0.29703683	0.38506904	0.35827665
124.348554	52.0615752	143.4367	175.091707	117.320052	115.104673	122.09269
0.00031464	0.0002843	0.00016152	0.02238478	0.00030251	0.00057604	0.00041952
0.33572304	0.15261533	0.29737399	0.44196165	0.34450169	0.29368283	0.37520256
0.01741766	0.00940752	0.0210292	0.02267814	0.0184318	0.02652192	0.0222686
0.02210698	0.0258077	0.01955334	0.01367483	0.01491281	0.1334972	0.01311367
0.01603202	0.00199712	0.00075292	0.0022918	0.01852741	0.00674601	0.00108914
0.0536953	0.02177151	0.04997009	0.01212597	0.05127026	0.06388032	0.06167045
0.03518001	0.0164793	0.03286423	0.04144184	0.03849753	0.04208675	0.02859037
0.13899778	0.03951122	0.14101568	0.0700488	0.13126126	0.12175518	0.06635126
0.00554886	0.00197877	0.00808446	0.0106412	0.00625038	0.0059885	0.00492316
0.45131945	0.1669053	0.34885764	0.21835185	0.18023749	0.19703142	0.14931834
0.41949571	0.13620959	0.33106781	0.4016235	0.42728945	0.31362506	0.35338906
0.2580043	0.11378094	0.21042758	0.30282912	0.27336611	0.25418988	0.22868565
0.32736648	0.22129824	0.38208149	0.56149476	0.42258017	0.49314754	0.43651951
3.46416683	3.36613589	4.01248769	6.41607099	4.48605875	5.23817426	5.96080718
0.13069083	0.07905943	0.13315724	0.19392369	0.11887576	0.10107828	0.11191504
0.00084561	0.00044493	0.00096835	0.00185894	0.00095486	0.00107416	0.00100959
0.01192725	0.00589649	0.00970037	0.01930109	0.01292093	0.0116815	0.01163554
0.27005264	0.10976068	0.33028807	0.49066978	0.28088675	0.3180047	0.28186195
0.28796412	0.12687369	0.32245998	0.59973123	0.23545711	0.17650429	0.42053231
0.06964037	0.04102022	0.14837775	0.22981415	0.18753958	0.19299212	0.13638292
0.03516007	0.00626838	0.03568624	0.07214608	0.02632147	0.06414996	0.02893674
3.48102671	1.08432468	1.0093608	0.86904386	1.30761886	0.94776757	1.66514199
0.71804291	0.34582134	0.63924933	1.10596814	0.59845689	0.61954475	0.61347645
2.49705194	2.43049281	2.13951123	2.20625697	2.8201968	2.42694302	4.44379073
30.5224512	16.5187665	28.6481073	41.8483304	29.4976895	28.341177	31.3190726
4.5700462	2.13907465	3.66396844	3.33216295	3.67926819	4.14323588	3.56557143
0.04606426	0.01223523	0.02499289	0.12005092	0.06857257	0.0689842	0.02794213
3.2885341	1.55737793	7.24037332	7.76798212	4.47384969	1.9387824	3.96050199
0.48648649	0.47958793	0.53126571	0.81501546	0.54501222	0.64461246	0.70016173
1.85383472	0.73991833	1.74390782	2.54487272	1.35025107	0.92159265	1.80139549
2.91783629	1.21264708	2.84568877	4.51814024	2.85645992	2.50799752	3.35830692
0.05802291	0.11428821	0.08805205	0.1212601	0.06144311	0.08779811	0.11814745
7.37294347	3.19426687	6.72239391	11.0472305	7.00224139	6.33551922	8.03406386
0.09236783	0.03442153	0.19638057	0.46243288	0.33444678	0.34374041	0.42046588
28.9103657	13.6161709	27.1052035	25.3014179	26.681777	29.7084042	25.5103512
0.00111281	0.00142482	0.00108311	0.07147643	0.00455495	0.00357835	0.01421268
0.05627351	0.01382064	0.05500579	0.0660241	0.07939265	0.05100459	0.05113833
0.03515804	0.00421071	0.00108038	0.00277028	0.04427484	0.01652224	0.00198629
0.04720596	0.01004296	0.04629024	0.01339029	0.02349975	0.0163683	0.01607196
0.0028448	0.00098511	0.00144328	0.00241354	0.00177317	0.00170768	0.0019783
0.08707427	0.023121	0.07723366	0.04223536	0.04183956	0.04585591	0.03645897

0.00442069	0.00102025	0.00147563	0.00878882	0.006972	0.00464585	0.00279385
0.01338484	0.00910006	0.01062894	0.01462464	0.01317029	0.01522201	0.01042148
0.22788913	0.08165509	0.31778467	0.43166958	0.30400351	0.21766508	0.26775184
0.00346457	0.00280285	0.00381149	0.00604723	0.00425478	0.00505406	0.00524517
5.98278727	0.6819081	4.38761008	0.73756806	3.61705483	0.52740541	3.78585659
5.5399122	1.86576325	6.5800714	8.73571145	10.1071378	11.3916563	9.85507446
0.01078202	0.00484553	0.01220262	0.03845537	0.01769151	0.01273206	0.01691253
2.36374869	1.16108121	1.7906891	1.45436604	2.12652951	1.66552629	1.52576233
7.19558988	3.67831389	9.10018228	8.00651173	7.44685602	7.46586853	5.59911186
4.18325302	0.00143724	2.34508618	4.1431048	0.78549232	5.46160356	0.18098908
0.02885238	0.02680349	0.06790967	0.0270598	0.03981467	0.03443357	0.8215561
0.00338779	0.00582454	0.00433878	0.00910007	0.00656746	0.00637071	0.00435906
0.12881153	0.00155017	0.04408442	0.09444779	0.01695397	0.1364333	0.01576942
0.00317359	0.00133436	0.00051198	0.00221048	0.00367942	0.00101721	0.00082351
0.03004402	0.01107298	0.02352436	0.04309477	0.02231087	0.02779873	0.03744906
0.00814695	0.00303322	0.00167693	0.00160162	0.04979824	0.01471205	0.00211504
0.00704889	0.00410652	0.00883967	0.03400593	0.00910676	0.01063799	0.011191
0.79991921	0.416499	2.02012401	5.71352138	0.80832794	0.1940793	3.02411687
0.26710296	0.10308625	0.32893728	0.47611886	0.52092267	0.66198543	0.53405386
0.9423578	0.59644852	1.33808421	1.14998051	1.07638385	1.10934539	0.82898645
0.14956061	0.05958387	0.13612776	0.19625163	0.11335539	0.12655723	0.14668819
0.45094304	0.21531558	0.55544648	0.93375168	0.54461422	0.33335237	0.5617828
0.08158411	0.04690369	0.15250266	0.26887511	0.16675214	0.12448802	0.15765035
0.32498599	0.16656375	0.27693429	0.16269146	0.28061222	0.2729411	0.19628223
0.85784507	0.40025387	0.692378	1.03596597	0.97341553	0.80935271	0.76739021
0.00453533	0.00294335	0.00535285	0.00852862	0.00642716	0.00747505	0.00910913
53.9260871	29.5452971	46.5925003	72.9056704	59.7423707	53.8323576	49.6559513
0.12412315	0.04752155	0.16286035	0.16853395	0.15988222	0.08607718	0.17405485
0.0289199	0.01461196	0.72079953	1.06230111	0.71529905	0.00206511	0.76356365
40.210831	13.6259005	20.116349	31.3563584	25.8157116	39.663523	22.0907455
0.24519253	0.09076815	0.24346748	0.26538428	0.22658532	0.24210244	0.20322494
1.6542241	0.70257107	0.74116213	0.80645446	1.09332147	1.11844616	0.86143238
46.8901965	11.6006537	66.7613783	0.75083664	53.7340442	0.7533995	0.48101509
0.28820467	0.1332237	0.20986162	0.11377173	0.14947464	0.18056201	0.1252917
0.02061102	0.00798081	0.04580243	0.02231256	0.04996434	0.03647654	0.01724522
1.13948459	0.72549644	1.52198786	3.91272268	1.27513651	1.0471049	1.98189328
0.00346955	0.00443325	0.003728	0.00573906	0.00463757	0.16478725	0.00477546
0.02604355	0.01921029	0.02048087	0.02050379	0.02725138	0.02546652	0.02249792
16.5074914	5.96647629	10.0349403	11.3593971	15.0031952	12.2685645	12.0810337
1.00328399	0.4079219	1.03583804	2.00888531	0.85915988	0.76673085	1.20442589
20.1465374	8.56056038	17.8785032	15.5377725	18.3689452	16.3750896	14.2290061
9.89869887	5.67167594	12.861618	20.2160962	13.8793094	12.1694665	13.1442243
0.26167686	0.12645278	0.19971707	0.15077237	0.19890819	0.19177899	0.14908027

19.4009142	7.53219297	17.9557445	17.6175105	21.1321775	16.9390702	13.5282997
0.00807377	0.0066052	0.0082192	0.01373311	0.00861532	0.01215572	0.00703146
1.13678433	0.51917928	0.89245372	1.13258894	0.86422688	0.89361291	0.84875011
56.6703963	15.9511897	45.7844287	51.6351386	55.3172957	55.9408836	44.6804787
0.16572342	0.06936337	0.16935917	0.19817027	0.14296835	0.16699805	0.13724154
0.02926024	0.01571874	0.02786058	0.03387447	0.02785125	0.03312629	0.02452387
0.01650678	0.01148013	0.0300194	0.05013373	0.03148792	0.02451665	0.03239506
0.21927542	0.09892454	0.32604318	0.65085075	0.21243932	0.15303086	0.32939564
0.01905059	0.01154415	0.02064074	0.03589925	0.02457284	0.02738221	0.02349135
0.09710967	0.01140059	0.00868928	0.02748286	0.16964801	0.05098268	0.01110902
0.77653695	0.53312366	1.24000042	2.1357946	1.21233363	1.47305235	1.36715381
0.05311935	0.01191365	0.00966668	0.01450077	0.04819253	0.01923506	0.01697532
0.03387306	0.02765264	0.08787807	0.17381323	0.06996032	0.20553308	0.19332963
0.00808187	0.00603155	0.00628419	0.00147889	0.0009853	0.00333963	0.00145277
0.0615925	0.02515482	0.06576669	0.08149653	0.07112525	0.05124972	0.05576196
0.03467924	0.02192562	0.03681889	0.09402923	0.0377748	0.04591166	0.03972533
0.00922596	0.00190879	0.00435414	0.00310675	0.00082309	0.00354889	0.00051933
0.02457218	0.01443638	0.02124221	0.02231573	0.01565096	0.01816404	0.01978485
0.21476078	0.10544442	0.24189058	0.29568742	0.23320012	0.22454075	0.26903912
19.4967034	8.40324585	14.3324553	25.9378517	22.8669711	17.6707003	14.5251851
0.18150879	0.07511268	0.1342792	0.23501972	0.22824284	0.13246129	0.16639678
0.02420437	0.04427063	0.02017458	0.02792864	0.02973845	0.02894216	0.02727836
0.75523557	0.45114539	0.71463631	1.52304296	0.96179982	0.88407973	0.9508381
0.06417633	0.09390175	0.04994143	0.05590914	0.06502512	0.08223701	0.06275498
0.00526118	0.07689911	0.27499211	0.31726671	0.17852732	0.24234419	0.18214361
0.04355356	0.01718744	0.04480818	0.060924	0.04478476	0.04255971	0.03599139
1.05143151	0.4773893	1.09949786	1.47349293	0.99469801	0.85940277	0.83041176
1.89920567	0.97288899	1.83231401	1.8754433	1.43979604	1.37890913	1.70708554
0.03678364	0.06111383	0.45346603	0.07880063	0.05564023	0.14147969	0.1591028
0.20128276	0.1472094	0.18126213	0.46577653	0.18286654	0.42477719	0.24910828
0.01240129	0.00615697	0.00918039	0.01606052	0.01253531	0.01012313	0.01136271
0.00107058	0.00320417	0.02911694	0.00037738	0.03757756	0.01520199	0.00025004
0.01214391	0.00448549	0.00947188	0.03476655	0.01051838	0.01620655	0.01038052
0.10129619	0.04303899	0.05132495	0.00106525	0.12316559	0.11882853	0.00031611
0.00699585	0.0031587	0.01004144	0.01032388	0.01031332	0.01125175	0.00894689
0.04987963	0.02345361	0.05529513	0.01695773	0.014823	0.04064482	0.05293954
0.56632449	0.13111904	0.5665303	0.43462153	0.58519541	0.44954138	0.46110544
0.0181115	0.00079271	0.02922441	0.00263213	0.19822085	0.00075352	0.17385328
0.00683882	0.00140404	0.00574605	0.02077402	0.01082711	0.00744059	0.0060174
0.00322205	0.0018653	0.00025947	0.00227703	0.00019493	0.00421447	0.00235552
0.08923364	0.13458364	0.07906489	0.08136212	0.0894638	0.13062474	0.07317071
2.10266452	1.89893159	2.38850113	2.93232887	3.01886414	5.55716327	2.78341912
0.0625656	0.05410407	0.04864895	0.05671911	0.09361253	0.11348739	0.03871616

1.19357628	0.30158294	0.10016413	0.13507532	2.33058711	0.56051305	0.16416817
0.01137348	0.00642703	0.0088028	0.016654	0.01174098	0.01060092	0.01256449
0.03013398	0.00362546	0.00306623	0.00880152	0.03782738	0.01845947	0.00399187
0.0838374	0.04805654	0.06243857	0.04398952	0.08651246	0.0003369	0.03708969
0.51637983	0.24944228	0.52143194	0.90605226	0.65801526	0.54395094	0.62536294
0.01572397	0.01138798	0.01447895	0.01952576	0.01658502	0.01704167	0.01627973
0.50935961	0.24842841	0.48005961	0.50850983	0.61351138	0.4735535	0.43122977
0.04858267	0.0304086	0.03115293	0.03996244	0.05094054	0.05878225	0.0242922
0.03221973	0.03230435	0.03022521	0.01306925	0.02553396	0.03630293	0.01716454
0.35209909	0.79142775	0.48887875	0.38492231	0.4572237	0.50329456	0.39468714
8.55874573	5.2449138	8.94276596	16.0346441	11.2290263	8.99289063	11.0126725
9.37836248	3.9154998	7.8409012	8.74162047	8.64979624	7.95736747	7.47532364
5.68943098	1.83083683	4.99493873	4.32634663	5.48402624	4.62565555	3.61164173
0.30138691	0.23842004	0.30351409	0.40199317	0.29321691	0.32081267	0.25263013
0.36080115	0.16688894	0.35615999	0.38377295	0.30840017	0.26055763	0.30165844
0.06892463	0.04811767	0.03872214	0.06400805	0.04496729	0.05655217	0.04147564
1.28913089	0.96969013	1.31658189	1.67995356	1.43765247	1.49559931	1.52714893
0.61495807	0.3134476	0.56784388	0.41684009	0.39082951	0.49707658	0.39910324

TNFa500pg.n	TNFa500pg.n	TNFa500pg.n	Butyrate500u	Butyrate500u	Butyrate500u	Butyrate500u
T6	T7	T8	B1	B2	B3	B4
500pg/mL TN	500pg/mL TN	500pg/mL TN	500µM Butyr	500µM Butyr	500µM Butyr	500µM Butyr
44032	44032	44032	44032	44032	44032	44032
Sp14	Sp15	Sp16	Sp17	Sp18	Sp19	Sp20
0.05940985	0.01665791	0.22356312	1.24145504	0.21847884	0.32787324	0.04537349
0.06482396	0.06486564	0.02716867	0.06338874	0.02045255	0.0904223	0.03735325
0.15056148	0.08697819	0.05987263	0.0857537	0.05725064	0.11264572	0.08149182
0.21925856	0.05745669	0.07644218	0.15260083	0.00407214	0.14004583	0.11197257
0.2500603	0.15483512	0.0916033	0.17312998	0.0743283	0.15534845	0.13620448
0.0349459	0.03001041	0.02312222	0.03221098	0.02062373	0.02676093	0.03012279
0.05038002	0.02561704	0.01499381	0.02910721	0.01246806	0.02644874	0.02464789
0.01802657	0.01653297	0.01037628	0.0179978	0.01122371	0.01968909	0.01043499
0.36664791	0.21130657	0.14701677	0.23939621	0.13908625	0.25710955	0.24717448
0.04218457	0.08662051	0.057065	0.06997851	0.10240917	0.07715912	0.06336702
0.57602015	0.45931768	0.19106703	0.67290085	0.19122346	0.89126551	0.4968022
0.04549258	0.0065307	0.00841738	0.01818075	0.00876794	0.0254878	0.01696305
0.15506675	0.12582965	0.07830342	0.11378966	0.08586427	0.11930017	0.08440316
3.89054401	2.15817294	1.07396721	5.8599338	1.36756914	4.52390609	1.60897553
0.50652571	0.26170852	0.1881856	0.35878991	0.18749217	0.43620946	0.31821608
0.6459865	0.20589722	0.2667268	0.18219767	0.19656397	0.43774288	0.23473273
0.04941658	0.02544753	0.01859107	0.04374174	0.01941202	0.04789938	0.05205548
0.06941319	0.04531623	0.04756057	0.04364001	0.01808244	0.03197868	0.04906505
0.04467118	0.01613617	0.0149285	0.02012029	0.01352558	0.02898705	0.02834443
0.26447509	0.29211853	0.20030446	0.23991992	0.09213148	0.23949798	0.22963216
0.06414699	0.02563767	0.0201082	0.03599178	0.01813187	0.03023106	0.034257
0.37934098	0.17301207	0.13030716	0.26926272	0.12396315	0.24090473	0.19930858
0.0218997	0.01036094	0.00570748	0.01163139	0.00691991	0.01505345	0.00782538
0.0839859	0.03727946	0.03105956	0.03202868	0.03129123	0.03764111	0.04507937
0.04629834	0.02459948	0.01456547	0.02220511	0.00936274	0.03827601	0.02615208
0.00679107	0.07077758	0.05109305	0.09525576	0.05303773	0.12030521	0.08741666
0.15072307	0.09022548	0.05857263	0.09924835	0.05738796	0.10258299	0.0896138
0.111845	0.03718682	0.03940045	0.02254428	0.0278861	0.04220854	0.03478603
0.02022551	0.0087482	0.00539058	0.01149084	0.00070757	0.01163297	0.00882283
0.08166206	0.05394481	0.03287961	0.06149452	0.0374861	0.04768367	0.04866414
0.04363122	0.01981625	0.01346409	0.0269091	0.01539366	0.02615218	0.01850388
0.03449864	0.01140897	0.01321715	0.02394413	0.00852408	0.02278965	0.02119591
2.9519129	2.28529091	1.46840768	2.52510212	1.37617634	2.87231681	2.27360339
1.43423711	1.16664206	1.15985147	0.57732191	0.42868171	1.9154607	1.41330065
0.03073191	0.01985813	0.01004662	0.01814234	0.00543863	0.02512952	0.01647762
2.04909736	0.80508539	0.4726384	1.15763212	0.3215908	1.41944785	1.10880859
0.12200085	0.10751668	0.10894085	0.08587177	0.11992982	0.12819438	0.1068126
0.03399538	0.0143173	0.01151833	0.01157036	0.0064996	0.01928696	0.01473009

0.79040081	0.35947764	0.30792081	0.64598671	0.23089073	0.4943374	0.40296586
311.04715	130.148162	89.1396356	210.723578	72.744061	197.840018	168.401882
0.00989041	0.00024998	0.00251621	0.00021275	0.00039723	0.00031429	0.00029524
0.93866477	0.4845305	0.24559095	0.33151284	0.15087561	0.47148482	0.36871261
0.0521393	0.02202153	0.01617318	0.0280767	0.01298252	0.03597	0.02543749
0.0590804	0.02051307	0.0122039	0.011227	0.00552105	0.032203	0.01695262
0.04957812	0.01393494	0.00805494	0.01274146	0.00234957	0.01494292	0.03313307
0.15900698	0.06176769	0.05859098	0.05721888	0.03912584	0.09164148	0.13935435
0.0625668	0.0436477	0.02056902	0.04297451	0.02963783	0.05780004	0.03798045
0.13695291	0.09185265	0.07642317	0.4288894	0.07590765	0.1119717	0.15468641
0.007801	0.00934674	0.01181067	0.00683425	0.00158515	0.00768719	0.00662592
0.35629537	0.26886798	0.21651077	0.29992152	0.23907981	0.2231648	0.21262112
1.0520331	0.5712913	0.21431765	0.58014841	0.23064416	0.90044455	0.46357978
0.55580751	0.30976213	0.19531678	0.31702074	0.20427434	0.43679844	0.28795166
0.76396652	0.55254744	0.31450794	0.45479979	0.31518247	0.48894088	0.35268126
8.81804275	5.45189198	4.88883191	4.00938209	3.82415786	4.65266293	5.30564149
0.3114315	0.17938445	0.08314105	0.16885172	0.07416308	0.22399069	0.11600076
0.00221222	0.00108033	0.00076911	0.00102472	0.00070726	0.00145983	0.00103027
0.02373354	0.01283139	0.00858077	0.0206642	0.00818163	0.01964003	0.01179936
0.79632326	0.37582548	0.21716876	0.42238666	0.15380124	0.43522395	0.3249979
1.24429763	0.45534238	0.15778927	0.40613769	0.11172667	0.63884689	0.30555144
0.36215204	0.20111613	0.11648663	0.13081881	0.09738944	0.19447687	0.12261043
0.1230894	0.03426568	0.09770188	0.06987968	0.0162569	0.05148981	0.0532588
4.36858896	2.62200461	1.25138973	1.41861002	1.5026722	1.59437934	1.35681584
1.49561242	0.76429182	0.41387008	0.82345658	0.42421749	1.09461655	0.72238171
7.11559216	2.86657457	2.6872552	3.19387697	2.3010081	0.36475028	3.16302974
70.1853506	38.2666797	23.5460884	39.601209	21.2855822	46.1544177	37.8553034
6.30167166	5.13086513	2.52937469	2.40631995	2.7864117	4.97961161	3.49054224
0.16611777	0.04730964	0.07069245	0.08016063	0.03061749	0.08895153	0.06753792
18.0608626	4.47547679	4.1364423	6.62238661	1.83722887	8.28921561	4.01778837
1.00755301	0.6045872	0.54869983	0.50129916	0.44919284	0.65153509	0.57216268
5.50553115	2.29171081	1.75647875	1.98897473	0.91467378	2.34653998	1.4741725
7.80018538	4.15532757	2.18264241	2.83789773	1.45333004	3.13520237	2.1592082
0.17812146	0.0909862	0.10106493	0.0701519	0.07369817	0.04353572	0.07120532
18.7126525	9.82207905	5.19243833	7.7956148	4.0246915	9.89099299	6.64219454
0.62315846	0.39069966	0.23175188	0.32257424	0.12296398	0.28758894	0.33926132
36.3772109	36.5938033	19.4002578	27.0938764	19.3029046	29.9423992	25.2664898
0.01985619	0.00218735	0.00576616	0.01050191	0.00394038	0.02462635	0.01109869
0.1223145	0.09558454	0.02169336	0.04723738	0.01922237	0.08432262	0.04763814
0.05088983	0.03660305	0.01391736	0.02620284	0.00545829	0.02061182	0.07266466
0.02609283	0.02535463	0.03176413	0.03440209	0.00029728	0.01857316	0.02919471
0.00237928	0.00545458	0.00195539	0.00217456	0.00066412	0.00211217	0.02201794
0.05912748	0.0586947	0.06958422	0.07060146	0.04572596	0.03808914	0.06211307

0.01119473	0.00317979	0.00569209	0.00583896	0.00261451	0.00444316	0.00543874
0.03283291	0.01734629	0.00973897	0.011141	0.01022849	0.02180936	0.01654067
0.81995687	0.31437657	0.18682362	0.51349422	0.12043084	0.60863214	0.48824148
0.00851549	0.08213106	0.08763893	0.00386727	0.0034313	0.00502413	0.09139123
5.47860428	5.43507461	2.49633335	3.42682892	2.49138679	0.86503481	0.35872519
9.04748905	8.44656048	5.42553647	7.64095461	4.5231779	6.76784819	5.7508604
0.04813327	0.01396978	0.01185119	0.01564595	0.01020992	0.02383037	0.01625476
1.65727265	2.25119365	1.4461731	2.75994931	1.86209397	2.6431259	2.34860693
12.7591851	8.53498296	5.63955772	10.5552532	4.90010341	10.1275103	9.54271589
0.01870981	0.00313291	0.9020537	19.4990004	1.51170434	3.51001019	0.03221262
0.06586625	0.05190097	2.12881549	0.02587305	0.01137385	0.07576304	0.04108952
0.00230388	0.00644851	0.01047833	0.02342189	0.01331841	0.01065964	0.01040203
0.00783914	0.00417195	0.1195628	0.47273182	0.04423714	0.10392651	0.00634451
0.00379059	0.00260145	0.00079473	0.00078146	0.00083537	0.00122088	0.00228668
0.07612731	0.02977371	0.01650552	0.04167021	0.01499518	0.03825876	0.03078772
0.02156457	0.02256773	0.00862769	0.00860102	0.00353986	0.00813997	0.03763834
0.02693252	0.00907766	0.00919217	0.00829516	0.01085974	0.02055468	0.00943451
1.10672496	1.56151024	1.3941489	4.41421049	1.01117682	0.19282952	0.02144171
0.47554906	0.42840561	0.29342804	0.3617774	0.12890939	0.33312455	0.32204779
1.44311355	1.17031932	0.6069308	1.18595067	0.0352589	1.43339275	1.10724549
0.30720429	0.17102766	0.12523772	0.14165695	0.0722441	0.18113754	0.10678833
1.71732816	0.55832256	0.32761715	0.78267077	0.17263676	0.93784291	0.6724622
0.41311651	0.20398057	0.13751173	0.26313377	0.058615	0.29403125	0.24956209
0.18319104	0.28883225	0.19897453	0.21714779	0.22059186	0.00318297	0.22875911
1.42696013	1.00126217	0.60854873	1.0193212	0.60116253	1.53350105	1.12610854
0.01368698	0.00696768	0.00631902	0.00637121	0.00678517	0.0064359	0.00642826
98.6153047	60.081323	41.5328069	66.7906401	37.1124411	86.0866366	64.385043
0.5773236	0.28710732	0.12525446	0.32025139	0.07689931	0.36975225	0.25403159
1.71889083	0.79802354	0.5420612	0.82812035	0.04113706	0.93942894	0.76572144
40.4588866	31.2546707	18.9235159	17.9202386	21.3734325	36.7108504	30.2769759
0.34012656	0.2123775	0.22911318	0.27317173	0.1843736	0.28199222	0.30805876
0.57720343	1.36213888	0.80121768	1.38744946	1.25051092	0.94145057	1.23330459
115.856776	0.88150602	0.51987962	0.67095671	0.47991433	0.28101455	53.6788126
0.26594484	0.19662669	0.22994898	0.0235494	0.24857122	0.13759565	0.17205578
0.03192665	0.05337845	0.01190678	0.04797222	0.00932324	0.03902188	0.03088213
8.23888108	1.72683064	0.74940629	1.80998432	0.3846109	3.32169412	1.84186096
0.1006851	0.00390931	0.00405639	0.00332297	0.0643999	0.00371908	0.00341074
0.02341718	0.02577569	0.02100443	0.0316676	0.02037732	0.04470805	0.03309391
17.443318	15.4111553	7.54052683	12.5785085	10.6046999	19.1842989	13.4105861
3.0223637	1.39341403	0.65564341	1.32173924	0.35581844	2.25849558	1.22943822
20.7588884	20.9449061	13.0957136	22.3580257	13.8144781	24.6297313	19.6647471
30.0369779	14.7191905	9.30770528	15.9344055	7.69736532	20.8316513	15.9470436
0.214969	0.25972059	0.10402246	0.23952275	0.18733852	0.2586802	0.19391455

25.8058064	22.2754025	12.6617166	24.2909026	11.1684158	26.3745617	27.1725888
0.01316082	0.01433904	0.00985264	0.01209295	0.00957623	0.01171178	0.00945737
2.5058439	1.23381239	0.79938339	1.10791752	0.59046121	1.28323194	0.95844603
89.4807533	61.4530197	29.7781151	56.1139669	22.9298635	71.7655028	46.1144705
0.28373976	0.18742602	0.12916337	0.12021301	0.0925863	0.15095936	0.10760853
0.04174835	0.03728606	0.03004372	0.0319119	0.0342151	0.0405649	0.04157082
0.07521398	0.0471702	0.01945427	0.04008622	0.01457701	0.05026677	0.04934749
1.95397179	0.31185749	0.11314416	0.40779413	0.02955752	0.62003369	0.33296343
0.03879245	0.02893921	0.02189224	0.030154	0.01742763	0.03079098	0.02816125
0.19620458	0.07825909	0.04336935	0.06370736	0.02694123	0.05524655	0.1584819
1.58095266	1.49719159	1.12259166	1.19922689	0.95768613	1.63738562	1.27841766
0.06422172	0.02660926	0.00567821	0.01186027	0.01044279	0.0218902	0.02243963
0.15282136	0.05966241	0.05110358	0.06796007	0.04241183	0.10193059	0.03073315
0.00221999	0.00189946	0.01220616	0.00515445	0.02436575	0.00061474	0.00179732
0.09354836	0.06567145	0.04990808	0.08952511	0.04647894	0.05667098	0.05968265
0.08600005	0.04259805	0.04114713	0.05124842	0.03284041	0.06273792	0.0553988
0.00152593	0.00109205	0.0133745	0.00126905	0.00111641	0.00499185	0.01073714
0.03517436	0.02475415	0.01642938	0.01954308	0.01644796	0.02022785	0.01604965
0.32707455	0.30094019	0.10245381	0.17746086	0.08182334	0.24239164	0.1242467
45.1945993	23.5509119	14.8396112	24.01878	12.4372848	31.5568165	28.5296535
0.61140523	0.2619005	0.12397559	0.20616347	0.1154652	0.34044457	0.27775029
0.0307232	0.0194825	0.0257372	0.02351555	0.03524036	0.02854236	0.02726349
1.81623146	0.92144348	0.92403349	0.77106486	0.71403184	1.10030272	1.20817943
0.06781841	0.05410618	0.05673389	0.05706256	0.0677331	0.0732206	0.06383496
0.47236882	0.25671588	0.14076698	0.22964824	0.1069704	0.26629355	0.17261721
0.10769871	0.05016853	0.03129066	0.06017477	0.02621889	0.07111022	0.04742523
2.31206375	1.02314358	0.83274686	1.47449258	0.61395287	1.48555104	1.06082998
3.15889549	1.65936804	0.93905897	2.26079621	0.89104572	2.11639749	1.33894978
0.66568246	3.01700606	0.01946253	0.17856653	0.43289039	0.17482358	0.80927854
0.39508226	0.28822222	0.31391359	0.43846671	0.30542718	0.39181872	0.46071636
0.01797429	0.0112259	0.00883665	0.01276378	0.01004914	0.0151842	0.01612264
0.0007669	0.04121365	0.00032393	0.01049352	0.00281471	0.00085389	0.00309477
0.02044608	0.0110605	0.0096905	0.01411583	0.00726112	0.01752289	0.0110737
0.15994026	0.00825151	0.06037569	0.10561172	0.06279928	0.00130366	0.10669602
0.01366946	0.01029398	0.00764328	0.01478507	0.00191238	0.02656864	0.01452363
0.12821859	0.02149181	0.02193557	0.01795129	0.05399132	0.06234517	0.06698559
1.03066968	0.87246634	0.28935025	0.47702542	0.20512881	0.56030199	0.38077908
0.07562707	0.0273252	0.00231748	0.21698659	0.00187874	0.10054253	0.01176757
0.01589724	0.00975089	0.00761712	0.01219207	0.00634895	0.01573215	0.00915736
0.00052029	0.00156701	0.00021298	0.00034307	0.00138194	0.00049239	0.00048061
0.07857637	0.0710476	0.07822558	0.07837382	0.11829098	0.11057188	0.09514062
2.07021112	2.4854021	3.17538111	2.00699842	4.77435795	2.23091406	2.07323902
0.04798303	0.05714127	0.0957372	0.06733689	0.20591699	0.07346463	0.08112441

0.86103778	0.7785996	0.15841798	0.42892477	0.31252422	0.4083631	0.75547657
0.01860933	0.01190738	0.00929817	0.01404854	0.00925399	0.01527207	0.01057485
0.0640972	0.02287472	0.01758593	0.0234852	0.01063024	0.02131021	0.04913817
0.03765167	0.06050345	0.05436255	0.06252865	0.08409089	0.00078551	0.04994757
1.16048238	0.68070611	0.3454983	0.75980079	0.39143163	1.02795948	0.70153957
0.02582301	0.02041381	0.01419309	0.01410421	0.02328367	0.02768793	0.02289958
0.94851321	0.57672413	0.34293383	0.66076339	0.28275785	0.85047758	0.51885743
0.01956772	0.00085758	0.03037895	0.03207165	0.00060361	0.03152636	0.02501131
0.01396045	0.03188779	0.02610346	0.02358032	0.0389659	0.02303507	0.02320008
0.44306526	0.29651183	0.15317461	0.51059322	0.49238182	0.48186333	0.45275234
25.7175012	11.5280281	7.93287193	9.8292759	6.55563978	12.9641003	5.35631289
12.9384478	10.1996586	6.10970817	9.93360507	6.2068022	12.8569823	9.80476976
8.92614022	8.59634912	3.9854743	8.93012661	2.77105946	9.14740155	8.47883597
0.41658051	0.32845436	0.25737768	0.35171842	0.26716097	0.3486136	0.36025182
0.71619409	0.42023214	0.25459047	0.47387522	0.18470309	0.41265017	0.32526534
0.06089132	0.03739746	0.0264849	0.06539932	0.09459659	0.08139575	0.07158135
2.68445277	1.38262499	1.19627719	1.33673819	1.05078227	1.5757217	1.59278395
0.70716897	0.4754769	0.55749103	0.43460133	0.48425008	0.36637311	0.49607436

Butyrate500u	Butyrate500u	Butyrate500u	Butyrate500u	TNFaButyrate	TNFaButyrate	TNFaButyrate
B5	B6	B7	B8	C1	C2	C3
500µM Butyr	500µM Butyr	500µM Butyr	500µM Butyr	Combined 50	Combined 50	Combined 50
44032	44032	44032	44032	44032	44032	44032
Sp21	Sp22	Sp23	Sp24	Sp25	Sp26	Sp27
0.03578272	0.80312003	0.28766453	0.02795791	0.03076857	0.01103337	0.02051957
0.06027562	0.06034765	0.07209489	0.01012157	0.03806593	0.01304066	0.12138734
0.11339779	0.11405137	0.11347189	0.03904309	0.10019786	0.01455375	0.15502189
0.10576209	0.10568731	0.07862974	0.04535734	0.15287697	0.17531726	0.23180368
0.14418868	0.15963258	0.14170543	0.04337158	0.1251254	0.03815795	0.20014611
0.03187989	0.03266262	0.0320614	0.02264351	0.03446522	0.04112146	0.08020637
0.03204255	0.02454501	0.02550221	0.0097553	0.02280585	0.01669727	0.05037175
0.01428282	0.01828715	0.01660985	0.00369687	0.01219871	0.0007239	0.01534266
0.20184655	0.20319244	0.19460549	0.08815841	0.26743798	0.13583382	0.42984382
0.08559217	0.09626205	0.08057527	0.05781456	0.07057354	0.03479815	0.10020007
0.44060334	0.57707929	0.74801027	0.13292774	0.50400791	0.13540864	0.84577326
0.01252638	0.01254124	0.00871391	0.01040686	0.0277809	0.04888367	0.04719692
0.11853444	0.15693672	0.13266403	0.03593682	0.14128473	0.02438015	0.14948294
2.31950232	1.902001	3.6471167	1.62601927	1.89236558	5.01271196	2.59861378
0.4465518	0.42670725	0.27179212	0.1354561	0.34368752	0.23533051	0.40774834
0.19090927	0.22637009	0.15109188	0.14846927	0.24578923	0.31721177	0.16673824
0.05353359	0.04237001	0.03499261	0.01041669	0.09512216	0.01210291	0.04677021
0.04689842	0.05884452	0.03729254	0.03342576	0.03758849	0.0548501	0.10792802
0.02595217	0.02176107	0.01693358	0.0166027	0.05316265	0.06209317	0.05158366
0.20730252	0.21024291	0.22279812	0.06233297	0.18750811	0.08384165	0.40535886
0.03487461	0.02899814	0.02127275	0.01667833	0.03608315	0.01663573	0.04040515
0.21747476	0.18407802	0.17044798	0.0670928	0.25419066	0.14439344	0.36894278
0.01083587	0.00995077	0.01050687	0.0025808	0.00996619	0.0043216	0.02163426
0.02831342	0.05208944	0.03005447	0.01890879	0.02620363	0.01769868	0.0541541
0.02504438	0.0304927	0.02872093	0.00978489	0.02330179	0.01718815	0.08708758
0.13938516	0.10706374	0.06357576	0.02835109	0.09521391	0.06028325	0.10681016
0.09878098	0.10013533	0.08502398	0.04610136	0.10117374	0.05046541	0.1219035
0.02607457	0.03280948	0.02991867	0.02558531	0.10852753	0.05746544	0.12358025
0.0088138	0.00885264	0.01119666	0.00368296	0.00897395	0.00677464	0.00288241
0.03724234	0.05229238	0.05237914	0.02637172	0.05490965	0.03088247	0.06484833
0.02409543	0.02133182	0.02058646	0.00968118	0.02234145	0.01212259	0.05114334
0.00751506	0.01235074	0.012867	0.00799432	0.0271385	0.02298654	0.03954192
2.30424374	1.28040259	2.35714438	0.75742285	2.14493298	0.64839426	4.22702379
1.38163872	1.28884766	1.21273058	0.0664986	1.25545483	1.34675417	0.82156983
0.02257481	0.01716467	0.01870056	0.00883923	0.01993684	0.08265245	0.03729203
0.93656982	1.04530272	1.01374713	0.23816211	0.97887783	0.51864709	1.82846017
0.10818445	0.1105158	0.10959246	0.10367286	0.13426237	0.25434386	0.22351741
0.01540833	0.01626892	0.01596452	0.01018292	0.00747423	0.01391786	0.03223668

0.32849763	0.40360696	0.43683808	0.29972949	0.41401625	0.57679092	0.77229459
145.68094	136.06706	140.400364	55.8705017	177.226089	136.901626	323.707208
0.00032088	0.00537505	0.00026333	0.00022604	0.03751297	0.00038454	0.00061134
0.45350047	0.48056113	0.4054273	0.11745514	0.39825666	0.22425466	0.94492273
0.0299992	0.02970435	0.02480793	0.01215669	0.03133097	0.01922136	0.04826739
0.01354368	0.02401122	0.042314	0.01672985	0.01931618	0.03945219	0.13700158
0.01874334	0.05546572	0.01202009	0.01086902	0.01763529	0.00070231	0.00155148
0.10633862	0.09431167	0.08065695	0.03775576	1.10670341	0.04539673	0.14684255
0.04768735	0.0537749	0.05138782	0.0116442	0.03163626	0.01107327	0.05951425
0.09459074	0.07985731	0.08575959	0.09418328	0.16514932	0.13951914	0.21163253
0.01540779	0.0085772	0.00799398	0.01317509	0.00382217	0.01141457	0.00737105
0.25318782	0.2386036	0.24450786	0.25073566	0.19118078	0.11843694	0.2553459
0.52291535	0.67305638	0.63221347	0.17128063	0.56941831	0.19448888	1.22593257
0.3398162	0.39027319	0.32640391	0.13346813	0.28909485	0.18152588	0.66827307
0.43991963	0.44563786	0.44459504	0.23745121	0.38700647	0.33192669	0.9508122
6.35891299	5.8420434	5.23328472	3.28858337	5.56740727	4.77238471	7.7698141
0.14274936	0.30316966	0.17345803	0.09107374	0.16314678	0.18166099	0.2564477
0.00114823	0.00202837	0.00113418	0.00061528	0.03959102	0.00152127	0.00252973
0.01468505	0.01536638	0.01391492	0.00551569	0.01286509	0.0074064	0.02787534
0.27916395	0.35299204	0.4067545	0.15641605	0.31633758	0.20823999	0.58525374
0.42877308	0.55213101	0.46856904	0.07977427	0.33894861	0.223352	1.0312744
0.17896032	0.19463569	0.16054172	0.05621484	0.23172182	0.1195759	0.51774646
0.03809064	0.04454517	0.0590643	0.05054602	0.06949242	0.06194963	0.11261998
0.90651793	0.52837139	0.96920105	0.61736594	2.62561175	1.48374804	5.44701447
1.11047375	1.05606612	0.86082871	0.22536508	0.99213039	0.44154641	1.53240752
3.26309443	2.74319408	2.9978141	1.70556163	2.81057686	3.60330025	4.01162298
42.2432651	43.04217	39.1398947	16.7217643	35.9411004	20.0269552	76.0877858
4.16542613	4.50794192	2.72069909	0.9187677	2.32036688	1.70518128	5.97045396
0.0443799	0.06855725	0.09547985	0.03611857	0.09149299	0.01877148	0.1039277
5.80283712	5.23519274	6.55555293	3.27447892	4.60695137	6.61067769	10.2694884
0.64922337	0.60758671	0.598237	0.35888554	0.82864569	0.49958272	1.18506629
1.5756515	1.89146765	1.81637853	0.66854692	1.72447151	2.16907537	3.89601877
3.15679707	3.46021985	3.10087332	0.71072746	3.28449108	1.11645484	5.92839263
0.09979722	0.08867499	0.09404235	0.06195721	0.11083238	0.07186589	0.10195597
7.87161594	9.17798851	8.12971747	2.05011578	8.02200909	2.98712867	15.6476033
0.30191908	0.21247522	0.22268649	0.09255554	0.18318136	0.4269628	0.40883942
25.3155924	28.8230456	28.4920398	11.6355571	24.92015	10.884863	43.6527652
0.00772435	0.00852145	0.00275658	0.0186811	0.027109	0.03173583	0.02443361
0.04627174	0.04302427	0.0512713	0.00648185	0.05515108	0.01206135	0.13108086
0.02762542	0.05340828	0.01451656	0.02635258	0.04420584	0.00061393	0.00324975
0.04033621	0.03389996	0.03090927	0.0406255	0.02074159	0.01717916	0.00756617
0.00555282	0.01019243	0.00190243	0.01601847	0.00389893	0.00181558	0.005174
0.08674463	0.07140138	0.05125029	0.05690748	0.04851931	0.03977481	0.0224441

0.00378261	0.00416447	0.00500661	0.00438677	0.0659453	0.00383758	0.00675481
0.02081799	0.02218306	0.01938876	0.00786531	0.02037615	0.00428774	0.03261212
0.38288068	0.42398873	0.32930616	0.09618037	0.40641497	0.25001511	0.91492522
0.00481422	0.00476436	0.00453103	0.00285769	0.00596982	0.00411133	0.00900634
3.79054026	2.29025345	0.86073433	0.83707408	9.42033492	0.97126754	7.47617692
5.89145625	4.82360152	4.73519404	3.35791837	5.7586642	6.46883027	5.57933072
0.02520463	0.01353018	0.01709438	0.00905551	0.01784679	0.02924873	0.04681935
2.35767005	2.52203044	2.43540696	0.89087134	2.38710233	0.39624134	2.59677042
7.09672412	8.24839665	8.2198499	4.16482809	5.11114911	3.13114468	14.442296
0.0168009	6.48718999	4.09199478	0.11896658	0.02351193	0.00742738	1.10860414
0.04663546	0.01905881	0.03375913	1.15195532	0.04046994	1.20994664	3.7972921
0.01805807	0.01559299	0.01078993	0.0008111	0.005108	0.00045402	0.00086592
0.00405575	0.08010823	0.07701875	0.01179825	0.00396056	0.00806885	0.17228795
0.00267785	0.00320656	0.00108874	0.00139512	0.35790873	0.00065338	0.00184175
0.03650055	0.02625999	0.03613276	0.01810104	0.03156531	0.06199295	0.06152788
0.02580366	0.05207143	0.00713414	0.05551123	0.02119106	0.00051022	0.00498777
0.01231337	0.01100255	0.01125216	0.00915186	0.01346711	0.01686456	0.02857541
2.87809254	3.5019616	3.17109716	0.68497184	1.59580066	0.54112515	6.75796899
0.28655405	0.2681913	0.19316078	0.18223912	0.30024093	0.3217453	0.261269
0.64656698	0.65539064	0.8714135	0.38012982	1.05040425	0.41473996	1.22061104
0.15559219	0.17680199	0.17371482	0.07188786	0.16962357	0.07937533	0.19668578
0.64045263	0.61145272	0.66139131	0.19097554	0.80721651	0.53996932	1.13579628
0.17618992	0.19076605	0.20411039	0.12908854	0.15901598	0.42677021	0.60460258
0.19224831	0.24763239	0.23716152	0.17968699	0.23111182	0.08980969	0.31844684
1.08230813	1.14320084	1.0666536	0.25650158	1.00840953	0.32920293	2.04191862
0.00891959	0.0114876	0.00518954	0.00301367	0.00466205	0.00731551	0.01344042
69.2044951	89.0845572	68.5658724	20.7570002	63.8033943	25.2157504	121.141561
0.1163923	0.13899657	0.19567748	0.04636486	0.28224997	0.12576053	0.80559867
0.0749742	0.07306472	0.00164458	0.55322222	0.85406518	0.01203048	0.1310558
31.8347894	38.1447503	30.5844878	10.7527948	31.543592	7.22740791	59.9835831
0.1950109	0.18792259	0.22749099	0.23422584	0.23068382	0.37765818	0.37024114
0.93335781	1.17899801	1.01899066	0.34934522	1.15948474	0.09657618	0.95671759
45.8077731	51.0075413	1.31127325	14.2916411	74.6346735	0.36935224	94.5316945
0.28235442	0.12573873	0.12632465	0.09270613	0.1762894	0.14164594	0.21954594
0.03791513	0.0392768	0.04291074	0.00291342	0.02431913	0.00099006	0.0441323
1.90270857	2.11596254	1.83578412	0.37206212	2.64799684	1.32202952	10.5364077
0.00592206	0.11434419	0.00450082	0.00275441	0.00477044	0.00238723	0.00629002
0.03070568	0.03354576	0.03262042	0.01557124	0.03383074	0.01826983	0.05270238
12.8030254	15.9764601	14.9672514	4.28667556	12.9500456	3.0757022	20.9511801
1.47437526	1.7275324	1.51349674	0.28891467	1.567267	0.65319402	4.18007395
19.0877658	22.0742359	21.851107	8.49138034	21.8429948	7.21228504	27.3274289
17.304112	17.30066	14.451596	5.04144817	18.2315979	10.6346497	43.1603649
0.18715229	0.22102487	0.19347542	0.09477196	0.22884937	0.09305314	0.31158824

18.1378197	20.9004972	22.6535976	11.3580426	25.8324251	11.6420951	30.0706351
0.00965926	0.01174437	0.01149817	0.00303759	0.01190197	0.00270849	0.01344078
1.213837	1.35981312	1.14745179	0.3072594	0.93695999	0.28927908	1.86092827
42.3687996	46.1227196	57.52968	15.3562103	56.1163347	15.7606394	74.9192076
0.17397702	0.2126561	0.16378075	0.07421175	0.17593393	0.10277691	0.16935753
0.04411732	0.04144529	0.0446954	0.02366001	0.04159555	0.01255474	0.03722114
0.04607548	0.02917725	0.03247978	0.00917053	0.04195518	0.0172488	0.09944975
0.27529071	0.38282172	0.43853133	0.05973289	0.49043458	0.28076535	2.05250533
0.03154611	0.02963625	0.02872296	0.01094785	0.02460444	0.00900234	0.04948598
0.08336886	0.11154179	0.03895155	0.22569458	0.14585026	0.01825528	0.04396296
1.96227224	1.8438937	1.5947541	1.33883715	1.22089196	1.8547222	2.13361392
0.04259096	0.05984659	0.02160398	0.02456458	0.02166651	0.01248051	0.0277224
0.05728007	0.05432754	0.09289242	0.06606936	0.17215126	0.07141051	0.14140935
0.00826199	0.00525025	0.00139354	0.01736877	0.00113721	0.00093071	0.00189489
0.05751467	0.0562795	0.05424164	0.04341807	0.07069572	0.05362538	0.11204047
0.0553082	0.0440935	0.04736337	0.02312487	0.07584414	0.04019487	0.11104276
0.00707043	0.11206207	0.00306199	0.00134548	0.00424338	0.00221295	0.01543255
0.02133489	0.02355618	0.0209053	0.01176454	0.02057791	0.01007399	0.03789983
0.20733912	0.17064946	0.22473772	0.04539228	0.21425912	0.09093922	0.39259317
34.0933615	33.8073682	29.3968289	7.08384782	26.6650506	7.18459035	46.3736908
0.26855867	0.25600233	0.28941757	0.10183384	0.28244203	0.16842328	0.87731092
0.01952188	0.02881303	0.02929316	0.0178244	0.03172439	0.02557316	0.02900974
1.14595938	0.99712489	0.85250165	0.86337618	1.10125466	1.96532912	2.33140634
0.0574895	0.05926643	0.09163746	0.05082755	0.06658668	0.03525534	0.07682773
0.14355888	0.23036694	0.25952586	0.19033753	0.14354701	0.23257545	0.34452218
0.05187295	0.0504472	0.05406118	0.0178651	0.05209389	0.02698686	0.10535732
0.96365668	0.99633665	1.11593652	0.59877855	1.3214473	1.27093857	2.2887749
1.73757523	1.57490429	1.93545283	0.65652104	1.52618253	0.60328128	2.24218646
5.70526945	0.91132198	1.39543235	0.01714492	0.18293568	1.56304855	0.1625572
0.21553287	0.31117768	0.26748988	0.37734079	0.2381249	0.67052709	0.30215486
0.016612	0.01601357	0.01235912	0.00576618	0.01474891	0.00387495	0.02442387
0.00028658	0.00044391	0.0003477	0.00482308	0.04629685	0.05518194	0.08514474
0.01772787	0.02073586	0.01951082	0.00682775	0.0182567	0.00921798	0.02195251
0.09477826	0.00106078	0.1528202	0.04961873	0.08279358	0.07925613	0.23676484
0.03254577	0.0201313	0.01218576	0.00696023	0.0114063	0.01630049	0.01515519
0.05772627	0.01962483	0.01626576	0.04304393	0.02102053	0.01552528	0.11947528
0.33577494	0.34127873	0.51694065	0.13321966	0.44644773	0.19424103	0.87754136
0.04923736	0.03827318	0.01787635	0.00100854	0.03926418	0.00089703	0.00575367
0.02140919	0.01740615	0.02243245	0.00570122	0.00372703	0.0089668	0.01424088
0.00310358	0.00423763	0.00026603	0.00017328	0.00027014	0.00033973	0.00064455
0.07334927	0.08430896	0.0759526	0.07538044	0.09757619	0.04428221	0.07391103
2.15087598	2.50950666	2.26826379	1.98175825	2.93913892	2.09594514	2.30994159
0.0664119	0.06926197	0.06917694	0.09940487	0.07389388	0.04890357	0.04466175

1.49601026	2.4424875	0.43795477	0.96390949	0.71771911	0.10707409	0.27207078
0.01365607	0.01419993	0.01301143	0.00663954	0.01015579	0.00601091	0.0205296
0.02511193	0.03247041	0.01287575	0.05358838	0.05414992	0.00844313	0.01693572
0.06529969	0.07400471	0.06410219	0.0544783	0.0552312	0.03575955	0.06408844
0.70788466	0.81195435	0.68465218	0.14078275	0.83970423	0.2722306	1.72931926
0.02989683	0.02868174	0.03323294	0.01259434	0.02637136	0.00438691	0.02648221
0.59138809	0.680317	0.62982042	0.14777169	0.43117986	0.10810472	1.0379101
0.04383785	0.04758042	0.03814916	0.02909286	0.02645158	0.01887981	0.03287324
0.02686794	0.03525638	0.03263534	0.01954074	0.02219682	0.0036077	0.02111822
0.38986527	0.37829847	0.36914004	0.46743544	0.73518189	0.42620603	0.39632457
12.2537647	14.3568142	11.0981006	3.00897744	11.5246632	4.35248814	21.7449388
9.30567099	11.0057724	10.2027035	3.57558396	9.46938116	3.2009448	18.3630529
6.14335841	7.46536526	8.35158549	1.53301435	12.3039496	1.64463736	19.8280867
0.31831424	0.34235265	0.33448759	0.18693095	0.38302708	0.16211556	0.51857972
0.42056987	0.55494579	0.42855127	0.16128894	0.29622757	0.15112722	0.48860305
0.05386674	0.05671056	0.04546616	0.03831497	0.06989772	0.10126816	0.07576514
1.41334546	1.63744946	1.42302631	0.91149249	2.15663508	1.58656633	3.53959319
0.56669693	0.60297461	0.43973419	0.4886029	0.38389316	0.26852575	0.41982066

TNFaButyrate	TNFaButyrate	TNFaButyrate	TNFaButyrate	TNFaButyrate_Combined
C4	C5	C6	C7	C8
Combined 50	Combined 50	Combined 50	Combined 50	Combined 500µM Butyrate + 500pg/mL
44032	44032	44032	44032	44032
Sp28	Sp29	Sp30	Sp31	Sp32
0.06900911	0.71703582	0.01217769	0.18722349	0.10429956
0.01611985	0.02947896	0.03840981	0.07399534	0.0364262
0.04263857	0.04521352	0.06205888	0.11578718	0.02322315
0.02843064	0.05666544	0.15691097	0.12394213	0.00028981
0.04956278	0.09474569	0.15247017	0.24750986	0.81922554
0.01419031	0.01637991	0.03620894	0.04486841	0.1365247
0.00827532	0.01346153	0.03088047	0.02035488	0.07568209
0.00686405	0.01260926	0.01008684	0.02163144	0.00715661
0.10527618	0.16730756	0.21426815	0.40076736	6.34502882
0.08198052	0.04455674	0.0428058	0.08858702	0.25347193
0.15882168	0.25899716	0.31789101	0.57980211	0.49533141
0.00430626	0.0011516	0.02261662	0.02646363	0.02139186
0.06331577	0.05888135	0.06256535	0.13409523	0.01331182
0.27540567	1.52835518	2.60450595	2.73236515	0.01296138
0.16912109	0.24477565	0.27502827	0.39454358	0.07788557
0.04037304	0.11354014	0.21392007	0.3209382	0.03463304
0.01485197	0.0226243	0.03264488	0.03925268	7.97022551
0.0174564	0.03217692	0.05385356	0.057863	0.52847076
0.01145892	0.02032572	0.02453556	0.03506197	3.38606419
0.11371327	0.12720116	0.21516673	0.33813621	0.00024563
0.01210375	0.01891853	0.02553363	0.04992693	0.02550321
0.06578756	0.14338087	0.24157206	0.34914217	0.01860072
0.00343346	0.00692478	0.00806904	0.01649687	0.30762674
0.0198413	0.02210735	0.03434413	0.04689136	1.07343558
0.00626545	0.01323908	0.02396842	0.04054349	0.0073039
0.04155838	0.05627095	0.06352345	0.09437115	9.70826078
0.0502743	0.06161668	0.08320198	0.11333197	0.07338567
0.0319542	0.03960907	0.03989063	0.05768739	0.00079807
0.00324068	0.00567563	0.01076511	0.01483718	0.01666332
0.02771621	0.04145737	0.04978092	0.06563234	0.14582357
0.00954126	0.01110577	0.02121634	0.02488057	0.05034169
0.00576802	0.00723776	0.02783676	0.03467283	0.01263025
1.00999793	1.64347493	1.64785499	4.00498986	0.00950836
1.00255176	1.42530099	1.23376275	1.21762805	0.01095143
0.00400676	0.01241579	0.02526272	0.02999675	0.11230734
0.18656769	0.45292559	0.81022235	1.3048927	61.4343986
0.13108413	0.13656658	0.14259445	0.17901111	0.20041332
0.00601727	0.01005218	0.0174155	0.02292504	10.9793091

0.19161052	0.36096508	0.44288361	0.52139369	0.04026592
62.3368575	113.686031	138.569682	195.073494	0.84126264
9.5305E-05	0.00014717	0.01082122	0.00055035	0.00081282
0.13198102	0.25019169	0.41290364	0.65161742	0.00250524
0.00841628	0.01719372	0.02499994	0.04062651	0.00880368
0.04500894	0.01121327	0.02905521	0.02439408	0.02366632
0.00030424	0.00248505	0.00763479	0.00537805	0.18500781
0.04081246	0.04136819	0.06286547	0.11129059	0.09650431
0.01513467	0.03198435	0.03121395	0.05717944	0.1152261
0.04676589	0.12607188	0.1394833	0.13806075	0.03827583
0.00511843	0.00809525	0.00785654	0.00409796	5.17567112
0.22928976	0.24176248	0.23987451	0.25322128	10.2501759
0.2208217	0.28334791	0.6141643	0.84078218	0.9376105
0.15956526	0.25642118	0.32634608	0.45814421	0.23195021
0.34608791	0.50773672	0.53760563	0.81746127	0.00518523
4.0799228	5.83692545	5.66280117	8.10782421	2.31823865
0.03729605	0.08423704	0.17073256	0.19835048	0.11846157
0.00104266	0.00087921	0.00139107	0.00166503	0.00554104
0.00489466	0.00677457	0.01326533	0.02621252	0.00529218
0.11953371	0.24607113	0.34306258	0.46723361	11.9912047
0.01852915	0.14652701	0.4127791	0.65590546	0.05699693
0.10266505	0.17110968	0.22670611	0.38108718	0.01512214
0.0270805	0.04123802	0.05931291	0.04195261	0.02602672
1.13800774	1.36112362	1.64533535	2.51583737	0.09632244
0.21266775	0.38956473	0.67099208	1.23961902	0.03207443
3.14133979	4.32641829	2.7090014	3.67624196	15.6531748
16.2805711	27.9279182	39.924103	57.3560474	0.05725306
2.06375794	3.28148327	3.95894868	3.95557512	0.00037253
0.01593259	0.03001916	0.06563117	0.06482471	0.01642149
0.80567598	3.00219838	8.27601807	5.26650284	0.12942897
0.53682779	0.6424502	0.71921743	1.0634976	0.04220575
0.49782431	1.23168978	2.08519547	2.47385197	0.10823434
0.93344753	2.15001553	2.99784547	5.25261127	11.6666197
0.0948223	0.11269663	0.09259856	0.12606527	2.40731653
2.51757762	5.46471019	7.86806355	12.8045068	0.02256366
0.16684445	0.25376498	0.40443972	0.66604869	0.09908317
15.4460858	23.0404064	23.4984804	38.2790302	0.25125269
0.00160599	0.01317768	0.02881861	0.07810252	0.01051785
0.01857799	0.02932748	0.03350786	0.09268051	0.02924845
0.00146076	0.00656376	0.01657068	0.00519869	0.14753932
0.03225654	0.028885	0.03998218	0.01799186	0.01391057
0.00055622	0.00080358	0.00191446	0.00339982	0.01117062
0.03500869	0.06217079	0.07747668	0.00352667	0.02592

0.00104444	0.00202628	0.00559562	0.00589911	0.04320863
0.00739943	0.00751434	0.00578701	0.01632368	0.01006368
0.10606575	0.23545437	0.3625018	0.53828009	2.35851037
0.00369977	0.00470973	0.00556935	0.00794249	0.03681295
1.43456301	0.67779532	0.86603386	4.16231171	0.05167469
8.97883246	9.01562761	8.99997534	11.4233499	0.13207617
0.00810586	0.0089162	0.02021077	0.03209139	0.00323173
1.46801184	1.69179153	1.7248563	2.81294667	0.14862383
4.58928182	6.04341765	8.07011506	9.1135689	0.02602168
0.18455243	5.82583225	2.21400499	1.93130401	5.95262678
0.01080246	0.03581701	2.17455999	3.34758567	0.01222472
0.00831736	0.00814553	0.00615776	0.0154668	0.00143285
0.01201375	0.17056513	0.14045745	0.13432175	0.10323565
0.0003979	0.00067776	0.00125826	0.00213236	0.15043422
0.01279153	0.01946779	0.03064634	0.03000283	31.3171587
0.00204955	0.00867794	0.014395	0.00563371	0.01555907
0.00536746	0.01095206	0.01248538	0.02133045	0.00055432
0.4421071	0.23504762	4.83785458	6.83898786	0.0062166
0.47879202	0.46285898	0.46606581	0.58426911	0.25538945
0.41039261	0.51599493	0.59154414	0.99863674	0.35241285
0.05911009	0.09594048	0.12522492	0.18790528	0.15917686
0.14222493	0.25579689	0.53858863	0.78816609	0.04524036
0.04568693	0.11382569	0.26134564	0.25697917	0.98882274
0.16278442	0.00168451	0.15953961	0.26548737	0.01048072
0.32210667	0.59871285	0.71585478	1.37301356	0.05117918
0.00360973	0.00644092	0.01515429	0.00849872	0.06749496
20.0550927	35.7754994	48.9143745	83.7057005	13.6406685
0.03497783	0.12356878	0.2592214	0.40324706	202.187951
0.38195221	0.51325847	0.70781557	0.11692805	0.05114376
16.5263224	22.4642591	20.0506278	47.330816	0.00661814
0.17644231	0.2445954	0.25571113	0.26278144	0.08558944
0.91702662	0.97994493	0.87940968	1.59473354	0.44968419
0.46024744	35.0134207	55.0251957	70.3159887	0.71107864
0.17596613	0.12511881	0.25159787	0.17897803	0.02140161
0.00702692	0.01699859	0.01698193	0.03388668	0.00311369
0.28552967	0.5956795	1.63855909	3.94189178	0.05803563
0.0048951	0.00472658	0.00438962	0.33053007	0.03653032
0.0187676	0.02726875	0.02729412	0.0528384	0.18501631
7.20529485	11.5252697	9.11048596	20.2631246	0.01118098
0.15642921	0.46707542	1.14946822	2.50134267	0.0039026
8.97322992	13.4710967	16.424327	24.5704547	0.01580253
8.40096753	11.0650229	14.5858231	24.9939605	0.81139359
0.12541325	0.20809592	0.17516561	0.34258541	0.04421843

11.65967	16.6905356	15.3223488	28.5624691	0.12876133
0.00686041	0.00865492	0.0074854	0.01561498	0.01628838
0.31416323	0.69162087	0.94239542	1.69746926	0.02051575
20.7876699	29.6458684	34.3418918	59.5652283	0.53145674
0.09419233	0.13505678	0.12546275	0.20179938	0.29003187
0.02395895	0.03366779	0.02395354	0.04622668	0.0412673
0.00978043	0.02483254	0.04963155	0.0860985	0.00565466
0.01932073	0.07644793	0.30855565	0.52416225	0.7094366
0.01487152	0.02290223	0.0258342	0.05729965	0.11853648
0.01246043	0.03186686	0.08160451	0.02996159	0.03854553
0.95975804	1.06848878	1.46495219	2.16603905	0.00341198
0.00624782	0.01058075	0.02551867	0.02225824	1.24549752
0.03574895	0.05821977	0.07563367	0.10158153	0.0068192
0.03228738	0.02089613	0.0013242	0.00240565	0.03694074
0.03830643	0.04010858	0.06343663	0.10291485	0.02028535
0.02943036	0.03722979	0.05369346	0.10214787	0.11068378
0.00503203	0.00354954	0.00539107	0.06416131	0.00051446
0.01232873	0.01830127	0.01876261	0.03366453	0.00764238
0.05308236	0.1061476	0.18418971	0.35402562	3.70494519
7.95280819	12.7780699	18.7074356	31.7160341	0.92399626
0.06271317	0.14567509	0.22604418	0.40753578	0.00897503
0.02828475	0.02839167	0.01501926	0.02814584	0.04163019
0.70164517	0.90729816	1.15981255	1.54399685	3.49322114
0.0725513	0.05784633	0.04128907	0.0712439	0.00668056
0.07019404	0.12152943	0.25111904	0.19682642	0.27748114
0.01410932	0.02266475	0.04547142	0.0611327	0.00373641
0.42940719	0.59735213	0.97705423	1.40708037	1.40386609
0.66962479	1.1197335	1.53898293	1.36672791	1.43324354
0.04517373	0.01516526	0.77210512	1.59743836	0.29310304
0.26427165	0.36162542	0.28612162	0.41537628	0.0460925
0.00818606	0.00972218	0.01496373	0.0194402	0.00959707
0.01422444	0.04642127	0.00688285	0.00448089	0.93145474
0.0053778	0.00623868	0.00862767	0.01600812	5.0059261
0.06061416	0.00089564	0.09865798	0.00132028	0.00914341
0.0037451	0.00508883	0.00859342	0.00345805	3.82461291
0.04477269	0.07587886	0.0755083	0.07165128	0.00449797
0.24111193	0.42283924	0.44448861	0.71079016	0.05819087
0.00470829	0.05313372	0.01630689	0.04349399	0.02471532
0.0035618	0.00503221	0.00880172	0.01147437	0.03125938
0.00171931	0.00045579	0.00047957	0.03116101	0.03429681
0.12703898	0.09986597	0.06983032	0.09214831	0.02001308
1.92004527	4.41008018	2.39840749	2.89384494	1.08120632
0.27719085	0.18017376	0.0549944	0.05934165	0.40735661

0.07638395	0.24680879	0.4268371	0.24300804	0.00339459
0.00703134	0.00964288	0.01273129	0.01802232	0.7036546
0.00506525	0.01241374	0.03058934	0.01112942	0.00304911
0.06210392	0.0003254	0.04084076	0.05199349	4.98367485
0.23043092	0.44500396	0.63420331	1.05621953	0.00592128
0.01737181	0.01511231	0.00814003	0.01971624	0.26933799
0.18294376	0.28694564	0.49578282	0.58487928	0.04333161
0.00085813	0.03126328	0.01839456	0.03391319	0.0030178
0.03128959	0.02582236	0.00892061	0.01899858	0.24593127
0.82411252	0.84364847	0.472014	0.39319115	0.54367064
3.65810284	7.37821918	12.4706273	18.7813262	0.00508804
4.51789586	7.04888038	7.72423248	13.7624577	0.03150482
2.22631889	5.15753594	4.47592929	7.33799423	0.2891975
0.21146187	0.23147458	0.29906976	0.48590419	0.08284005
0.11624002	0.25238399	0.39683528	0.43890656	0.11926399
0.04835664	0.02920734	0.03303259	0.04623933	0.06477369
1.2226168	1.48287913	1.7023364	2.44894889	0.01246282
0.4735852	0.55969908	0.51036684	0.42780757	0.04315302

TNFα #8

Name	SRM1950_	SRM1950_	SRM1950_	Ave	SD	%CV	
(+/-)13-HO	0.010	0.007	0.021	0.01	0.01	57.42	**Median CV**
(1R,9S)-11-	0.001	0.001	0.001	0.00	0.00	8.93	
(4-Fluorop	0.000	0.000	0.001	0.00	0.00	38.86	
[(3S)-3-(1H	0.001	0.001	0.001	0.00	0.00	12.27	
[1-(4-fluoro	0.000	0.000	0.000	0.00	0.00	8.19	
1-(4-chloro	0.002	0.002	0.002	0.00	0.00	7.02	
1-(4-Fluoro	0.000	0.000	0.000	0.00	0.00	17.54	
1-allyl-4,5-	0.000	0.000	0.000	0.00	0.00	9.92	
1,5-diphen	0.001	0.001	0.001	0.00	0.00	9.16	
2-(2-amino	0.029	0.030	0.026	0.03	0.00	5.98	
2-(Acetylar	0.176	0.125	0.169	0.16	0.03	17.64	
2-[(3S)-1-(2	0.000	0.000	0.000	0.00	0.00	8.61	
2-[(3S)-1-(3	0.000	0.000	0.000	0.00	0.00	12.52	
2-Amino-N	0.182	0.080	0.182	0.15	0.06	39.97	
2-Furoic ac	0.447	0.413	0.428	0.43	0.02	4.01	
2-Naphtha	1.971	1.870	1.936	1.93	0.05	2.65	
2,3,4,9-Tet	0.000	0.000	0.000	0.00	0.00	11.42	
2,4-Dimeth	0.002	0.002	0.002	0.00	0.00	14.33	
2,5-Dimeth	0.001	0.001	0.001	0.00	0.00	15.23	
3-(2-fluoro	0.019	0.019	0.019	0.02	0.00	1.43	
3-(allylsulfa	0.000	0.000	0.000	0.00	0.00	15.60	
3-(benzylth	0.001	0.000	0.000	0.00	0.00	4.52	
3-[4-(tert-b	0.001	0.001	0.001	0.00	0.00	12.35	
3-amino-2-	0.002	0.002	0.002	0.00	0.00	12.35	
3-Hydroxy-	0.034	0.034	0.026	0.03	0.00	13.95	
3-Hydroxy-	0.080	0.076	0.077	0.08	0.00	2.52	
3-Phenyl-2	0.001	0.001	0.001	0.00	0.00	17.10	
3,5-Dimeth	0.023	0.022	0.023	0.02	0.00	3.46	
4-(2-metho	0.001	0.001	0.001	0.00	0.00	11.48	
4-(4-metho	0.002	0.001	0.001	0.00	0.00	26.81	
4-[2-(2-Thi	0.000	0.000	0.000	0.00	0.00	9.98	
4-{[(3S)-3-(	0.000	0.000	0.000	0.00	0.00	4.10	
4-Hydroxyl	1.416	1.682	1.645	1.58	0.14	9.11	
4-Methoxy	0.004	0.003	0.016	0.01	0.01	89.01	
4-methyl-6	0.009	0.024	0.013	0.02	0.01	52.18	
4-Methylp	0.469	0.384	0.422	0.43	0.04	10.09	
4-Morphol	0.001	0.001	0.001	0.00	0.00	15.03	
4-morphol	0.005	0.005	0.005	0.00	0.00	5.35	
4-Nitrophe	0.291	0.219	0.228	0.25	0.04	15.84	
4-Oxoproli	31.083	25.994	31.925	29.67	3.21	10.82	
4,4'-Bis(die	0.000	0.000	0.000	0.00	0.00	6.96	
5-(2-Chloro	0.001	0.001	0.001	0.00	0.00	9.49	

5-Fluoro Tl	0.004	0.002	0.002	0.00	0.00	39.24
5-Hydroxyl	0.022	0.003	0.009	0.01	0.01	86.53
5(Z),8(Z),1:	0.001	0.001	0.001	0.00	0.00	18.50
6-Methyl-2	0.009	0.008	0.006	0.01	0.00	17.43
7-Methylg	0.059	0.069	0.066	0.06	0.01	8.27
8-iso Prost	0.009	0.011	0.003	0.01	0.00	50.59
8,8-dimeth	0.003	0.001	0.002	0.00	0.00	39.59
9-Phenant	0.012	0.011	0.011	0.01	0.00	6.21
Acetyl-L-ca	8.778	7.707	7.714	8.07	0.62	7.64
Acetylchol	1.266	1.086	1.125	1.16	0.09	8.16
Adenine	0.003	0.003	0.003	0.00	0.00	2.41
Adenosine	0.003	0.002	0.002	0.00	0.00	25.03
Adipic acid	0.213	0.185	0.152	0.18	0.03	16.70
Aniline	0.001	0.001	0.001	0.00	0.00	3.20
Benperido	0.000	0.000	0.000	0.00	0.00	13.80
Benzoic ac	0.527	0.388	0.345	0.42	0.09	22.60
Betaine	11.908	11.791	12.564	12.09	0.42	3.45
Biopterin	0.000986	0.000874	0.000818	0.00	0.00	9.59
Bis(4-ethyl	0.001	0.001	0.001	0.00	0.00	17.53
Chlormequ	2.014	1.489	2.122	1.87	0.34	18.04
Choline	0.246	0.233	0.231	0.24	0.01	3.40
Citric acid	4.622	4.237	5.135	4.66	0.45	9.66
Creatine	22.432	19.692	20.470	20.86	1.41	6.77
Creatinine	14.527	12.826	13.499	13.62	0.86	6.29
Cyprohept	0.001	0.000	0.000	0.00	0.00	72.28
Cystathion	0.009	0.008	0.008	0.01	0.00	6.23
Cysteinylgl	0.002	0.001	0.002	0.00	0.00	6.05
Cytarabine	0.098	0.090	0.086	0.09	0.01	6.85
Cytidine	0.004	0.002	0.003	0.00	0.00	32.88
Cytidine 5'	0.001	0.001	0.001	0.00	0.00	7.85
Cytosine	0.052	0.043	0.049	0.05	0.00	9.08
D-(+)-Malic	0.056	0.036	0.039	0.04	0.01	24.49
D-(+)-Proli	11.105	10.975	11.448	11.18	0.24	2.18
D-Panthen	0.012	0.003	0.007	0.01	0.00	61.72
D-Serine	0.016	0.014	0.013	0.01	0.00	11.56
delta8-THC	0.002	0.002	0.002	0.00	0.00	17.01
Desmethyl	0.000	0.000	0.000	0.00	0.00	6.40
Di(2-ethylh	0.029	0.006	0.014	0.02	0.01	70.96
Didemethy	0.000	0.000	0.000	0.00	0.00	6.79
Diphenylar	0.001	0.002	0.001	0.00	0.00	26.98
Disperse b	0.000	0.000	0.001	0.00	0.00	34.68
DL-Alanine	0.358	0.271	0.292	0.31	0.05	14.71
DL-Arginin	0.009	0.007	0.007	0.01	0.00	11.10

DL-Lactic A	3.228	11.386	16.398	10.34	6.65	64.30
DL-Malic a	1.214	1.055	1.218	1.16	0.09	8.02
DL-Serine	0.000	0.000	0.000	0.00	0.00	9.12
DL-Tryptop	0.006	0.044	0.037	0.03	0.02	69.64
DL-β-Leuci	5.568	5.424	5.553	5.51	0.08	1.43
Docosatrie	0.006	0.007	0.010	0.01	0.00	31.02
Dodecyl su	0.972	0.070	0.051	0.36	0.53	144.39
Ethyl 2-(4-	0.000	0.000	0.000	0.00	0.00	20.25
Ethyl myris	0.174	0.178	0.138	0.16	0.02	13.36
Ethyl viole	0.002	0.001	0.001	0.00	0.00	28.67
Ethylmalor	0.114	0.080	0.099	0.10	0.02	17.39
Fingolimoc	0.001	0.001	0.001	0.00	0.00	29.09
Flurandrer	0.014	0.011	0.011	0.01	0.00	11.40
Formonon	0.020	0.023	0.012	0.02	0.01	30.69
Fumaric ac	0.071	0.057	0.066	0.06	0.01	10.54
Gluconic a	1.895	1.360	1.501	1.59	0.28	17.47
Glutaric ac	0.228	0.216	0.188	0.21	0.02	9.86
Glycine	0.239	0.174	0.173	0.20	0.04	19.20
Glycolic ac	0.610	0.391	0.331	0.44	0.15	33.05
Glycyl-L-le	0.010	0.001	0.008	0.01	0.00	76.19
Guanine	0.002	0.002	0.003	0.00	0.00	32.88
Hexanoylc	0.158	0.122	0.158	0.15	0.02	14.18
Hypoxanth	2.287	2.124	2.114	2.17	0.10	4.48
Indole-3-a	0.140	0.093	0.101	0.11	0.03	22.49
Isobutyric	0.293	0.008	0.483	0.26	0.24	91.38
Isoleucine	19.227	17.866	22.460	19.85	2.36	11.89
Isophthalic	0.016	0.035	0.062	0.04	0.02	62.23
L-(-)-Methi	0.002	0.001	0.001	0.00	0.00	15.53
L-(+)-Lactic	2.405	0.222	191.414	64.68	109.76	169.70
L-Alanyl-L-	0.003	0.004	0.005	0.00	0.00	15.85
L-Dopa	0.003	0.003	0.003	0.00	0.00	14.98
L-Glutamic	0.728	0.595	0.675	0.67	0.07	10.06
L-Glutathic	0.000	0.000	0.002	0.00	0.00	123.91
L-Histidine	0.205	0.156	0.187	0.18	0.02	13.51
L-Isoleucin	10.018	10.000	11.780	10.60	1.02	9.65
L-Methion	0.641	0.617	0.675	0.64	0.03	4.55
L-Phenylala	10.450	10.394	10.701	10.52	0.16	1.56
L-Pyroglut	3.326	3.175	0.181	2.23	1.77	79.64
L-Threonin	0.373	0.449	1.261	0.69	0.49	70.84
L-Tyrosine	22.669	17.665	23.979	21.44	3.33	15.54
L-Tyrosine	0.002	0.002	0.002	0.00	0.00	4.30
L(-)-Carniti	16.915	14.445	15.256	15.54	1.26	8.10
Methylmal	3.934	3.791	3.799	3.84	0.08	2.09

Methylsuc	0.031	0.031	0.025	0.03	0.00	11.43
N-[5-(tert-	0.000	0.000	0.000	0.00	0.00	11.32
N-{[(1S,4S,	0.000	0.000	0.000	0.00	0.00	16.87
N-Ethylgly	0.297	0.317	0.295	0.30	0.01	3.94
N,N-Bis(2-	0.000	0.000	0.000	0.00	0.00	5.88
N,N-Diethy	0.065	0.046	0.062	0.06	0.01	17.73
N,N-Diethy	0.332	0.202	0.339	0.29	0.08	26.59
N,N-Dimet	0.004	0.003	0.003	0.00	0.00	13.20
N,N'-Dicyc	0.028	0.020	0.025	0.02	0.00	18.41
N'-{6-[(5-cl	0.001	0.000	0.000	0.00	0.00	29.04
N2-(2,4-dir	0.000	0.000	0.000	0.00	0.00	6.40
N2-(3-Pyri	0.001	0.001	0.001	0.00	0.00	9.32
N2,1-Diph	0.000	0.000	0.000	0.00	0.00	10.18
N6,N6,N6-	0.116	0.117	0.116	0.12	0.00	0.76
N8-Acetyls	0.003	0.002	0.003	0.00	0.00	25.65
Nicotinami	0.073	0.075	0.073	0.07	0.00	1.65
Nicotinami	0.045	0.040	0.041	0.04	0.00	6.51
Nicotinami	0.001	0.001	0.001	0.00	0.00	8.47
Niflumic ac	0.001	0.000	0.000	0.00	0.00	35.30
NP-00134€	0.001	0.001	0.001	0.00	0.00	7.30
NP-00814	0.035	0.030	0.027	0.03	0.00	13.03
NP-01122(	0.066	0.065	0.061	0.06	0.00	4.43
NP-01645	0.093	0.079	0.095	0.09	0.01	9.69
Orotidine	0.022	0.021	0.019	0.02	0.00	8.59
Palmitoyl s	0.881	0.452	0.141	0.49	0.37	75.59
Perfluoro-	0.017	0.007	0.011	0.01	0.00	39.77
Periciazine	0.000	0.001	0.000	0.00	0.00	29.53
Perillartine	0.000	0.000	0.000	0.00	0.00	8.55
Prolylleuci	0.001	0.001	0.001	0.00	0.00	28.62
Propionylc	0.617	0.219	0.611	0.48	0.23	47.31
Pyridoxam	0.002	0.001	0.002	0.00	0.00	41.45
Pyrimethar	0.007	0.001	0.002	0.00	0.00	92.23
Pyruvic aci	0.567	0.510	0.603	0.56	0.05	8.40
Rhodamin	0.001	0.001	0.001	0.00	0.00	23.98
Ricinine	0.000	0.000	0.000	0.00	0.00	28.43
S-Adenosy	0.000	0.000	0.000	0.00	0.00	9.61
S-Adenosy	0.001	0.001	0.001	0.00	0.00	7.30
Spermidin	0.004	0.004	0.008	0.01	0.00	44.97
Spermine	0.002	0.002	0.002	0.00	0.00	10.91
Sphingosin	0.040	0.104	0.184	0.11	0.07	65.87
Spiperone	0.000	0.000	0.000	0.00	0.00	8.20
Stearamid	0.063	0.048	0.065	0.06	0.01	15.89
tert-Butyl	0.032	0.035	0.029	0.03	0.00	10.23

Thiamine	0.011	0.012	0.012	0.01	0.00	7.84
Thiosildena	0.001	0.000	0.001	0.00	0.00	34.78
Thymine	0.004	0.004	0.004	0.00	0.00	3.92
Tolycaine	0.016	0.001	0.013	0.01	0.01	75.76
Tyrosylalar	0.000	0.000	0.000	0.00	0.00	18.65
UDP-N-ace	0.003	0.002	0.002	0.00	0.00	16.81
Uracil	0.426	0.259	0.395	0.36	0.09	24.62
Valine	14.785	14.221	14.453	14.49	0.28	1.96
Xanthine	2.262	1.887	1.947	2.03	0.20	9.91
α-Aspartyl	0.002	0.002	0.001	0.00	0.00	23.56
β-Alanine	0.003	0.002	0.002	0.00	0.00	25.38
β-Nicotina	0.001	0.001	0.001	0.00	0.00	10.82
Y-Glutamy	0.008	0.005	0.005	0.01	0.00	30.08
Y-L-Glutam	0.002	0.002	0.002	0.00	0.00	28.19

13.20

With outlier

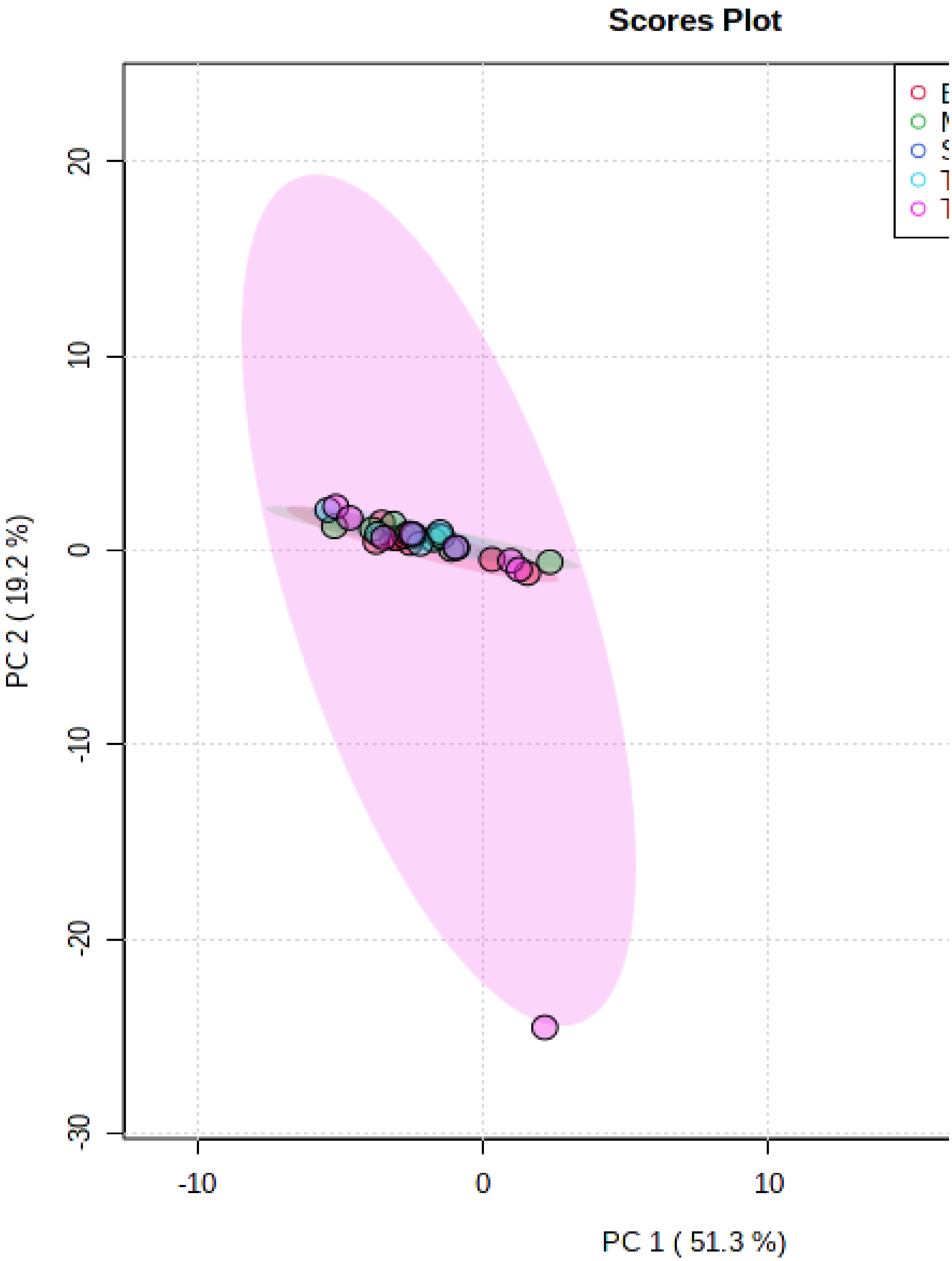

With outlier without SRM1950

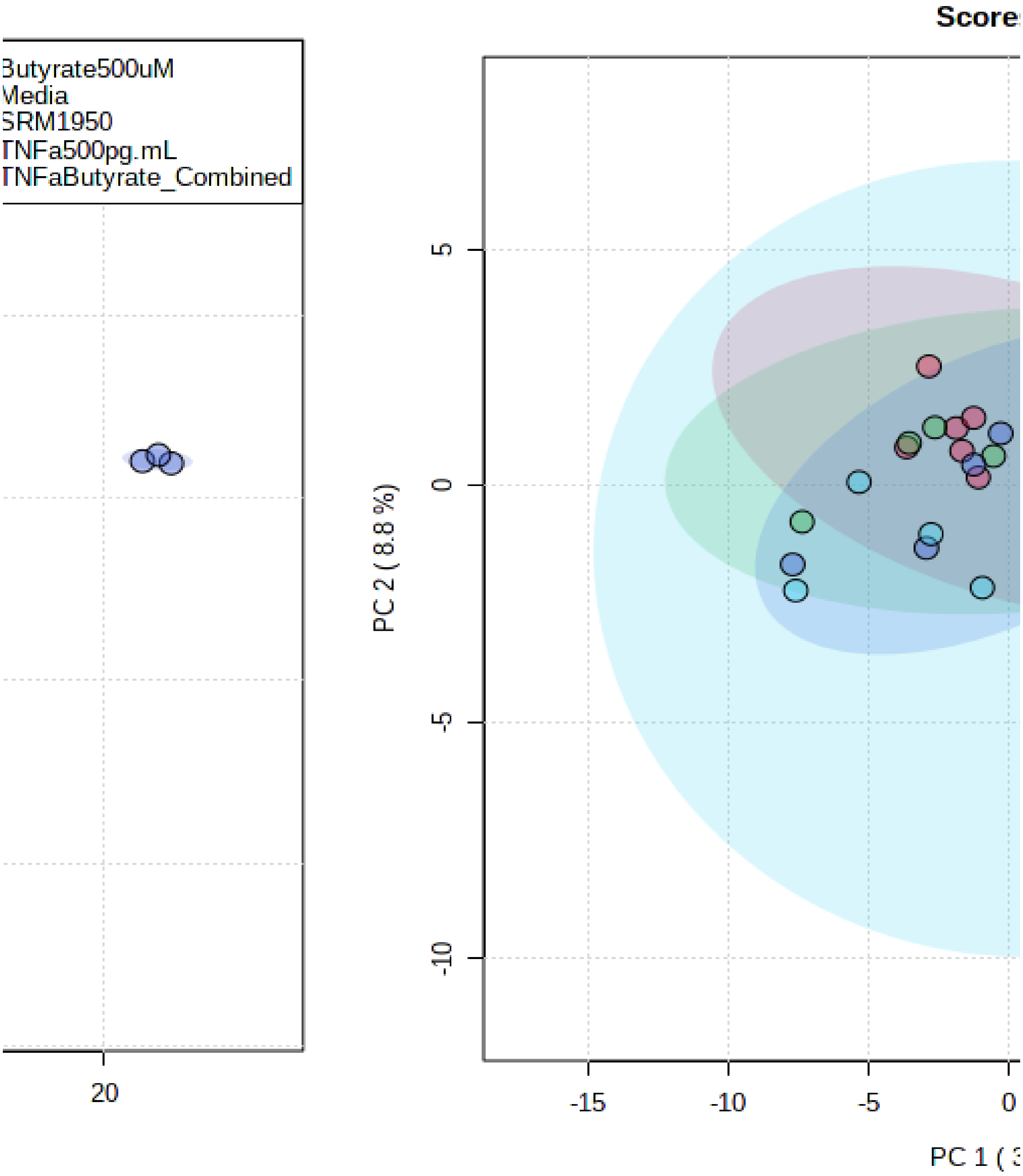

Without outlier without SR

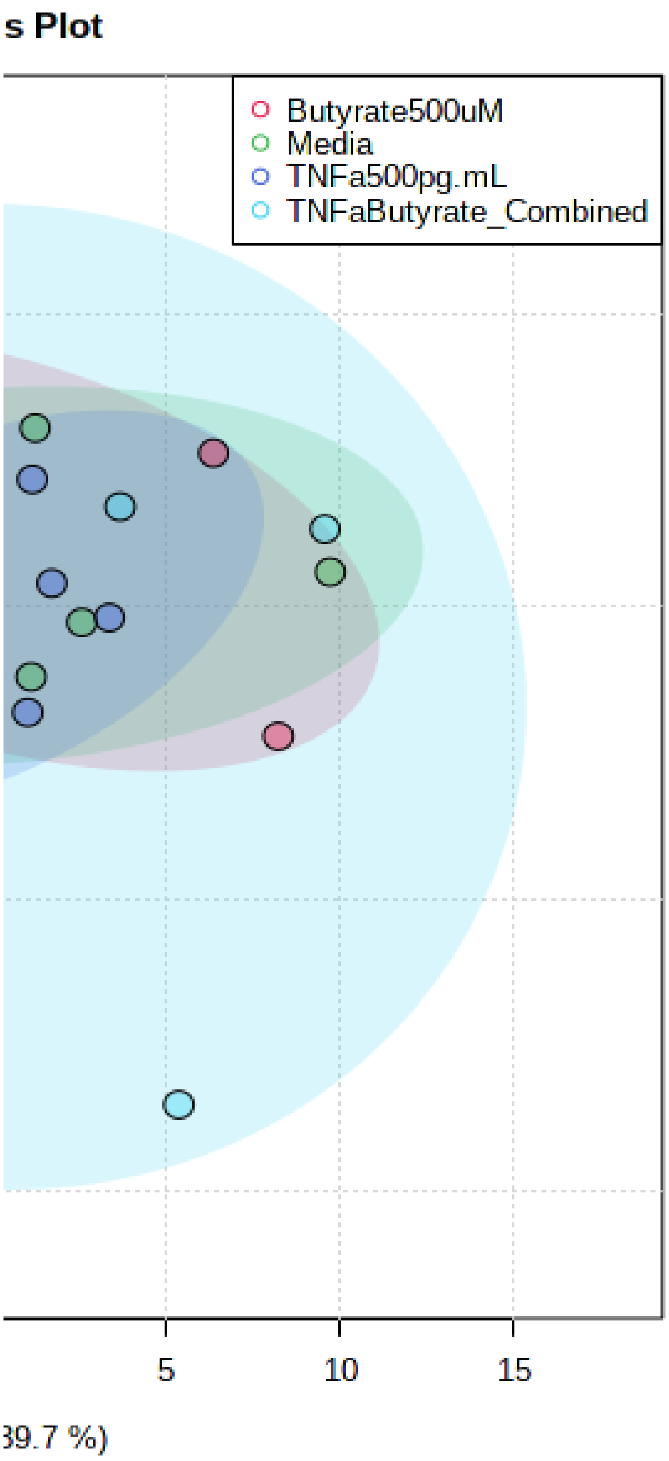

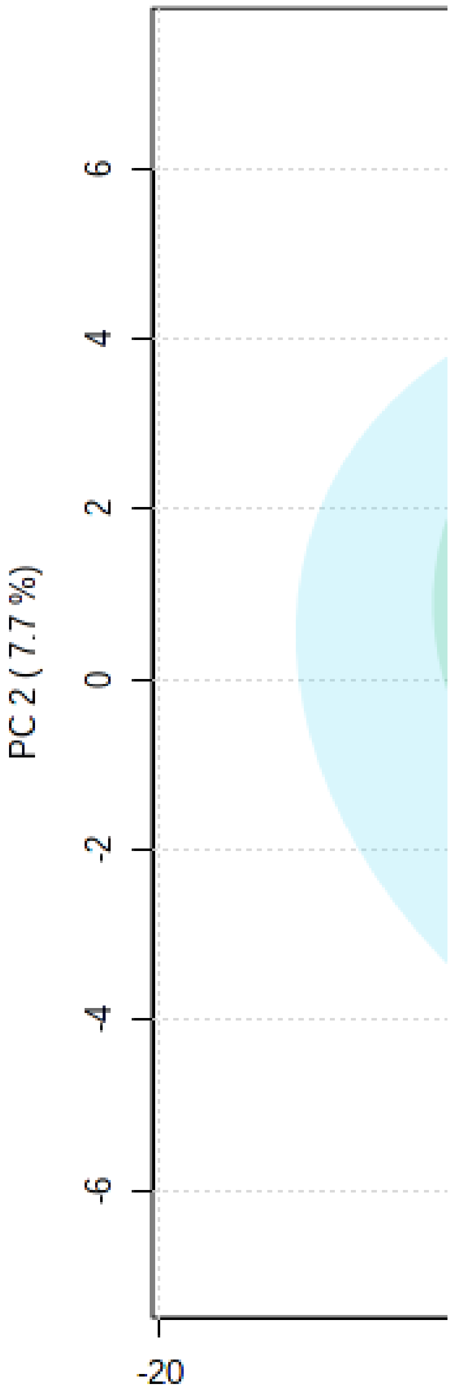

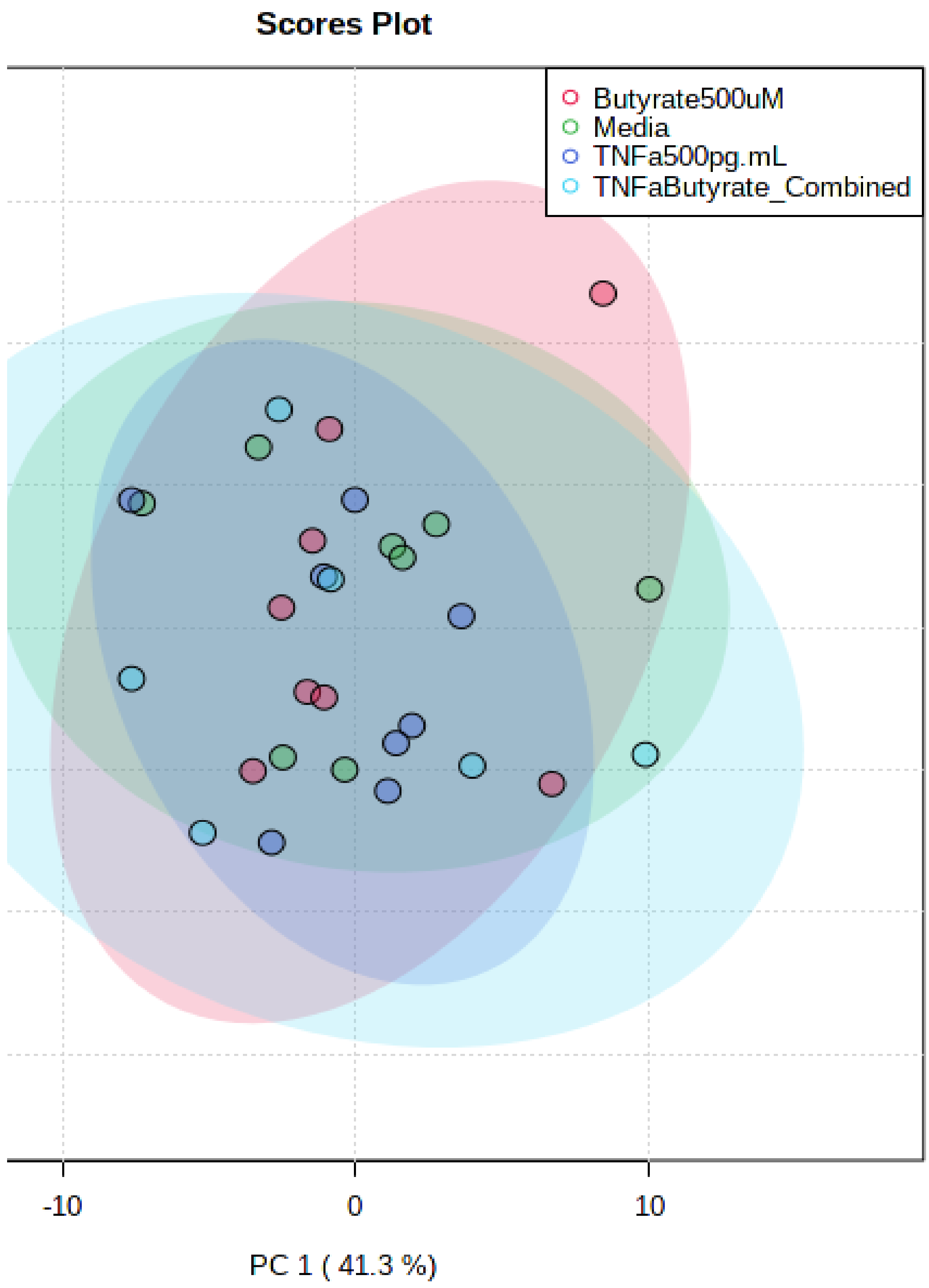

Scores Plot
Butyrate500uM
Media
TNFa500pg.mL
TNFaButyrate_Combined
-10
0
10
PC 1 (41.3 %)

	f.value	p.value	-LOG10(p)	FDR	Fisher's LSD
Biopterin	10.488	0.00010608	3.9744	0.019624	Butyrate500uM - Media; TN

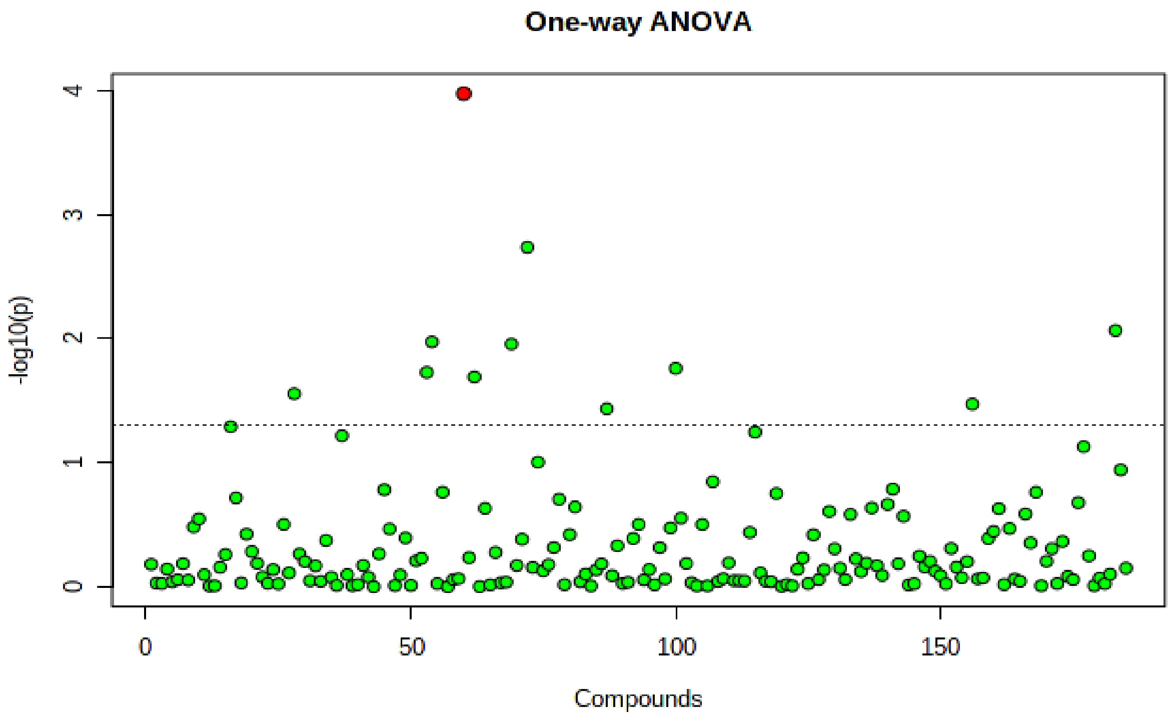

NFaButyrate_Combined - Butyrate500uM; TNFa500pg.mL - Media; TNFaButyrate_Combined - Me

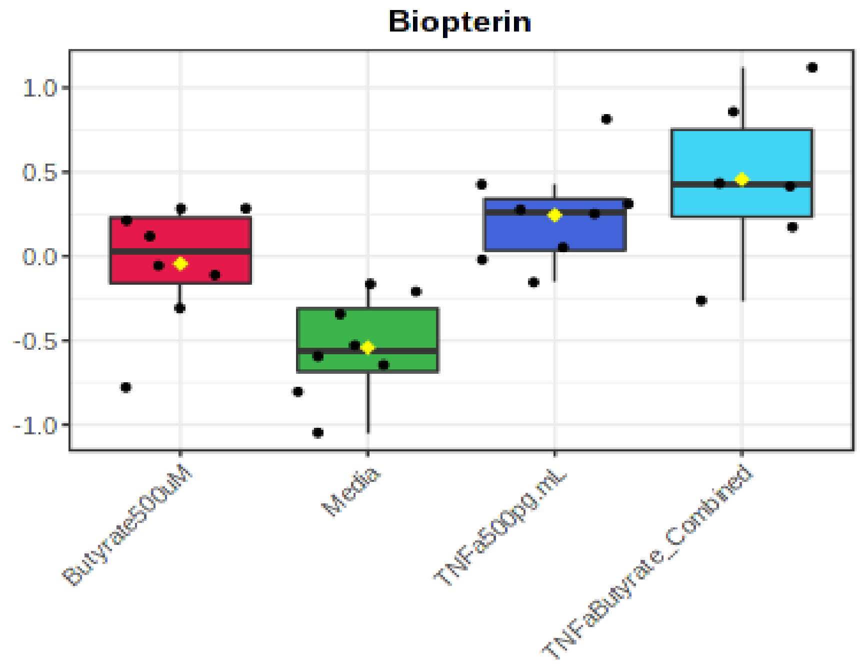

edia

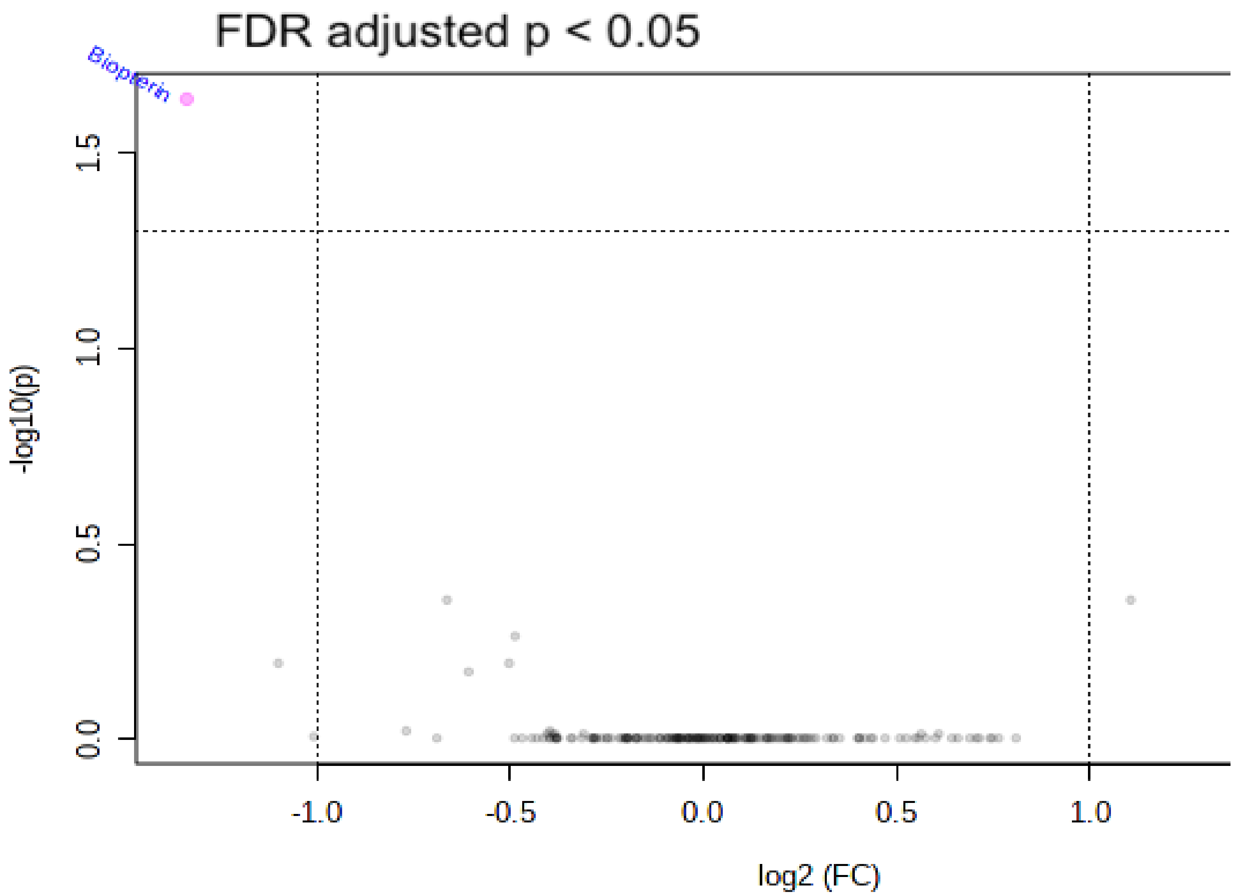
FDR adjusted p < 0.05
Biopterin
-log10(p)
0.0
0.5
1.0
1.5
-1.0
-0.5
0.0
0.5
1.0
log2 (FC)

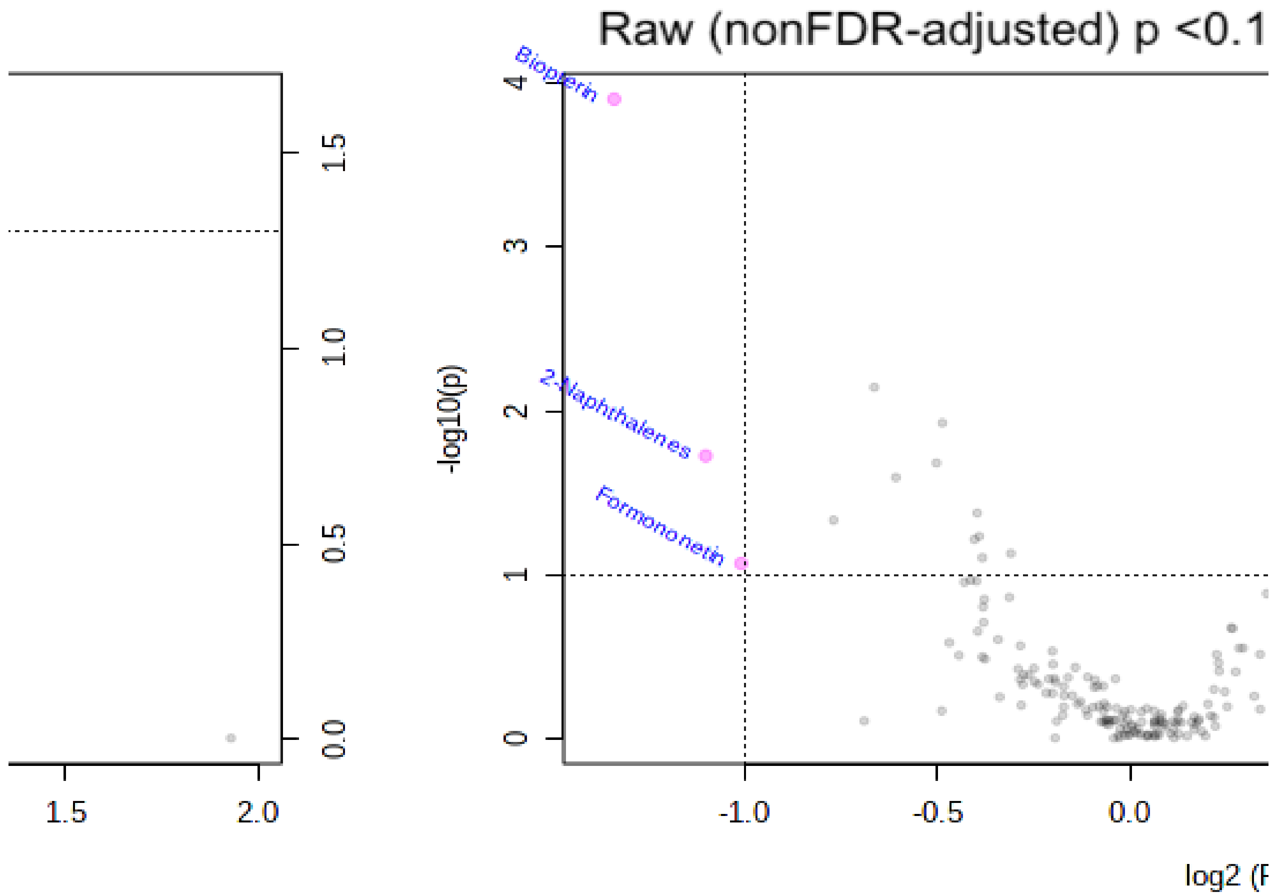
Raw (nonFDR-adjusted) p <0.1
Biopterin
2-Naphthalenes
Formononetin
-log10(p)
log2 (F
1.5
2.0
0.0
0.5
1.0
1.5
0
1
2
3
4
-1.0
-0.5
0.0

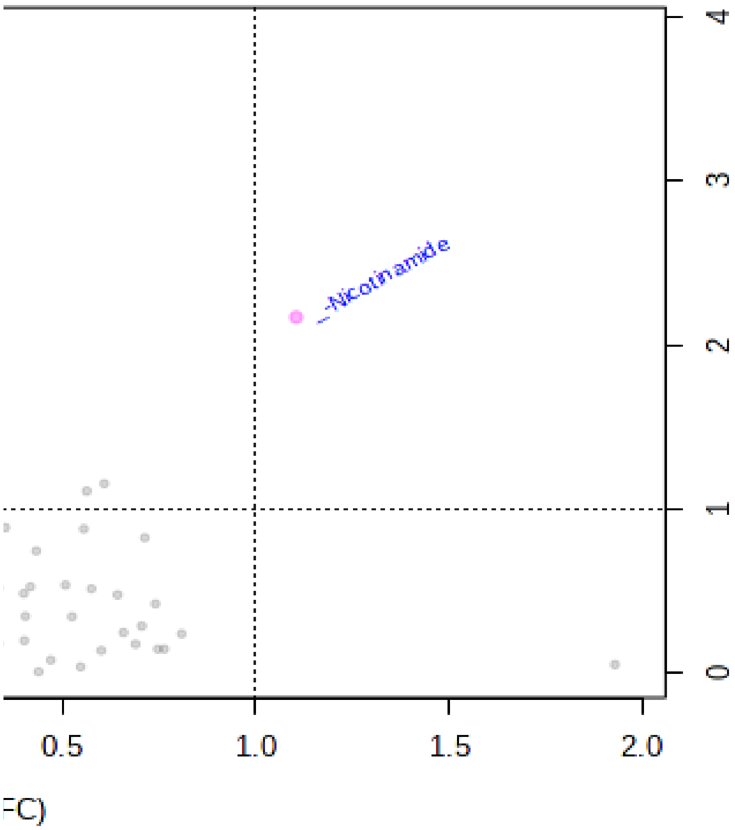

Nicotinamide
0.5
1.0
1.5
2.0
FC)
0
1
2
3
4

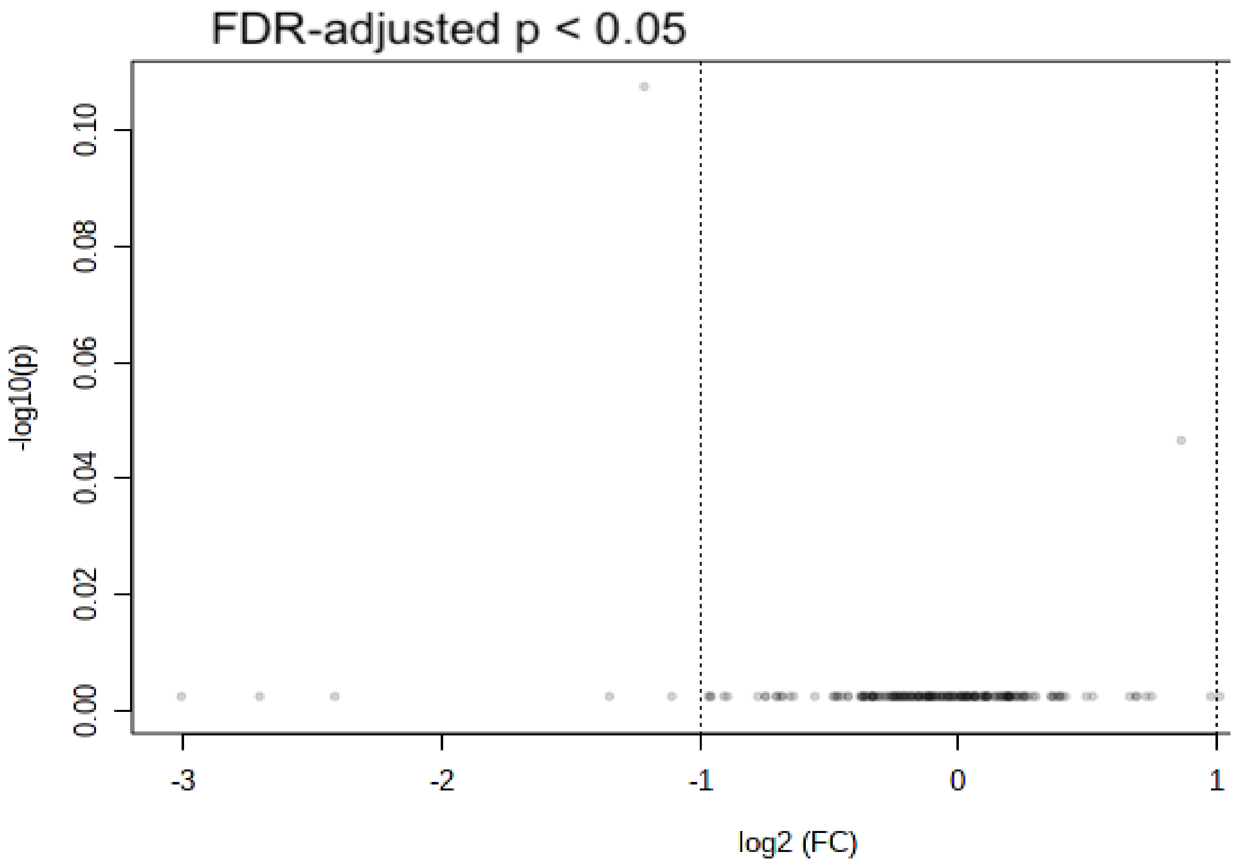

FDR-adjusted p < 0.05
-log10(p)
0.00
0.02
0.04
0.06
0.08
0.10
-3
-2
-1
0
1
log2 (FC)

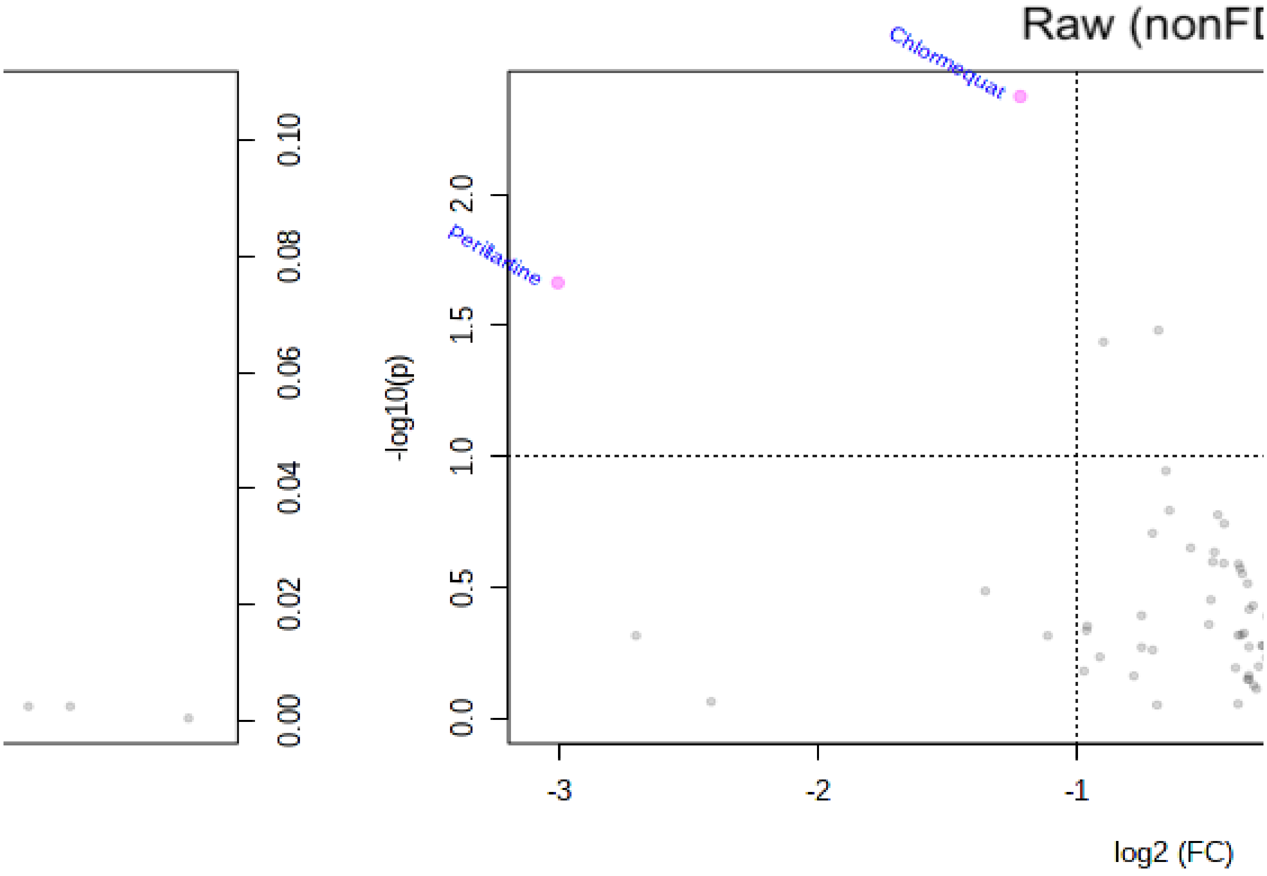

Raw (nonFD
Chlormequat
Perillartine
-log10(p)
log2 (FC)
0.10
0.08
0.06
0.04
0.02
0.00
2.0
1.5
1.0
0.5
0.0
-3
-2
-1

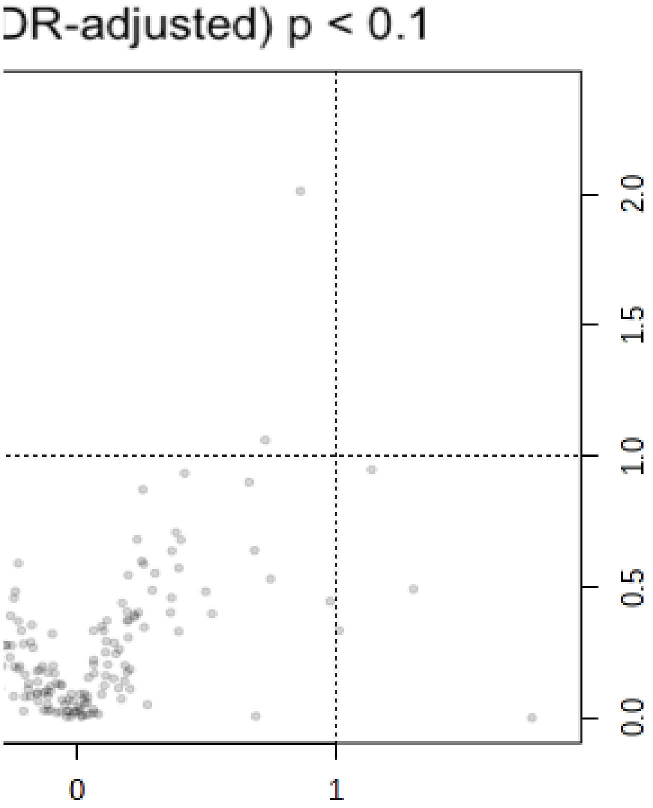
DR-adjusted) p < 0.1
0.0
0.5
1.0
1.5
2.0
0
1

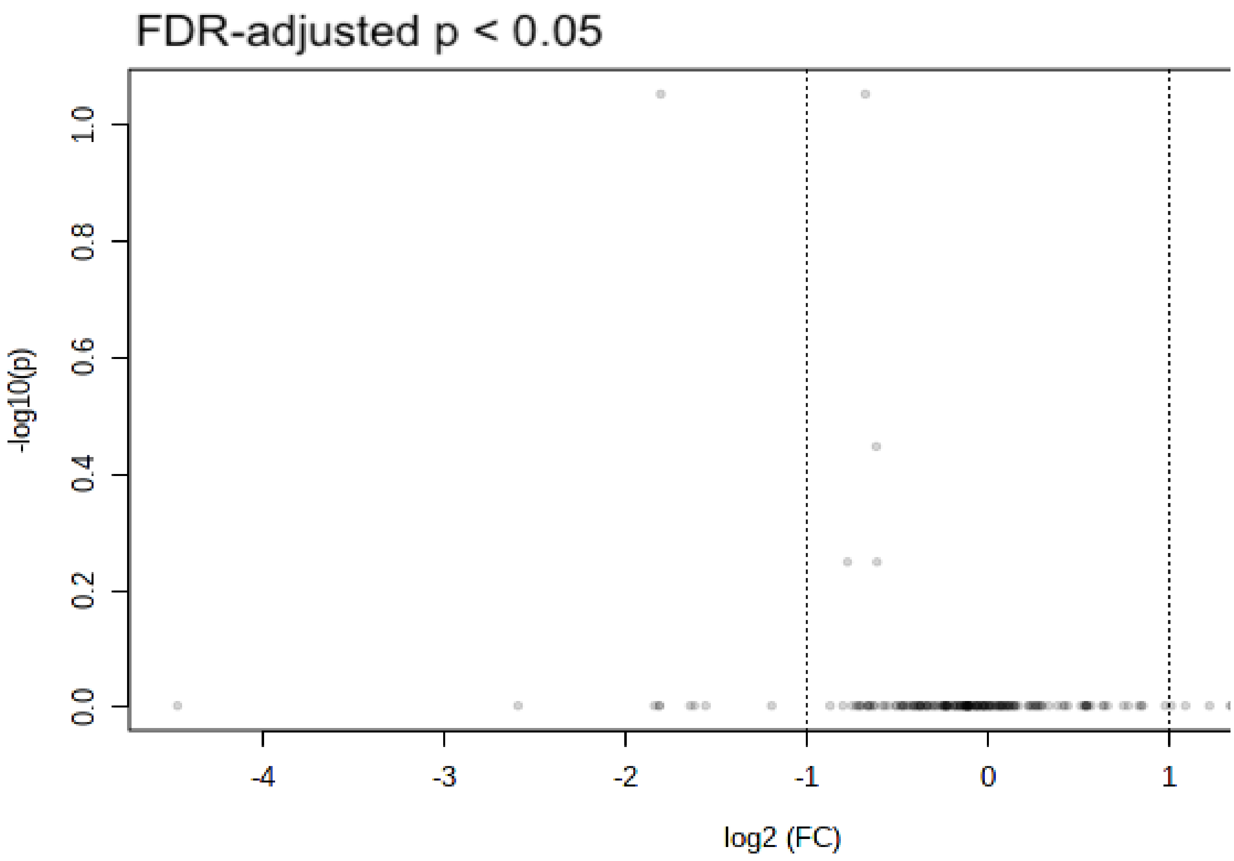

FDR-adjusted p < 0.05
-log10(p)
0.0
0.2
0.4
0.6
0.8
1.0
-4
-3
-2
-1
0
1
log2 (FC)

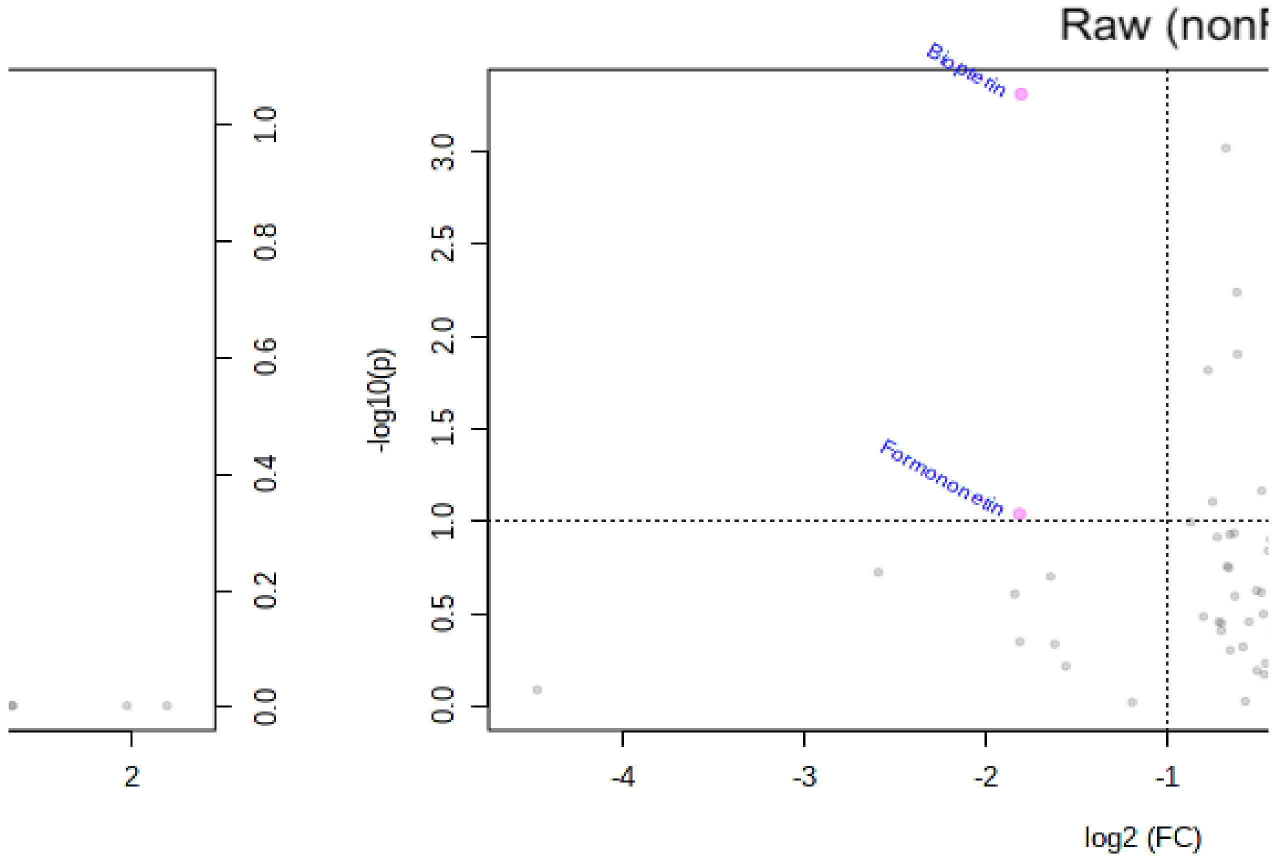
Raw (nonF
Biopterin
Formononetin
-log10(p)
log2 (FC)
0.0
0.2
0.4
0.6
0.8
1.0
2
0.0
0.5
1.0
1.5
2.0
2.5
3.0
-4
-3
-2
-1

FDR-adjusted p < 0.1

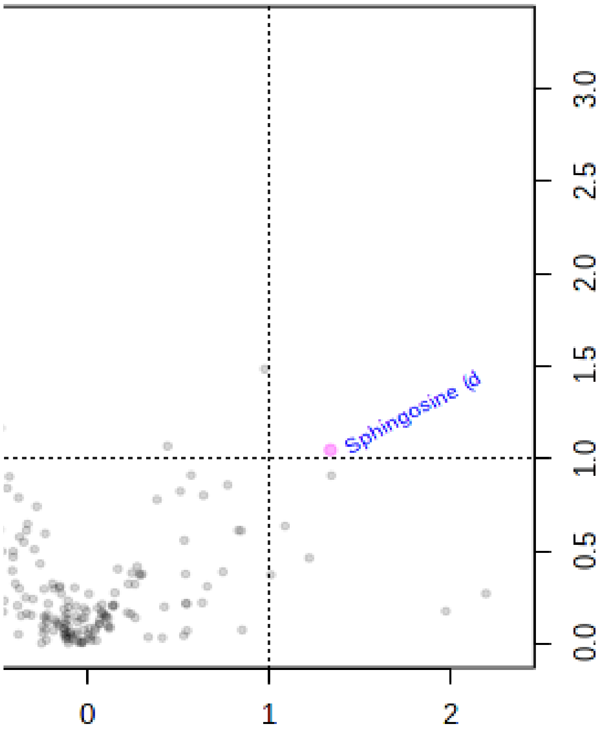

Treatment	Dunn's multiple comparisons test	Mean rank	Significant'	Summary	
Media vs lysis	Media vs. Lysis	82.17	Yes	**	A-B
Media vs TNF alpha	Media vs. 50pg/mL	21.5	No	ns	A-C
Media vs TNF alpha	Media vs. 100pg/mL	7.167	No	ns	A-D
Media vs TNF alpha	Media vs. 150pg/mL	31.17	No	ns	A-E
Media vs TNF alpha	Media vs. 200pg/mL	37.5	No	ns	A-F
Media vs TNF alpha	Media vs. 250pg/mL	35.17	No	ns	A-G
Media vs TNF alpha	Media vs. 500pg/mL	48.5	No	ns	A-H
Media vs TNF alpha	Media vs. 1ng/mL	59.17	No	ns	A-I
Media vs TNF alpha	Media vs. 2.5ng/mL	73.17	Yes	*	A-J
Media vs TNF alpha	Media vs. 5ng/mL	76.17	Yes	*	A-K
Media vs TNF alpha	Media vs. 10ng/mL	79.17	Yes	**	A-L
Media vs butyrate	Media vs. 50μM	7.167	No	ns	A-M
Media vs butyrate	Media vs. 75μM	7.833	No	ns	A-N
Media vs butyrate	Media vs. 100μM	12.17	No	ns	A-O
Media vs butyrate	Media vs. 250μM	0.8333	No	ns	A-P
Media vs butyrate	Media vs. 500μM	19.17	No	ns	A-Q
Media vs butyrate	Media vs. 750μM	30.83	No	ns	A-R
Media vs butyrate	Media vs. 1mM	42.5	No	ns	A-S
Media vs butyrate	Media vs. 2.5mM	55.17	No	ns	A-T
Media vs butyrate	Media vs. 5mM	60.17	No	ns	A-U
Media vs butyrate	Media vs. 7.5mM	65.17	No	ns	A-V
Media vs butyrate	Media vs. 10mM	70.17	No	ns	A-W
Media vs butyrate	Media vs. 25mM	66.17	No	ns	A-X
Media vs. Combo	Media vs. 50pg/mL	16.17	No	ns	A-Y
Media vs. Combo	Media vs. 150pg/mL	26.17	No	ns	A-Z
Media vs. Combo	Media vs. 250pg/mL	30.5	No	ns	A-AA
Media vs. Combo	Media vs. 500pg/mL	46.83	No	ns	A-AB
Media vs. Combo	Media vs. 1ng/mL	52.17	No	ns	A-AC
Lysis vs TNF alpha	Lysis vs. 50pg/mL	-60.67	No	ns	B-C
Lysis vs TNF alpha	Lysis vs. 100pg/mL	-75	No	ns	B-D
Lysis vs TNF alpha	Lysis vs. 150pg/mL	-51	No	ns	B-E
Lysis vs TNF alpha	Lysis vs. 200pg/mL	-44.67	No	ns	B-F
Lysis vs TNF alpha	Lysis vs. 250pg/mL	-47	No	ns	B-G
Lysis vs TNF alpha	Lysis vs. 500pg/mL	-33.67	No	ns	B-H
Lysis vs TNF alpha	Lysis vs. 1ng/mL	-23	No	ns	B-I
Lysis vs TNF alpha	Lysis vs. 2.5ng/mL	-9	No	ns	B-J
Lysis vs TNF alpha	Lysis vs. 5ng/mL	-6	No	ns	B-K
Lysis vs TNF alpha	Lysis vs. 10ng/mL	-3	No	ns	B-L
Lysis vs butyrate	Lysis vs. 50μM	-75	No	ns	B-M
Lysis vs butyrate	Lysis vs. 75μM	-74.33	No	ns	B-N
Lysis vs butyrate	Lysis vs. 100μM	-70	No	ns	B-O
Lysis vs butyrate	Lysis vs. 250μM	-81.33	No	ns	B-P
Lysis vs butyrate	Lysis vs. 500μM	-63	No	ns	B-Q
Lysis vs butyrate	Lysis vs. 750μM	-51.33	No	ns	B-R
Lysis vs butyrate	Lysis vs. 1mM	-39.67	No	ns	B-S
Lysis vs butyrate	Lysis vs. 2.5mM	-27	No	ns	B-T

Lysis vs butyrate	Lysis vs. 5mM	-22	No	ns	B-U
Lysis vs butyrate	Lysis vs. 7.5mM	-17	No	ns	B-V
Lysis vs butyrate	Lysis vs. 10mM	-12	No	ns	B-W
Lysis vs butyrate	Lysis vs. 25mM	-16	No	ns	B-X
Lysis vs. Combo	Lysis vs. 50pg/mL	-66	No	ns	B-Y
Lysis vs. Combo	Lysis vs. 150pg/mL	-56	No	ns	B-Z
Lysis vs. Combo	Lysis vs. 250pg/mL	-51.67	No	ns	B-AA
Lysis vs. Combo	Lysis vs. 500pg/mL	-35.33	No	ns	B-AB
Lysis vs. Combo	Lysis vs. 1ng/mL	-30	No	ns	B-AC
TNFa vs. TNFa	50pg/mL vs. 100pg/mL	-14.33	No	ns	C-D
TNFa vs. TNFa	50pg/mL vs. 150pg/mL	9.667	No	ns	C-E
TNFa vs. TNFa	50pg/mL vs. 200pg/mL	16	No	ns	C-F
TNFa vs. TNFa	50pg/mL vs. 250pg/mL	13.67	No	ns	C-G
TNFa vs. TNFa	50pg/mL vs. 500pg/mL	27	No	ns	C-H
TNFa vs. TNFa	50pg/mL vs. 1ng/mL	37.67	No	ns	C-I
TNFa vs. TNFa	50pg/mL vs. 2.5ng/mL	51.67	No	ns	C-J
TNFa vs. TNFa	50pg/mL vs. 5ng/mL	54.67	No	ns	C-K
TNFa vs. TNFa	50pg/mL vs. 10ng/mL	57.67	No	ns	C-L
TNFa vs. Butyrate	50pg/mL vs. 50μM	-14.33	No	ns	C-M
TNFa vs. Butyrate	50pg/mL vs. 75μM	-13.67	No	ns	C-N
TNFa vs. Butyrate	50pg/mL vs. 100μM	-9.333	No	ns	C-O
TNFa vs. Butyrate	50pg/mL vs. 250μM	-20.67	No	ns	C-P
TNFa vs. Butyrate	50pg/mL vs. 500μM	-2.333	No	ns	C-Q
TNFa vs. Butyrate	50pg/mL vs. 750μM	9.333	No	ns	C-R
TNFa vs. Butyrate	50pg/mL vs. 1mM	21	No	ns	C-S
TNFa vs. Butyrate	50pg/mL vs. 2.5mM	33.67	No	ns	C-T
TNFa vs. Butyrate	50pg/mL vs. 5mM	38.67	No	ns	C-U
TNFa vs. Butyrate	50pg/mL vs. 7.5mM	43.67	No	ns	C-V
TNFa vs. Butyrate	50pg/mL vs. 10mM	48.67	No	ns	C-W
TNFa vs. Butyrate	50pg/mL vs. 25mM	44.67	No	ns	C-X
TNFa vs. Combo	50pg/mL vs. 50pg/mL	-5.333	No	ns	C-Y
TNFa vs. Combo	50pg/mL vs. 150pg/mL	4.667	No	ns	C-Z
TNFa vs. Combo	50pg/mL vs. 250pg/mL	9	No	ns	C-AA
TNFa vs. Combo	50pg/mL vs. 500pg/mL	25.33	No	ns	C-AB
TNFa vs. Combo	50pg/mL vs. 1ng/mL	30.67	No	ns	C-AC
TNFa vs. TNFa	100pg/mL vs. 150pg/mL	24	No	ns	D-E
TNFa vs. TNFa	100pg/mL vs. 200pg/mL	30.33	No	ns	D-F
TNFa vs. TNFa	100pg/mL vs. 250pg/mL	28	No	ns	D-G
TNFa vs. TNFa	100pg/mL vs. 500pg/mL	41.33	No	ns	D-H
TNFa vs. TNFa	100pg/mL vs. 1ng/mL	52	No	ns	D-I
TNFa vs. TNFa	100pg/mL vs. 2.5ng/mL	66	No	ns	D-J
TNFa vs. TNFa	100pg/mL vs. 5ng/mL	69	No	ns	D-K
TNFa vs. TNFa	100pg/mL vs. 10ng/mL	72	No	ns	D-L
TNFa vs. Butyrate	100pg/mL vs. 50μM	0	No	ns	D-M
TNFa vs. Butyrate	100pg/mL vs. 75μM	0.6667	No	ns	D-N
TNFa vs. Butyrate	100pg/mL vs. 100μM	5	No	ns	D-O
TNFa vs. Butyrate	100pg/mL vs. 250μM	-6.333	No	ns	D-P

TNFa vs. Butyrate	100pg/mL vs. 500µM	12	No	ns	D-Q
TNFa vs. Butyrate	100pg/mL vs. 750µM	23.67	No	ns	D-R
TNFa vs. Butyrate	100pg/mL vs. 1mM	35.33	No	ns	D-S
TNFa vs. Butyrate	100pg/mL vs. 2.5mM	48	No	ns	D-T
TNFa vs. Butyrate	100pg/mL vs. 5mM	53	No	ns	D-U
TNFa vs. Butyrate	100pg/mL vs. 7.5mM	58	No	ns	D-V
TNFa vs. Butyrate	100pg/mL vs. 10mM	63	No	ns	D-W
TNFa vs. Butyrate	100pg/mL vs. 25mM	59	No	ns	D-X
TNFa vs. Combo	100pg/mL vs. 50pg/mL	9	No	ns	D-Y
TNFa vs. Combo	100pg/mL vs. 150pg/mL	19	No	ns	D-Z
TNFa vs. Combo	100pg/mL vs. 250pg/mL	23.33	No	ns	D-AA
TNFa vs. Combo	100pg/mL vs. 500pg/mL	39.67	No	ns	D-AB
TNFa vs. Combo	100pg/mL vs. 1ng/mL	45	No	ns	D-AC
TNFa vs. TNFa	150pg/mL vs. 200pg/mL	6.333	No	ns	E-F
TNFa vs. TNFa	150pg/mL vs. 250pg/mL	4	No	ns	E-G
TNFa vs. TNFa	150pg/mL vs. 500pg/mL	17.33	No	ns	E-H
TNFa vs. TNFa	150pg/mL vs. 1ng/mL	28	No	ns	E-I
TNFa vs. TNFa	150pg/mL vs. 2.5ng/mL	42	No	ns	E-J
TNFa vs. TNFa	150pg/mL vs. 5ng/mL	45	No	ns	E-K
TNFa vs. TNFa	150pg/mL vs. 10ng/mL	48	No	ns	E-L
TNFa vs. Butyrate	150pg/mL vs. 50µM	-24	No	ns	E-M
TNFa vs. Butyrate	150pg/mL vs. 75µM	-23.33	No	ns	E-N
TNFa vs. Butyrate	150pg/mL vs. 100µM	-19	No	ns	E-O
TNFa vs. Butyrate	150pg/mL vs. 250µM	-30.33	No	ns	E-P
TNFa vs. Butyrate	150pg/mL vs. 500µM	-12	No	ns	E-Q
TNFa vs. Butyrate	150pg/mL vs. 750µM	-0.3333	No	ns	E-R
TNFa vs. Butyrate	150pg/mL vs. 1mM	11.33	No	ns	E-S
TNFa vs. Butyrate	150pg/mL vs. 2.5mM	24	No	ns	E-T
TNFa vs. Butyrate	150pg/mL vs. 5mM	29	No	ns	E-U
TNFa vs. Butyrate	150pg/mL vs. 7.5mM	34	No	ns	E-V
TNFa vs. Butyrate	150pg/mL vs. 10mM	39	No	ns	E-W
TNFa vs. Butyrate	150pg/mL vs. 25mM	35	No	ns	E-X
TNFa vs. Combo	150pg/mL vs. 50pg/mL	-15	No	ns	E-Y
TNFa vs. Combo	150pg/mL vs. 150pg/mL	-5	No	ns	E-Z
TNFa vs. Combo	150pg/mL vs. 250pg/mL	-0.6667	No	ns	E-AA
TNFa vs. Combo	150pg/mL vs. 500pg/mL	15.67	No	ns	E-AB
TNFa vs. Combo	150pg/mL vs. 1ng/mL	21	No	ns	E-AC
TNFa vs. TNFa	200pg/mL vs. 250pg/mL	-2.333	No	ns	F-G
TNFa vs. TNFa	200pg/mL vs. 500pg/mL	11	No	ns	F-H
TNFa vs. TNFa	200pg/mL vs. 1ng/mL	21.67	No	ns	F-I
TNFa vs. TNFa	200pg/mL vs. 2.5ng/mL	35.67	No	ns	F-J
TNFa vs. TNFa	200pg/mL vs. 5ng/mL	38.67	No	ns	F-K
TNFa vs. TNFa	200pg/mL vs. 10ng/mL	41.67	No	ns	F-L
TNFa vs. Butyrate	200pg/mL vs. 50µM	-30.33	No	ns	F-M
TNFa vs. Butyrate	200pg/mL vs. 75µM	-29.67	No	ns	F-N
TNFa vs. Butyrate	200pg/mL vs. 100µM	-25.33	No	ns	F-O
TNFa vs. Butyrate	200pg/mL vs. 250µM	-36.67	No	ns	F-P

TNFa vs. Butyrate	200pg/mL vs. 500µM	-18.33 No	ns	F-Q
TNFa vs. Butyrate	200pg/mL vs. 750µM	-6.667 No	ns	F-R
TNFa vs. Butyrate	200pg/mL vs. 1mM	5 No	ns	F-S
TNFa vs. Butyrate	200pg/mL vs. 2.5mM	17.67 No	ns	F-T
TNFa vs. Butyrate	200pg/mL vs. 5mM	22.67 No	ns	F-U
TNFa vs. Butyrate	200pg/mL vs. 7.5mM	27.67 No	ns	F-V
TNFa vs. Butyrate	200pg/mL vs. 10mM	32.67 No	ns	F-W
TNFa vs. Butyrate	200pg/mL vs. 25mM	28.67 No	ns	F-X
TNFa vs. Combo	200pg/mL vs. 50pg/mL	-21.33 No	ns	F-Y
TNFa vs. Combo	200pg/mL vs. 150pg/mL	-11.33 No	ns	F-Z
TNFa vs. Combo	200pg/mL vs. 250pg/mL	-7 No	ns	F-AA
TNFa vs. Combo	200pg/mL vs. 500pg/mL	9.333 No	ns	F-AB
TNFa vs. Combo	200pg/mL vs. 1ng/mL	14.67 No	ns	F-AC
TNFa vs. TNFa	250pg/mL vs. 500pg/mL	13.33 No	ns	G-H
TNFa vs. TNFa	250pg/mL vs. 1ng/mL	24 No	ns	G-I
TNFa vs. TNFa	250pg/mL vs. 2.5ng/mL	38 No	ns	G-J
TNFa vs. TNFa	250pg/mL vs. 5ng/mL	41 No	ns	G-K
TNFa vs. TNFa	250pg/mL vs. 10ng/mL	44 No	ns	G-L
TNFa vs. Butyrate	250pg/mL vs. 50µM	-28 No	ns	G-M
TNFa vs. Butyrate	250pg/mL vs. 75µM	-27.33 No	ns	G-N
TNFa vs. Butyrate	250pg/mL vs. 100µM	-23 No	ns	G-O
TNFa vs. Butyrate	250pg/mL vs. 250µM	-34.33 No	ns	G-P
TNFa vs. Butyrate	250pg/mL vs. 500µM	-16 No	ns	G-Q
TNFa vs. Butyrate	250pg/mL vs. 750µM	-4.333 No	ns	G-R
TNFa vs. Butyrate	250pg/mL vs. 1mM	7.333 No	ns	G-S
TNFa vs. Butyrate	250pg/mL vs. 2.5mM	20 No	ns	G-T
TNFa vs. Butyrate	250pg/mL vs. 5mM	25 No	ns	G-U
TNFa vs. Butyrate	250pg/mL vs. 7.5mM	30 No	ns	G-V
TNFa vs. Butyrate	250pg/mL vs. 10mM	35 No	ns	G-W
TNFa vs. Butyrate	250pg/mL vs. 25mM	31 No	ns	G-X
TNFa vs. Combo	250pg/mL vs. 50pg/mL	-19 No	ns	G-Y
TNFa vs. Combo	250pg/mL vs. 150pg/mL	-9 No	ns	G-Z
TNFa vs. Combo	250pg/mL vs. 250pg/mL	-4.667 No	ns	G-AA
TNFa vs. Combo	250pg/mL vs. 500pg/mL	11.67 No	ns	G-AB
TNFa vs. Combo	250pg/mL vs. 1ng/mL	17 No	ns	G-AC
TNFa vs. TNFa	500pg/mL vs. 1ng/mL	10.67 No	ns	H-I
TNFa vs. TNFa	500pg/mL vs. 2.5ng/mL	24.67 No	ns	H-J
TNFa vs. TNFa	500pg/mL vs. 5ng/mL	27.67 No	ns	H-K
TNFa vs. TNFa	500pg/mL vs. 10ng/mL	30.67 No	ns	H-L
TNFa vs. Butyrate	500pg/mL vs. 50µM	-41.33 No	ns	H-M
TNFa vs. Butyrate	500pg/mL vs. 75µM	-40.67 No	ns	H-N
TNFa vs. Butyrate	500pg/mL vs. 100µM	-36.33 No	ns	H-O
TNFa vs. Butyrate	500pg/mL vs. 250µM	-47.67 No	ns	H-P
TNFa vs. Butyrate	500pg/mL vs. 500µM	-29.33 No	ns	H-Q
TNFa vs. Butyrate	500pg/mL vs. 750µM	-17.67 No	ns	H-R
TNFa vs. Butyrate	500pg/mL vs. 1mM	-6 No	ns	H-S
TNFa vs. Butyrate	500pg/mL vs. 2.5mM	6.667 No	ns	H-T

TNFa vs. Butyrate	500pg/mL vs. 5mM	11.67	No	ns	H-U
TNFa vs. Butyrate	500pg/mL vs. 7.5mM	16.67	No	ns	H-V
TNFa vs. Butyrate	500pg/mL vs. 10mM	21.67	No	ns	H-W
TNFa vs. Butyrate	500pg/mL vs. 25mM	17.67	No	ns	H-X
TNFa vs. Combo	500pg/mL vs. 50pg/mL	-32.33	No	ns	H-Y
TNFa vs. Combo	500pg/mL vs. 150pg/mL	-22.33	No	ns	H-Z
TNFa vs. Combo	500pg/mL vs. 250pg/mL	-18	No	ns	H-AA
TNFa vs. Combo	500pg/mL vs. 500pg/mL	-1.667	No	ns	H-AB
TNFa vs. Combo	500pg/mL vs. 1ng/mL	3.667	No	ns	H-AC
TNFa vs. TNFa	1ng/mL vs. 2.5ng/mL	14	No	ns	I-J
TNFa vs. TNFa	1ng/mL vs. 5ng/mL	17	No	ns	I-K
TNFa vs. TNFa	1ng/mL vs. 10ng/mL	20	No	ns	I-L
TNFa vs. Butyrate	1ng/mL vs. 50µM	-52	No	ns	I-M
TNFa vs. Butyrate	1ng/mL vs. 75µM	-51.33	No	ns	I-N
TNFa vs. Butyrate	1ng/mL vs. 100µM	-47	No	ns	I-O
TNFa vs. Butyrate	1ng/mL vs. 250µM	-58.33	No	ns	I-P
TNFa vs. Butyrate	1ng/mL vs. 500µM	-40	No	ns	I-Q
TNFa vs. Butyrate	1ng/mL vs. 750µM	-28.33	No	ns	I-R
TNFa vs. Butyrate	1ng/mL vs. 1mM	-16.67	No	ns	I-S
TNFa vs. Butyrate	1ng/mL vs. 2.5mM	-4	No	ns	I-T
TNFa vs. Butyrate	1ng/mL vs. 5mM	1	No	ns	I-U
TNFa vs. Butyrate	1ng/mL vs. 7.5mM	6	No	ns	I-V
TNFa vs. Butyrate	1ng/mL vs. 10mM	11	No	ns	I-W
TNFa vs. Butyrate	1ng/mL vs. 25mM	7	No	ns	I-X
TNFa vs. Combo	1ng/mL vs. 50pg/mL	-43	No	ns	I-Y
TNFa vs. Combo	1ng/mL vs. 150pg/mL	-33	No	ns	I-Z
TNFa vs. Combo	1ng/mL vs. 250pg/mL	-28.67	No	ns	I-AA
TNFa vs. Combo	1ng/mL vs. 500pg/mL	-12.33	No	ns	I-AB
TNFa vs. Combo	1ng/mL vs. 1ng/mL	-7	No	ns	I-AC
TNFa vs. TNFa	2.5ng/mL vs. 5ng/mL	3	No	ns	J-K
TNFa vs. TNFa	2.5ng/mL vs. 10ng/mL	6	No	ns	J-L
TNFa vs. Butyrate	2.5ng/mL vs. 50µM	-66	No	ns	J-M
TNFa vs. Butyrate	2.5ng/mL vs. 75µM	-65.33	No	ns	J-N
TNFa vs. Butyrate	2.5ng/mL vs. 100µM	-61	No	ns	J-O
TNFa vs. Butyrate	2.5ng/mL vs. 250µM	-72.33	No	ns	J-P
TNFa vs. Butyrate	2.5ng/mL vs. 500µM	-54	No	ns	J-Q
TNFa vs. Butyrate	2.5ng/mL vs. 750µM	-42.33	No	ns	J-R
TNFa vs. Butyrate	2.5ng/mL vs. 1mM	-30.67	No	ns	J-S
TNFa vs. Butyrate	2.5ng/mL vs. 2.5mM	-18	No	ns	J-T
TNFa vs. Butyrate	2.5ng/mL vs. 5mM	-13	No	ns	J-U
TNFa vs. Butyrate	2.5ng/mL vs. 7.5mM	-8	No	ns	J-V
TNFa vs. Butyrate	2.5ng/mL vs. 10mM	-3	No	ns	J-W
TNFa vs. Butyrate	2.5ng/mL vs. 25mM	-7	No	ns	J-X
TNFa vs. Combo	2.5ng/mL vs. 50pg/mL	-57	No	ns	J-Y
TNFa vs. Combo	2.5ng/mL vs. 150pg/mL	-47	No	ns	J-Z
TNFa vs. Combo	2.5ng/mL vs. 250pg/mL	-42.67	No	ns	J-AA
TNFa vs. Combo	2.5ng/mL vs. 500pg/mL	-26.33	No	ns	J-AB

TNFa vs. Combo	2.5ng/mL vs. 1ng/mL	-21	No	ns	J-AC
TNFa vs. TNFa	5ng/mL vs. 10ng/mL	3	No	ns	K-L
TNFa vs. Butyrate	5ng/mL vs. 50μM	-69	No	ns	K-M
TNFa vs. Butyrate	5ng/mL vs. 75μM	-68.33	No	ns	K-N
TNFa vs. Butyrate	5ng/mL vs. 100μM	-64	No	ns	K-O
TNFa vs. Butyrate	5ng/mL vs. 250μM	-75.33	No	ns	K-P
TNFa vs. Butyrate	5ng/mL vs. 500μM	-57	No	ns	K-Q
TNFa vs. Butyrate	5ng/mL vs. 750μM	-45.33	No	ns	K-R
TNFa vs. Butyrate	5ng/mL vs. 1mM	-33.67	No	ns	K-S
TNFa vs. Butyrate	5ng/mL vs. 2.5mM	-21	No	ns	K-T
TNFa vs. Butyrate	5ng/mL vs. 5mM	-16	No	ns	K-U
TNFa vs. Butyrate	5ng/mL vs. 7.5mM	-11	No	ns	K-V
TNFa vs. Butyrate	5ng/mL vs. 10mM	-6	No	ns	K-W
TNFa vs. Butyrate	5ng/mL vs. 25mM	-10	No	ns	K-X
TNFa vs. Combo	5ng/mL vs. 50pg/mL	-60	No	ns	K-Y
TNFa vs. Combo	5ng/mL vs. 150pg/mL	-50	No	ns	K-Z
TNFa vs. Combo	5ng/mL vs. 250pg/mL	-45.67	No	ns	K-AA
TNFa vs. Combo	5ng/mL vs. 500pg/mL	-29.33	No	ns	K-AB
TNFa vs. Combo	5ng/mL vs. 1ng/mL	-24	No	ns	K-AC
TNFa vs. Butyrate	10ng/mL vs. 50μM	-72	No	ns	L-M
TNFa vs. Butyrate	10ng/mL vs. 75μM	-71.33	No	ns	L-N
TNFa vs. Butyrate	10ng/mL vs. 100μM	-67	No	ns	L-O
TNFa vs. Butyrate	10ng/mL vs. 250μM	-78.33	No	ns	L-P
TNFa vs. Butyrate	10ng/mL vs. 500μM	-60	No	ns	L-Q
TNFa vs. Butyrate	10ng/mL vs. 750μM	-48.33	No	ns	L-R
TNFa vs. Butyrate	10ng/mL vs. 1mM	-36.67	No	ns	L-S
TNFa vs. Butyrate	10ng/mL vs. 2.5mM	-24	No	ns	L-T
TNFa vs. Butyrate	10ng/mL vs. 5mM	-19	No	ns	L-U
TNFa vs. Butyrate	10ng/mL vs. 7.5mM	-14	No	ns	L-V
TNFa vs. Butyrate	10ng/mL vs. 10mM	-9	No	ns	L-W
TNFa vs. Butyrate	10ng/mL vs. 25mM	-13	No	ns	L-X
TNFa vs. Combo	10ng/mL vs. 50pg/mL	-63	No	ns	L-Y
TNFa vs. Combo	10ng/mL vs. 150pg/mL	-53	No	ns	L-Z
TNFa vs. Combo	10ng/mL vs. 250pg/mL	-48.67	No	ns	L-AA
TNFa vs. Combo	10ng/mL vs. 500pg/mL	-32.33	No	ns	L-AB
TNFa vs. Combo	10ng/mL vs. 1ng/mL	-27	No	ns	L-AC
Butyrate vs. Butyrate	50μM vs. 75μM	0.6667	No	ns	M-N
Butyrate vs. Butyrate	50μM vs. 100μM	5	No	ns	M-O
Butyrate vs. Butyrate	50μM vs. 250μM	-6.333	No	ns	M-P
Butyrate vs. Butyrate	50μM vs. 500μM	12	No	ns	M-Q
Butyrate vs. Butyrate	50μM vs. 750μM	23.67	No	ns	M-R
Butyrate vs. Butyrate	50μM vs. 1mM	35.33	No	ns	M-S
Butyrate vs. Butyrate	50μM vs. 2.5mM	48	No	ns	M-T
Butyrate vs. Butyrate	50μM vs. 5mM	53	No	ns	M-U
Butyrate vs. Butyrate	50μM vs. 7.5mM	58	No	ns	M-V
Butyrate vs. Butyrate	50μM vs. 10mM	63	No	ns	M-W
Butyrate vs. Butyrate	50μM vs. 25mM	59	No	ns	M-X

Butyrate vs. Combo	50µM vs. 50pg/mL	9	No	ns	M-Y
Butyrate vs. Combo	50µM vs. 150pg/mL	19	No	ns	M-Z
Butyrate vs. Combo	50µM vs. 250pg/mL	23.33	No	ns	M-AA
Butyrate vs. Combo	50µM vs. 500pg/mL	39.67	No	ns	M-AB
Butyrate vs. Combo	50µM vs. 1ng/mL	45	No	ns	M-AC
Butyrate vs. Butyrate	75µM vs. 100µM	4.333	No	ns	N-O
Butyrate vs. Butyrate	75µM vs. 250µM	-7	No	ns	N-P
Butyrate vs. Butyrate	75µM vs. 500µM	11.33	No	ns	N-Q
Butyrate vs. Butyrate	75µM vs. 750µM	23	No	ns	N-R
Butyrate vs. Butyrate	75µM vs. 1mM	34.67	No	ns	N-S
Butyrate vs. Butyrate	75µM vs. 2.5mM	47.33	No	ns	N-T
Butyrate vs. Butyrate	75µM vs. 5mM	52.33	No	ns	N-U
Butyrate vs. Butyrate	75µM vs. 7.5mM	57.33	No	ns	N-V
Butyrate vs. Butyrate	75µM vs. 10mM	62.33	No	ns	N-W
Butyrate vs. Butyrate	75µM vs. 25mM	58.33	No	ns	N-X
Butyrate vs. Combo	75µM vs. 50pg/mL	8.333	No	ns	N-Y
Butyrate vs. Combo	75µM vs. 150pg/mL	18.33	No	ns	N-Z
Butyrate vs. Combo	75µM vs. 250pg/mL	22.67	No	ns	N-AA
Butyrate vs. Combo	75µM vs. 500pg/mL	39	No	ns	N-AB
Butyrate vs. Combo	75µM vs. 1ng/mL	44.33	No	ns	N-AC
Butyrate vs. Butyrate	100µM vs. 250µM	-11.33	No	ns	O-P
Butyrate vs. Butyrate	100µM vs. 500µM	7	No	ns	O-Q
Butyrate vs. Butyrate	100µM vs. 750µM	18.67	No	ns	O-R
Butyrate vs. Butyrate	100µM vs. 1mM	30.33	No	ns	O-S
Butyrate vs. Butyrate	100µM vs. 2.5mM	43	No	ns	O-T
Butyrate vs. Butyrate	100µM vs. 5mM	48	No	ns	O-U
Butyrate vs. Butyrate	100µM vs. 7.5mM	53	No	ns	O-V
Butyrate vs. Butyrate	100µM vs. 10mM	58	No	ns	O-W
Butyrate vs. Butyrate	100µM vs. 25mM	54	No	ns	O-X
Butyrate vs. Combo	100µM vs. 50pg/mL	4	No	ns	O-Y
Butyrate vs. Combo	100µM vs. 150pg/mL	14	No	ns	O-Z
Butyrate vs. Combo	100µM vs. 250pg/mL	18.33	No	ns	O-AA
Butyrate vs. Combo	100µM vs. 500pg/mL	34.67	No	ns	O-AB
Butyrate vs. Combo	100µM vs. 1ng/mL	40	No	ns	O-AC
Butyrate vs. Butyrate	250µM vs. 500µM	18.33	No	ns	P-Q
Butyrate vs. Butyrate	250µM vs. 750µM	30	No	ns	P-R
Butyrate vs. Butyrate	250µM vs. 1mM	41.67	No	ns	P-S
Butyrate vs. Butyrate	250µM vs. 2.5mM	54.33	No	ns	P-T
Butyrate vs. Butyrate	250µM vs. 5mM	59.33	No	ns	P-U
Butyrate vs. Butyrate	250µM vs. 7.5mM	64.33	No	ns	P-V
Butyrate vs. Butyrate	250µM vs. 10mM	69.33	No	ns	P-W
Butyrate vs. Butyrate	250µM vs. 25mM	65.33	No	ns	P-X
Butyrate vs. Combo	250µM vs. 50pg/mL	15.33	No	ns	P-Y
Butyrate vs. Combo	250µM vs. 150pg/mL	25.33	No	ns	P-Z
Butyrate vs. Combo	250µM vs. 250pg/mL	29.67	No	ns	P-AA
Butyrate vs. Combo	250µM vs. 500pg/mL	46	No	ns	P-AB
Butyrate vs. Combo	250µM vs. 1ng/mL	51.33	No	ns	P-AC

Butyrate vs. Butyrate	500µM vs. 750µM	11.67	No	ns	Q-R
Butyrate vs. Butyrate	500µM vs. 1mM	23.33	No	ns	Q-S
Butyrate vs. Butyrate	500µM vs. 2.5mM	36	No	ns	Q-T
Butyrate vs. Butyrate	500µM vs. 5mM	41	No	ns	Q-U
Butyrate vs. Butyrate	500µM vs. 7.5mM	46	No	ns	Q-V
Butyrate vs. Butyrate	500µM vs. 10mM	51	No	ns	Q-W
Butyrate vs. Butyrate	500µM vs. 25mM	47	No	ns	Q-X
Butyrate vs. Combo	500µM vs. 50pg/mL	-3	No	ns	Q-Y
Butyrate vs. Combo	500µM vs. 150pg/mL	7	No	ns	Q-Z
Butyrate vs. Combo	500µM vs. 250pg/mL	11.33	No	ns	Q-AA
Butyrate vs. Combo	500µM vs. 500pg/mL	27.67	No	ns	Q-AB
Butyrate vs. Combo	500µM vs. 1ng/mL	33	No	ns	Q-AC
Butyrate vs. Butyrate	750µM vs. 1mM	11.67	No	ns	R-S
Butyrate vs. Butyrate	750µM vs. 2.5mM	24.33	No	ns	R-T
Butyrate vs. Butyrate	750µM vs. 5mM	29.33	No	ns	R-U
Butyrate vs. Butyrate	750µM vs. 7.5mM	34.33	No	ns	R-V
Butyrate vs. Butyrate	750µM vs. 10mM	39.33	No	ns	R-W
Butyrate vs. Butyrate	750µM vs. 25mM	35.33	No	ns	R-X
Butyrate vs. Combo	750µM vs. 50pg/mL	-14.67	No	ns	R-Y
Butyrate vs. Combo	750µM vs. 150pg/mL	-4.667	No	ns	R-Z
Butyrate vs. Combo	750µM vs. 250pg/mL	-0.3333	No	ns	R-AA
Butyrate vs. Combo	750µM vs. 500pg/mL	16	No	ns	R-AB
Butyrate vs. Combo	750µM vs. 1ng/mL	21.33	No	ns	R-AC
Butyrate vs. Butyrate	1mM vs. 2.5mM	12.67	No	ns	S-T
Butyrate vs. Butyrate	1mM vs. 5mM	17.67	No	ns	S-U
Butyrate vs. Butyrate	1mM vs. 7.5mM	22.67	No	ns	S-V
Butyrate vs. Butyrate	1mM vs. 10mM	27.67	No	ns	S-W
Butyrate vs. Butyrate	1mM vs. 25mM	23.67	No	ns	S-X
Butyrate vs. Combo	1mM vs. 50pg/mL	-26.33	No	ns	S-Y
Butyrate vs. Combo	1mM vs. 150pg/mL	-16.33	No	ns	S-Z
Butyrate vs. Combo	1mM vs. 250pg/mL	-12	No	ns	S-AA
Butyrate vs. Combo	1mM vs. 500pg/mL	4.333	No	ns	S-AB
Butyrate vs. Combo	1mM vs. 1ng/mL	9.667	No	ns	S-AC
Butyrate vs. Butyrate	2.5mM vs. 5mM	5	No	ns	T-U
Butyrate vs. Butyrate	2.5mM vs. 7.5mM	10	No	ns	T-V
Butyrate vs. Butyrate	2.5mM vs. 10mM	15	No	ns	T-W
Butyrate vs. Butyrate	2.5mM vs. 25mM	11	No	ns	T-X
Butyrate vs. Combo	2.5mM vs. 50pg/mL	-39	No	ns	T-Y
Butyrate vs. Combo	2.5mM vs. 150pg/mL	-29	No	ns	T-Z
Butyrate vs. Combo	2.5mM vs. 250pg/mL	-24.67	No	ns	T-AA
Butyrate vs. Combo	2.5mM vs. 500pg/mL	-8.333	No	ns	T-AB
Butyrate vs. Combo	2.5mM vs. 1ng/mL	-3	No	ns	T-AC
Butyrate vs. Butyrate	5mM vs. 7.5mM	5	No	ns	U-V
Butyrate vs. Butyrate	5mM vs. 10mM	10	No	ns	U-W
Butyrate vs. Butyrate	5mM vs. 25mM	6	No	ns	U-X
Butyrate vs. Combo	5mM vs. 50pg/mL	-44	No	ns	U-Y
Butyrate vs. Combo	5mM vs. 150pg/mL	-34	No	ns	U-Z

Butyrate vs. Combo	5mM vs. 250pg/mL	-29.67	No	ns	U-AA
Butyrate vs. Combo	5mM vs. 500pg/mL	-13.33	No	ns	U-AB
Butyrate vs. Combo	5mM vs. 1ng/mL	-8	No	ns	U-AC
Butyrate vs. Butyrate	7.5mM vs. 10mM	5	No	ns	V-W
Butyrate vs. Butyrate	7.5mM vs. 25mM	1	No	ns	V-X
Butyrate vs. Combo	7.5mM vs. 50pg/mL	-49	No	ns	V-Y
Butyrate vs. Combo	7.5mM vs. 150pg/mL	-39	No	ns	V-Z
Butyrate vs. Combo	7.5mM vs. 250pg/mL	-34.67	No	ns	V-AA
Butyrate vs. Combo	7.5mM vs. 500pg/mL	-18.33	No	ns	V-AB
Butyrate vs. Combo	7.5mM vs. 1ng/mL	-13	No	ns	V-AC
Butyrate vs. Butyrate	10mM vs. 25mM	-4	No	ns	W-X
Butyrate vs. Combo	10mM vs. 50pg/mL	-54	No	ns	W-Y
Butyrate vs. Combo	10mM vs. 150pg/mL	-44	No	ns	W-Z
Butyrate vs. Combo	10mM vs. 250pg/mL	-39.67	No	ns	W-AA
Butyrate vs. Combo	10mM vs. 500pg/mL	-23.33	No	ns	W-AB
Butyrate vs. Combo	10mM vs. 1ng/mL	-18	No	ns	W-AC
Butyrate vs. Combo	25mM vs. 50pg/mL	-50	No	ns	X-Y
Butyrate vs. Combo	25mM vs. 150pg/mL	-40	No	ns	X-Z
Butyrate vs. Combo	25mM vs. 250pg/mL	-35.67	No	ns	X-AA
Butyrate vs. Combo	25mM vs. 500pg/mL	-19.33	No	ns	X-AB
Butyrate vs. Combo	25mM vs. 1ng/mL	-14	No	ns	X-AC
Combo vs. Combo	50pg/mL vs. 150pg/mL	10	No	ns	Y-Z
Combo vs. Combo	50pg/mL vs. 250pg/mL	14.33	No	ns	Y-AA
Combo vs. Combo	50pg/mL vs. 500pg/mL	30.67	No	ns	Y-AB
Combo vs. Combo	50pg/mL vs. 1ng/mL	36	No	ns	Y-AC
Combo vs. Combo	150pg/mL vs. 250pg/mL	4.333	No	ns	Z-AA
Combo vs. Combo	150pg/mL vs. 500pg/mL	20.67	No	ns	Z-AB
Combo vs. Combo	150pg/mL vs. 1ng/mL	26	No	ns	Z-AC
Combo vs. Combo	250pg/mL vs. 500pg/mL	16.33	No	ns	AA-AB
Combo vs. Combo	250pg/mL vs. 1ng/mL	21.67	No	ns	AA-AC
Combo vs. Combo	500pg/mL vs. 1ng/mL	5.333	No	ns	

Treatment	Dunn's multiple comparisons test	Mean rank	Significant?	Summary	Adjusted P Value	
Media vs AA	Media vs. 50µM AA	156.5	Yes	****	<0.0001	A-B
Media vs TNF alpha	Media vs. 50pg/mL	17.33	No	ns	>0.9999	A-C
Media vs TNF alpha	Media vs. 100pg/mL	1.667	No	ns	>0.9999	A-D
Media vs TNF alpha	Media vs. 150pg/mL	13	No	ns	>0.9999	A-E
Media vs TNF alpha	Media vs. 200pg/mL	-1.5	No	ns	>0.9999	A-F
Media vs TNF alpha	Media vs. 250pg/mL	65	No	ns	>0.9999	A-G
Media vs TNF alpha	Media vs. 500pg/mL	23.83	No	ns	>0.9999	A-H
Media vs TNF alpha	Media vs. 1000pg/mL	19.67	No	ns	>0.9999	A-I
Media vs TNF alpha	Media vs. 2500pg/mL	52.17	No	ns	>0.9999	A-J
Media vs TNF alpha	Media vs. 5000pg/mL	89.67	No	ns	>0.9999	A-K
Media vs butyrate	Media vs. 50µM	32	No	ns	>0.9999	A-L
Media vs butyrate	Media vs. 75µM	49.33	No	ns	>0.9999	A-M
Media vs butyrate	Media vs. 100µM	42.67	No	ns	>0.9999	A-N
Media vs butyrate	Media vs. 250µM	114.7	No	ns	0.06	A-O
Media vs butyrate	Media vs. 500µM	60.17	No	ns	>0.9999	A-P
Media vs butyrate	Media vs. 750µM	36.83	No	ns	>0.9999	A-Q
Media vs butyrate	Media vs. 1000µM	88	No	ns	>0.9999	A-R
Media vs butyrate	Media vs. 2500µM	102.7	No	ns	0.2797	A-S
Media vs butyrate	Media vs. 5000µM	95.33	No	ns	0.6654	A-T
Media vs butyrate	Media vs. 7500µM	98.83	No	ns	0.4431	A-U
Media vs Combo	Media vs. 50pg/mL	62.67	No	ns	>0.9999	A-V
Media vs Combo	Media vs. 100pg/mL	131.2	Yes	**	0.0057	A-W
Media vs Combo	Media vs. 150pg/mL	77.17	No	ns	>0.9999	A-X
Media vs Combo	Media vs. 200pg/mL	49.67	No	ns	>0.9999	A-Y
Media vs Combo	Media vs. 250pg/mL	94.67	No	ns	0.718	A-Z
Media vs Combo	Media vs. 500pg/mL	107.8	No	ns	0.1469	A-AA
Media vs Combo	Media vs. 1000pg/mL	127.8	Yes	**	0.0093	A-AB
Media vs Combo	Media vs. 2500pg/mL	145.7	Yes	***	0.0006	A-AC
Media vs Combo	Media vs. 5000pg/mL	150.5	Yes	***	0.0002	A-AD
AA vs TNF alpha	50µM AA vs. 50pg/mL	-139.2	Yes	**	0.0016	B-C
AA vs TNF alpha	50µM AA vs. 100pg/mL	-154.8	Yes	***	0.0001	B-D
AA vs TNF alpha	50µM AA vs. 150pg/mL	-143.5	Yes	***	0.0008	B-E
AA vs TNF alpha	50µM AA vs. 200pg/mL	-158	Yes	****	<0.0001	B-F
AA vs TNF alpha	50µM AA vs. 250pg/mL	-91.5	No	ns	>0.9999	B-G
AA vs TNF alpha	50µM AA vs. 500pg/mL	-132.7	Yes	**	0.0045	B-H
AA vs TNF alpha	50µM AA vs. 1000pg/mL	-136.8	Yes	**	0.0024	B-I
AA vs TNF alpha	50µM AA vs. 2500pg/mL	-104.3	No	ns	0.228	B-J
AA vs TNF alpha	50µM AA vs. 5000pg/mL	-66.83	No	ns	>0.9999	B-K
AA vs butyrate	50µM AA vs. 50µM	-124.5	Yes	*	0.0152	B-L
AA vs butyrate	50µM AA vs. 75µM	-107.2	No	ns	0.1599	B-M
AA vs butyrate	50µM AA vs. 100µM	-113.8	No	ns	0.0671	B-N
AA vs butyrate	50µM AA vs. 250µM	-41.83	No	ns	>0.9999	B-O
AA vs butyrate	50µM AA vs. 500µM	-96.33	No	ns	0.5932	B-P
AA vs butyrate	50µM AA vs. 750µM	-119.7	Yes	*	0.0302	B-Q
AA vs butyrate	50µM AA vs. 1000µM	-68.5	No	ns	>0.9999	B-R
AA vs butyrate	50µM AA vs. 2500µM	-53.83	No	ns	>0.9999	B-S

AA vs butyrate	50µM AA vs. 5000µM	-61.17	No	ns	>0.9999	B-T
AA vs butyrate	50µM AA vs. 7500µM	-57.67	No	ns	>0.9999	B-U
AA vs. Combo	50µM AA vs. 50pg/mL	-93.83	No	ns	0.789	B-V
AA vs. Combo	50µM AA vs. 100pg/mL	-25.33	No	ns	>0.9999	B-W
AA vs. Combo	50µM AA vs. 150pg/mL	-79.33	No	ns	>0.9999	B-X
AA vs. Combo	50µM AA vs. 200pg/mL	-106.8	No	ns	0.1668	B-Y
AA vs. Combo	50µM AA vs. 250pg/mL	-61.83	No	ns	>0.9999	B-Z
AA vs. Combo	50µM AA vs. 500pg/mL	-48.67	No	ns	>0.9999	B-AA
AA vs. Combo	50µM AA vs. 1000pg/mL	-28.67	No	ns	>0.9999	B-AB
AA vs. Combo	50µM AA vs. 2500pg/mL	-10.83	No	ns	>0.9999	B-AC
AA vs. Combo	50µM AA vs. 5000pg/mL	-6	No	ns	>0.9999	B-AD
TNFa vs. TNFa	50pg/mL vs. 100pg/mL	-15.67	No	ns	>0.9999	C-D
TNFa vs. TNFa	50pg/mL vs. 150pg/mL	-4.333	No	ns	>0.9999	C-E
TNFa vs. TNFa	50pg/mL vs. 200pg/mL	-18.83	No	ns	>0.9999	C-F
TNFa vs. TNFa	50pg/mL vs. 250pg/mL	47.67	No	ns	>0.9999	C-G
TNFa vs. TNFa	50pg/mL vs. 500pg/mL	6.5	No	ns	>0.9999	C-H
TNFa vs. TNFa	50pg/mL vs. 1000pg/mL	2.333	No	ns	>0.9999	C-I
TNFa vs. TNFa	50pg/mL vs. 2500pg/mL	34.83	No	ns	>0.9999	C-J
TNFa vs. TNFa	50pg/mL vs. 5000pg/mL	72.33	No	ns	>0.9999	C-K
TNFa vs. Butyrate	50pg/mL vs. 50µM	14.67	No	ns	>0.9999	C-L
TNFa vs. Butyrate	50pg/mL vs. 75µM	32	No	ns	>0.9999	C-M
TNFa vs. Butyrate	50pg/mL vs. 100µM	25.33	No	ns	>0.9999	C-N
TNFa vs. Butyrate	50pg/mL vs. 250µM	97.33	No	ns	0.5283	C-O
TNFa vs. Butyrate	50pg/mL vs. 500µM	42.83	No	ns	>0.9999	C-P
TNFa vs. Butyrate	50pg/mL vs. 750µM	19.5	No	ns	>0.9999	C-Q
TNFa vs. Butyrate	50pg/mL vs. 1000µM	70.67	No	ns	>0.9999	C-R
TNFa vs. Butyrate	50pg/mL vs. 2500µM	85.33	No	ns	>0.9999	C-S
TNFa vs. Butyrate	50pg/mL vs. 5000µM	78	No	ns	>0.9999	C-T
TNFa vs. Butyrate	50pg/mL vs. 7500µM	81.5	No	ns	>0.9999	C-U
TNFa vs. Combo	50pg/mL vs. 50pg/mL	45.33	No	ns	>0.9999	C-V
TNFa vs. Combo	50pg/mL vs. 100pg/mL	113.8	No	ns	0.0671	C-W
TNFa vs. Combo	50pg/mL vs. 150pg/mL	59.83	No	ns	>0.9999	C-X
TNFa vs. Combo	50pg/mL vs. 200pg/mL	32.33	No	ns	>0.9999	C-Y
TNFa vs. Combo	50pg/mL vs. 250pg/mL	77.33	No	ns	>0.9999	C-Z
TNFa vs. Combo	50pg/mL vs. 500pg/mL	90.5	No	ns	>0.9999	C-AA
TNFa vs. Combo	50pg/mL vs. 1000pg/mL	110.5	No	ns	0.1042	C-AB
TNFa vs. Combo	50pg/mL vs. 2500pg/mL	128.3	Yes	**	0.0087	C-AC
TNFa vs. Combo	50pg/mL vs. 5000pg/mL	133.2	Yes	**	0.0042	C-AD
TNFa vs. TNFa	100pg/mL vs. 150pg/mL	11.33	No	ns	>0.9999	D-E
TNFa vs. TNFa	100pg/mL vs. 200pg/mL	-3.167	No	ns	>0.9999	D-F
TNFa vs. TNFa	100pg/mL vs. 250pg/mL	63.33	No	ns	>0.9999	D-G
TNFa vs. TNFa	100pg/mL vs. 500pg/mL	22.17	No	ns	>0.9999	D-H
TNFa vs. TNFa	100pg/mL vs. 1000pg/mL	18	No	ns	>0.9999	D-I
TNFa vs. TNFa	100pg/mL vs. 2500pg/mL	50.5	No	ns	>0.9999	D-J
TNFa vs. TNFa	100pg/mL vs. 5000pg/mL	88	No	ns	>0.9999	D-K
TNFa vs. Butyrate	100pg/mL vs. 50µM	30.33	No	ns	>0.9999	D-L
TNFa vs. Butyrate	100pg/mL vs. 75µM	47.67	No	ns	>0.9999	D-M

TNFa vs. Butyrate	100pg/mL vs. 100µM	41	No	ns	>0.9999	D-N
TNFa vs. Butyrate	100pg/mL vs. 250µM	113	No	ns	0.075	D-O
TNFa vs. Butyrate	100pg/mL vs. 500µM	58.5	No	ns	>0.9999	D-P
TNFa vs. Butyrate	100pg/mL vs. 750µM	35.17	No	ns	>0.9999	D-Q
TNFa vs. Butyrate	100pg/mL vs. 1000µM	86.33	No	ns	>0.9999	D-R
TNFa vs. Butyrate	100pg/mL vs. 2500µM	101	No	ns	0.3423	D-S
TNFa vs. Butyrate	100pg/mL vs. 5000µM	93.67	No	ns	0.804	D-T
TNFa vs. Butyrate	100pg/mL vs. 7500µM	97.17	No	ns	0.5386	D-U
TNFa vs. Combo	100pg/mL vs. 50pg/mL	61	No	ns	>0.9999	D-V
TNFa vs. Combo	100pg/mL vs. 100pg/mL	129.5	Yes	**	0.0073	D-W
TNFa vs. Combo	100pg/mL vs. 150pg/mL	75.5	No	ns	>0.9999	D-X
TNFa vs. Combo	100pg/mL vs. 200pg/mL	48	No	ns	>0.9999	D-Y
TNFa vs. Combo	100pg/mL vs. 250pg/mL	93	No	ns	0.8665	D-Z
TNFa vs. Combo	100pg/mL vs. 500pg/mL	106.2	No	ns	0.1814	D-AA
TNFa vs. Combo	100pg/mL vs. 1000pg/mL	126.2	Yes	*	0.0119	D-AB
TNFa vs. Combo	100pg/mL vs. 2500pg/mL	144	Yes	***	0.0007	D-AC
TNFa vs. Combo	100pg/mL vs. 5000pg/mL	148.8	Yes	***	0.0003	D-AD
TNFa vs. TNFa	150pg/mL vs. 200pg/mL	-14.5	No	ns	>0.9999	E-F
TNFa vs. TNFa	150pg/mL vs. 250pg/mL	52	No	ns	>0.9999	E-G
TNFa vs. TNFa	150pg/mL vs. 500pg/mL	10.83	No	ns	>0.9999	E-H
TNFa vs. TNFa	150pg/mL vs. 1000pg/mL	6.667	No	ns	>0.9999	E-I
TNFa vs. TNFa	150pg/mL vs. 2500pg/mL	39.17	No	ns	>0.9999	E-J
TNFa vs. TNFa	150pg/mL vs. 5000pg/mL	76.67	No	ns	>0.9999	E-K
TNFa vs. Butyrate	150pg/mL vs. 50µM	19	No	ns	>0.9999	E-L
TNFa vs. Butyrate	150pg/mL vs. 75µM	36.33	No	ns	>0.9999	E-M
TNFa vs. Butyrate	150pg/mL vs. 100µM	29.67	No	ns	>0.9999	E-N
TNFa vs. Butyrate	150pg/mL vs. 250µM	101.7	No	ns	0.3159	E-O
TNFa vs. Butyrate	150pg/mL vs. 500µM	47.17	No	ns	>0.9999	E-P
TNFa vs. Butyrate	150pg/mL vs. 750µM	23.83	No	ns	>0.9999	E-Q
TNFa vs. Butyrate	150pg/mL vs. 1000µM	75	No	ns	>0.9999	E-R
TNFa vs. Butyrate	150pg/mL vs. 2500µM	89.67	No	ns	>0.9999	E-S
TNFa vs. Butyrate	150pg/mL vs. 5000µM	82.33	No	ns	>0.9999	E-T
TNFa vs. Butyrate	150pg/mL vs. 7500µM	85.83	No	ns	>0.9999	E-U
TNFa vs. Combo	150pg/mL vs. 50pg/mL	49.67	No	ns	>0.9999	E-V
TNFa vs. Combo	150pg/mL vs. 100pg/mL	118.2	Yes	*	0.0373	E-W
TNFa vs. Combo	150pg/mL vs. 150pg/mL	64.17	No	ns	>0.9999	E-X
TNFa vs. Combo	150pg/mL vs. 200pg/mL	36.67	No	ns	>0.9999	E-Y
TNFa vs. Combo	150pg/mL vs. 250pg/mL	81.67	No	ns	>0.9999	E-Z
TNFa vs. Combo	150pg/mL vs. 500pg/mL	94.83	No	ns	0.7045	E-AA
TNFa vs. Combo	150pg/mL vs. 1000pg/mL	114.8	No	ns	0.0587	E-AB
TNFa vs. Combo	150pg/mL vs. 2500pg/mL	132.7	Yes	**	0.0045	E-AC
TNFa vs. Combo	150pg/mL vs. 5000pg/mL	137.5	Yes	**	0.0021	E-AD
TNFa vs. TNFa	200pg/mL vs. 250pg/mL	66.5	No	ns	>0.9999	F-G
TNFa vs. TNFa	200pg/mL vs. 500pg/mL	25.33	No	ns	>0.9999	F-H
TNFa vs. TNFa	200pg/mL vs. 1000pg/mL	21.17	No	ns	>0.9999	F-I
TNFa vs. TNFa	200pg/mL vs. 2500pg/mL	53.67	No	ns	>0.9999	F-J
TNFa vs. TNFa	200pg/mL vs. 5000pg/mL	91.17	No	ns	>0.9999	F-K

TNFa vs. Butyrate	200pg/mL vs. 50µM	33.5	No	ns	>0.9999	F-L
TNFa vs. Butyrate	200pg/mL vs. 75µM	50.83	No	ns	>0.9999	F-M
TNFa vs. Butyrate	200pg/mL vs. 100µM	44.17	No	ns	>0.9999	F-N
TNFa vs. Butyrate	200pg/mL vs. 250µM	116.2	Yes	*	0.049	F-O
TNFa vs. Butyrate	200pg/mL vs. 500µM	61.67	No	ns	>0.9999	F-P
TNFa vs. Butyrate	200pg/mL vs. 750µM	38.33	No	ns	>0.9999	F-Q
TNFa vs. Butyrate	200pg/mL vs. 1000µM	89.5	No	ns	>0.9999	F-R
TNFa vs. Butyrate	200pg/mL vs. 2500µM	104.2	No	ns	0.2327	F-S
TNFa vs. Butyrate	200pg/mL vs. 5000µM	96.83	No	ns	0.5599	F-T
TNFa vs. Butyrate	200pg/mL vs. 7500µM	100.3	No	ns	0.3708	F-U
TNFa vs. Combo	200pg/mL vs. 50pg/mL	64.17	No	ns	>0.9999	F-V
TNFa vs. Combo	200pg/mL vs. 100pg/mL	132.7	Yes	**	0.0045	F-W
TNFa vs. Combo	200pg/mL vs. 150pg/mL	78.67	No	ns	>0.9999	F-X
TNFa vs. Combo	200pg/mL vs. 200pg/mL	51.17	No	ns	>0.9999	F-Y
TNFa vs. Combo	200pg/mL vs. 250pg/mL	96.17	No	ns	0.6047	F-Z
TNFa vs. Combo	200pg/mL vs. 500pg/mL	109.3	No	ns	0.1212	F-AA
TNFa vs. Combo	200pg/mL vs. 1000pg/mL	129.3	Yes	**	0.0075	F-AB
TNFa vs. Combo	200pg/mL vs. 2500pg/mL	147.2	Yes	***	0.0004	F-AC
TNFa vs. Combo	200pg/mL vs. 5000pg/mL	152	Yes	***	0.0002	F-AD
TNFa vs. TNFa	250pg/mL vs. 500pg/mL	-41.17	No	ns	>0.9999	G-H
TNFa vs. TNFa	250pg/mL vs. 1000pg/mL	-45.33	No	ns	>0.9999	G-I
TNFa vs. TNFa	250pg/mL vs. 2500pg/mL	-12.83	No	ns	>0.9999	G-J
TNFa vs. TNFa	250pg/mL vs. 5000pg/mL	24.67	No	ns	>0.9999	G-K
TNFa vs. Butyrate	250pg/mL vs. 50µM	-33	No	ns	>0.9999	G-L
TNFa vs. Butyrate	250pg/mL vs. 75µM	-15.67	No	ns	>0.9999	G-M
TNFa vs. Butyrate	250pg/mL vs. 100µM	-22.33	No	ns	>0.9999	G-N
TNFa vs. Butyrate	250pg/mL vs. 250µM	49.67	No	ns	>0.9999	G-O
TNFa vs. Butyrate	250pg/mL vs. 500µM	-4.833	No	ns	>0.9999	G-P
TNFa vs. Butyrate	250pg/mL vs. 750µM	-28.17	No	ns	>0.9999	G-Q
TNFa vs. Butyrate	250pg/mL vs. 1000µM	23	No	ns	>0.9999	G-R
TNFa vs. Butyrate	250pg/mL vs. 2500µM	37.67	No	ns	>0.9999	G-S
TNFa vs. Butyrate	250pg/mL vs. 5000µM	30.33	No	ns	>0.9999	G-T
TNFa vs. Butyrate	250pg/mL vs. 7500µM	33.83	No	ns	>0.9999	G-U
TNFa vs. Combo	250pg/mL vs. 50pg/mL	-2.333	No	ns	>0.9999	G-V
TNFa vs. Combo	250pg/mL vs. 100pg/mL	66.17	No	ns	>0.9999	G-W
TNFa vs. Combo	250pg/mL vs. 150pg/mL	12.17	No	ns	>0.9999	G-X
TNFa vs. Combo	250pg/mL vs. 200pg/mL	-15.33	No	ns	>0.9999	G-Y
TNFa vs. Combo	250pg/mL vs. 250pg/mL	29.67	No	ns	>0.9999	G-Z
TNFa vs. Combo	250pg/mL vs. 500pg/mL	42.83	No	ns	>0.9999	G-AA
TNFa vs. Combo	250pg/mL vs. 1000pg/mL	62.83	No	ns	>0.9999	G-AB
TNFa vs. Combo	250pg/mL vs. 2500pg/mL	80.67	No	ns	>0.9999	G-AC
TNFa vs. Combo	250pg/mL vs. 5000pg/mL	85.5	No	ns	>0.9999	G-AD
TNFa vs. TNFa	500pg/mL vs. 1000pg/mL	-4.167	No	ns	>0.9999	H-I
TNFa vs. TNFa	500pg/mL vs. 2500pg/mL	28.33	No	ns	>0.9999	H-J
TNFa vs. TNFa	500pg/mL vs. 5000pg/mL	65.83	No	ns	>0.9999	H-K
TNFa vs. Butyrate	500pg/mL vs. 50µM	8.167	No	ns	>0.9999	H-L
TNFa vs. Butyrate	500pg/mL vs. 75µM	25.5	No	ns	>0.9999	H-M

TNFa vs. Butyrate	500pg/mL vs. 100µM	18.83	No	ns	>0.9999	H-N
TNFa vs. Butyrate	500pg/mL vs. 250µM	90.83	No	ns	>0.9999	H-O
TNFa vs. Butyrate	500pg/mL vs. 500µM	36.33	No	ns	>0.9999	H-P
TNFa vs. Butyrate	500pg/mL vs. 750µM	13	No	ns	>0.9999	H-Q
TNFa vs. Butyrate	500pg/mL vs. 1000µM	64.17	No	ns	>0.9999	H-R
TNFa vs. Butyrate	500pg/mL vs. 2500µM	78.83	No	ns	>0.9999	H-S
TNFa vs. Butyrate	500pg/mL vs. 5000µM	71.5	No	ns	>0.9999	H-T
TNFa vs. Butyrate	500pg/mL vs. 7500µM	75	No	ns	>0.9999	H-U
TNFa vs. Combo	500pg/mL vs. 50pg/mL	38.83	No	ns	>0.9999	H-V
TNFa vs. Combo	500pg/mL vs. 100pg/mL	107.3	No	ns	0.1565	H-W
TNFa vs. Combo	500pg/mL vs. 150pg/mL	53.33	No	ns	>0.9999	H-X
TNFa vs. Combo	500pg/mL vs. 200pg/mL	25.83	No	ns	>0.9999	H-Y
TNFa vs. Combo	500pg/mL vs. 250pg/mL	70.83	No	ns	>0.9999	H-Z
TNFa vs. Combo	500pg/mL vs. 500pg/mL	84	No	ns	>0.9999	H-AA
TNFa vs. Combo	500pg/mL vs. 1000pg/mL	104	No	ns	0.2375	H-AB
TNFa vs. Combo	500pg/mL vs. 2500pg/mL	121.8	Yes	*	0.0223	H-AC
TNFa vs. Combo	500pg/mL vs. 5000pg/mL	126.7	Yes	*	0.0111	H-AD
TNFa vs. TNFa	1000pg/mL vs. 2500pg/mL	32.5	No	ns	>0.9999	I-J
TNFa vs. TNFa	1000pg/mL vs. 5000pg/mL	70	No	ns	>0.9999	I-K
TNFa vs. Butyrate	1000pg/mL vs. 50µM	12.33	No	ns	>0.9999	I-L
TNFa vs. Butyrate	1000pg/mL vs. 75µM	29.67	No	ns	>0.9999	I-M
TNFa vs. Butyrate	1000pg/mL vs. 100µM	23	No	ns	>0.9999	I-N
TNFa vs. Butyrate	1000pg/mL vs. 250µM	95	No	ns	0.6912	I-O
TNFa vs. Butyrate	1000pg/mL vs. 500µM	40.5	No	ns	>0.9999	I-P
TNFa vs. Butyrate	1000pg/mL vs. 750µM	17.17	No	ns	>0.9999	I-Q
TNFa vs. Butyrate	1000pg/mL vs. 1000µM	68.33	No	ns	>0.9999	I-R
TNFa vs. Butyrate	1000pg/mL vs. 2500µM	83	No	ns	>0.9999	I-S
TNFa vs. Butyrate	1000pg/mL vs. 5000µM	75.67	No	ns	>0.9999	I-T
TNFa vs. Butyrate	1000pg/mL vs. 7500µM	79.17	No	ns	>0.9999	I-U
TNFa vs. Combo	1000pg/mL vs. 50pg/mL	43	No	ns	>0.9999	I-V
TNFa vs. Combo	1000pg/mL vs. 100pg/mL	111.5	No	ns	0.0915	I-W
TNFa vs. Combo	1000pg/mL vs. 150pg/mL	57.5	No	ns	>0.9999	I-X
TNFa vs. Combo	1000pg/mL vs. 200pg/mL	30	No	ns	>0.9999	I-Y
TNFa vs. Combo	1000pg/mL vs. 250pg/mL	75	No	ns	>0.9999	I-Z
TNFa vs. Combo	1000pg/mL vs. 500pg/mL	88.17	No	ns	>0.9999	I-AA
TNFa vs. Combo	1000pg/mL vs. 1000pg/mL	108.2	No	ns	0.1408	I-AB
TNFa vs. Combo	1000pg/mL vs. 2500pg/mL	126	Yes	*	0.0122	I-AC
TNFa vs. Combo	1000pg/mL vs. 5000pg/mL	130.8	Yes	**	0.0059	I-AD
TNFa vs. TNFa	2500pg/mL vs. 5000pg/mL	37.5	No	ns	>0.9999	J-K
TNFa vs. Butyrate	2500pg/mL vs. 50µM	-20.17	No	ns	>0.9999	J-L
TNFa vs. Butyrate	2500pg/mL vs. 75µM	-2.833	No	ns	>0.9999	J-M
TNFa vs. Butyrate	2500pg/mL vs. 100µM	-9.5	No	ns	>0.9999	J-N
TNFa vs. Butyrate	2500pg/mL vs. 250µM	62.5	No	ns	>0.9999	J-O
TNFa vs. Butyrate	2500pg/mL vs. 500µM	8	No	ns	>0.9999	J-P
TNFa vs. Butyrate	2500pg/mL vs. 750µM	-15.33	No	ns	>0.9999	J-Q
TNFa vs. Butyrate	2500pg/mL vs. 1000µM	35.83	No	ns	>0.9999	J-R
TNFa vs. Butyrate	2500pg/mL vs. 2500µM	50.5	No	ns	>0.9999	J-S

TNFa vs. Butyrate	2500pg/mL vs. 5000µM	43.17	No	ns	>0.9999	J-T
TNFa vs. Butyrate	2500pg/mL vs. 7500µM	46.67	No	ns	>0.9999	J-U
TNFa vs. Combo	2500pg/mL vs. 50pg/mL	10.5	No	ns	>0.9999	J-V
TNFa vs. Combo	2500pg/mL vs. 100pg/mL	79	No	ns	>0.9999	J-W
TNFa vs. Combo	2500pg/mL vs. 150pg/mL	25	No	ns	>0.9999	J-X
TNFa vs. Combo	2500pg/mL vs. 200pg/mL	-2.5	No	ns	>0.9999	J-Y
TNFa vs. Combo	2500pg/mL vs. 250pg/mL	42.5	No	ns	>0.9999	J-Z
TNFa vs. Combo	2500pg/mL vs. 500pg/mL	55.67	No	ns	>0.9999	J-AA
TNFa vs. Combo	2500pg/mL vs. 1000pg/mL	75.67	No	ns	>0.9999	J-AB
TNFa vs. Combo	2500pg/mL vs. 2500pg/mL	93.5	No	ns	0.8192	J-AC
TNFa vs. Combo	2500pg/mL vs. 5000pg/mL	98.33	No	ns	0.47	J-AD
TNFa vs. Butyrate	5000pg/mL vs. 50µM	-57.67	No	ns	>0.9999	K-L
TNFa vs. Butyrate	5000pg/mL vs. 75µM	-40.33	No	ns	>0.9999	K-M
TNFa vs. Butyrate	5000pg/mL vs. 100µM	-47	No	ns	>0.9999	K-N
TNFa vs. Butyrate	5000pg/mL vs. 250µM	25	No	ns	>0.9999	K-O
TNFa vs. Butyrate	5000pg/mL vs. 500µM	-29.5	No	ns	>0.9999	K-P
TNFa vs. Butyrate	5000pg/mL vs. 750µM	-52.83	No	ns	>0.9999	K-Q
TNFa vs. Butyrate	5000pg/mL vs. 1000µM	-1.667	No	ns	>0.9999	K-R
TNFa vs. Butyrate	5000pg/mL vs. 2500µM	13	No	ns	>0.9999	K-S
TNFa vs. Butyrate	5000pg/mL vs. 5000µM	5.667	No	ns	>0.9999	K-T
TNFa vs. Butyrate	5000pg/mL vs. 7500µM	9.167	No	ns	>0.9999	K-U
TNFa vs. Combo	5000pg/mL vs. 50pg/mL	-27	No	ns	>0.9999	K-V
TNFa vs. Combo	5000pg/mL vs. 100pg/mL	41.5	No	ns	>0.9999	K-W
TNFa vs. Combo	5000pg/mL vs. 150pg/mL	-12.5	No	ns	>0.9999	K-X
TNFa vs. Combo	5000pg/mL vs. 200pg/mL	-40	No	ns	>0.9999	K-Y
TNFa vs. Combo	5000pg/mL vs. 250pg/mL	5	No	ns	>0.9999	K-Z
TNFa vs. Combo	5000pg/mL vs. 500pg/mL	18.17	No	ns	>0.9999	K-AA
TNFa vs. Combo	5000pg/mL vs. 1000pg/mL	38.17	No	ns	>0.9999	K-AB
TNFa vs. Combo	5000pg/mL vs. 2500pg/mL	56	No	ns	>0.9999	K-AC
TNFa vs. Combo	5000pg/mL vs. 5000pg/mL	60.83	No	ns	>0.9999	K-AD
Butyrate vs. Butyrate	50µM vs. 75µM	17.33	No	ns	>0.9999	L-M
Butyrate vs. Butyrate	50µM vs. 100µM	10.67	No	ns	>0.9999	L-N
Butyrate vs. Butyrate	50µM vs. 250µM	82.67	No	ns	>0.9999	L-O
Butyrate vs. Butyrate	50µM vs. 500µM	28.17	No	ns	>0.9999	L-P
Butyrate vs. Butyrate	50µM vs. 750µM	4.833	No	ns	>0.9999	L-Q
Butyrate vs. Butyrate	50µM vs. 1000µM	56	No	ns	>0.9999	L-R
Butyrate vs. Butyrate	50µM vs. 2500µM	70.67	No	ns	>0.9999	L-S
Butyrate vs. Butyrate	50µM vs. 5000µM	63.33	No	ns	>0.9999	L-T
Butyrate vs. Butyrate	50µM vs. 7500µM	66.83	No	ns	>0.9999	L-U
Butyrate vs. Combo	50µM vs. 50pg/mL	30.67	No	ns	>0.9999	L-V
Butyrate vs. Combo	50µM vs. 100pg/mL	99.17	No	ns	0.426	L-W
Butyrate vs. Combo	50µM vs. 150pg/mL	45.17	No	ns	>0.9999	L-X
Butyrate vs. Combo	50µM vs. 200pg/mL	17.67	No	ns	>0.9999	L-Y
Butyrate vs. Combo	50µM vs. 250pg/mL	62.67	No	ns	>0.9999	L-Z
Butyrate vs. Combo	50µM vs. 500pg/mL	75.83	No	ns	>0.9999	L-AA
Butyrate vs. Combo	50µM vs. 1000pg/mL	95.83	No	ns	0.6284	L-AB
Butyrate vs. Combo	50µM vs. 2500pg/mL	113.7	No	ns	0.0687	L-AC

Butyrate vs. Combo	50µM vs. 5000pg/mL	118.5	Yes	*	0.0356	L-AD
Butyrate vs. Butyrate	75µM vs. 100µM	-6.667	No	ns	>0.9999	M-N
Butyrate vs. Butyrate	75µM vs. 250µM	65.33	No	ns	>0.9999	M-O
Butyrate vs. Butyrate	75µM vs. 500µM	10.83	No	ns	>0.9999	M-P
Butyrate vs. Butyrate	75µM vs. 750µM	-12.5	No	ns	>0.9999	M-Q
Butyrate vs. Butyrate	75µM vs. 1000µM	38.67	No	ns	>0.9999	M-R
Butyrate vs. Butyrate	75µM vs. 2500µM	53.33	No	ns	>0.9999	M-S
Butyrate vs. Butyrate	75µM vs. 5000µM	46	No	ns	>0.9999	M-T
Butyrate vs. Butyrate	75µM vs. 7500µM	49.5	No	ns	>0.9999	M-U
Butyrate vs. Combo	75µM vs. 50pg/mL	13.33	No	ns	>0.9999	M-V
Butyrate vs. Combo	75µM vs. 100pg/mL	81.83	No	ns	>0.9999	M-W
Butyrate vs. Combo	75µM vs. 150pg/mL	27.83	No	ns	>0.9999	M-X
Butyrate vs. Combo	75µM vs. 200pg/mL	0.3333	No	ns	>0.9999	M-Y
Butyrate vs. Combo	75µM vs. 250pg/mL	45.33	No	ns	>0.9999	M-Z
Butyrate vs. Combo	75µM vs. 500pg/mL	58.5	No	ns	>0.9999	M-AA
Butyrate vs. Combo	75µM vs. 1000pg/mL	78.5	No	ns	>0.9999	M-AB
Butyrate vs. Combo	75µM vs. 2500pg/mL	96.33	No	ns	0.5932	M-AC
Butyrate vs. Combo	75µM vs. 5000pg/mL	101.2	No	ns	0.3355	M-AD
Butyrate vs. Combo	100µM vs. 250µM	72	No	ns	>0.9999	N-O
Butyrate vs. Combo	100µM vs. 500µM	17.5	No	ns	>0.9999	N-P
Butyrate vs. Butyrate	100µM vs. 750µM	-5.833	No	ns	>0.9999	N-Q
Butyrate vs. Butyrate	100µM vs. 1000µM	45.33	No	ns	>0.9999	N-R
Butyrate vs. Butyrate	100µM vs. 2500µM	60	No	ns	>0.9999	N-S
Butyrate vs. Butyrate	100µM vs. 5000µM	52.67	No	ns	>0.9999	N-T
Butyrate vs. Butyrate	100µM vs. 7500µM	56.17	No	ns	>0.9999	N-U
Butyrate vs. Combo	100µM vs. 50pg/mL	20	No	ns	>0.9999	N-V
Butyrate vs. Combo	100µM vs. 100pg/mL	88.5	No	ns	>0.9999	N-W
Butyrate vs. Combo	100µM vs. 150pg/mL	34.5	No	ns	>0.9999	N-X
Butyrate vs. Combo	100µM vs. 200pg/mL	7	No	ns	>0.9999	N-Y
Butyrate vs. Combo	100µM vs. 250pg/mL	52	No	ns	>0.9999	N-Z
Butyrate vs. Combo	100µM vs. 500pg/mL	65.17	No	ns	>0.9999	N-AA
Butyrate vs. Combo	100µM vs. 1000pg/mL	85.17	No	ns	>0.9999	N-AB
Butyrate vs. Combo	100µM vs. 2500pg/mL	103	No	ns	0.2686	N-AC
Butyrate vs. Combo	100µM vs. 5000pg/mL	107.8	No	ns	0.1469	N-AD
Butyrate vs. Butyrate	250µM vs. 500µM	-54.5	No	ns	>0.9999	O-P
Butyrate vs. Butyrate	250µM vs. 750µM	-77.83	No	ns	>0.9999	O-Q
Butyrate vs. Butyrate	250µM vs. 1000µM	-26.67	No	ns	>0.9999	O-R
Butyrate vs. Butyrate	250µM vs. 2500µM	-12	No	ns	>0.9999	O-S
Butyrate vs. Butyrate	250µM vs. 5000µM	-19.33	No	ns	>0.9999	O-T
Butyrate vs. Butyrate	250µM vs. 7500µM	-15.83	No	ns	>0.9999	O-U
Butyrate vs. Combo	250µM vs. 50pg/mL	-52	No	ns	>0.9999	O-V
Butyrate vs. Combo	250µM vs. 100pg/mL	16.5	No	ns	>0.9999	O-W
Butyrate vs. Combo	250µM vs. 150pg/mL	-37.5	No	ns	>0.9999	O-X
Butyrate vs. Combo	250µM vs. 200pg/mL	-65	No	ns	>0.9999	O-Y
Butyrate vs. Combo	250µM vs. 250pg/mL	-20	No	ns	>0.9999	O-Z
Butyrate vs. Combo	250µM vs. 500pg/mL	-6.833	No	ns	>0.9999	O-AA
Butyrate vs. Combo	250µM vs. 1000pg/mL	13.17	No	ns	>0.9999	O-AB

Butyrate vs. Combo	250µM vs. 2500pg/mL	31	No	ns	>0.9999	O-AC
Butyrate vs. Combo	250µM vs. 5000pg/mL	35.83	No	ns	>0.9999	O-AD
Butyrate vs. Butyrate	500µM vs. 750µM	-23.33	No	ns	>0.9999	P-Q
Butyrate vs. Butyrate	500µM vs. 1000µM	27.83	No	ns	>0.9999	P-R
Butyrate vs. Butyrate	500µM vs. 2500µM	42.5	No	ns	>0.9999	P-S
Butyrate vs. Butyrate	500µM vs. 5000µM	35.17	No	ns	>0.9999	P-T
Butyrate vs. Butyrate	500µM vs. 7500µM	38.67	No	ns	>0.9999	P-U
Butyrate vs. Combo	500µM vs. 50pg/mL	2.5	No	ns	>0.9999	P-V
Butyrate vs. Combo	500µM vs. 100pg/mL	71	No	ns	>0.9999	P-W
Butyrate vs. Combo	500µM vs. 150pg/mL	17	No	ns	>0.9999	P-X
Butyrate vs. Combo	500µM vs. 200pg/mL	-10.5	No	ns	>0.9999	P-Y
Butyrate vs. Combo	500µM vs. 250pg/mL	34.5	No	ns	>0.9999	P-Z
Butyrate vs. Combo	500µM vs. 500pg/mL	47.67	No	ns	>0.9999	P-AA
Butyrate vs. Combo	500µM vs. 1000pg/mL	67.67	No	ns	>0.9999	P-AB
Butyrate vs. Combo	500µM vs. 2500pg/mL	85.5	No	ns	>0.9999	P-AC
Butyrate vs. Combo	500µM vs. 5000pg/mL	90.33	No	ns	>0.9999	P-AD
Butyrate vs. Butyrate	750µM vs. 1000µM	51.17	No	ns	>0.9999	Q-R
Butyrate vs. Butyrate	750µM vs. 2500µM	65.83	No	ns	>0.9999	Q-S
Butyrate vs. Butyrate	750µM vs. 5000µM	58.5	No	ns	>0.9999	Q-T
Butyrate vs. Butyrate	750µM vs. 7500µM	62	No	ns	>0.9999	Q-U
Butyrate vs. Combo	750µM vs. 50pg/mL	25.83	No	ns	>0.9999	Q-V
Butyrate vs. Combo	750µM vs. 100pg/mL	94.33	No	ns	0.7457	Q-W
Butyrate vs. Combo	750µM vs. 150pg/mL	40.33	No	ns	>0.9999	Q-X
Butyrate vs. Combo	750µM vs. 200pg/mL	12.83	No	ns	>0.9999	Q-Y
Butyrate vs. Combo	750µM vs. 250pg/mL	57.83	No	ns	>0.9999	Q-Z
Butyrate vs. Combo	750µM vs. 500pg/mL	71	No	ns	>0.9999	Q-AA
Butyrate vs. Combo	750µM vs. 1000pg/mL	91	No	ns	>0.9999	Q-AB
Butyrate vs. Combo	750µM vs. 2500pg/mL	108.8	No	ns	0.1293	Q-AC
Butyrate vs. Combo	750µM vs. 5000pg/mL	113.7	No	ns	0.0687	Q-AD
Butyrate vs. Butyrate	1000µM vs. 2500µM	14.67	No	ns	>0.9999	R-S
Butyrate vs. Butyrate	1000µM vs. 5000µM	7.333	No	ns	>0.9999	R-T
Butyrate vs. Butyrate	1000µM vs. 7500µM	10.83	No	ns	>0.9999	R-U
Butyrate vs. Combo	1000µM vs. 50pg/mL	-25.33	No	ns	>0.9999	R-V
Butyrate vs. Combo	1000µM vs. 100pg/mL	43.17	No	ns	>0.9999	R-W
Butyrate vs. Combo	1000µM vs. 150pg/mL	-10.83	No	ns	>0.9999	R-X
Butyrate vs. Combo	1000µM vs. 200pg/mL	-38.33	No	ns	>0.9999	R-Y
Butyrate vs. Combo	1000µM vs. 250pg/mL	6.667	No	ns	>0.9999	R-Z
Butyrate vs. Combo	1000µM vs. 500pg/mL	19.83	No	ns	>0.9999	R-AA
Butyrate vs. Combo	1000µM vs. 1000pg/mL	39.83	No	ns	>0.9999	R-AB
Butyrate vs. Combo	1000µM vs. 2500pg/mL	57.67	No	ns	>0.9999	R-AC
Butyrate vs. Combo	1000µM vs. 5000pg/mL	62.5	No	ns	>0.9999	R-AD
Butyrate vs. Butyrate	2500µM vs. 5000µM	-7.333	No	ns	>0.9999	S-T
Butyrate vs. Butyrate	2500µM vs. 7500µM	-3.833	No	ns	>0.9999	S-U
Butyrate vs. Combo	2500µM vs. 50pg/mL	-40	No	ns	>0.9999	S-V
Butyrate vs. Combo	2500µM vs. 100pg/mL	28.5	No	ns	>0.9999	S-W
Butyrate vs. Combo	2500µM vs. 150pg/mL	-25.5	No	ns	>0.9999	S-X
Butyrate vs. Combo	2500µM vs. 200pg/mL	-53	No	ns	>0.9999	S-Y

Butyrate vs. Combo	2500µM vs. 250pg/mL	-8	No	ns	>0.9999	S-Z
Butyrate vs. Combo	2500µM vs. 500pg/mL	5.167	No	ns	>0.9999	S-AA
Butyrate vs. Combo	2500µM vs. 1000pg/mL	25.17	No	ns	>0.9999	S-AB
Butyrate vs. Combo	2500µM vs. 2500pg/mL	43	No	ns	>0.9999	S-AC
Butyrate vs. Combo	2500µM vs. 5000pg/mL	47.83	No	ns	>0.9999	S-AD
Butyrate vs. Butyrate	5000µM vs. 7500µM	3.5	No	ns	>0.9999	T-U
Butyrate vs. Combo	5000µM vs. 50pg/mL	-32.67	No	ns	>0.9999	T-V
Butyrate vs. Combo	5000µM vs. 100pg/mL	35.83	No	ns	>0.9999	T-W
Butyrate vs. Combo	5000µM vs. 150pg/mL	-18.17	No	ns	>0.9999	T-X
Butyrate vs. Combo	5000µM vs. 200pg/mL	-45.67	No	ns	>0.9999	T-Y
Butyrate vs. Combo	5000µM vs. 250pg/mL	-0.6667	No	ns	>0.9999	T-Z
Butyrate vs. Combo	5000µM vs. 500pg/mL	12.5	No	ns	>0.9999	T-AA
Butyrate vs. Combo	5000µM vs. 1000pg/mL	32.5	No	ns	>0.9999	T-AB
Butyrate vs. Combo	5000µM vs. 2500pg/mL	50.33	No	ns	>0.9999	T-AC
Butyrate vs. Combo	5000µM vs. 5000pg/mL	55.17	No	ns	>0.9999	T-AD
Butyrate vs. Combo	7500µM vs. 50pg/mL	-36.17	No	ns	>0.9999	U-V
Butyrate vs. Combo	7500µM vs. 100pg/mL	32.33	No	ns	>0.9999	U-W
Butyrate vs. Combo	7500µM vs. 150pg/mL	-21.67	No	ns	>0.9999	U-X
Butyrate vs. Combo	7500µM vs. 200pg/mL	-49.17	No	ns	>0.9999	U-Y
Butyrate vs. Combo	7500µM vs. 250pg/mL	-4.167	No	ns	>0.9999	U-Z
Butyrate vs. Combo	7500µM vs. 500pg/mL	9	No	ns	>0.9999	U-AA
Butyrate vs. Combo	7500µM vs. 1000pg/mL	29	No	ns	>0.9999	U-AB
Butyrate vs. Combo	7500µM vs. 2500pg/mL	46.83	No	ns	>0.9999	U-AC
Butyrate vs. Combo	7500µM vs. 5000pg/mL	51.67	No	ns	>0.9999	U-AD
Butyrate vs. Combo	50pg/mL vs. 100pg/mL	68.5	No	ns	>0.9999	V-W
Combo vs. Combo	50pg/mL vs. 150pg/mL	14.5	No	ns	>0.9999	V-X
Combo vs. Combo	50pg/mL vs. 200pg/mL	-13	No	ns	>0.9999	V-Y
Combo vs. Combo	50pg/mL vs. 250pg/mL	32	No	ns	>0.9999	V-Z
Combo vs. Combo	50pg/mL vs. 500pg/mL	45.17	No	ns	>0.9999	V-AA
Combo vs. Combo	50pg/mL vs. 1000pg/mL	65.17	No	ns	>0.9999	V-AB
Combo vs. Combo	50pg/mL vs. 2500pg/mL	83	No	ns	>0.9999	V-AC
Combo vs. Combo	50pg/mL vs. 5000pg/mL	87.83	No	ns	>0.9999	V-AD
Combo vs. Combo	100pg/mL vs. 150pg/mL	-54	No	ns	>0.9999	W-X
Combo vs. Combo	100pg/mL vs. 200pg/mL	-81.5	No	ns	>0.9999	W-Y
Combo vs. Combo	100pg/mL vs. 250pg/mL	-36.5	No	ns	>0.9999	W-Z
Combo vs. Combo	100pg/mL vs. 500pg/mL	-23.33	No	ns	>0.9999	W-AA
Combo vs. Combo	100pg/mL vs. 1000pg/mL	-3.333	No	ns	>0.9999	W-AB
Combo vs. Combo	100pg/mL vs. 2500pg/mL	14.5	No	ns	>0.9999	W-AC
Combo vs. Combo	100pg/mL vs. 5000pg/mL	19.33	No	ns	>0.9999	W-AD
Combo vs. Combo	150pg/mL vs. 200pg/mL	-27.5	No	ns	>0.9999	X-Y
Combo vs. Combo	150pg/mL vs. 250pg/mL	17.5	No	ns	>0.9999	X-Z
Combo vs. Combo	150pg/mL vs. 500pg/mL	30.67	No	ns	>0.9999	X-AA
Combo vs. Combo	150pg/mL vs. 1000pg/mL	50.67	No	ns	>0.9999	X-AB
Combo vs. Combo	150pg/mL vs. 2500pg/mL	68.5	No	ns	>0.9999	X-AC
Combo vs. Combo	150pg/mL vs. 5000pg/mL	73.33	No	ns	>0.9999	X-AD
Combo vs. Combo	200pg/mL vs. 250pg/mL	45	No	ns	>0.9999	Y-Z
Combo vs. Combo	200pg/mL vs. 500pg/mL	58.17	No	ns	>0.9999	Y-AA

Combo vs. Combo	200pg/mL vs. 1000pg/mL	78.17	No	ns	>0.9999	Y-AB
Combo vs. Combo	200pg/mL vs. 2500pg/mL	96	No	ns	0.6164	Y-AC
Combo vs. Combo	200pg/mL vs. 5000pg/mL	100.8	No	ns	0.3492	Y-AD
Combo vs. Combo	250pg/mL vs. 500pg/mL	13.17	No	ns	>0.9999	Z-AA
Combo vs. Combo	250pg/mL vs. 1000pg/mL	33.17	No	ns	>0.9999	Z-AB
Combo vs. Combo	250pg/mL vs. 2500pg/mL	51	No	ns	>0.9999	Z-AC
Combo vs. Combo	250pg/mL vs. 5000pg/mL	55.83	No	ns	>0.9999	Z-AD
Combo vs. Combo	500pg/mL vs. 1000pg/mL	20	No	ns	>0.9999	AA-AB
Combo vs. Combo	500pg/mL vs. 2500pg/mL	37.83	No	ns	>0.9999	AA-AC
Combo vs. Combo	500pg/mL vs. 5000pg/mL	42.67	No	ns	>0.9999	AA-AD
Combo vs. Combo	1000pg/mL vs. 2500pg/mL	17.83	No	ns	>0.9999	AB-AC
Combo vs. Combo	1000pg/mL vs. 5000pg/mL	22.67	No	ns	>0.9999	AB-AD

Treatment	Dunn's multiple comparisons test	Mean rank	Significant’	Summary	
Media vs. Lysis	Media vs. Lysis	-70.83	No	ns	A-B
Media vs. TNFa	Media vs. 50pg/mL	-15.17	No	ns	A-C
Media vs. TNFa	Media vs. 100pg/mL	-23.17	No	ns	A-D
Media vs. TNFa	Media vs. 150pg/mL	-28.83	No	ns	A-E
Media vs. TNFa	Media vs. 200pg/mL	-35.83	No	ns	A-F
Media vs. TNFa	Media vs. 250pg/mL	-40.5	No	ns	A-G
Media vs. TNFa	Media vs. 500pg/mL	-55.83	No	ns	A-H
Media vs. TNFa	Media vs. 1ng/mL	-58.83	No	ns	A-I
Media vs. TNFa	Media vs. 2.5ng/mL	-62.17	No	ns	A-J
Media vs. TNFa	Media vs. 5ng/mL	-66.17	No	ns	A-K
Media vs. TNFa	Media vs. 10ng/mL	-66.17	No	ns	A-L
Media vs. Butyrate	Media vs. 50μM	1.167	No	ns	A-M
Media vs. Butyrate	Media vs. 75μM	6.5	No	ns	A-N
Media vs. Butyrate	Media vs. 100μM	13.83	No	ns	A-O
Media vs. Butyrate	Media vs. 250μM	15.17	No	ns	A-P
Media vs. Butyrate	Media vs. 500μM	6.833	No	ns	A-Q
Media vs. Butyrate	Media vs. 750μM	1.5	No	ns	A-R
Media vs. Butyrate	Media vs. 1mM	-8.167	No	ns	A-S
Media vs. Butyrate	Media vs. 2.5mM	-25.5	No	ns	A-T
Media vs. Butyrate	Media vs. 5mM	-47.83	No	ns	A-U
Media vs. Butyrate	Media vs. 7.5mM	-45.5	No	ns	A-V
Media vs. Butyrate	Media vs. 10mM	-51.83	No	ns	A-W
Media vs. Butyrate	Media vs. 25mM	-38.17	No	ns	A-X
Media vs. Combo	Media vs. 50pg/mL	-10.17	No	ns	A-Y
Media vs. Combo	Media vs. 150pg/mL	-15.5	No	ns	A-Z
Media vs. Combo	Media vs. 250pg/mL	-19.83	No	ns	A-AA
Media vs. Combo	Media vs. 500pg/mL	-32.17	No	ns	A-AB
Media vs. Combo	Media vs. 1ng/mL	-46.83	No	ns	A-AC
Lysis vs. TNFa	Lysis vs. 50pg/mL	55.67	No	ns	B-C
Lysis vs. TNFa	Lysis vs. 100pg/mL	47.67	No	ns	B-D
Lysis vs. TNFa	Lysis vs. 150pg/mL	42	No	ns	B-E
Lysis vs. TNFa	Lysis vs. 200pg/mL	35	No	ns	B-F
Lysis vs. TNFa	Lysis vs. 250pg/mL	30.33	No	ns	B-G
Lysis vs. TNFa	Lysis vs. 500pg/mL	15	No	ns	B-H
Lysis vs. TNFa	Lysis vs. 1ng/mL	12	No	ns	B-I
Lysis vs. TNFa	Lysis vs. 2.5ng/mL	8.667	No	ns	B-J
Lysis vs. TNFa	Lysis vs. 5ng/mL	4.667	No	ns	B-K
Lysis vs. TNFa	Lysis vs. 10ng/mL	4.667	No	ns	B-L
Lysis vs. Butyrate	Lysis vs. 50μM	72	No	ns	B-M
Lysis vs. Butyrate	Lysis vs. 75μM	77.33	No	ns	B-N
Lysis vs. Butyrate	Lysis vs. 100μM	84.67	Yes	*	B-O
Lysis vs. Butyrate	Lysis vs. 250μM	86	Yes	*	B-P
Lysis vs. Butyrate	Lysis vs. 500μM	77.67	No	ns	B-Q
Lysis vs. Butyrate	Lysis vs. 750μM	72.33	No	ns	B-R
Lysis vs. Butyrate	Lysis vs. 1mM	62.67	No	ns	B-S
Lysis vs. Butyrate	Lysis vs. 2.5mM	45.33	No	ns	B-T

Lysis vs. Butyrate	Lysis vs. 5mM	23	No	ns	B-U
Lysis vs. Butyrate	Lysis vs. 7.5mM	25.33	No	ns	B-V
Lysis vs. Butyrate	Lysis vs. 10mM	19	No	ns	B-W
Lysis vs. Butyrate	Lysis vs. 25mM	32.67	No	ns	B-X
Lysis vs. Combo	Lysis vs. 50pg/mL	60.67	No	ns	B-Y
Lysis vs. Combo	Lysis vs. 150pg/mL	55.33	No	ns	B-Z
Lysis vs. Combo	Lysis vs. 250pg/mL	51	No	ns	B-AA
Lysis vs. Combo	Lysis vs. 500pg/mL	38.67	No	ns	B-AB
Lysis vs. Combo	Lysis vs. 1ng/mL	24	No	ns	B-AC
TNFa vs. TNFa	50pg/mL vs. 100pg/mL	-8	No	ns	C-D
TNFa vs. TNFa	50pg/mL vs. 150pg/mL	-13.67	No	ns	C-E
TNFa vs. TNFa	50pg/mL vs. 200pg/mL	-20.67	No	ns	C-F
TNFa vs. TNFa	50pg/mL vs. 250pg/mL	-25.33	No	ns	C-G
TNFa vs. TNFa	50pg/mL vs. 500pg/mL	-40.67	No	ns	C-H
TNFa vs. TNFa	50pg/mL vs. 1ng/mL	-43.67	No	ns	C-I
TNFa vs. TNFa	50pg/mL vs. 2.5ng/mL	-47	No	ns	C-J
TNFa vs. TNFa	50pg/mL vs. 5ng/mL	-51	No	ns	C-K
TNFa vs. TNFa	50pg/mL vs. 10ng/mL	-51	No	ns	C-L
TNFa vs. Butyrate	50pg/mL vs. 50μM	16.33	No	ns	C-M
TNFa vs. Butyrate	50pg/mL vs. 75μM	21.67	No	ns	C-N
TNFa vs. Butyrate	50pg/mL vs. 100μM	29	No	ns	C-O
TNFa vs. Butyrate	50pg/mL vs. 250μM	30.33	No	ns	C-P
TNFa vs. Butyrate	50pg/mL vs. 500μM	22	No	ns	C-Q
TNFa vs. Butyrate	50pg/mL vs. 750μM	16.67	No	ns	C-R
TNFa vs. Butyrate	50pg/mL vs. 1mM	7	No	ns	C-S
TNFa vs. Butyrate	50pg/mL vs. 2.5mM	-10.33	No	ns	C-T
TNFa vs. Butyrate	50pg/mL vs. 5mM	-32.67	No	ns	C-U
TNFa vs. Butyrate	50pg/mL vs. 7.5mM	-30.33	No	ns	C-V
TNFa vs. Butyrate	50pg/mL vs. 10mM	-36.67	No	ns	C-W
TNFa vs. Butyrate	50pg/mL vs. 25mM	-23	No	ns	C-X
TNFa vs. Combo	50pg/mL vs. 50pg/mL	5	No	ns	C-Y
TNFa vs. Combo	50pg/mL vs. 150pg/mL	-0.3333	No	ns	C-Z
TNFa vs. Combo	50pg/mL vs. 250pg/mL	-4.667	No	ns	C-AA
TNFa vs. Combo	50pg/mL vs. 500pg/mL	-17	No	ns	C-AB
TNFa vs. Combo	50pg/mL vs. 1ng/mL	-31.67	No	ns	C-AC
TNFa vs. TNFa	100pg/mL vs. 150pg/mL	-5.667	No	ns	D-E
TNFa vs. TNFa	100pg/mL vs. 200pg/mL	-12.67	No	ns	D-F
TNFa vs. TNFa	100pg/mL vs. 250pg/mL	-17.33	No	ns	D-G
TNFa vs. TNFa	100pg/mL vs. 500pg/mL	-32.67	No	ns	D-H
TNFa vs. TNFa	100pg/mL vs. 1ng/mL	-35.67	No	ns	D-I
TNFa vs. TNFa	100pg/mL vs. 2.5ng/mL	-39	No	ns	D-J
TNFa vs. TNFa	100pg/mL vs. 5ng/mL	-43	No	ns	D-K
TNFa vs. TNFa	100pg/mL vs. 10ng/mL	-43	No	ns	D-L
TNFa vs. Butyrate	100pg/mL vs. 50μM	24.33	No	ns	D-M
TNFa vs. Butyrate	100pg/mL vs. 75μM	29.67	No	ns	D-N
TNFa vs. Butyrate	100pg/mL vs. 100μM	37	No	ns	D-O
TNFa vs. Butyrate	100pg/mL vs. 250μM	38.33	No	ns	D-P

TNFa vs. Butyrate	100pg/mL vs. 500μM	30	No	ns	D-Q
TNFa vs. Butyrate	100pg/mL vs. 750μM	24.67	No	ns	D-R
TNFa vs. Butyrate	100pg/mL vs. 1mM	15	No	ns	D-S
TNFa vs. Butyrate	100pg/mL vs. 2.5mM	-2.333	No	ns	D-T
TNFa vs. Butyrate	100pg/mL vs. 5mM	-24.67	No	ns	D-U
TNFa vs. Butyrate	100pg/mL vs. 7.5mM	-22.33	No	ns	D-V
TNFa vs. Butyrate	100pg/mL vs. 10mM	-28.67	No	ns	D-W
TNFa vs. Butyrate	100pg/mL vs. 25mM	-15	No	ns	D-X
TNFa vs. Combo	100pg/mL vs. 50pg/mL	13	No	ns	D-Y
TNFa vs. Combo	100pg/mL vs. 150pg/mL	7.667	No	ns	D-Z
TNFa vs. Combo	100pg/mL vs. 250pg/mL	3.333	No	ns	D-AA
TNFa vs. Combo	100pg/mL vs. 500pg/mL	-9	No	ns	D-AB
TNFa vs. Combo	100pg/mL vs. 1ng/mL	-23.67	No	ns	D-AC
TNFa vs. TNFa	150pg/mL vs. 200pg/mL	-7	No	ns	E-F
TNFa vs. TNFa	150pg/mL vs. 250pg/mL	-11.67	No	ns	E-G
TNFa vs. TNFa	150pg/mL vs. 500pg/mL	-27	No	ns	E-H
TNFa vs. TNFa	150pg/mL vs. 1ng/mL	-30	No	ns	E-I
TNFa vs. TNFa	150pg/mL vs. 2.5ng/mL	-33.33	No	ns	E-J
TNFa vs. TNFa	150pg/mL vs. 5ng/mL	-37.33	No	ns	E-K
TNFa vs. TNFa	150pg/mL vs. 10ng/mL	-37.33	No	ns	E-L
TNFa vs. Butyrate	150pg/mL vs. 50μM	30	No	ns	E-M
TNFa vs. Butyrate	150pg/mL vs. 75μM	35.33	No	ns	E-N
TNFa vs. Butyrate	150pg/mL vs. 100μM	42.67	No	ns	E-O
TNFa vs. Butyrate	150pg/mL vs. 250μM	44	No	ns	E-P
TNFa vs. Butyrate	150pg/mL vs. 500μM	35.67	No	ns	E-Q
TNFa vs. Butyrate	150pg/mL vs. 750μM	30.33	No	ns	E-R
TNFa vs. Butyrate	150pg/mL vs. 1mM	20.67	No	ns	E-S
TNFa vs. Butyrate	150pg/mL vs. 2.5mM	3.333	No	ns	E-T
TNFa vs. Butyrate	150pg/mL vs. 5mM	-19	No	ns	E-U
TNFa vs. Butyrate	150pg/mL vs. 7.5mM	-16.67	No	ns	E-V
TNFa vs. Butyrate	150pg/mL vs. 10mM	-23	No	ns	E-W
TNFa vs. Butyrate	150pg/mL vs. 25mM	-9.333	No	ns	E-X
TNFa vs. Combo	150pg/mL vs. 50pg/mL	18.67	No	ns	E-Y
TNFa vs. Combo	150pg/mL vs. 150pg/mL	13.33	No	ns	E-Z
TNFa vs. Combo	150pg/mL vs. 250pg/mL	9	No	ns	E-AA
TNFa vs. Combo	150pg/mL vs. 500pg/mL	-3.333	No	ns	E-AB
TNFa vs. Combo	150pg/mL vs. 1ng/mL	-18	No	ns	E-AC
TNFa vs. TNFa	200pg/mL vs. 250pg/mL	-4.667	No	ns	F-G
TNFa vs. TNFa	200pg/mL vs. 500pg/mL	-20	No	ns	F-H
TNFa vs. TNFa	200pg/mL vs. 1ng/mL	-23	No	ns	F-I
TNFa vs. TNFa	200pg/mL vs. 2.5ng/mL	-26.33	No	ns	F-J
TNFa vs. TNFa	200pg/mL vs. 5ng/mL	-30.33	No	ns	F-K
TNFa vs. TNFa	200pg/mL vs. 10ng/mL	-30.33	No	ns	F-L
TNFa vs. Butyrate	200pg/mL vs. 50μM	37	No	ns	F-M
TNFa vs. Butyrate	200pg/mL vs. 75μM	42.33	No	ns	F-N
TNFa vs. Butyrate	200pg/mL vs. 100μM	49.67	No	ns	F-O
TNFa vs. Butyrate	200pg/mL vs. 250μM	51	No	ns	F-P

TNFa vs. Butyrate	200pg/mL vs. 500μM	42.67	No	ns	F-Q
TNFa vs. Butyrate	200pg/mL vs. 750μM	37.33	No	ns	F-R
TNFa vs. Butyrate	200pg/mL vs. 1mM	27.67	No	ns	F-S
TNFa vs. Butyrate	200pg/mL vs. 2.5mM	10.33	No	ns	F-T
TNFa vs. Butyrate	200pg/mL vs. 5mM	-12	No	ns	F-U
TNFa vs. Butyrate	200pg/mL vs. 7.5mM	-9.667	No	ns	F-V
TNFa vs. Butyrate	200pg/mL vs. 10mM	-16	No	ns	F-W
TNFa vs. Butyrate	200pg/mL vs. 25mM	-2.333	No	ns	F-X
TNFa vs. Combo	200pg/mL vs. 50pg/mL	25.67	No	ns	F-Y
TNFa vs. Combo	200pg/mL vs. 150pg/mL	20.33	No	ns	F-Z
TNFa vs. Combo	200pg/mL vs. 250pg/mL	16	No	ns	F-AA
TNFa vs. Combo	200pg/mL vs. 500pg/mL	3.667	No	ns	F-AB
TNFa vs. Combo	200pg/mL vs. 1ng/mL	-11	No	ns	F-AC
TNFa vs. TNFa	250pg/mL vs. 500pg/mL	-15.33	No	ns	G-H
TNFa vs. TNFa	250pg/mL vs. 1ng/mL	-18.33	No	ns	G-I
TNFa vs. TNFa	250pg/mL vs. 2.5ng/mL	-21.67	No	ns	G-J
TNFa vs. TNFa	250pg/mL vs. 5ng/mL	-25.67	No	ns	G-K
TNFa vs. TNFa	250pg/mL vs. 10ng/mL	-25.67	No	ns	G-L
TNFa vs. Butyrate	250pg/mL vs. 50μM	41.67	No	ns	G-M
TNFa vs. Butyrate	250pg/mL vs. 75μM	47	No	ns	G-N
TNFa vs. Butyrate	250pg/mL vs. 100μM	54.33	No	ns	G-O
TNFa vs. Butyrate	250pg/mL vs. 250μM	55.67	No	ns	G-P
TNFa vs. Butyrate	250pg/mL vs. 500μM	47.33	No	ns	G-Q
TNFa vs. Butyrate	250pg/mL vs. 750μM	42	No	ns	G-R
TNFa vs. Butyrate	250pg/mL vs. 1mM	32.33	No	ns	G-S
TNFa vs. Butyrate	250pg/mL vs. 2.5mM	15	No	ns	G-T
TNFa vs. Butyrate	250pg/mL vs. 5mM	-7.333	No	ns	G-U
TNFa vs. Butyrate	250pg/mL vs. 7.5mM	-5	No	ns	G-V
TNFa vs. Butyrate	250pg/mL vs. 10mM	-11.33	No	ns	G-W
TNFa vs. Butyrate	250pg/mL vs. 25mM	2.333	No	ns	G-X
TNFa vs. Combo	250pg/mL vs. 50pg/mL	30.33	No	ns	G-Y
TNFa vs. Combo	250pg/mL vs. 150pg/mL	25	No	ns	G-Z
TNFa vs. Combo	250pg/mL vs. 250pg/mL	20.67	No	ns	G-AA
TNFa vs. Combo	250pg/mL vs. 500pg/mL	8.333	No	ns	G-AB
TNFa vs. Combo	250pg/mL vs. 1ng/mL	-6.333	No	ns	G-AC
TNFa vs. TNFa	500pg/mL vs. 1ng/mL	-3	No	ns	H-I
TNFa vs. TNFa	500pg/mL vs. 2.5ng/mL	-6.333	No	ns	H-J
TNFa vs. TNFa	500pg/mL vs. 5ng/mL	-10.33	No	ns	H-K
TNFa vs. TNFa	500pg/mL vs. 10ng/mL	-10.33	No	ns	H-L
TNFa vs. Butyrate	500pg/mL vs. 50μM	57	No	ns	H-M
TNFa vs. Butyrate	500pg/mL vs. 75μM	62.33	No	ns	H-N
TNFa vs. Butyrate	500pg/mL vs. 100μM	69.67	No	ns	H-O
TNFa vs. Butyrate	500pg/mL vs. 250μM	71	No	ns	H-P
TNFa vs. Butyrate	500pg/mL vs. 500μM	62.67	No	ns	H-Q
TNFa vs. Butyrate	500pg/mL vs. 750μM	57.33	No	ns	H-R
TNFa vs. Butyrate	500pg/mL vs. 1mM	47.67	No	ns	H-S
TNFa vs. Butyrate	500pg/mL vs. 2.5mM	30.33	No	ns	H-T

TNFa vs. Butyrate	500pg/mL vs. 5mM	8	No	ns	H-U
TNFa vs. Butyrate	500pg/mL vs. 7.5mM	10.33	No	ns	H-V
TNFa vs. Butyrate	500pg/mL vs. 10mM	4	No	ns	H-W
TNFa vs. Butyrate	500pg/mL vs. 25mM	17.67	No	ns	H-X
TNFa vs. Combo	500pg/mL vs. 50pg/mL	45.67	No	ns	H-Y
TNFa vs. Combo	500pg/mL vs. 150pg/mL	40.33	No	ns	H-Z
TNFa vs. Combo	500pg/mL vs. 250pg/mL	36	No	ns	H-AA
TNFa vs. Combo	500pg/mL vs. 500pg/mL	23.67	No	ns	H-AB
TNFa vs. Combo	500pg/mL vs. 1ng/mL	9	No	ns	H-AC
TNFa vs. TNFa	1ng/mL vs. 2.5ng/mL	-3.333	No	ns	I-J
TNFa vs. TNFa	1ng/mL vs. 5ng/mL	-7.333	No	ns	I-K
TNFa vs. TNFa	1ng/mL vs. 10ng/mL	-7.333	No	ns	I-L
TNFa vs. Butyrate	1ng/mL vs. 50μM	60	No	ns	I-M
TNFa vs. Butyrate	1ng/mL vs. 75μM	65.33	No	ns	I-N
TNFa vs. Butyrate	1ng/mL vs. 100μM	72.67	No	ns	I-O
TNFa vs. Butyrate	1ng/mL vs. 250μM	74	No	ns	I-P
TNFa vs. Butyrate	1ng/mL vs. 500μM	65.67	No	ns	I-Q
TNFa vs. Butyrate	1ng/mL vs. 750μM	60.33	No	ns	I-R
TNFa vs. Butyrate	1ng/mL vs. 1mM	50.67	No	ns	I-S
TNFa vs. Butyrate	1ng/mL vs. 2.5mM	33.33	No	ns	I-T
TNFa vs. Butyrate	1ng/mL vs. 5mM	11	No	ns	I-U
TNFa vs. Butyrate	1ng/mL vs. 7.5mM	13.33	No	ns	I-V
TNFa vs. Butyrate	1ng/mL vs. 10mM	7	No	ns	I-W
TNFa vs. Butyrate	1ng/mL vs. 25mM	20.67	No	ns	I-X
TNFa vs. Combo	1ng/mL vs. 50pg/mL	48.67	No	ns	I-Y
TNFa vs. Combo	1ng/mL vs. 150pg/mL	43.33	No	ns	I-Z
TNFa vs. Combo	1ng/mL vs. 250pg/mL	39	No	ns	I-AA
TNFa vs. Combo	1ng/mL vs. 500pg/mL	26.67	No	ns	I-AB
TNFa vs. Combo	1ng/mL vs. 1ng/mL	12	No	ns	I-AC
TNFa vs. TNFa	2.5ng/mL vs. 5ng/mL	-4	No	ns	J-K
TNFa vs. TNFa	2.5ng/mL vs. 10ng/mL	-4	No	ns	J-L
TNFa vs. Butyrate	2.5ng/mL vs. 50μM	63.33	No	ns	J-M
TNFa vs. Butyrate	2.5ng/mL vs. 75μM	68.67	No	ns	J-N
TNFa vs. Butyrate	2.5ng/mL vs. 100μM	76	No	ns	J-O
TNFa vs. Butyrate	2.5ng/mL vs. 250μM	77.33	No	ns	J-P
TNFa vs. Butyrate	2.5ng/mL vs. 500μM	69	No	ns	J-Q
TNFa vs. Butyrate	2.5ng/mL vs. 750μM	63.67	No	ns	J-R
TNFa vs. Butyrate	2.5ng/mL vs. 1mM	54	No	ns	J-S
TNFa vs. Butyrate	2.5ng/mL vs. 2.5mM	36.67	No	ns	J-T
TNFa vs. Butyrate	2.5ng/mL vs. 5mM	14.33	No	ns	J-U
TNFa vs. Butyrate	2.5ng/mL vs. 7.5mM	16.67	No	ns	J-V
TNFa vs. Butyrate	2.5ng/mL vs. 10mM	10.33	No	ns	J-W
TNFa vs. Butyrate	2.5ng/mL vs. 25mM	24	No	ns	J-X
TNFa vs. Combo	2.5ng/mL vs. 50pg/mL	52	No	ns	J-Y
TNFa vs. Combo	2.5ng/mL vs. 150pg/mL	46.67	No	ns	J-Z
TNFa vs. Combo	2.5ng/mL vs. 250pg/mL	42.33	No	ns	J-AA
TNFa vs. Combo	2.5ng/mL vs. 500pg/mL	30	No	ns	J-AB

TNFa vs. Combo	2.5ng/mL vs. 1ng/mL	15.33	No	ns	J-AC
TNFa vs. TNFa	5ng/mL vs. 10ng/mL	0	No	ns	K-L
TNFa vs. Butyrate	5ng/mL vs. 50µM	67.33	No	ns	K-M
TNFa vs. Butyrate	5ng/mL vs. 75µM	72.67	No	ns	K-N
TNFa vs. Butyrate	5ng/mL vs. 100µM	80	No	ns	K-O
TNFa vs. Butyrate	5ng/mL vs. 250µM	81.33	No	ns	K-P
TNFa vs. Butyrate	5ng/mL vs. 500µM	73	No	ns	K-Q
TNFa vs. Butyrate	5ng/mL vs. 750µM	67.67	No	ns	K-R
TNFa vs. Butyrate	5ng/mL vs. 1mM	58	No	ns	K-S
TNFa vs. Butyrate	5ng/mL vs. 2.5mM	40.67	No	ns	K-T
TNFa vs. Butyrate	5ng/mL vs. 5mM	18.33	No	ns	K-U
TNFa vs. Butyrate	5ng/mL vs. 7.5mM	20.67	No	ns	K-V
TNFa vs. Butyrate	5ng/mL vs. 10mM	14.33	No	ns	K-W
TNFa vs. Butyrate	5ng/mL vs. 25mM	28	No	ns	K-X
TNFa vs. Combo	5ng/mL vs. 50pg/mL	56	No	ns	K-Y
TNFa vs. Combo	5ng/mL vs. 150pg/mL	50.67	No	ns	K-Z
TNFa vs. Combo	5ng/mL vs. 250pg/mL	46.33	No	ns	K-AA
TNFa vs. Combo	5ng/mL vs. 500pg/mL	34	No	ns	K-AB
TNFa vs. Combo	5ng/mL vs. 1ng/mL	19.33	No	ns	K-AC
TNFa vs. Butyrate	10ng/mL vs. 50µM	67.33	No	ns	L-M
TNFa vs. Butyrate	10ng/mL vs. 75µM	72.67	No	ns	L-N
TNFa vs. Butyrate	10ng/mL vs. 100µM	80	No	ns	L-O
TNFa vs. Butyrate	10ng/mL vs. 250µM	81.33	No	ns	L-P
TNFa vs. Butyrate	10ng/mL vs. 500µM	73	No	ns	L-Q
TNFa vs. Butyrate	10ng/mL vs. 750µM	67.67	No	ns	L-R
TNFa vs. Butyrate	10ng/mL vs. 1mM	58	No	ns	L-S
TNFa vs. Butyrate	10ng/mL vs. 2.5mM	40.67	No	ns	L-T
TNFa vs. Butyrate	10ng/mL vs. 5mM	18.33	No	ns	L-U
TNFa vs. Butyrate	10ng/mL vs. 7.5mM	20.67	No	ns	L-V
TNFa vs. Butyrate	10ng/mL vs. 10mM	14.33	No	ns	L-W
TNFa vs. Butyrate	10ng/mL vs. 25mM	28	No	ns	L-X
TNFa vs. Combo	10ng/mL vs. 50pg/mL	56	No	ns	L-Y
TNFa vs. Combo	10ng/mL vs. 150pg/mL	50.67	No	ns	L-Z
TNFa vs. Combo	10ng/mL vs. 250pg/mL	46.33	No	ns	L-AA
TNFa vs. Combo	10ng/mL vs. 500pg/mL	34	No	ns	L-AB
TNFa vs. Combo	10ng/mL vs. 1ng/mL	19.33	No	ns	L-AC
Butyrate vs. Butyrate	50µM vs. 75µM	5.333	No	ns	M-N
Butyrate vs. Butyrate	50µM vs. 100µM	12.67	No	ns	M-O
Butyrate vs. Butyrate	50µM vs. 250µM	14	No	ns	M-P
Butyrate vs. Butyrate	50µM vs. 500µM	5.667	No	ns	M-Q
Butyrate vs. Butyrate	50µM vs. 750µM	0.3333	No	ns	M-R
Butyrate vs. Butyrate	50µM vs. 1mM	-9.333	No	ns	M-S
Butyrate vs. Butyrate	50µM vs. 2.5mM	-26.67	No	ns	M-T
Butyrate vs. Butyrate	50µM vs. 5mM	-49	No	ns	M-U
Butyrate vs. Butyrate	50µM vs. 7.5mM	-46.67	No	ns	M-V
Butyrate vs. Butyrate	50µM vs. 10mM	-53	No	ns	M-W
Butyrate vs. Butyrate	50µM vs. 25mM	-39.33	No	ns	M-X

Butyrate vs. Combo	50µM vs. 50pg/mL	-11.33	No	ns	M-Y
Butyrate vs. Combo	50µM vs. 150pg/mL	-16.67	No	ns	M-Z
Butyrate vs. Combo	50µM vs. 250pg/mL	-21	No	ns	M-AA
Butyrate vs. Combo	50µM vs. 500pg/mL	-33.33	No	ns	M-AB
Butyrate vs. Combo	50µM vs. 1ng/mL	-48	No	ns	M-AC
Butyrate vs. Butyrate	75µM vs. 100µM	7.333	No	ns	N-O
Butyrate vs. Butyrate	75µM vs. 250µM	8.667	No	ns	N-P
Butyrate vs. Butyrate	75µM vs. 500µM	0.3333	No	ns	N-Q
Butyrate vs. Butyrate	75µM vs. 750µM	-5	No	ns	N-R
Butyrate vs. Butyrate	75µM vs. 1mM	-14.67	No	ns	N-S
Butyrate vs. Butyrate	75µM vs. 2.5mM	-32	No	ns	N-T
Butyrate vs. Butyrate	75µM vs. 5mM	-54.33	No	ns	N-U
Butyrate vs. Butyrate	75µM vs. 7.5mM	-52	No	ns	N-V
Butyrate vs. Butyrate	75µM vs. 10mM	-58.33	No	ns	N-W
Butyrate vs. Butyrate	75µM vs. 25mM	-44.67	No	ns	N-X
Butyrate vs. Combo	75µM vs. 50pg/mL	-16.67	No	ns	N-Y
Butyrate vs. Combo	75µM vs. 150pg/mL	-22	No	ns	N-Z
Butyrate vs. Combo	75µM vs. 250pg/mL	-26.33	No	ns	N-AA
Butyrate vs. Combo	75µM vs. 500pg/mL	-38.67	No	ns	N-AB
Butyrate vs. Combo	75µM vs. 1ng/mL	-53.33	No	ns	N-AC
Butyrate vs. Butyrate	100µM vs. 250µM	1.333	No	ns	O-P
Butyrate vs. Butyrate	100µM vs. 500µM	-7	No	ns	O-Q
Butyrate vs. Butyrate	100µM vs. 750µM	-12.33	No	ns	O-R
Butyrate vs. Butyrate	100µM vs. 1mM	-22	No	ns	O-S
Butyrate vs. Butyrate	100µM vs. 2.5mM	-39.33	No	ns	O-T
Butyrate vs. Butyrate	100µM vs. 5mM	-61.67	No	ns	O-U
Butyrate vs. Butyrate	100µM vs. 7.5mM	-59.33	No	ns	O-V
Butyrate vs. Butyrate	100µM vs. 10mM	-65.67	No	ns	O-W
Butyrate vs. Butyrate	100µM vs. 25mM	-52	No	ns	O-X
Butyrate vs. Combo	100µM vs. 50pg/mL	-24	No	ns	O-Y
Butyrate vs. Combo	100µM vs. 150pg/mL	-29.33	No	ns	O-Z
Butyrate vs. Combo	100µM vs. 250pg/mL	-33.67	No	ns	O-AA
Butyrate vs. Combo	100µM vs. 500pg/mL	-46	No	ns	O-AB
Butyrate vs. Combo	100µM vs. 1ng/mL	-60.67	No	ns	O-AC
Butyrate vs. Butyrate	250µM vs. 500µM	-8.333	No	ns	P-Q
Butyrate vs. Butyrate	250µM vs. 750µM	-13.67	No	ns	P-R
Butyrate vs. Butyrate	250µM vs. 1mM	-23.33	No	ns	P-S
Butyrate vs. Butyrate	250µM vs. 2.5mM	-40.67	No	ns	P-T
Butyrate vs. Butyrate	250µM vs. 5mM	-63	No	ns	P-U
Butyrate vs. Butyrate	250µM vs. 7.5mM	-60.67	No	ns	P-V
Butyrate vs. Butyrate	250µM vs. 10mM	-67	No	ns	P-W
Butyrate vs. Butyrate	250µM vs. 25mM	-53.33	No	ns	P-X
Butyrate vs. Combo	250µM vs. 50pg/mL	-25.33	No	ns	P-Y
Butyrate vs. Combo	250µM vs. 150pg/mL	-30.67	No	ns	P-Z
Butyrate vs. Combo	250µM vs. 250pg/mL	-35	No	ns	P-AA
Butyrate vs. Combo	250µM vs. 500pg/mL	-47.33	No	ns	P-AB
Butyrate vs. Combo	250µM vs. 1ng/mL	-62	No	ns	P-AC

Butyrate vs. Butyrate	500µM vs. 750µM	-5.333	No	ns	Q-R
Butyrate vs. Butyrate	500µM vs. 1mM	-15	No	ns	Q-S
Butyrate vs. Butyrate	500µM vs. 2.5mM	-32.33	No	ns	Q-T
Butyrate vs. Butyrate	500µM vs. 5mM	-54.67	No	ns	Q-U
Butyrate vs. Butyrate	500µM vs. 7.5mM	-52.33	No	ns	Q-V
Butyrate vs. Butyrate	500µM vs. 10mM	-58.67	No	ns	Q-W
Butyrate vs. Butyrate	500µM vs. 25mM	-45	No	ns	Q-X
Butyrate vs. Combo	500µM vs. 50pg/mL	-17	No	ns	Q-Y
Butyrate vs. Combo	500µM vs. 150pg/mL	-22.33	No	ns	Q-Z
Butyrate vs. Combo	500µM vs. 250pg/mL	-26.67	No	ns	Q-AA
Butyrate vs. Combo	500µM vs. 500pg/mL	-39	No	ns	Q-AB
Butyrate vs. Combo	500µM vs. 1ng/mL	-53.67	No	ns	Q-AC
Butyrate vs. Butyrate	750µM vs. 1mM	-9.667	No	ns	R-S
Butyrate vs. Butyrate	750µM vs. 2.5mM	-27	No	ns	R-T
Butyrate vs. Butyrate	750µM vs. 5mM	-49.33	No	ns	R-U
Butyrate vs. Butyrate	750µM vs. 7.5mM	-47	No	ns	R-V
Butyrate vs. Butyrate	750µM vs. 10mM	-53.33	No	ns	R-W
Butyrate vs. Butyrate	750µM vs. 25mM	-39.67	No	ns	R-X
Butyrate vs. Combo	750µM vs. 50pg/mL	-11.67	No	ns	R-Y
Butyrate vs. Combo	750µM vs. 150pg/mL	-17	No	ns	R-Z
Butyrate vs. Combo	750µM vs. 250pg/mL	-21.33	No	ns	R-AA
Butyrate vs. Combo	750µM vs. 500pg/mL	-33.67	No	ns	R-AB
Butyrate vs. Combo	750µM vs. 1ng/mL	-48.33	No	ns	R-AC
Butyrate vs. Butyrate	1mM vs. 2.5mM	-17.33	No	ns	S-T
Butyrate vs. Butyrate	1mM vs. 5mM	-39.67	No	ns	S-U
Butyrate vs. Butyrate	1mM vs. 7.5mM	-37.33	No	ns	S-V
Butyrate vs. Butyrate	1mM vs. 10mM	-43.67	No	ns	S-W
Butyrate vs. Butyrate	1mM vs. 25mM	-30	No	ns	S-X
Butyrate vs. Combo	1mM vs. 50pg/mL	-2	No	ns	S-Y
Butyrate vs. Combo	1mM vs. 150pg/mL	-7.333	No	ns	S-Z
Butyrate vs. Combo	1mM vs. 250pg/mL	-11.67	No	ns	S-AA
Butyrate vs. Combo	1mM vs. 500pg/mL	-24	No	ns	S-AB
Butyrate vs. Combo	1mM vs. 1ng/mL	-38.67	No	ns	S-AC
Butyrate vs. Butyrate	2.5mM vs. 5mM	-22.33	No	ns	T-U
Butyrate vs. Butyrate	2.5mM vs. 7.5mM	-20	No	ns	T-V
Butyrate vs. Butyrate	2.5mM vs. 10mM	-26.33	No	ns	T-W
Butyrate vs. Butyrate	2.5mM vs. 25mM	-12.67	No	ns	T-X
Butyrate vs. Combo	2.5mM vs. 50pg/mL	15.33	No	ns	T-Y
Butyrate vs. Combo	2.5mM vs. 150pg/mL	10	No	ns	T-Z
Butyrate vs. Combo	2.5mM vs. 250pg/mL	5.667	No	ns	T-AA
Butyrate vs. Combo	2.5mM vs. 500pg/mL	-6.667	No	ns	T-AB
Butyrate vs. Combo	2.5mM vs. 1ng/mL	-21.33	No	ns	T-AC
Butyrate vs. Butyrate	5mM vs. 7.5mM	2.333	No	ns	U-V
Butyrate vs. Butyrate	5mM vs. 10mM	-4	No	ns	U-W
Butyrate vs. Butyrate	5mM vs. 25mM	9.667	No	ns	U-X
Butyrate vs. Combo	5mM vs. 50pg/mL	37.67	No	ns	U-Y
Butyrate vs. Combo	5mM vs. 150pg/mL	32.33	No	ns	U-Z

Butyrate vs. Combo	5mM vs. 250pg/mL	28	No	ns	U-AA
Butyrate vs. Combo	5mM vs. 500pg/mL	15.67	No	ns	U-AB
Butyrate vs. Combo	5mM vs. 1ng/mL	1	No	ns	U-AC
Butyrate vs. Butyrate	7.5mM vs. 10mM	-6.333	No	ns	V-W
Butyrate vs. Butyrate	7.5mM vs. 25mM	7.333	No	ns	V-X
Butyrate vs. Combo	7.5mM vs. 50pg/mL	35.33	No	ns	V-Y
Butyrate vs. Combo	7.5mM vs. 150pg/mL	30	No	ns	V-Z
Butyrate vs. Combo	7.5mM vs. 250pg/mL	25.67	No	ns	V-AA
Butyrate vs. Combo	7.5mM vs. 500pg/mL	13.33	No	ns	V-AB
Butyrate vs. Combo	7.5mM vs. 1ng/mL	-1.333	No	ns	V-AC
Butyrate vs. Butyrate	10mM vs. 25mM	13.67	No	ns	W-X
Butyrate vs. Combo	10mM vs. 50pg/mL	41.67	No	ns	W-Y
Butyrate vs. Combo	10mM vs. 150pg/mL	36.33	No	ns	W-Z
Butyrate vs. Combo	10mM vs. 250pg/mL	32	No	ns	W-AA
Butyrate vs. Combo	10mM vs. 500pg/mL	19.67	No	ns	W-AB
Butyrate vs. Combo	10mM vs. 1ng/mL	5	No	ns	W-AC
Butyrate vs. Combo	25mM vs. 50pg/mL	28	No	ns	X-Y
Butyrate vs. Combo	25mM vs. 150pg/mL	22.67	No	ns	X-Z
Butyrate vs. Combo	25mM vs. 250pg/mL	18.33	No	ns	X-AA
Butyrate vs. Combo	25mM vs. 500pg/mL	6	No	ns	X-AB
Butyrate vs. Combo	25mM vs. 1ng/mL	-8.667	No	ns	X-AC
Combo vs. Combo	50pg/mL vs. 150pg/mL	-5.333	No	ns	Y-Z
Combo vs. Combo	50pg/mL vs. 250pg/mL	-9.667	No	ns	Y-AA
Combo vs. Combo	50pg/mL vs. 500pg/mL	-22	No	ns	Y-AB
Combo vs. Combo	50pg/mL vs. 1ng/mL	-36.67	No	ns	Y-AC
Combo vs. Combo	150pg/mL vs. 250pg/mL	-4.333	No	ns	Z-AA
Combo vs. Combo	150pg/mL vs. 500pg/mL	-16.67	No	ns	Z-AB
Combo vs. Combo	150pg/mL vs. 1ng/mL	-31.33	No	ns	Z-AC
Combo vs. Combo	250pg/mL vs. 500pg/mL	-12.33	No	ns	AA-AB
Combo vs. Combo	250pg/mL vs. 1ng/mL	-27	No	ns	AA-AC
Combo vs. Combo	500pg/mL vs. 1ng/mL	-14.67			

Dunn's multiple comparisons test	Mean rank diff.	Significant?	Summary
Media vs. 8µM FCCP	50.17	No	ns
Media vs. 10pg/mL TNFa	-21.67	No	ns
Media vs. 25pg/mL TNFa	-21.83	No	ns
Media vs. 50pg/mL TNFa	12	No	ns
Media vs. 100pg/mL TNFa	17.33	No	ns
Media vs. 250pg/mL TNFa	72	No	ns
Media vs. 10µM Butyrate	-43.17	No	ns
Media vs. 25µM Butyrate	-16.83	No	ns
Media vs. 50µM Butyrate	-43	No	ns
Media vs. 100µM Butyrate	-25.67	No	ns
Media vs. 250µM Butyrate	21.17	No	ns
Media vs. 500µM Butyrate	58	No	ns
Media vs. 10pg/mL Combo	-36.33	No	ns
Media vs. 25pg/mL Combo	-3.333	No	ns
Media vs. 50pg/mL Combo	5.667	No	ns
Media vs. 100pg/mL Combo	34.5	No	ns
Media vs. 250pg/mL Combo	80.5	No	ns
Media vs. 10pg/mL Combo	39.83	No	ns
Media vs. 25pg/mL Combo	39.33	No	ns
Media vs. 50pg/mL Combo	52.33	No	ns
Media vs. 100pg/mL Combo	63.83	No	ns
Media vs. 250pg/mL Combo	79.17	No	ns
8µM FCCP vs. 10pg/mL TNFa	-71.83	No	ns
8µM FCCP vs. 25pg/mL TNFa	-72	No	ns
8µM FCCP vs. 50pg/mL TNFa	-38.17	No	ns
8µM FCCP vs. 100pg/mL TNFa	-32.83	No	ns
8µM FCCP vs. 250pg/mL TNFa	21.83	No	ns
8µM FCCP vs. 10µM Butyrate	-93.33	Yes	*
8µM FCCP vs. 25µM Butyrate	-67	No	ns
8µM FCCP vs. 50µM Butyrate	-93.17	Yes	*
8µM FCCP vs. 100µM Butyrate	-75.83	No	ns
8µM FCCP vs. 250µM Butyrate	-29	No	ns
8µM FCCP vs. 500µM Butyrate	7.833	No	ns
8µM FCCP vs. 10pg/mL Combo	-86.5	Yes	*
8µM FCCP vs. 25pg/mL Combo	-53.5	No	ns
8µM FCCP vs. 50pg/mL Combo	-44.5	No	ns
8µM FCCP vs. 100pg/mL Combo	-15.67	No	ns
8µM FCCP vs. 250pg/mL Combo	30.33	No	ns
8µM FCCP vs. 10pg/mL Combo	-10.33	No	ns
8µM FCCP vs. 25pg/mL Combo	-10.83	No	ns
8µM FCCP vs. 50pg/mL Combo	2.167	No	ns
8µM FCCP vs. 100pg/mL Combo	13.67	No	ns
8µM FCCP vs. 250pg/mL Combo	29	No	ns
10pg/mL TNFa vs. 25pg/mL TNFa	-0.1667	No	ns
10pg/mL TNFa vs. 50pg/mL TNFa	33.67	No	ns
10pg/mL TNFa vs. 100pg/mL TNFa	39	No	ns

10pg/mL TNFa vs. 250pg/mL TNFa	93.67	Yes	*
10pg/mL TNFa vs. 10µM Butyrate	-21.5	No	ns
10pg/mL TNFa vs. 25µM Butyrate	4.833	No	ns
10pg/mL TNFa vs. 50µM Butyrate	-21.33	No	ns
10pg/mL TNFa vs. 100µM Butyrate	-4	No	ns
10pg/mL TNFa vs. 250µM Butyrate	42.83	No	ns
10pg/mL TNFa vs. 500µM Butyrate	79.67	No	ns
10pg/mL TNFa vs. 10pg/mL Combo	-14.67	No	ns
10pg/mL TNFa vs. 25pg/mL Combo	18.33	No	ns
10pg/mL TNFa vs. 50pg/mL Combo	27.33	No	ns
10pg/mL TNFa vs. 100pg/mL Combo	56.17	No	ns
10pg/mL TNFa vs. 250pg/mL Combo	102.2	Yes	**
10pg/mL TNFa vs. 10pg/mL Combo	61.5	No	ns
10pg/mL TNFa vs. 25pg/mL Combo	61	No	ns
10pg/mL TNFa vs. 50pg/mL Combo	74	No	ns
10pg/mL TNFa vs. 100pg/mL Combo	85.5	No	ns
10pg/mL TNFa vs. 250pg/mL Combo	100.8	Yes	**
25pg/mL TNFa vs. 50pg/mL TNFa	33.83	No	ns
25pg/mL TNFa vs. 100pg/mL TNFa	39.17	No	ns
25pg/mL TNFa vs. 250pg/mL TNFa	93.83	Yes	*
25pg/mL TNFa vs. 10µM Butyrate	-21.33	No	ns
25pg/mL TNFa vs. 25µM Butyrate	5	No	ns
25pg/mL TNFa vs. 50µM Butyrate	-21.17	No	ns
25pg/mL TNFa vs. 100µM Butyrate	-3.833	No	ns
25pg/mL TNFa vs. 250µM Butyrate	43	No	ns
25pg/mL TNFa vs. 500µM Butyrate	79.83	No	ns
25pg/mL TNFa vs. 10pg/mL Combo	-14.5	No	ns
25pg/mL TNFa vs. 25pg/mL Combo	18.5	No	ns
25pg/mL TNFa vs. 50pg/mL Combo	27.5	No	ns
25pg/mL TNFa vs. 100pg/mL Combo	56.33	No	ns
25pg/mL TNFa vs. 250pg/mL Combo	102.3	Yes	**
25pg/mL TNFa vs. 10pg/mL Combo	61.67	No	ns
25pg/mL TNFa vs. 25pg/mL Combo	61.17	No	ns
25pg/mL TNFa vs. 50pg/mL Combo	74.17	No	ns
25pg/mL TNFa vs. 100pg/mL Combo	85.67	No	ns
25pg/mL TNFa vs. 250pg/mL Combo	101	Yes	**
50pg/mL TNFa vs. 100pg/mL TNFa	5.333	No	ns
50pg/mL TNFa vs. 250pg/mL TNFa	60	No	ns
50pg/mL TNFa vs. 10µM Butyrate	-55.17	No	ns
50pg/mL TNFa vs. 25µM Butyrate	-28.83	No	ns
50pg/mL TNFa vs. 50µM Butyrate	-55	No	ns
50pg/mL TNFa vs. 100µM Butyrate	-37.67	No	ns
50pg/mL TNFa vs. 250µM Butyrate	9.167	No	ns
50pg/mL TNFa vs. 500µM Butyrate	46	No	ns
50pg/mL TNFa vs. 10pg/mL Combo	-48.33	No	ns
50pg/mL TNFa vs. 25pg/mL Combo	-15.33	No	ns
50pg/mL TNFa vs. 50pg/mL Combo	-6.333	No	ns

50pg/mL TNFa vs. 100pg/mL Combo	22.5	No	ns
50pg/mL TNFa vs. 250pg/mL Combo	68.5	No	ns
50pg/mL TNFa vs. 10pg/mL Combo	27.83	No	ns
50pg/mL TNFa vs. 25pg/mL Combo	27.33	No	ns
50pg/mL TNFa vs. 50pg/mL Combo	40.33	No	ns
50pg/mL TNFa vs. 100pg/mL Combo	51.83	No	ns
50pg/mL TNFa vs. 250pg/mL Combo	67.17	No	ns
100pg/mL TNFa vs. 250pg/mL TNFa	54.67	No	ns
100pg/mL TNFa vs. 10µM Butyrate	-60.5	No	ns
100pg/mL TNFa vs. 25µM Butyrate	-34.17	No	ns
100pg/mL TNFa vs. 50µM Butyrate	-60.33	No	ns
100pg/mL TNFa vs. 100µM Butyrate	-43	No	ns
100pg/mL TNFa vs. 250µM Butyrate	3.833	No	ns
100pg/mL TNFa vs. 500µM Butyrate	40.67	No	ns
100pg/mL TNFa vs. 10pg/mL Combo	-53.67	No	ns
100pg/mL TNFa vs. 25pg/mL Combo	-20.67	No	ns
100pg/mL TNFa vs. 50pg/mL Combo	-11.67	No	ns
100pg/mL TNFa vs. 100pg/mL Combo	17.17	No	ns
100pg/mL TNFa vs. 250pg/mL Combo	63.17	No	ns
100pg/mL TNFa vs. 10pg/mL Combo	22.5	No	ns
100pg/mL TNFa vs. 25pg/mL Combo	22	No	ns
100pg/mL TNFa vs. 50pg/mL Combo	35	No	ns
100pg/mL TNFa vs. 100pg/mL Combo	46.5	No	ns
100pg/mL TNFa vs. 250pg/mL Combo	61.83	No	ns
250pg/mL TNFa vs. 10µM Butyrate	-115.2	Yes	***
250pg/mL TNFa vs. 25µM Butyrate	-88.83	Yes	*
250pg/mL TNFa vs. 50µM Butyrate	-115	Yes	***
250pg/mL TNFa vs. 100µM Butyrate	-97.67	Yes	**
250pg/mL TNFa vs. 250µM Butyrate	-50.83	No	ns
250pg/mL TNFa vs. 500µM Butyrate	-14	No	ns
250pg/mL TNFa vs. 10pg/mL Combo	-108.3	Yes	***
250pg/mL TNFa vs. 25pg/mL Combo	-75.33	No	ns
250pg/mL TNFa vs. 50pg/mL Combo	-66.33	No	ns
250pg/mL TNFa vs. 100pg/mL Combo	-37.5	No	ns
250pg/mL TNFa vs. 250pg/mL Combo	8.5	No	ns
250pg/mL TNFa vs. 10pg/mL Combo	-32.17	No	ns
250pg/mL TNFa vs. 25pg/mL Combo	-32.67	No	ns
250pg/mL TNFa vs. 50pg/mL Combo	-19.67	No	ns
250pg/mL TNFa vs. 100pg/mL Combo	-8.167	No	ns
250pg/mL TNFa vs. 250pg/mL Combo	7.167	No	ns
10µM Butyrate vs. 25µM Butyrate	26.33	No	ns
10µM Butyrate vs. 50µM Butyrate	0.1667	No	ns
10µM Butyrate vs. 100µM Butyrate	17.5	No	ns
10µM Butyrate vs. 250µM Butyrate	64.33	No	ns
10µM Butyrate vs. 500µM Butyrate	101.2	Yes	**
10µM Butyrate vs. 10pg/mL Combo	6.833	No	ns
10µM Butyrate vs. 25pg/mL Combo	39.83	No	ns

10µM Butyrate vs. 50pg/mL Combo	48.83	No	ns
10µM Butyrate vs. 100pg/mL Combo	77.67	No	ns
10µM Butyrate vs. 250pg/mL Combo	123.7	Yes	****
10µM Butyrate vs. 10pg/mL Combo	83	No	ns
10µM Butyrate vs. 25pg/mL Combo	82.5	No	ns
10µM Butyrate vs. 50pg/mL Combo	95.5	Yes	**
10µM Butyrate vs. 100pg/mL Combo	107	Yes	***
10µM Butyrate vs. 250pg/mL Combo	122.3	Yes	****
25µM Butyrate vs. 50µM Butyrate	-26.17	No	ns
25µM Butyrate vs. 100µM Butyrate	-8.833	No	ns
25µM Butyrate vs. 250µM Butyrate	38	No	ns
25µM Butyrate vs. 500µM Butyrate	74.83	No	ns
25µM Butyrate vs. 10pg/mL Combo	-19.5	No	ns
25µM Butyrate vs. 25pg/mL Combo	13.5	No	ns
25µM Butyrate vs. 50pg/mL Combo	22.5	No	ns
25µM Butyrate vs. 100pg/mL Combo	51.33	No	ns
25µM Butyrate vs. 250pg/mL Combo	97.33	Yes	**
25µM Butyrate vs. 10pg/mL Combo	56.67	No	ns
25µM Butyrate vs. 25pg/mL Combo	56.17	No	ns
25µM Butyrate vs. 50pg/mL Combo	69.17	No	ns
25µM Butyrate vs. 100pg/mL Combo	80.67	No	ns
25µM Butyrate vs. 250pg/mL Combo	96	Yes	**
50µM Butyrate vs. 100µM Butyrate	17.33	No	ns
50µM Butyrate vs. 250µM Butyrate	64.17	No	ns
50µM Butyrate vs. 500µM Butyrate	101	Yes	**
50µM Butyrate vs. 10pg/mL Combo	6.667	No	ns
50µM Butyrate vs. 25pg/mL Combo	39.67	No	ns
50µM Butyrate vs. 50pg/mL Combo	48.67	No	ns
50µM Butyrate vs. 100pg/mL Combo	77.5	No	ns
50µM Butyrate vs. 250pg/mL Combo	123.5	Yes	****
50µM Butyrate vs. 10pg/mL Combo	82.83	No	ns
50µM Butyrate vs. 25pg/mL Combo	82.33	No	ns
50µM Butyrate vs. 50pg/mL Combo	95.33	Yes	**
50µM Butyrate vs. 100pg/mL Combo	106.8	Yes	***
50µM Butyrate vs. 250pg/mL Combo	122.2	Yes	****
100µM Butyrate vs. 250µM Butyrate	46.83	No	ns
100µM Butyrate vs. 500µM Butyrate	83.67	No	ns
100µM Butyrate vs. 10pg/mL Combo	-10.67	No	ns
100µM Butyrate vs. 25pg/mL Combo	22.33	No	ns
100µM Butyrate vs. 50pg/mL Combo	31.33	No	ns
100µM Butyrate vs. 100pg/mL Combo	60.17	No	ns
100µM Butyrate vs. 250pg/mL Combo	106.2	Yes	**
100µM Butyrate vs. 10pg/mL Combo	65.5	No	ns
100µM Butyrate vs. 25pg/mL Combo	65	No	ns
100µM Butyrate vs. 50pg/mL Combo	78	No	ns
100µM Butyrate vs. 100pg/mL Combo	89.5	Yes	*
100µM Butyrate vs. 250pg/mL Combo	104.8	Yes	**

250µM Butyrate vs. 500µM Butyrate	36.83	No	ns
250µM Butyrate vs. 10pg/mL Combo	-57.5	No	ns
250µM Butyrate vs. 25pg/mL Combo	-24.5	No	ns
250µM Butyrate vs. 50pg/mL Combo	-15.5	No	ns
250µM Butyrate vs. 100pg/mL Combo	13.33	No	ns
250µM Butyrate vs. 250pg/mL Combo	59.33	No	ns
250µM Butyrate vs. 10pg/mL Combo	18.67	No	ns
250µM Butyrate vs. 25pg/mL Combo	18.17	No	ns
250µM Butyrate vs. 50pg/mL Combo	31.17	No	ns
250µM Butyrate vs. 100pg/mL Combo	42.67	No	ns
250µM Butyrate vs. 250pg/mL Combo	58	No	ns
500µM Butyrate vs. 10pg/mL Combo	-94.33	Yes	*
500µM Butyrate vs. 25pg/mL Combo	-61.33	No	ns
500µM Butyrate vs. 50pg/mL Combo	-52.33	No	ns
500µM Butyrate vs. 100pg/mL Combo	-23.5	No	ns
500µM Butyrate vs. 250pg/mL Combo	22.5	No	ns
500µM Butyrate vs. 10pg/mL Combo	-18.17	No	ns
500µM Butyrate vs. 25pg/mL Combo	-18.67	No	ns
500µM Butyrate vs. 50pg/mL Combo	-5.667	No	ns
500µM Butyrate vs. 100pg/mL Combo	5.833	No	ns
500µM Butyrate vs. 250pg/mL Combo	21.17	No	ns
10pg/mL Combo vs. 25pg/mL Combo	33	No	ns
10pg/mL Combo vs. 50pg/mL Combo	42	No	ns
10pg/mL Combo vs. 100pg/mL Combo	70.83	No	ns
10pg/mL Combo vs. 250pg/mL Combo	116.8	Yes	***
10pg/mL Combo vs. 10pg/mL Combo	76.17	No	ns
10pg/mL Combo vs. 25pg/mL Combo	75.67	No	ns
10pg/mL Combo vs. 50pg/mL Combo	88.67	Yes	*
10pg/mL Combo vs. 100pg/mL Combo	100.2	Yes	**
10pg/mL Combo vs. 250pg/mL Combo	115.5	Yes	***
25pg/mL Combo vs. 50pg/mL Combo	9	No	ns
25pg/mL Combo vs. 100pg/mL Combo	37.83	No	ns
25pg/mL Combo vs. 250pg/mL Combo	83.83	No	ns
25pg/mL Combo vs. 10pg/mL Combo	43.17	No	ns
25pg/mL Combo vs. 25pg/mL Combo	42.67	No	ns
25pg/mL Combo vs. 50pg/mL Combo	55.67	No	ns
25pg/mL Combo vs. 100pg/mL Combo	67.17	No	ns
25pg/mL Combo vs. 250pg/mL Combo	82.5	No	ns
50pg/mL Combo vs. 100pg/mL Combo	28.83	No	ns
50pg/mL Combo vs. 250pg/mL Combo	74.83	No	ns
50pg/mL Combo vs. 10pg/mL Combo	34.17	No	ns
50pg/mL Combo vs. 25pg/mL Combo	33.67	No	ns
50pg/mL Combo vs. 50pg/mL Combo	46.67	No	ns
50pg/mL Combo vs. 100pg/mL Combo	58.17	No	ns
50pg/mL Combo vs. 250pg/mL Combo	73.5	No	ns
100pg/mL Combo vs. 250pg/mL Combo	46	No	ns
100pg/mL Combo vs. 10pg/mL Combo	5.333	No	ns

100pg/mL Combo vs. 25pg/mL Combo	4.833	No	ns
100pg/mL Combo vs. 50pg/mL Combo	17.83	No	ns
100pg/mL Combo vs. 100pg/mL Combo	29.33	No	ns
100pg/mL Combo vs. 250pg/mL Combo	44.67	No	ns
250pg/mL Combo vs. 10pg/mL Combo	-40.67	No	ns
250pg/mL Combo vs. 25pg/mL Combo	-41.17	No	ns
250pg/mL Combo vs. 50pg/mL Combo	-28.17	No	ns
250pg/mL Combo vs. 100pg/mL Combo	-16.67	No	ns
250pg/mL Combo vs. 250pg/mL Combo	-1.333	No	ns
10pg/mL Combo vs. 25pg/mL Combo	-0.5	No	ns
10pg/mL Combo vs. 50pg/mL Combo	12.5	No	ns
10pg/mL Combo vs. 100pg/mL Combo	24	No	ns
10pg/mL Combo vs. 250pg/mL Combo	39.33	No	ns
25pg/mL Combo vs. 50pg/mL Combo	13	No	ns
25pg/mL Combo vs. 100pg/mL Combo	24.5	No	ns
25pg/mL Combo vs. 250pg/mL Combo	39.83	No	ns
50pg/mL Combo vs. 100pg/mL Combo	11.5	No	ns
50pg/mL Combo vs. 250pg/mL Combo	26.83	No	ns
100pg/mL Combo vs. 250pg/mL Combo	15.33	No	

A-B
A-C
A-D
A-E
A-F
A-G
A-L
A-M
A-N
A-O
A-P
A-Q
A-V
A-W
A-X
A-Y
A-Z
A-AA
A-AB
A-AC
A-AD
A-AE
B-C
B-D
B-E
B-F
B-G
B-L
B-M
B-N
B-O
B-P
B-Q
B-V
B-W
B-X
B-Y
B-Z
B-AA
B-AB
B-AC
B-AD
B-AE
C-D
C-E
C-F

C-G
C-L
C-M
C-N
C-O
C-P
C-Q
C-V
C-W
C-X
C-Y
C-Z
C-AA
C-AB
C-AC
C-AD
C-AE
D-E
D-F
D-G
D-L
D-M
D-N
D-O
D-P
D-Q
D-V
D-W
D-X
D-Y
D-Z
D-AA
D-AB
D-AC
D-AD
D-AE
E-F
E-G
E-L
E-M
E-N
E-O
E-P
E-Q
E-V
E-W
E-X

E-Y
E-Z
E-AA
E-AB
E-AC
E-AD
E-AE
F-G
F-L
F-M
F-N
F-O
F-P
F-Q
F-V
F-W
F-X
F-Y
F-Z
F-AA
F-AB
F-AC
F-AD
F-AE
G-L
G-M
G-N
G-O
G-P
G-Q
G-V
G-W
G-X
G-Y
G-Z
G-AA
G-AB
G-AC
G-AD
G-AE
L-M
L-N
L-O
L-P
L-Q
L-V
L-W

L-X
L-Y
L-Z
L-AA
L-AB
L-AC
L-AD
L-AE
M-N
M-O
M-P
M-Q
M-V
M-W
M-X
M-Y
M-Z
M-AA
M-AB
M-AC
M-AD
M-AE
N-O
N-P
N-Q
N-V
N-W
N-X
N-Y
N-Z
N-AA
N-AB
N-AC
N-AD
N-AE
O-P
O-Q
O-V
O-W
O-X
O-Y
O-Z
O-AA
O-AB
O-AC
O-AD
O-AE

P-Q
P-V
P-W
P-X
P-Y
P-Z
P-AA
P-AB
P-AC
P-AD
P-AE
Q-V
Q-W
Q-X
Q-Y
Q-Z
Q-AA
Q-AB
Q-AC
Q-AD
Q-AE
V-W
V-X
V-Y
V-Z
V-AA
V-AB
V-AC
V-AD
V-AE
W-X
W-Y
W-Z
W-AA
W-AB
W-AC
W-AD
W-AE
X-Y
X-Z
X-AA
X-AB
X-AC
X-AD
X-AE
Y-Z
Y-AA

Y-AB
Y-AC
Y-AD
Y-AE
Z-AA
Z-AB
Z-AC
Z-AD
Z-AE
AA-AB
AA-AC
AA-AD
AA-AE
AB-AC
AB-AD
AB-AE
AC-AD
AC-AE

Treatment	Dunn's multiple comparisons test	Mean rank	Significant?	Summary	Adjusted P
Media vs. AA	Media vs. 10 µM AA	122.6	No	ns	0.0733
Media vs TNFa	Media vs. 50 pg/mL	-17.6	No	ns	>0.9999
Media vs TNFa	Media vs. 100 pg/mL	-0.4603	No	ns	>0.9999
Media vs TNFa	Media vs. 150 pg/mL	2.54	No	ns	>0.9999
Media vs TNFa	Media vs. 200 pg/mL	6.54	No	ns	>0.9999
Media vs TNFa	Media vs. 250 pg/mL	25.4	No	ns	>0.9999
Media vs TNFa	Media vs. 500 pg/mL	61.4	No	ns	>0.9999
Media vs TNFa	Media vs. 1 ng/mL	85.83	No	ns	0.7767
Media vs TNFa	Media vs. 2.5 ng/mL	110.7	Yes	*	0.0217
Media vs TNFa	Media vs. 5 ng/mL	139.1	Yes	***	0.0001
Media vs TNFa	Media vs. 10 ng/mL	148.6	Yes	**	0.002
Media Vs. Butyrate	Media vs. 50 µM	-1.603	No	ns	>0.9999
Media Vs. Butyrate	Media vs. 75 µM	-4.175	No	ns	>0.9999
Media Vs. Butyrate	Media vs. 100 µM	1.254	No	ns	>0.9999
Media Vs. Butyrate	Media vs. 250 µM	41.25	No	ns	>0.9999
Media Vs. Butyrate	Media vs. 500 µM	57.4	No	ns	>0.9999
Media Vs. Butyrate	Media vs. 750 µM	61.54	No	ns	>0.9999
Media Vs. Butyrate	Media vs. 1 mM	54.4	No	ns	>0.9999
Media Vs. Butyrate	Media vs. 2.5 mM	95.25	No	ns	0.2196
Media Vs. Butyrate	Media vs. 5 mM	103.8	No	ns	0.063
Media Vs. Butyrate	Media vs. 7.5 mM	106.3	Yes	*	0.0435
Media Vs. Butyrate	Media vs. 10 mM	115.6	No	ns	0.1728
Media Vs. Butyrate	Media vs. 25 mM	122.6	No	ns	0.0733
Media Vs. Combo	Media vs. 50 pg/mL	46.11	No	ns	>0.9999
Media Vs. Combo	Media vs. 100 pg/mL	66.11	No	ns	>0.9999
Media Vs. Combo	Media vs. 150 pg/mL	58.11	No	ns	>0.9999
Media Vs. Combo	Media vs. 200 pg/mL	60.44	No	ns	>0.9999
Media Vs. Combo	Media vs. 250 pg/mL	60.78	No	ns	>0.9999
Media Vs. Combo	Media vs. 500 pg/mL	77.78	No	ns	>0.9999
Media Vs. Combo	Media vs. 1 ng/mL	94.11	No	ns	>0.9999
Media Vs. Combo	Media vs. 2.5 ng/mL	132.8	No	ns	0.1055
Media Vs. Combo	Media vs. 5 ng/mL	140.8	Yes	*	0.0424
AA vs TNFa	10 µM AA vs. 50 pg/mL	-140.2	Yes	*	0.0156
AA vs TNFa	10 µM AA vs. 100 pg/mL	-123.1	No	ns	0.1297
AA vs TNFa	10 µM AA vs. 150 pg/mL	-120.1	No	ns	0.1832
AA vs TNFa	10 µM AA vs. 200 pg/mL	-116.1	No	ns	0.2871
AA vs TNFa	10 µM AA vs. 250 pg/mL	-97.21	No	ns	>0.9999
AA vs TNFa	10 µM AA vs. 500 pg/mL	-61.21	No	ns	>0.9999
AA vs TNFa	10 µM AA vs. 1 ng/mL	-36.79	No	ns	>0.9999
AA vs TNFa	10 µM AA vs. 2.5 ng/mL	-11.93	No	ns	>0.9999
AA vs TNFa	10 µM AA vs. 5 ng/mL	16.5	No	ns	>0.9999
AA vs TNFa	10 µM AA vs. 10 ng/mL	26	No	ns	>0.9999
AA Vs. Butyrate	10 µM AA vs. 50 µM	-124.2	No	ns	0.1135
AA Vs. Butyrate	10 µM AA vs. 75 µM	-126.8	No	ns	0.0837
AA Vs. Butyrate	10 µM AA vs. 100 µM	-121.4	No	ns	0.1581
AA Vs. Butyrate	10 µM AA vs. 250 µM	-81.36	No	ns	>0.9999

AA Vs. Butyrate	10 µM AA vs. 500 µM	-65.21	No	ns	>0.9999
AA Vs. Butyrate	10 µM AA vs. 750 µM	-61.07	No	ns	>0.9999
AA Vs. Butyrate	10 µM AA vs. 1 mM	-68.21	No	ns	>0.9999
AA Vs. Butyrate	10 µM AA vs. 2.5 mM	-27.36	No	ns	>0.9999
AA Vs. Butyrate	10 µM AA vs. 5 mM	-18.79	No	ns	>0.9999
AA Vs. Butyrate	10 µM AA vs. 7.5 mM	-16.36	No	ns	>0.9999
AA Vs. Butyrate	10 µM AA vs. 10 mM	-7	No	ns	>0.9999
AA Vs. Butyrate	10 µM AA vs. 25 mM	0	No	ns	>0.9999
AA Vs. Combo	10 µM AA vs. 50 pg/mL	-76.5	No	ns	>0.9999
AA Vs. Combo	10 µM AA vs. 100 pg/mL	-56.5	No	ns	>0.9999
AA Vs. Combo	10 µM AA vs. 150 pg/mL	-64.5	No	ns	>0.9999
AA Vs. Combo	10 µM AA vs. 200 pg/mL	-62.17	No	ns	>0.9999
AA Vs. Combo	10 µM AA vs. 250 pg/mL	-61.83	No	ns	>0.9999
AA Vs. Combo	10 µM AA vs. 500 pg/mL	-44.83	No	ns	>0.9999
AA Vs. Combo	10 µM AA vs. 1 ng/mL	-28.5	No	ns	>0.9999
AA Vs. Combo	10 µM AA vs. 2.5 ng/mL	10.17	No	ns	>0.9999
AA Vs. Combo	10 µM AA vs. 5 ng/mL	18.17	No	ns	>0.9999
TNFa vs. TNFa	50 pg/mL vs. 100 pg/mL	17.14	No	ns	>0.9999
TNFa vs. TNFa	50 pg/mL vs. 150 pg/mL	20.14	No	ns	>0.9999
TNFa vs. TNFa	50 pg/mL vs. 200 pg/mL	24.14	No	ns	>0.9999
TNFa vs. TNFa	50 pg/mL vs. 250 pg/mL	43	No	ns	>0.9999
TNFa vs. TNFa	50 pg/mL vs. 500 pg/mL	79	No	ns	>0.9999
TNFa vs. TNFa	50 pg/mL vs. 1 ng/mL	103.4	No	ns	0.1595
TNFa vs. TNFa	50 pg/mL vs. 2.5 ng/mL	128.3	Yes	**	0.0039
TNFa vs. TNFa	50 pg/mL vs. 5 ng/mL	156.7	Yes	****	<0.0001
TNFa vs. TNFa	50 pg/mL vs. 10 ng/mL	166.2	Yes	***	0.0004
TNFa vs. Butyrate	50 pg/mL vs. 50 µM	16	No	ns	>0.9999
TNFa vs. Butyrate	50 pg/mL vs. 75 µM	13.43	No	ns	>0.9999
TNFa vs. Butyrate	50 pg/mL vs. 100 µM	18.86	No	ns	>0.9999
TNFa vs. Butyrate	50 pg/mL vs. 250 µM	58.86	No	ns	>0.9999
TNFa vs. Butyrate	50 pg/mL vs. 500 µM	75	No	ns	>0.9999
TNFa vs. Butyrate	50 pg/mL vs. 750 µM	79.14	No	ns	>0.9999
TNFa vs. Butyrate	50 pg/mL vs. 1 mM	72	No	ns	>0.9999
TNFa vs. Butyrate	50 pg/mL vs. 2.5 mM	112.9	Yes	*	0.0425
TNFa vs. Butyrate	50 pg/mL vs. 5 mM	121.4	Yes	*	0.0117
TNFa vs. Butyrate	50 pg/mL vs. 7.5 mM	123.9	Yes	**	0.008
TNFa vs. Butyrate	50 pg/mL vs. 10 mM	133.2	Yes	*	0.0381
TNFa vs. Butyrate	50 pg/mL vs. 25 mM	140.2	Yes	*	0.0156
TNFa vs. Combo	50 pg/mL vs. 50 pg/mL	63.71	No	ns	>0.9999
TNFa vs. Combo	50 pg/mL vs. 100 pg/mL	83.71	No	ns	>0.9999
TNFa vs. Combo	50 pg/mL vs. 150 pg/mL	75.71	No	ns	>0.9999
TNFa vs. Combo	50 pg/mL vs. 200 pg/mL	78.05	No	ns	>0.9999
TNFa vs. Combo	50 pg/mL vs. 250 pg/mL	78.38	No	ns	>0.9999
TNFa vs. Combo	50 pg/mL vs. 500 pg/mL	95.38	No	ns	>0.9999
TNFa vs. Combo	50 pg/mL vs. 1 ng/mL	111.7	No	ns	>0.9999
TNFa vs. Combo	50 pg/mL vs. 2.5 ng/mL	150.4	Yes	*	0.0249
TNFa vs. Combo	50 pg/mL vs. 5 ng/mL	158.4	Yes	**	0.0096

TNFa vs. TNFa	100 pg/mL vs. 150 pg/mL	3	No	ns	>0.9999
TNFa vs. TNFa	100 pg/mL vs. 200 pg/mL	7	No	ns	>0.9999
TNFa vs. TNFa	100 pg/mL vs. 250 pg/mL	25.86	No	ns	>0.9999
TNFa vs. TNFa	100 pg/mL vs. 500 pg/mL	61.86	No	ns	>0.9999
TNFa vs. TNFa	100 pg/mL vs. 1 ng/mL	86.29	No	ns	>0.9999
TNFa vs. TNFa	100 pg/mL vs. 2.5 ng/mL	111.1	No	ns	0.0545
TNFa vs. TNFa	100 pg/mL vs. 5 ng/mL	139.6	Yes	***	0.0006
TNFa vs. TNFa	100 pg/mL vs. 10 ng/mL	149.1	Yes	**	0.0047
TNFa vs. Butyrate	100 pg/mL vs. 50 µM	-1.143	No	ns	>0.9999
TNFa vs. Butyrate	100 pg/mL vs. 75 µM	-3.714	No	ns	>0.9999
TNFa vs. Butyrate	100 pg/mL vs. 100 µM	1.714	No	ns	>0.9999
TNFa vs. Butyrate	100 pg/mL vs. 250 µM	41.71	No	ns	>0.9999
TNFa vs. Butyrate	100 pg/mL vs. 500 µM	57.86	No	ns	>0.9999
TNFa vs. Butyrate	100 pg/mL vs. 750 µM	62	No	ns	>0.9999
TNFa vs. Butyrate	100 pg/mL vs. 1 mM	54.86	No	ns	>0.9999
TNFa vs. Butyrate	100 pg/mL vs. 2.5 mM	95.71	No	ns	0.4361
TNFa vs. Butyrate	100 pg/mL vs. 5 mM	104.3	No	ns	0.1421
TNFa vs. Butyrate	100 pg/mL vs. 7.5 mM	106.7	No	ns	0.1018
TNFa vs. Butyrate	100 pg/mL vs. 10 mM	116.1	No	ns	0.2871
TNFa vs. Butyrate	100 pg/mL vs. 25 mM	123.1	No	ns	0.1297
TNFa vs. Combo	100 pg/mL vs. 50 pg/mL	46.57	No	ns	>0.9999
TNFa vs. Combo	100 pg/mL vs. 100 pg/mL	66.57	No	ns	>0.9999
TNFa vs. Combo	100 pg/mL vs. 150 pg/mL	58.57	No	ns	>0.9999
TNFa vs. Combo	100 pg/mL vs. 200 pg/mL	60.9	No	ns	>0.9999
TNFa vs. Combo	100 pg/mL vs. 250 pg/mL	61.24	No	ns	>0.9999
TNFa vs. Combo	100 pg/mL vs. 500 pg/mL	78.24	No	ns	>0.9999
TNFa vs. Combo	100 pg/mL vs. 1 ng/mL	94.57	No	ns	>0.9999
TNFa vs. Combo	100 pg/mL vs. 2.5 ng/mL	133.2	No	ns	0.1644
TNFa vs. Combo	100 pg/mL vs. 5 ng/mL	141.2	No	ns	0.0698
TNFa vs. TNFa	150 pg/mL vs. 200 pg/mL	4	No	ns	>0.9999
TNFa vs. TNFa	150 pg/mL vs. 250 pg/mL	22.86	No	ns	>0.9999
TNFa vs. TNFa	150 pg/mL vs. 500 pg/mL	58.86	No	ns	>0.9999
TNFa vs. TNFa	150 pg/mL vs. 1 ng/mL	83.29	No	ns	>0.9999
TNFa vs. TNFa	150 pg/mL vs. 2.5 ng/mL	108.1	No	ns	0.0834
TNFa vs. TNFa	150 pg/mL vs. 5 ng/mL	136.6	Yes	***	0.001
TNFa vs. TNFa	150 pg/mL vs. 10 ng/mL	146.1	Yes	**	0.0071
TNFa vs. Butyrate	150 pg/mL vs. 50 µM	-4.143	No	ns	>0.9999
TNFa vs. Butyrate	150 pg/mL vs. 75 µM	-6.714	No	ns	>0.9999
TNFa vs. Butyrate	150 pg/mL vs. 100 µM	-1.286	No	ns	>0.9999
TNFa vs. Butyrate	150 pg/mL vs. 250 µM	38.71	No	ns	>0.9999
TNFa vs. Butyrate	150 pg/mL vs. 500 µM	54.86	No	ns	>0.9999
TNFa vs. Butyrate	150 pg/mL vs. 750 µM	59	No	ns	>0.9999
TNFa vs. Butyrate	150 pg/mL vs. 1 mM	51.86	No	ns	>0.9999
TNFa vs. Butyrate	150 pg/mL vs. 2.5 mM	92.71	No	ns	0.633
TNFa vs. Butyrate	150 pg/mL vs. 5 mM	101.3	No	ns	0.2124
TNFa vs. Butyrate	150 pg/mL vs. 7.5 mM	103.7	No	ns	0.1535
TNFa vs. Butyrate	150 pg/mL vs. 10 mM	113.1	No	ns	0.3985

TNFa vs. Butyrate	150 pg/mL vs. 25 mM	120.1	No	ns	0.1832
TNFa vs. Combo	150 pg/mL vs. 50 pg/mL	43.57	No	ns	>0.9999
TNFa vs. Combo	150 pg/mL vs. 100 pg/mL	63.57	No	ns	>0.9999
TNFa vs. Combo	150 pg/mL vs. 150 pg/mL	55.57	No	ns	>0.9999
TNFa vs. Combo	150 pg/mL vs. 200 pg/mL	57.9	No	ns	>0.9999
TNFa vs. Combo	150 pg/mL vs. 250 pg/mL	58.24	No	ns	>0.9999
TNFa vs. Combo	150 pg/mL vs. 500 pg/mL	75.24	No	ns	>0.9999
TNFa vs. Combo	150 pg/mL vs. 1 ng/mL	91.57	No	ns	>0.9999
TNFa vs. Combo	150 pg/mL vs. 2.5 ng/mL	130.2	No	ns	0.224
TNFa vs. Combo	150 pg/mL vs. 5 ng/mL	138.2	No	ns	0.0968
TNFa vs. TNFa	200 pg/mL vs. 250 pg/mL	18.86	No	ns	>0.9999
TNFa vs. TNFa	200 pg/mL vs. 500 pg/mL	54.86	No	ns	>0.9999
TNFa vs. TNFa	200 pg/mL vs. 1 ng/mL	79.29	No	ns	>0.9999
TNFa vs. TNFa	200 pg/mL vs. 2.5 ng/mL	104.1	No	ns	0.1448
TNFa vs. TNFa	200 pg/mL vs. 5 ng/mL	132.6	Yes	**	0.0019
TNFa vs. TNFa	200 pg/mL vs. 10 ng/mL	142.1	Yes	*	0.0122
TNFa vs. Butyrate	200 pg/mL vs. 50 µM	-8.143	No	ns	>0.9999
TNFa vs. Butyrate	200 pg/mL vs. 75 µM	-10.71	No	ns	>0.9999
TNFa vs. Butyrate	200 pg/mL vs. 100 µM	-5.286	No	ns	>0.9999
TNFa vs. Butyrate	200 pg/mL vs. 250 µM	34.71	No	ns	>0.9999
TNFa vs. Butyrate	200 pg/mL vs. 500 µM	50.86	No	ns	>0.9999
TNFa vs. Butyrate	200 pg/mL vs. 750 µM	55	No	ns	>0.9999
TNFa vs. Butyrate	200 pg/mL vs. 1 mM	47.86	No	ns	>0.9999
TNFa vs. Butyrate	200 pg/mL vs. 2.5 mM	88.71	No	ns	>0.9999
TNFa vs. Butyrate	200 pg/mL vs. 5 mM	97.29	No	ns	0.3573
TNFa vs. Butyrate	200 pg/mL vs. 7.5 mM	99.71	No	ns	0.2611
TNFa vs. Butyrate	200 pg/mL vs. 10 mM	109.1	No	ns	0.61
TNFa vs. Butyrate	200 pg/mL vs. 25 mM	116.1	No	ns	0.2871
TNFa vs. Combo	200 pg/mL vs. 50 pg/mL	39.57	No	ns	>0.9999
TNFa vs. Combo	200 pg/mL vs. 100 pg/mL	59.57	No	ns	>0.9999
TNFa vs. Combo	200 pg/mL vs. 150 pg/mL	51.57	No	ns	>0.9999
TNFa vs. Combo	200 pg/mL vs. 200 pg/mL	53.9	No	ns	>0.9999
TNFa vs. Combo	200 pg/mL vs. 250 pg/mL	54.24	No	ns	>0.9999
TNFa vs. Combo	200 pg/mL vs. 500 pg/mL	71.24	No	ns	>0.9999
TNFa vs. Combo	200 pg/mL vs. 1 ng/mL	87.57	No	ns	>0.9999
TNFa vs. Combo	200 pg/mL vs. 2.5 ng/mL	126.2	No	ns	0.3352
TNFa vs. Combo	200 pg/mL vs. 5 ng/mL	134.2	No	ns	0.1481
TNFa vs. TNFa	250 pg/mL vs. 500 pg/mL	36	No	ns	>0.9999
TNFa vs. TNFa	250 pg/mL vs. 1 ng/mL	60.43	No	ns	>0.9999
TNFa vs. TNFa	250 pg/mL vs. 2.5 ng/mL	85.29	No	ns	>0.9999
TNFa vs. TNFa	250 pg/mL vs. 5 ng/mL	113.7	Yes	*	0.0375
TNFa vs. TNFa	250 pg/mL vs. 10 ng/mL	123.2	No	ns	0.1275
TNFa vs. Butyrate	250 pg/mL vs. 50 µM	-27	No	ns	>0.9999
TNFa vs. Butyrate	250 pg/mL vs. 75 µM	-29.57	No	ns	>0.9999
TNFa vs. Butyrate	250 pg/mL vs. 100 µM	-24.14	No	ns	>0.9999
TNFa vs. Butyrate	250 pg/mL vs. 250 µM	15.86	No	ns	>0.9999
TNFa vs. Butyrate	250 pg/mL vs. 500 µM	32	No	ns	>0.9999

TNFa vs. Butyrate	250 pg/mL vs. 750 µM	36.14	No	ns	>0.9999
TNFa vs. Butyrate	250 pg/mL vs. 1 mM	29	No	ns	>0.9999
TNFa vs. Butyrate	250 pg/mL vs. 2.5 mM	69.86	No	ns	>0.9999
TNFa vs. Butyrate	250 pg/mL vs. 5 mM	78.43	No	ns	>0.9999
TNFa vs. Butyrate	250 pg/mL vs. 7.5 mM	80.86	No	ns	>0.9999
TNFa vs. Butyrate	250 pg/mL vs. 10 mM	90.21	No	ns	>0.9999
TNFa vs. Butyrate	250 pg/mL vs. 25 mM	97.21	No	ns	>0.9999
TNFa vs. Combo	250 pg/mL vs. 50 pg/mL	20.71	No	ns	>0.9999
TNFa vs. Combo	250 pg/mL vs. 100 pg/mL	40.71	No	ns	>0.9999
TNFa vs. Combo	250 pg/mL vs. 150 pg/mL	32.71	No	ns	>0.9999
TNFa vs. Combo	250 pg/mL vs. 200 pg/mL	35.05	No	ns	>0.9999
TNFa vs. Combo	250 pg/mL vs. 250 pg/mL	35.38	No	ns	>0.9999
TNFa vs. Combo	250 pg/mL vs. 500 pg/mL	52.38	No	ns	>0.9999
TNFa vs. Combo	250 pg/mL vs. 1 ng/mL	68.71	No	ns	>0.9999
TNFa vs. Combo	250 pg/mL vs. 2.5 ng/mL	107.4	No	ns	>0.9999
TNFa vs. Combo	250 pg/mL vs. 5 ng/mL	115.4	No	ns	0.9471
TNFa vs. TNFa	500 pg/mL vs. 1 ng/mL	24.43	No	ns	>0.9999
TNFa vs. TNFa	500 pg/mL vs. 2.5 ng/mL	49.29	No	ns	>0.9999
TNFa vs. TNFa	500 pg/mL vs. 5 ng/mL	77.71	No	ns	>0.9999
TNFa vs. TNFa	500 pg/mL vs. 10 ng/mL	87.21	No	ns	>0.9999
TNFa vs. Butyrate	500 pg/mL vs. 50 µM	-63	No	ns	>0.9999
TNFa vs. Butyrate	500 pg/mL vs. 75 µM	-65.57	No	ns	>0.9999
TNFa vs. Butyrate	500 pg/mL vs. 100 µM	-60.14	No	ns	>0.9999
TNFa vs. Butyrate	500 pg/mL vs. 250 µM	-20.14	No	ns	>0.9999
TNFa vs. Butyrate	500 pg/mL vs. 500 µM	-4	No	ns	>0.9999
TNFa vs. Butyrate	500 pg/mL vs. 750 µM	0.1429	No	ns	>0.9999
TNFa vs. Butyrate	500 pg/mL vs. 1 mM	-7	No	ns	>0.9999
TNFa vs. Butyrate	500 pg/mL vs. 2.5 mM	33.86	No	ns	>0.9999
TNFa vs. Butyrate	500 pg/mL vs. 5 mM	42.43	No	ns	>0.9999
TNFa vs. Butyrate	500 pg/mL vs. 7.5 mM	44.86	No	ns	>0.9999
TNFa vs. Butyrate	500 pg/mL vs. 10 mM	54.21	No	ns	>0.9999
TNFa vs. Butyrate	500 pg/mL vs. 25 mM	61.21	No	ns	>0.9999
TNFa vs. Combo	500 pg/mL vs. 50 pg/mL	-15.29	No	ns	>0.9999
TNFa vs. Combo	500 pg/mL vs. 100 pg/mL	4.714	No	ns	>0.9999
TNFa vs. Combo	500 pg/mL vs. 150 pg/mL	-3.286	No	ns	>0.9999
TNFa vs. Combo	500 pg/mL vs. 200 pg/mL	-0.9524	No	ns	>0.9999
TNFa vs. Combo	500 pg/mL vs. 250 pg/mL	-0.619	No	ns	>0.9999
TNFa vs. Combo	500 pg/mL vs. 500 pg/mL	16.38	No	ns	>0.9999
TNFa vs. Combo	500 pg/mL vs. 1 ng/mL	32.71	No	ns	>0.9999
TNFa vs. Combo	500 pg/mL vs. 2.5 ng/mL	71.38	No	ns	>0.9999
TNFa vs. Combo	500 pg/mL vs. 5 ng/mL	79.38	No	ns	>0.9999
TNFa vs. TNFa	1 ng/mL vs. 2.5 ng/mL	24.86	No	ns	>0.9999
TNFa vs. TNFa	1 ng/mL vs. 5 ng/mL	53.29	No	ns	>0.9999
TNFa vs. TNFa	1 ng/mL vs. 10 ng/mL	62.79	No	ns	>0.9999
TNFa vs. Butyrate	1 ng/mL vs. 50 µM	-87.43	No	ns	>0.9999
TNFa vs. Butyrate	1 ng/mL vs. 75 µM	-90	No	ns	0.879
TNFa vs. Butyrate	1 ng/mL vs. 100 µM	-84.57	No	ns	>0.9999

TNFa vs. Butyrate	1 ng/mL vs. 250 µM	-44.57	No	ns	>0.9999
TNFa vs. Butyrate	1 ng/mL vs. 500 µM	-28.43	No	ns	>0.9999
TNFa vs. Butyrate	1 ng/mL vs. 750 µM	-24.29	No	ns	>0.9999
TNFa vs. Butyrate	1 ng/mL vs. 1 mM	-31.43	No	ns	>0.9999
TNFa vs. Butyrate	1 ng/mL vs. 2.5 mM	9.429	No	ns	>0.9999
TNFa vs. Butyrate	1 ng/mL vs. 5 mM	18	No	ns	>0.9999
TNFa vs. Butyrate	1 ng/mL vs. 7.5 mM	20.43	No	ns	>0.9999
TNFa vs. Butyrate	1 ng/mL vs. 10 mM	29.79	No	ns	>0.9999
TNFa vs. Butyrate	1 ng/mL vs. 25 mM	36.79	No	ns	>0.9999
TNFa vs. Combo	1 ng/mL vs. 50 pg/mL	-39.71	No	ns	>0.9999
TNFa vs. Combo	1 ng/mL vs. 100 pg/mL	-19.71	No	ns	>0.9999
TNFa vs. Combo	1 ng/mL vs. 150 pg/mL	-27.71	No	ns	>0.9999
TNFa vs. Combo	1 ng/mL vs. 200 pg/mL	-25.38	No	ns	>0.9999
TNFa vs. Combo	1 ng/mL vs. 250 pg/mL	-25.05	No	ns	>0.9999
TNFa vs. Combo	1 ng/mL vs. 500 pg/mL	-8.048	No	ns	>0.9999
TNFa vs. Combo	1 ng/mL vs. 1 ng/mL	8.286	No	ns	>0.9999
TNFa vs. Combo	1 ng/mL vs. 2.5 ng/mL	46.95	No	ns	>0.9999
TNFa vs. Combo	1 ng/mL vs. 5 ng/mL	54.95	No	ns	>0.9999
TNFa vs. TNFa	2.5 ng/mL vs. 5 ng/mL	28.43	No	ns	>0.9999
TNFa vs. TNFa	2.5 ng/mL vs. 10 ng/mL	37.93	No	ns	>0.9999
TNFa vs. Butyrate	2.5 ng/mL vs. 50 µM	-112.3	Yes	*	0.0462
TNFa vs. Butyrate	2.5 ng/mL vs. 75 µM	-114.9	Yes	*	0.0317
TNFa vs. Butyrate	2.5 ng/mL vs. 100 µM	-109.4	No	ns	0.0696
TNFa vs. Butyrate	2.5 ng/mL vs. 250 µM	-69.43	No	ns	>0.9999
TNFa vs. Butyrate	2.5 ng/mL vs. 500 µM	-53.29	No	ns	>0.9999
TNFa vs. Butyrate	2.5 ng/mL vs. 750 µM	-49.14	No	ns	>0.9999
TNFa vs. Butyrate	2.5 ng/mL vs. 1 mM	-56.29	No	ns	>0.9999
TNFa vs. Butyrate	2.5 ng/mL vs. 2.5 mM	-15.43	No	ns	>0.9999
TNFa vs. Butyrate	2.5 ng/mL vs. 5 mM	-6.857	No	ns	>0.9999
TNFa vs. Butyrate	2.5 ng/mL vs. 7.5 mM	-4.429	No	ns	>0.9999
TNFa vs. Butyrate	2.5 ng/mL vs. 10 mM	4.929	No	ns	>0.9999
TNFa vs. Butyrate	2.5 ng/mL vs. 25 mM	11.93	No	ns	>0.9999
TNFa vs. Combo	2.5 ng/mL vs. 50 pg/mL	-64.57	No	ns	>0.9999
TNFa vs. Combo	2.5 ng/mL vs. 100 pg/mL	-44.57	No	ns	>0.9999
TNFa vs. Combo	2.5 ng/mL vs. 150 pg/mL	-52.57	No	ns	>0.9999
TNFa vs. Combo	2.5 ng/mL vs. 200 pg/mL	-50.24	No	ns	>0.9999
TNFa vs. Combo	2.5 ng/mL vs. 250 pg/mL	-49.9	No	ns	>0.9999
TNFa vs. Combo	2.5 ng/mL vs. 500 pg/mL	-32.9	No	ns	>0.9999
TNFa vs. Combo	2.5 ng/mL vs. 1 ng/mL	-16.57	No	ns	>0.9999
TNFa vs. Combo	2.5 ng/mL vs. 2.5 ng/mL	22.1	No	ns	>0.9999
TNFa vs. Combo	2.5 ng/mL vs. 5 ng/mL	30.1	No	ns	>0.9999
TNFa vs. TNFa	5 ng/mL vs. 10 ng/mL	9.5	No	ns	>0.9999
TNFa vs. Butyrate	5 ng/mL vs. 50 µM	-140.7	Yes	***	0.0005
TNFa vs. Butyrate	5 ng/mL vs. 75 µM	-143.3	Yes	***	0.0003
TNFa vs. Butyrate	5 ng/mL vs. 100 µM	-137.9	Yes	***	0.0008
TNFa vs. Butyrate	5 ng/mL vs. 250 µM	-97.86	No	ns	0.3321
TNFa vs. Butyrate	5 ng/mL vs. 500 µM	-81.71	No	ns	>0.9999

TNFa vs. Butyrate	5 ng/mL vs. 750 µM	-77.57	No	ns	>0.9999
TNFa vs. Butyrate	5 ng/mL vs. 1 mM	-84.71	No	ns	>0.9999
TNFa vs. Butyrate	5 ng/mL vs. 2.5 mM	-43.86	No	ns	>0.9999
TNFa vs. Butyrate	5 ng/mL vs. 5 mM	-35.29	No	ns	>0.9999
TNFa vs. Butyrate	5 ng/mL vs. 7.5 mM	-32.86	No	ns	>0.9999
TNFa vs. Butyrate	5 ng/mL vs. 10 mM	-23.5	No	ns	>0.9999
TNFa vs. Butyrate	5 ng/mL vs. 25 mM	-16.5	No	ns	>0.9999
TNFa vs. Combo	5 ng/mL vs. 50 pg/mL	-93	No	ns	>0.9999
TNFa vs. Combo	5 ng/mL vs. 100 pg/mL	-73	No	ns	>0.9999
TNFa vs. Combo	5 ng/mL vs. 150 pg/mL	-81	No	ns	>0.9999
TNFa vs. Combo	5 ng/mL vs. 200 pg/mL	-78.67	No	ns	>0.9999
TNFa vs. Combo	5 ng/mL vs. 250 pg/mL	-78.33	No	ns	>0.9999
TNFa vs. Combo	5 ng/mL vs. 500 pg/mL	-61.33	No	ns	>0.9999
TNFa vs. Combo	5 ng/mL vs. 1 ng/mL	-45	No	ns	>0.9999
TNFa vs. Combo	5 ng/mL vs. 2.5 ng/mL	-6.333	No	ns	>0.9999
TNFa vs. Combo	5 ng/mL vs. 5 ng/mL	1.667	No	ns	>0.9999
TNFa vs. Butyrate	10 ng/mL vs. 50 µM	-150.2	Yes	**	0.004
TNFa vs. Butyrate	10 ng/mL vs. 75 µM	-152.8	Yes	**	0.0028
TNFa vs. Butyrate	10 ng/mL vs. 100 µM	-147.4	Yes	**	0.006
TNFa vs. Butyrate	10 ng/mL vs. 250 µM	-107.4	No	ns	0.7291
TNFa vs. Butyrate	10 ng/mL vs. 500 µM	-91.21	No	ns	>0.9999
TNFa vs. Butyrate	10 ng/mL vs. 750 µM	-87.07	No	ns	>0.9999
TNFa vs. Butyrate	10 ng/mL vs. 1 mM	-94.21	No	ns	>0.9999
TNFa vs. Butyrate	10 ng/mL vs. 2.5 mM	-53.36	No	ns	>0.9999
TNFa vs. Butyrate	10 ng/mL vs. 5 mM	-44.79	No	ns	>0.9999
TNFa vs. Butyrate	10 ng/mL vs. 7.5 mM	-42.36	No	ns	>0.9999
TNFa vs. Butyrate	10 ng/mL vs. 10 mM	-33	No	ns	>0.9999
TNFa vs. Butyrate	10 ng/mL vs. 25 mM	-26	No	ns	>0.9999
TNFa vs. Combo	10 ng/mL vs. 50 pg/mL	-102.5	No	ns	>0.9999
TNFa vs. Combo	10 ng/mL vs. 100 pg/mL	-82.5	No	ns	>0.9999
TNFa vs. Combo	10 ng/mL vs. 150 pg/mL	-90.5	No	ns	>0.9999
TNFa vs. Combo	10 ng/mL vs. 200 pg/mL	-88.17	No	ns	>0.9999
TNFa vs. Combo	10 ng/mL vs. 250 pg/mL	-87.83	No	ns	>0.9999
TNFa vs. Combo	10 ng/mL vs. 500 pg/mL	-70.83	No	ns	>0.9999
TNFa vs. Combo	10 ng/mL vs. 1 ng/mL	-54.5	No	ns	>0.9999
TNFa vs. Combo	10 ng/mL vs. 2.5 ng/mL	-15.83	No	ns	>0.9999
TNFa vs. Combo	10 ng/mL vs. 5 ng/mL	-7.833	No	ns	>0.9999
Butyrate vs. Butyrate	50 µM vs. 75 µM	-2.571	No	ns	>0.9999
Butyrate vs. Butyrate	50 µM vs. 100 µM	2.857	No	ns	>0.9999
Butyrate vs. Butyrate	50 µM vs. 250 µM	42.86	No	ns	>0.9999
Butyrate vs. Butyrate	50 µM vs. 500 µM	59	No	ns	>0.9999
Butyrate vs. Butyrate	50 µM vs. 750 µM	63.14	No	ns	>0.9999
Butyrate vs. Butyrate	50 µM vs. 1 mM	56	No	ns	>0.9999
Butyrate vs. Butyrate	50 µM vs. 2.5 mM	96.86	No	ns	0.3774
Butyrate vs. Butyrate	50 µM vs. 5 mM	105.4	No	ns	0.1215
Butyrate vs. Butyrate	50 µM vs. 7.5 mM	107.9	No	ns	0.0868
Butyrate vs. Butyrate	50 µM vs. 10 mM	117.2	No	ns	0.2528

Butyrate vs. Butyrate	50 µM vs. 25 mM	124.2	No	ns	0.1135
Butyrate vs. Combo	50 µM vs. 50 pg/mL	47.71	No	ns	>0.9999
Butyrate vs. Combo	50 µM vs. 100 pg/mL	67.71	No	ns	>0.9999
Butyrate vs. Combo	50 µM vs. 150 pg/mL	59.71	No	ns	>0.9999
Butyrate vs. Combo	50 µM vs. 200 pg/mL	62.05	No	ns	>0.9999
Butyrate vs. Combo	50 µM vs. 250 pg/mL	62.38	No	ns	>0.9999
Butyrate vs. Combo	50 µM vs. 500 pg/mL	79.38	No	ns	>0.9999
Butyrate vs. Combo	50 µM vs. 1 ng/mL	95.71	No	ns	>0.9999
Butyrate vs. Combo	50 µM vs. 2.5 ng/mL	134.4	No	ns	0.1459
Butyrate vs. Combo	50 µM vs. 5 ng/mL	142.4	No	ns	0.0616
Butyrate vs. Butyrate	75 µM vs. 100 µM	5.429	No	ns	>0.9999
Butyrate vs. Butyrate	75 µM vs. 250 µM	45.43	No	ns	>0.9999
Butyrate vs. Butyrate	75 µM vs. 500 µM	61.57	No	ns	>0.9999
Butyrate vs. Butyrate	75 µM vs. 750 µM	65.71	No	ns	>0.9999
Butyrate vs. Butyrate	75 µM vs. 1 mM	58.57	No	ns	>0.9999
Butyrate vs. Butyrate	75 µM vs. 2.5 mM	99.43	No	ns	0.271
Butyrate vs. Butyrate	75 µM vs. 5 mM	108	No	ns	0.0851
Butyrate vs. Butyrate	75 µM vs. 7.5 mM	110.4	No	ns	0.0604
Butyrate vs. Butyrate	75 µM vs. 10 mM	119.8	No	ns	0.1893
Butyrate vs. Butyrate	75 µM vs. 25 mM	126.8	No	ns	0.0837
Butyrate vs. Combo	75 µM vs. 50 pg/mL	50.29	No	ns	>0.9999
Butyrate vs. Combo	75 µM vs. 100 pg/mL	70.29	No	ns	>0.9999
Butyrate vs. Combo	75 µM vs. 150 pg/mL	62.29	No	ns	>0.9999
Butyrate vs. Combo	75 µM vs. 200 pg/mL	64.62	No	ns	>0.9999
Butyrate vs. Combo	75 µM vs. 250 pg/mL	64.95	No	ns	>0.9999
Butyrate vs. Combo	75 µM vs. 500 pg/mL	81.95	No	ns	>0.9999
Butyrate vs. Combo	75 µM vs. 1 ng/mL	98.29	No	ns	>0.9999
Butyrate vs. Combo	75 µM vs. 2.5 ng/mL	137	No	ns	0.1111
Butyrate vs. Combo	75 µM vs. 5 ng/mL	145	Yes	*	0.0462
Butyrate vs. Butyrate	100 µM vs. 250 µM	40	No	ns	>0.9999
Butyrate vs. Butyrate	100 µM vs. 500 µM	56.14	No	ns	>0.9999
Butyrate vs. Butyrate	100 µM vs. 750 µM	60.29	No	ns	>0.9999
Butyrate vs. Butyrate	100 µM vs. 1 mM	53.14	No	ns	>0.9999
Butyrate vs. Butyrate	100 µM vs. 2.5 mM	94	No	ns	0.5403
Butyrate vs. Butyrate	100 µM vs. 5 mM	102.6	No	ns	0.179
Butyrate vs. Butyrate	100 µM vs. 7.5 mM	105	No	ns	0.1289
Butyrate vs. Butyrate	100 µM vs. 10 mM	114.4	No	ns	0.3466
Butyrate vs. Butyrate	100 µM vs. 25 mM	121.4	No	ns	0.1581
Butyrate vs. Combo	100 µM vs. 50 pg/mL	44.86	No	ns	>0.9999
Butyrate vs. Combo	100 µM vs. 100 pg/mL	64.86	No	ns	>0.9999
Butyrate vs. Combo	100 µM vs. 150 pg/mL	56.86	No	ns	>0.9999
Butyrate vs. Combo	100 µM vs. 200 pg/mL	59.19	No	ns	>0.9999
Butyrate vs. Combo	100 µM vs. 250 pg/mL	59.52	No	ns	>0.9999
Butyrate vs. Combo	100 µM vs. 500 pg/mL	76.52	No	ns	>0.9999
Butyrate vs. Combo	100 µM vs. 1 ng/mL	92.86	No	ns	>0.9999
Butyrate vs. Combo	100 µM vs. 2.5 ng/mL	131.5	No	ns	0.1963
Butyrate vs. Combo	100 µM vs. 5 ng/mL	139.5	No	ns	0.0842

Butyrate vs. Butyrate	250 µM vs. 500 µM	16.14	No	ns	>0.9999
Butyrate vs. Butyrate	250 µM vs. 750 µM	20.29	No	ns	>0.9999
Butyrate vs. Butyrate	250 µM vs. 1 mM	13.14	No	ns	>0.9999
Butyrate vs. Butyrate	250 µM vs. 2.5 mM	54	No	ns	>0.9999
Butyrate vs. Butyrate	250 µM vs. 5 mM	62.57	No	ns	>0.9999
Butyrate vs. Butyrate	250 µM vs. 7.5 mM	65	No	ns	>0.9999
Butyrate vs. Butyrate	250 µM vs. 10 mM	74.36	No	ns	>0.9999
Butyrate vs. Butyrate	250 µM vs. 25 mM	81.36	No	ns	>0.9999
Butyrate vs. Combo	250 µM vs. 50 pg/mL	4.857	No	ns	>0.9999
Butyrate vs. Combo	250 µM vs. 100 pg/mL	24.86	No	ns	>0.9999
Butyrate vs. Combo	250 µM vs. 150 pg/mL	16.86	No	ns	>0.9999
Butyrate vs. Combo	250 µM vs. 200 pg/mL	19.19	No	ns	>0.9999
Butyrate vs. Combo	250 µM vs. 250 pg/mL	19.52	No	ns	>0.9999
Butyrate vs. Combo	250 µM vs. 500 pg/mL	36.52	No	ns	>0.9999
Butyrate vs. Combo	250 µM vs. 1 ng/mL	52.86	No	ns	>0.9999
Butyrate vs. Combo	250 µM vs. 2.5 ng/mL	91.52	No	ns	>0.9999
Butyrate vs. Combo	250 µM vs. 5 ng/mL	99.52	No	ns	>0.9999
Butyrate vs. Butyrate	500 µM vs. 750 µM	4.143	No	ns	>0.9999
Butyrate vs. Butyrate	500 µM vs. 1 mM	-3	No	ns	>0.9999
Butyrate vs. Butyrate	500 µM vs. 2.5 mM	37.86	No	ns	>0.9999
Butyrate vs. Butyrate	500 µM vs. 5 mM	46.43	No	ns	>0.9999
Butyrate vs. Butyrate	500 µM vs. 7.5 mM	48.86	No	ns	>0.9999
Butyrate vs. Butyrate	500 µM vs. 10 mM	58.21	No	ns	>0.9999
Butyrate vs. Butyrate	500 µM vs. 25 mM	65.21	No	ns	>0.9999
Butyrate vs. Combo	500 µM vs. 50 pg/mL	-11.29	No	ns	>0.9999
Butyrate vs. Combo	500 µM vs. 100 pg/mL	8.714	No	ns	>0.9999
Butyrate vs. Combo	500 µM vs. 150 pg/mL	0.7143	No	ns	>0.9999
Butyrate vs. Combo	500 µM vs. 200 pg/mL	3.048	No	ns	>0.9999
Butyrate vs. Combo	500 µM vs. 250 pg/mL	3.381	No	ns	>0.9999
Butyrate vs. Combo	500 µM vs. 500 pg/mL	20.38	No	ns	>0.9999
Butyrate vs. Combo	500 µM vs. 1 ng/mL	36.71	No	ns	>0.9999
Butyrate vs. Combo	500 µM vs. 2.5 ng/mL	75.38	No	ns	>0.9999
Butyrate vs. Combo	500 µM vs. 5 ng/mL	83.38	No	ns	>0.9999
Butyrate vs. Butyrate	750 µM vs. 1 mM	-7.143	No	ns	>0.9999
Butyrate vs. Butyrate	750 µM vs. 2.5 mM	33.71	No	ns	>0.9999
Butyrate vs. Butyrate	750 µM vs. 5 mM	42.29	No	ns	>0.9999
Butyrate vs. Butyrate	750 µM vs. 7.5 mM	44.71	No	ns	>0.9999
Butyrate vs. Butyrate	750 µM vs. 10 mM	54.07	No	ns	>0.9999
Butyrate vs. Butyrate	750 µM vs. 25 mM	61.07	No	ns	>0.9999
Butyrate vs. Combo	750 µM vs. 50 pg/mL	-15.43	No	ns	>0.9999
Butyrate vs. Combo	750 µM vs. 100 pg/mL	4.571	No	ns	>0.9999
Butyrate vs. Combo	750 µM vs. 150 pg/mL	-3.429	No	ns	>0.9999
Butyrate vs. Combo	750 µM vs. 200 pg/mL	-1.095	No	ns	>0.9999
Butyrate vs. Combo	750 µM vs. 250 pg/mL	-0.7619	No	ns	>0.9999
Butyrate vs. Combo	750 µM vs. 500 pg/mL	16.24	No	ns	>0.9999
Butyrate vs. Combo	750 µM vs. 1 ng/mL	32.57	No	ns	>0.9999
Butyrate vs. Combo	750 µM vs. 2.5 ng/mL	71.24	No	ns	>0.9999

Butyrate vs. Combo	750 µM vs. 5 ng/mL	79.24	No	ns	>0.9999
Butyrate vs. Butyrate	1 mM vs. 2.5 mM	40.86	No	ns	>0.9999
Butyrate vs. Butyrate	1 mM vs. 5 mM	49.43	No	ns	>0.9999
Butyrate vs. Butyrate	1 mM vs. 7.5 mM	51.86	No	ns	>0.9999
Butyrate vs. Butyrate	1 mM vs. 10 mM	61.21	No	ns	>0.9999
Butyrate vs. Butyrate	1 mM vs. 25 mM	68.21	No	ns	>0.9999
Butyrate vs. Combo	1 mM vs. 50 pg/mL	-8.286	No	ns	>0.9999
Butyrate vs. Combo	1 mM vs. 100 pg/mL	11.71	No	ns	>0.9999
Butyrate vs. Combo	1 mM vs. 150 pg/mL	3.714	No	ns	>0.9999
Butyrate vs. Combo	1 mM vs. 200 pg/mL	6.048	No	ns	>0.9999
Butyrate vs. Combo	1 mM vs. 250 pg/mL	6.381	No	ns	>0.9999
Butyrate vs. Combo	1 mM vs. 500 pg/mL	23.38	No	ns	>0.9999
Butyrate vs. Combo	1 mM vs. 1 ng/mL	39.71	No	ns	>0.9999
Butyrate vs. Combo	1 mM vs. 2.5 ng/mL	78.38	No	ns	>0.9999
Butyrate vs. Combo	1 mM vs. 5 ng/mL	86.38	No	ns	>0.9999
Butyrate vs. Butyrate	2.5 mM vs. 5 mM	8.571	No	ns	>0.9999
Butyrate vs. Butyrate	2.5 mM vs. 7.5 mM	11	No	ns	>0.9999
Butyrate vs. Butyrate	2.5 mM vs. 10 mM	20.36	No	ns	>0.9999
Butyrate vs. Butyrate	2.5 mM vs. 25 mM	27.36	No	ns	>0.9999
Butyrate vs. Combo	2.5 mM vs. 50 pg/mL	-49.14	No	ns	>0.9999
Butyrate vs. Combo	2.5 mM vs. 100 pg/mL	-29.14	No	ns	>0.9999
Butyrate vs. Combo	2.5 mM vs. 150 pg/mL	-37.14	No	ns	>0.9999
Butyrate vs. Combo	2.5 mM vs. 200 pg/mL	-34.81	No	ns	>0.9999
Butyrate vs. Combo	2.5 mM vs. 250 pg/mL	-34.48	No	ns	>0.9999
Butyrate vs. Combo	2.5 mM vs. 500 pg/mL	-17.48	No	ns	>0.9999
Butyrate vs. Combo	2.5 mM vs. 1 ng/mL	-1.143	No	ns	>0.9999
Butyrate vs. Combo	2.5 mM vs. 2.5 ng/mL	37.52	No	ns	>0.9999
Butyrate vs. Combo	2.5 mM vs. 5 ng/mL	45.52	No	ns	>0.9999
Butyrate vs. Butyrate	5 mM vs. 7.5 mM	2.429	No	ns	>0.9999
Butyrate vs. Butyrate	5 mM vs. 10 mM	11.79	No	ns	>0.9999
Butyrate vs. Butyrate	5 mM vs. 25 mM	18.79	No	ns	>0.9999
Butyrate vs. Combo	5 mM vs. 50 pg/mL	-57.71	No	ns	>0.9999
Butyrate vs. Combo	5 mM vs. 100 pg/mL	-37.71	No	ns	>0.9999
Butyrate vs. Combo	5 mM vs. 150 pg/mL	-45.71	No	ns	>0.9999
Butyrate vs. Combo	5 mM vs. 200 pg/mL	-43.38	No	ns	>0.9999
Butyrate vs. Combo	5 mM vs. 250 pg/mL	-43.05	No	ns	>0.9999
Butyrate vs. Combo	5 mM vs. 500 pg/mL	-26.05	No	ns	>0.9999
Butyrate vs. Combo	5 mM vs. 1 ng/mL	-9.714	No	ns	>0.9999
Butyrate vs. Combo	5 mM vs. 2.5 ng/mL	28.95	No	ns	>0.9999
Butyrate vs. Combo	5 mM vs. 5 ng/mL	36.95	No	ns	>0.9999
Butyrate vs. Butyrate	7.5 mM vs. 10 mM	9.357	No	ns	>0.9999
Butyrate vs. Butyrate	7.5 mM vs. 25 mM	16.36	No	ns	>0.9999
Butyrate vs. Combo	7.5 mM vs. 50 pg/mL	-60.14	No	ns	>0.9999
Butyrate vs. Combo	7.5 mM vs. 100 pg/mL	-40.14	No	ns	>0.9999
Butyrate vs. Combo	7.5 mM vs. 150 pg/mL	-48.14	No	ns	>0.9999
Butyrate vs. Combo	7.5 mM vs. 200 pg/mL	-45.81	No	ns	>0.9999
Butyrate vs. Combo	7.5 mM vs. 250 pg/mL	-45.48	No	ns	>0.9999

Butyrate vs. Combo	7.5 mM vs. 500 pg/mL	-28.48	No	ns	>0.9999
Butyrate vs. Combo	7.5 mM vs. 1 ng/mL	-12.14	No	ns	>0.9999
Butyrate vs. Combo	7.5 mM vs. 2.5 ng/mL	26.52	No	ns	>0.9999
Butyrate vs. Combo	7.5 mM vs. 5 ng/mL	34.52	No	ns	>0.9999
Butyrate vs. Butyrate	10 mM vs. 25 mM	7	No	ns	>0.9999
Butyrate vs. Combo	10 mM vs. 50 pg/mL	-69.5	No	ns	>0.9999
Butyrate vs. Combo	10 mM vs. 100 pg/mL	-49.5	No	ns	>0.9999
Butyrate vs. Combo	10 mM vs. 150 pg/mL	-57.5	No	ns	>0.9999
Butyrate vs. Combo	10 mM vs. 200 pg/mL	-55.17	No	ns	>0.9999
Butyrate vs. Combo	10 mM vs. 250 pg/mL	-54.83	No	ns	>0.9999
Butyrate vs. Combo	10 mM vs. 500 pg/mL	-37.83	No	ns	>0.9999
Butyrate vs. Combo	10 mM vs. 1 ng/mL	-21.5	No	ns	>0.9999
Butyrate vs. Combo	10 mM vs. 2.5 ng/mL	17.17	No	ns	>0.9999
Butyrate vs. Combo	10 mM vs. 5 ng/mL	25.17	No	ns	>0.9999
Butyrate vs. Combo	25 mM vs. 50 pg/mL	-76.5	No	ns	>0.9999
Butyrate vs. Combo	25 mM vs. 100 pg/mL	-56.5	No	ns	>0.9999
Butyrate vs. Combo	25 mM vs. 150 pg/mL	-64.5	No	ns	>0.9999
Butyrate vs. Combo	25 mM vs. 200 pg/mL	-62.17	No	ns	>0.9999
Butyrate vs. Combo	25 mM vs. 250 pg/mL	-61.83	No	ns	>0.9999
Butyrate vs. Combo	25 mM vs. 500 pg/mL	-44.83	No	ns	>0.9999
Butyrate vs. Combo	25 mM vs. 1 ng/mL	-28.5	No	ns	>0.9999
Butyrate vs. Combo	25 mM vs. 2.5 ng/mL	10.17	No	ns	>0.9999
Butyrate vs. Combo	25 mM vs. 5 ng/mL	18.17	No	ns	>0.9999
Combo vs. Combo	50 pg/mL vs. 100 pg/mL	20	No	ns	>0.9999
Combo vs. Combo	50 pg/mL vs. 150 pg/mL	12	No	ns	>0.9999
Combo vs. Combo	50 pg/mL vs. 200 pg/mL	14.33	No	ns	>0.9999
Combo vs. Combo	50 pg/mL vs. 250 pg/mL	14.67	No	ns	>0.9999
Combo vs. Combo	50 pg/mL vs. 500 pg/mL	31.67	No	ns	>0.9999
Combo vs. Combo	50 pg/mL vs. 1 ng/mL	48	No	ns	>0.9999
Combo vs. Combo	50 pg/mL vs. 2.5 ng/mL	86.67	No	ns	>0.9999
Combo vs. Combo	50 pg/mL vs. 5 ng/mL	94.67	No	ns	>0.9999
Combo vs. Combo	100 pg/mL vs. 150 pg/mL	-8	No	ns	>0.9999
Combo vs. Combo	100 pg/mL vs. 200 pg/mL	-5.667	No	ns	>0.9999
Combo vs. Combo	100 pg/mL vs. 250 pg/mL	-5.333	No	ns	>0.9999
Combo vs. Combo	100 pg/mL vs. 500 pg/mL	11.67	No	ns	>0.9999
Combo vs. Combo	100 pg/mL vs. 1 ng/mL	28	No	ns	>0.9999
Combo vs. Combo	100 pg/mL vs. 2.5 ng/mL	66.67	No	ns	>0.9999
Combo vs. Combo	100 pg/mL vs. 5 ng/mL	74.67	No	ns	>0.9999
Combo vs. Combo	150 pg/mL vs. 200 pg/mL	2.333	No	ns	>0.9999
Combo vs. Combo	150 pg/mL vs. 250 pg/mL	2.667	No	ns	>0.9999
Combo vs. Combo	150 pg/mL vs. 500 pg/mL	19.67	No	ns	>0.9999
Combo vs. Combo	150 pg/mL vs. 1 ng/mL	36	No	ns	>0.9999
Combo vs. Combo	150 pg/mL vs. 2.5 ng/mL	74.67	No	ns	>0.9999
Combo vs. Combo	150 pg/mL vs. 5 ng/mL	82.67	No	ns	>0.9999
Combo vs. Combo	200 pg/mL vs. 250 pg/mL	0.3333	No	ns	>0.9999
Combo vs. Combo	200 pg/mL vs. 500 pg/mL	17.33	No	ns	>0.9999
Combo vs. Combo	200 pg/mL vs. 1 ng/mL	33.67	No	ns	>0.9999

Combo vs. Combo	200 pg/mL vs. 2.5 ng/mL	72.33	No	ns	>0.9999
Combo vs. Combo	200 pg/mL vs. 5 ng/mL	80.33	No	ns	>0.9999
Combo vs. Combo	250 pg/mL vs. 500 pg/mL	17	No	ns	>0.9999
Combo vs. Combo	250 pg/mL vs. 1 ng/mL	33.33	No	ns	>0.9999
Combo vs. Combo	250 pg/mL vs. 2.5 ng/mL	72	No	ns	>0.9999
Combo vs. Combo	250 pg/mL vs. 5 ng/mL	80	No	ns	>0.9999
Combo vs. Combo	500 pg/mL vs. 1 ng/mL	16.33	No	ns	>0.9999
Combo vs. Combo	500 pg/mL vs. 2.5 ng/mL	55	No	ns	>0.9999
Combo vs. Combo	500 pg/mL vs. 5 ng/mL	63	No	ns	>0.9999
Combo vs. Combo	1 ng/mL vs. 2.5 ng/mL	38.67	No	ns	>0.9999
Combo vs. Combo	1 ng/mL vs. 5 ng/mL	46.67	No	ns	>0.9999
Combo vs. Combo	2.5 ng/mL vs. 5 ng/mL				

Value
A-B
A-C
A-D
A-E
A-F
A-G
A-H
A-I
A-J
A-K
A-L
A-M
A-N
A-O
A-P
A-Q
A-R
A-S
A-T
A-U
A-V
A-W
A-X
A-Y
A-Z
A-AA
A-AB
A-AC
A-AD
A-AE
A-AF
A-AG
B-C
B-D
B-E
B-F
B-G
B-H
B-I
B-J
B-K
B-L
B-M
B-N
B-O
B-P

B-Q
B-R
B-S
B-T
B-U
B-V
B-W
B-X
B-Y
B-Z
B-AA
B-AB
B-AC
B-AD
B-AE
B-AF
B-AG
C-D
C-E
C-F
C-G
C-H
C-I
C-J
C-K
C-L
C-M
C-N
C-O
C-P
C-Q
C-R
C-S
C-T
C-U
C-V
C-W
C-X
C-Y
C-Z
C-AA
C-AB
C-AC
C-AD
C-AE
C-AF
C-AG

D-E
D-F
D-G
D-H
D-I
D-J
D-K
D-L
D-M
D-N
D-O
D-P
D-Q
D-R
D-S
D-T
D-U
D-V
D-W
D-X
D-Y
D-Z
D-AA
D-AB
D-AC
D-AD
D-AE
D-AF
D-AG
E-F
E-G
E-H
E-I
E-J
E-K
E-L
E-M
E-N
E-O
E-P
E-Q
E-R
E-S
E-T
E-U
E-V
E-W

E-X
E-Y
E-Z
E-AA
E-AB
E-AC
E-AD
E-AE
E-AF
E-AG
F-G
F-H
F-I
F-J
F-K
F-L
F-M
F-N
F-O
F-P
F-Q
F-R
F-S
F-T
F-U
F-V
F-W
F-X
F-Y
F-Z
F-AA
F-AB
F-AC
F-AD
F-AE
F-AF
F-AG
G-H
G-I
G-J
G-K
G-L
G-M
G-N
G-O
G-P
G-Q

G-R
G-S
G-T
G-U
G-V
G-W
G-X
G-Y
G-Z
G-AA
G-AB
G-AC
G-AD
G-AE
G-AF
G-AG
H-I
H-J
H-K
H-L
H-M
H-N
H-O
H-P
H-Q
H-R
H-S
H-T
H-U
H-V
H-W
H-X
H-Y
H-Z
H-AA
H-AB
H-AC
H-AD
H-AE
H-AF
H-AG
I-J
I-K
I-L
I-M
I-N
I-O

I-P
I-Q
I-R
I-S
I-T
I-U
I-V
I-W
I-X
I-Y
I-Z
I-AA
I-AB
I-AC
I-AD
I-AE
I-AF
I-AG
J-K
J-L
J-M
J-N
J-O
J-P
J-Q
J-R
J-S
J-T
J-U
J-V
J-W
J-X
J-Y
J-Z
J-AA
J-AB
J-AC
J-AD
J-AE
J-AF
J-AG
K-L
K-M
K-N
K-O
K-P
K-Q

K-R
K-S
K-T
K-U
K-V
K-W
K-X
K-Y
K-Z
K-AA
K-AB
K-AC
K-AD
K-AE
K-AF
K-AG
L-M
L-N
L-O
L-P
L-Q
L-R
L-S
L-T
L-U
L-V
L-W
L-X
L-Y
L-Z
L-AA
L-AB
L-AC
L-AD
L-AE
L-AF
L-AG
M-N
M-O
M-P
M-Q
M-R
M-S
M-T
M-U
M-V
M-W

M-X
M-Y
M-Z
M-AA
M-AB
M-AC
M-AD
M-AE
M-AF
M-AG
N-O
N-P
N-Q
N-R
N-S
N-T
N-U
N-V
N-W
N-X
N-Y
N-Z
N-AA
N-AB
N-AC
N-AD
N-AE
N-AF
N-AG
O-P
O-Q
O-R
O-S
O-T
O-U
O-V
O-W
O-X
O-Y
O-Z
O-AA
O-AB
O-AC
O-AD
O-AE
O-AF
O-AG

P-Q
P-R
P-S
P-T
P-U
P-V
P-W
P-X
P-Y
P-Z
P-AA
P-AB
P-AC
P-AD
P-AE
P-AF
P-AG
Q-R
Q-S
Q-T
Q-U
Q-V
Q-W
Q-X
Q-Y
Q-Z
Q-AA
Q-AB
Q-AC
Q-AD
Q-AE
Q-AF
Q-AG
R-S
R-T
R-U
R-V
R-W
R-X
R-Y
R-Z
R-AA
R-AB
R-AC
R-AD
R-AE
R-AF

R-AG
S-T
S-U
S-V
S-W
S-X
S-Y
S-Z
S-AA
S-AB
S-AC
S-AD
S-AE
S-AF
S-AG
T-U
T-V
T-W
T-X
T-Y
T-Z
T-AA
T-AB
T-AC
T-AD
T-AE
T-AF
T-AG
U-V
U-W
U-X
U-Y
U-Z
U-AA
U-AB
U-AC
U-AD
U-AE
U-AF
U-AG
V-W
V-X
V-Y
V-Z
V-AA
V-AB
V-AC

V-AD
V-AE
V-AF
V-AG
W-X
W-Y
W-Z
W-AA
W-AB
W-AC
W-AD
W-AE
W-AF
W-AG
X-Y
X-Z
X-AA
X-AB
X-AC
X-AD
X-AE
X-AF
X-AG
Y-Z
Y-AA
Y-AB
Y-AC
Y-AD
Y-AE
Y-AF
Y-AG
Z-AA
Z-AB
Z-AC
Z-AD
Z-AE
Z-AF
Z-AG
AA-AB
AA-AC
AA-AD
AA-AE
AA-AF
AA-AG
AB-AC
AB-AD
AB-AE

AB-AF
AB-AG
AC-AD
AC-AE
AC-AF
AC-AG
AD-AE
AD-AF
AD-AG
AE-AF
AE-AG

Treatment	Dunn's multiple comparisons test	Mean rank	Significant′	Summary
Media Vs. FCCP	Media vs. 10 µM FCCP	39.3	No	ns
Media vs. TNFa	Media vs. 50 pg/mL	3.8	No	ns
Media vs. TNFa	Media vs. 100 pg/mL	10.47	No	ns
Media vs. TNFa	Media vs. 150 pg/mL	1.467	No	ns
Media vs. TNFa	Media vs. 200 pg/mL	15.47	No	ns
Media vs. TNFa	Media vs. 250 pg/mL	12.13	No	ns
Media vs. TNFa	Media vs. 500 pg/mL	19.13	No	ns
Media vs. TNFa	Media vs. 1 ng/mL	17.47	No	ns
Media vs. TNFa	Media vs. 2.5 ng/mL	33.13	No	ns
Media vs. TNFa	Media vs. 5 ng/mL	19.47	No	ns
Media vs. Butyrate	Media vs. 50 µM	-30.2	No	ns
Media vs. Butyrate	Media vs. 75 µM	-31.87	No	ns
Media vs. Butyrate	Media vs. 100 µM	-22.87	No	ns
Media vs. Butyrate	Media vs. 250 µM	0.1333	No	ns
Media vs. Butyrate	Media vs. 500 µM	-7.2	No	ns
Media vs. Butyrate	Media vs. 750 µM	-17.2	No	ns
Media vs. Butyrate	Media vs. 1 mM	-22.2	No	ns
Media vs. Butyrate	Media vs. 2.5 mM	-28.2	No	ns
Media vs. Butyrate	Media vs. 5 mM	0.4667	No	ns
Media vs. Butyrate	Media vs. 7.5 mM	-0.2	No	ns
Media Vs. Combo	Media vs. 50 pg/mL	-32.87	No	ns
Media Vs. Combo	Media vs. 100 pg/mL	7.467	No	ns
Media Vs. Combo	Media vs. 150 pg/mL	-14.7	No	ns
Media Vs. Combo	Media vs. 200 pg/mL	-22.7	No	ns
Media Vs. Combo	Media vs. 250 pg/mL	-33.2	No	ns
Media Vs. Combo	Media vs. 500 pg/mL	-42.53	No	ns
Media Vs. Combo	Media vs. 1 ng/mL	-28.2	No	ns
Media Vs. Combo	Media vs. 2.5 ng/mL	-17.53	No	ns
Media Vs. Combo	Media vs. 5 ng/mL	-4.533	No	ns
FCCp vs. TNFa	10 µM FCCP vs. 50 pg/mL	-35.5	No	ns
FCCp vs. TNFa	10 µM FCCP vs. 100 pg/mL	-28.83	No	ns
FCCp vs. TNFa	10 µM FCCP vs. 150 pg/mL	-37.83	No	ns
FCCp vs. TNFa	10 µM FCCP vs. 200 pg/mL	-23.83	No	ns
FCCp vs. TNFa	10 µM FCCP vs. 250 pg/mL	-27.17	No	ns
FCCp vs. TNFa	10 µM FCCP vs. 500 pg/mL	-20.17	No	ns
FCCp vs. TNFa	10 µM FCCP vs. 1 ng/mL	-21.83	No	ns
FCCp vs. TNFa	10 µM FCCP vs. 2.5 ng/mL	-6.167	No	ns
FCCp vs. TNFa	10 µM FCCP vs. 5 ng/mL	-19.83	No	ns
FCCp vs. Butyrate	10 µM FCCP vs. 50 µM	-69.5	No	ns
FCCp vs. Butyrate	10 µM FCCP vs. 75 µM	-71.17	No	ns
FCCp vs. Butyrate	10 µM FCCP vs. 100 µM	-62.17	No	ns
FCCp vs. Butyrate	10 µM FCCP vs. 250 µM	-39.17	No	ns
FCCp vs. Butyrate	10 µM FCCP vs. 500 µM	-46.5	No	ns
FCCp vs. Butyrate	10 µM FCCP vs. 750 µM	-56.5	No	ns
FCCp vs. Butyrate	10 µM FCCP vs. 1 mM	-61.5	No	ns
FCCp vs. Butyrate	10 µM FCCP vs. 2.5 mM	-67.5	No	ns

FCCp vs. Butyrate	10 µM FCCP vs. 5 mM	-38.83	No	ns
FCCp vs. Butyrate	10 µM FCCP vs. 7.5 mM	-39.5	No	ns
FCCp Vs. Combo	10 µM FCCP vs. 50 pg/mL	-72.17	No	ns
FCCp Vs. Combo	10 µM FCCP vs. 100 pg/mL	-31.83	No	ns
FCCp Vs. Combo	10 µM FCCP vs. 150 pg/mL	-54	No	ns
FCCp Vs. Combo	10 µM FCCP vs. 200 pg/mL	-62	No	ns
FCCp Vs. Combo	10 µM FCCP vs. 250 pg/mL	-72.5	No	ns
FCCp Vs. Combo	10 µM FCCP vs. 500 pg/mL	-81.83	Yes	**
FCCp Vs. Combo	10 µM FCCP vs. 1 ng/mL	-67.5	No	ns
FCCp Vs. Combo	10 µM FCCP vs. 2.5 ng/mL	-56.83	No	ns
FCCp Vs. Combo	10 µM FCCP vs. 5 ng/mL	-43.83	No	ns
TNFa vs. TNFa	50 pg/mL vs. 100 pg/mL	6.667	No	ns
TNFa vs. TNFa	50 pg/mL vs. 150 pg/mL	-2.333	No	ns
TNFa vs. TNFa	50 pg/mL vs. 200 pg/mL	11.67	No	ns
TNFa vs. TNFa	50 pg/mL vs. 250 pg/mL	8.333	No	ns
TNFa vs. TNFa	50 pg/mL vs. 500 pg/mL	15.33	No	ns
TNFa vs. TNFa	50 pg/mL vs. 1 ng/mL	13.67	No	ns
TNFa vs. TNFa	50 pg/mL vs. 2.5 ng/mL	29.33	No	ns
TNFa vs. TNFa	50 pg/mL vs. 5 ng/mL	15.67	No	ns
TNFa vs. Butyrate	50 pg/mL vs. 50 µM	-34	No	ns
TNFa vs. Butyrate	50 pg/mL vs. 75 µM	-35.67	No	ns
TNFa vs. Butyrate	50 pg/mL vs. 100 µM	-26.67	No	ns
TNFa vs. Butyrate	50 pg/mL vs. 250 µM	-3.667	No	ns
TNFa vs. Butyrate	50 pg/mL vs. 500 µM	-11	No	ns
TNFa vs. Butyrate	50 pg/mL vs. 750 µM	-21	No	ns
TNFa vs. Butyrate	50 pg/mL vs. 1 mM	-26	No	ns
TNFa vs. Butyrate	50 pg/mL vs. 2.5 mM	-32	No	ns
TNFa vs. Butyrate	50 pg/mL vs. 5 mM	-3.333	No	ns
TNFa vs. Butyrate	50 pg/mL vs. 7.5 mM	-4	No	ns
TNFa vs. Combo	50 pg/mL vs. 50 pg/mL	-36.67	No	ns
TNFa vs. Combo	50 pg/mL vs. 100 pg/mL	3.667	No	ns
TNFa vs. Combo	50 pg/mL vs. 150 pg/mL	-18.5	No	ns
TNFa vs. Combo	50 pg/mL vs. 200 pg/mL	-26.5	No	ns
TNFa vs. Combo	50 pg/mL vs. 250 pg/mL	-37	No	ns
TNFa vs. Combo	50 pg/mL vs. 500 pg/mL	-46.33	No	ns
TNFa vs. Combo	50 pg/mL vs. 1 ng/mL	-32	No	ns
TNFa vs. Combo	50 pg/mL vs. 2.5 ng/mL	-21.33	No	ns
TNFa vs. Combo	50 pg/mL vs. 5 ng/mL	-8.333	No	ns
TNFa vs. TNFa	100 pg/mL vs. 150 pg/mL	-9	No	ns
TNFa vs. TNFa	100 pg/mL vs. 200 pg/mL	5	No	ns
TNFa vs. TNFa	100 pg/mL vs. 250 pg/mL	1.667	No	ns
TNFa vs. TNFa	100 pg/mL vs. 500 pg/mL	8.667	No	ns
TNFa vs. TNFa	100 pg/mL vs. 1 ng/mL	7	No	ns
TNFa vs. TNFa	100 pg/mL vs. 2.5 ng/mL	22.67	No	ns
TNFa vs. TNFa	100 pg/mL vs. 5 ng/mL	9	No	ns
TNFa vs. Butyrate	100 pg/mL vs. 50 µM	-40.67	No	ns
TNFa vs. Butyrate	100 pg/mL vs. 75 µM	-42.33	No	ns

TNFa vs. Butyrate	100 pg/mL vs. 100 µM	-33.33	No	ns
TNFa vs. Butyrate	100 pg/mL vs. 250 µM	-10.33	No	ns
TNFa vs. Butyrate	100 pg/mL vs. 500 µM	-17.67	No	ns
TNFa vs. Butyrate	100 pg/mL vs. 750 µM	-27.67	No	ns
TNFa vs. Butyrate	100 pg/mL vs. 1 mM	-32.67	No	ns
TNFa vs. Butyrate	100 pg/mL vs. 2.5 mM	-38.67	No	ns
TNFa vs. Butyrate	100 pg/mL vs. 5 mM	-10	No	ns
TNFa vs. Butyrate	100 pg/mL vs. 7.5 mM	-10.67	No	ns
TNFa vs. Combo	100 pg/mL vs. 50 pg/mL	-43.33	No	ns
TNFa vs. Combo	100 pg/mL vs. 100 pg/mL	-3	No	ns
TNFa vs. Combo	100 pg/mL vs. 150 pg/mL	-25.17	No	ns
TNFa vs. Combo	100 pg/mL vs. 200 pg/mL	-33.17	No	ns
TNFa vs. Combo	100 pg/mL vs. 250 pg/mL	-43.67	No	ns
TNFa vs. Combo	100 pg/mL vs. 500 pg/mL	-53	No	ns
TNFa vs. Combo	100 pg/mL vs. 1 ng/mL	-38.67	No	ns
TNFa vs. Combo	100 pg/mL vs. 2.5 ng/mL	-28	No	ns
TNFa vs. Combo	100 pg/mL vs. 5 ng/mL	-15	No	ns
TNFa vs. TNFa	150 pg/mL vs. 200 pg/mL	14	No	ns
TNFa vs. TNFa	150 pg/mL vs. 250 pg/mL	10.67	No	ns
TNFa vs. TNFa	150 pg/mL vs. 500 pg/mL	17.67	No	ns
TNFa vs. TNFa	150 pg/mL vs. 1 ng/mL	16	No	ns
TNFa vs. TNFa	150 pg/mL vs. 2.5 ng/mL	31.67	No	ns
TNFa vs. TNFa	150 pg/mL vs. 5 ng/mL	18	No	ns
TNFa vs. Butyrate	150 pg/mL vs. 50 µM	-31.67	No	ns
TNFa vs. Butyrate	150 pg/mL vs. 75 µM	-33.33	No	ns
TNFa vs. Butyrate	150 pg/mL vs. 100 µM	-24.33	No	ns
TNFa vs. Butyrate	150 pg/mL vs. 250 µM	-1.333	No	ns
TNFa vs. Butyrate	150 pg/mL vs. 500 µM	-8.667	No	ns
TNFa vs. Butyrate	150 pg/mL vs. 750 µM	-18.67	No	ns
TNFa vs. Butyrate	150 pg/mL vs. 1 mM	-23.67	No	ns
TNFa vs. Butyrate	150 pg/mL vs. 2.5 mM	-29.67	No	ns
TNFa vs. Butyrate	150 pg/mL vs. 5 mM	-1	No	ns
TNFa vs. Butyrate	150 pg/mL vs. 7.5 mM	-1.667	No	ns
TNFa vs. Combo	150 pg/mL vs. 50 pg/mL	-34.33	No	ns
TNFa vs. Combo	150 pg/mL vs. 100 pg/mL	6	No	ns
TNFa vs. Combo	150 pg/mL vs. 150 pg/mL	-16.17	No	ns
TNFa vs. Combo	150 pg/mL vs. 200 pg/mL	-24.17	No	ns
TNFa vs. Combo	150 pg/mL vs. 250 pg/mL	-34.67	No	ns
TNFa vs. Combo	150 pg/mL vs. 500 pg/mL	-44	No	ns
TNFa vs. Combo	150 pg/mL vs. 1 ng/mL	-29.67	No	ns
TNFa vs. Combo	150 pg/mL vs. 2.5 ng/mL	-19	No	ns
TNFa vs. Combo	150 pg/mL vs. 5 ng/mL	-6	No	ns
TNFa vs. TNFa	200 pg/mL vs. 250 pg/mL	-3.333	No	ns
TNFa vs. TNFa	200 pg/mL vs. 500 pg/mL	3.667	No	ns
TNFa vs. TNFa	200 pg/mL vs. 1 ng/mL	2	No	ns
TNFa vs. TNFa	200 pg/mL vs. 2.5 ng/mL	17.67	No	ns
TNFa vs. TNFa	200 pg/mL vs. 5 ng/mL	4	No	ns

TNFa vs. Butyrate	200 pg/mL vs. 50 µM	-45.67	No	ns
TNFa vs. Butyrate	200 pg/mL vs. 75 µM	-47.33	No	ns
TNFa vs. Butyrate	200 pg/mL vs. 100 µM	-38.33	No	ns
TNFa vs. Butyrate	200 pg/mL vs. 250 µM	-15.33	No	ns
TNFa vs. Butyrate	200 pg/mL vs. 500 µM	-22.67	No	ns
TNFa vs. Butyrate	200 pg/mL vs. 750 µM	-32.67	No	ns
TNFa vs. Butyrate	200 pg/mL vs. 1 mM	-37.67	No	ns
TNFa vs. Butyrate	200 pg/mL vs. 2.5 mM	-43.67	No	ns
TNFa vs. Butyrate	200 pg/mL vs. 5 mM	-15	No	ns
TNFa vs. Butyrate	200 pg/mL vs. 7.5 mM	-15.67	No	ns
TNFa vs. Combo	200 pg/mL vs. 50 pg/mL	-48.33	No	ns
TNFa vs. Combo	200 pg/mL vs. 100 pg/mL	-8	No	ns
TNFa vs. Combo	200 pg/mL vs. 150 pg/mL	-30.17	No	ns
TNFa vs. Combo	200 pg/mL vs. 200 pg/mL	-38.17	No	ns
TNFa vs. Combo	200 pg/mL vs. 250 pg/mL	-48.67	No	ns
TNFa vs. Combo	200 pg/mL vs. 500 pg/mL	-58	No	ns
TNFa vs. Combo	200 pg/mL vs. 1 ng/mL	-43.67	No	ns
TNFa vs. Combo	200 pg/mL vs. 2.5 ng/mL	-33	No	ns
TNFa vs. Combo	200 pg/mL vs. 5 ng/mL	-20	No	ns
TNFa vs. TNFa	250 pg/mL vs. 500 pg/mL	7	No	ns
TNFa vs. TNFa	250 pg/mL vs. 1 ng/mL	5.333	No	ns
TNFa vs. TNFa	250 pg/mL vs. 2.5 ng/mL	21	No	ns
TNFa vs. TNFa	250 pg/mL vs. 5 ng/mL	7.333	No	ns
TNFa vs. Butyrate	250 pg/mL vs. 50 µM	-42.33	No	ns
TNFa vs. Butyrate	250 pg/mL vs. 75 µM	-44	No	ns
TNFa vs. Butyrate	250 pg/mL vs. 100 µM	-35	No	ns
TNFa vs. Butyrate	250 pg/mL vs. 250 µM	-12	No	ns
TNFa vs. Butyrate	250 pg/mL vs. 500 µM	-19.33	No	ns
TNFa vs. Butyrate	250 pg/mL vs. 750 µM	-29.33	No	ns
TNFa vs. Butyrate	250 pg/mL vs. 1 mM	-34.33	No	ns
TNFa vs. Butyrate	250 pg/mL vs. 2.5 mM	-40.33	No	ns
TNFa vs. Butyrate	250 pg/mL vs. 5 mM	-11.67	No	ns
TNFa vs. Butyrate	250 pg/mL vs. 7.5 mM	-12.33	No	ns
TNFa vs. Combo	250 pg/mL vs. 50 pg/mL	-45	No	ns
TNFa vs. Combo	250 pg/mL vs. 100 pg/mL	-4.667	No	ns
TNFa vs. Combo	250 pg/mL vs. 150 pg/mL	-26.83	No	ns
TNFa vs. Combo	250 pg/mL vs. 200 pg/mL	-34.83	No	ns
TNFa vs. Combo	250 pg/mL vs. 250 pg/mL	-45.33	No	ns
TNFa vs. Combo	250 pg/mL vs. 500 pg/mL	-54.67	No	ns
TNFa vs. Combo	250 pg/mL vs. 1 ng/mL	-40.33	No	ns
TNFa vs. Combo	250 pg/mL vs. 2.5 ng/mL	-29.67	No	ns
TNFa vs. Combo	250 pg/mL vs. 5 ng/mL	-16.67	No	ns
TNFa vs. TNFa	500 pg/mL vs. 1 ng/mL	-1.667	No	ns
TNFa vs. TNFa	500 pg/mL vs. 2.5 ng/mL	14	No	ns
TNFa vs. TNFa	500 pg/mL vs. 5 ng/mL	0.3333	No	ns
TNFa vs. Butyrate	500 pg/mL vs. 50 µM	-49.33	No	ns
TNFa vs. Butyrate	500 pg/mL vs. 75 µM	-51	No	ns

TNFa vs. Butyrate	500 pg/mL vs. 100 µM	-42	No	ns
TNFa vs. Butyrate	500 pg/mL vs. 250 µM	-19	No	ns
TNFa vs. Butyrate	500 pg/mL vs. 500 µM	-26.33	No	ns
TNFa vs. Butyrate	500 pg/mL vs. 750 µM	-36.33	No	ns
TNFa vs. Butyrate	500 pg/mL vs. 1 mM	-41.33	No	ns
TNFa vs. Butyrate	500 pg/mL vs. 2.5 mM	-47.33	No	ns
TNFa vs. Butyrate	500 pg/mL vs. 5 mM	-18.67	No	ns
TNFa vs. Butyrate	500 pg/mL vs. 7.5 mM	-19.33	No	ns
TNFa vs. Combo	500 pg/mL vs. 50 pg/mL	-52	No	ns
TNFa vs. Combo	500 pg/mL vs. 100 pg/mL	-11.67	No	ns
TNFa vs. Combo	500 pg/mL vs. 150 pg/mL	-33.83	No	ns
TNFa vs. Combo	500 pg/mL vs. 200 pg/mL	-41.83	No	ns
TNFa vs. Combo	500 pg/mL vs. 250 pg/mL	-52.33	No	ns
TNFa vs. Combo	500 pg/mL vs. 500 pg/mL	-61.67	No	ns
TNFa vs. Combo	500 pg/mL vs. 1 ng/mL	-47.33	No	ns
TNFa vs. Combo	500 pg/mL vs. 2.5 ng/mL	-36.67	No	ns
TNFa vs. Combo	500 pg/mL vs. 5 ng/mL	-23.67	No	ns
TNFa vs. TNFa	1 ng/mL vs. 2.5 ng/mL	15.67	No	ns
TNFa vs. TNFa	1 ng/mL vs. 5 ng/mL	2	No	ns
TNFa vs. Butyrate	1 ng/mL vs. 50 µM	-47.67	No	ns
TNFa vs. Butyrate	1 ng/mL vs. 75 µM	-49.33	No	ns
TNFa vs. Butyrate	1 ng/mL vs. 100 µM	-40.33	No	ns
TNFa vs. Butyrate	1 ng/mL vs. 250 µM	-17.33	No	ns
TNFa vs. Butyrate	1 ng/mL vs. 500 µM	-24.67	No	ns
TNFa vs. Butyrate	1 ng/mL vs. 750 µM	-34.67	No	ns
TNFa vs. Butyrate	1 ng/mL vs. 1 mM	-39.67	No	ns
TNFa vs. Butyrate	1 ng/mL vs. 2.5 mM	-45.67	No	ns
TNFa vs. Butyrate	1 ng/mL vs. 5 mM	-17	No	ns
TNFa vs. Butyrate	1 ng/mL vs. 7.5 mM	-17.67	No	ns
TNFa vs. Combo	1 ng/mL vs. 50 pg/mL	-50.33	No	ns
TNFa vs. Combo	1 ng/mL vs. 100 pg/mL	-10	No	ns
TNFa vs. Combo	1 ng/mL vs. 150 pg/mL	-32.17	No	ns
TNFa vs. Combo	1 ng/mL vs. 200 pg/mL	-40.17	No	ns
TNFa vs. Combo	1 ng/mL vs. 250 pg/mL	-50.67	No	ns
TNFa vs. Combo	1 ng/mL vs. 500 pg/mL	-60	No	ns
TNFa vs. Combo	1 ng/mL vs. 1 ng/mL	-45.67	No	ns
TNFa vs. Combo	1 ng/mL vs. 2.5 ng/mL	-35	No	ns
TNFa vs. Combo	1 ng/mL vs. 5 ng/mL	-22	No	ns
TNFa vs. TNFa	2.5 ng/mL vs. 5 ng/mL	-13.67	No	ns
TNFa vs. Butyrate	2.5 ng/mL vs. 50 µM	-63.33	No	ns
TNFa vs. Butyrate	2.5 ng/mL vs. 75 µM	-65	No	ns
TNFa vs. Butyrate	2.5 ng/mL vs. 100 µM	-56	No	ns
TNFa vs. Butyrate	2.5 ng/mL vs. 250 µM	-33	No	ns
TNFa vs. Butyrate	2.5 ng/mL vs. 500 µM	-40.33	No	ns
TNFa vs. Butyrate	2.5 ng/mL vs. 750 µM	-50.33	No	ns
TNFa vs. Butyrate	2.5 ng/mL vs. 1 mM	-55.33	No	ns
TNFa vs. Butyrate	2.5 ng/mL vs. 2.5 mM	-61.33	No	ns

TNFa vs. Butyrate	2.5 ng/mL vs. 5 mM	-32.67	No	ns
TNFa vs. Butyrate	2.5 ng/mL vs. 7.5 mM	-33.33	No	ns
TNFa vs. Combo	2.5 ng/mL vs. 50 pg/mL	-66	No	ns
TNFa vs. Combo	2.5 ng/mL vs. 100 pg/mL	-25.67	No	ns
TNFa vs. Combo	2.5 ng/mL vs. 150 pg/mL	-47.83	No	ns
TNFa vs. Combo	2.5 ng/mL vs. 200 pg/mL	-55.83	No	ns
TNFa vs. Combo	2.5 ng/mL vs. 250 pg/mL	-66.33	No	ns
TNFa vs. Combo	2.5 ng/mL vs. 500 pg/mL	-75.67	No	ns
TNFa vs. Combo	2.5 ng/mL vs. 1 ng/mL	-61.33	No	ns
TNFa vs. Combo	2.5 ng/mL vs. 2.5 ng/mL	-50.67	No	ns
TNFa vs. Combo	2.5 ng/mL vs. 5 ng/mL	-37.67	No	ns
TNFa vs. Butyrate	5 ng/mL vs. 50 µM	-49.67	No	ns
TNFa vs. Butyrate	5 ng/mL vs. 75 µM	-51.33	No	ns
TNFa vs. Butyrate	5 ng/mL vs. 100 µM	-42.33	No	ns
TNFa vs. Butyrate	5 ng/mL vs. 250 µM	-19.33	No	ns
TNFa vs. Butyrate	5 ng/mL vs. 500 µM	-26.67	No	ns
TNFa vs. Butyrate	5 ng/mL vs. 750 µM	-36.67	No	ns
TNFa vs. Butyrate	5 ng/mL vs. 1 mM	-41.67	No	ns
TNFa vs. Butyrate	5 ng/mL vs. 2.5 mM	-47.67	No	ns
TNFa vs. Butyrate	5 ng/mL vs. 5 mM	-19	No	ns
TNFa vs. Butyrate	5 ng/mL vs. 7.5 mM	-19.67	No	ns
TNFa vs. Combo	5 ng/mL vs. 50 pg/mL	-52.33	No	ns
TNFa vs. Combo	5 ng/mL vs. 100 pg/mL	-12	No	ns
TNFa vs. Combo	5 ng/mL vs. 150 pg/mL	-34.17	No	ns
TNFa vs. Combo	5 ng/mL vs. 200 pg/mL	-42.17	No	ns
TNFa vs. Combo	5 ng/mL vs. 250 pg/mL	-52.67	No	ns
TNFa vs. Combo	5 ng/mL vs. 500 pg/mL	-62	No	ns
TNFa vs. Combo	5 ng/mL vs. 1 ng/mL	-47.67	No	ns
TNFa vs. Combo	5 ng/mL vs. 2.5 ng/mL	-37	No	ns
TNFa vs. Combo	5 ng/mL vs. 5 ng/mL	-24	No	ns
Butyrate vs. Butyrate	50 µM vs. 75 µM	-1.667	No	ns
Butyrate vs. Butyrate	50 µM vs. 100 µM	7.333	No	ns
Butyrate vs. Butyrate	50 µM vs. 250 µM	30.33	No	ns
Butyrate vs. Butyrate	50 µM vs. 500 µM	23	No	ns
Butyrate vs. Butyrate	50 µM vs. 750 µM	13	No	ns
Butyrate vs. Butyrate	50 µM vs. 1 mM	8	No	ns
Butyrate vs. Butyrate	50 µM vs. 2.5 mM	2	No	ns
Butyrate vs. Butyrate	50 µM vs. 5 mM	30.67	No	ns
Butyrate vs. Butyrate	50 µM vs. 7.5 mM	30	No	ns
Butyrate vs. Combo	50 µM vs. 50 pg/mL	-2.667	No	ns
Butyrate vs. Combo	50 µM vs. 100 pg/mL	37.67	No	ns
Butyrate vs. Combo	50 µM vs. 150 pg/mL	15.5	No	ns
Butyrate vs. Combo	50 µM vs. 200 pg/mL	7.5	No	ns
Butyrate vs. Combo	50 µM vs. 250 pg/mL	-3	No	ns
Butyrate vs. Combo	50 µM vs. 500 pg/mL	-12.33	No	ns
Butyrate vs. Combo	50 µM vs. 1 ng/mL	2	No	ns
Butyrate vs. Combo	50 µM vs. 2.5 ng/mL	12.67	No	ns

Butyrate vs. Combo	50 µM vs. 5 ng/mL	25.67	No	ns
Butyrate vs. Butyrate	75 µM vs. 100 µM	9	No	ns
Butyrate vs. Butyrate	75 µM vs. 250 µM	32	No	ns
Butyrate vs. Butyrate	75 µM vs. 500 µM	24.67	No	ns
Butyrate vs. Butyrate	75 µM vs. 750 µM	14.67	No	ns
Butyrate vs. Butyrate	75 µM vs. 1 mM	9.667	No	ns
Butyrate vs. Butyrate	75 µM vs. 2.5 mM	3.667	No	ns
Butyrate vs. Butyrate	75 µM vs. 5 mM	32.33	No	ns
Butyrate vs. Butyrate	75 µM vs. 7.5 mM	31.67	No	ns
Butyrate vs. Combo	75 µM vs. 50 pg/mL	-1	No	ns
Butyrate vs. Combo	75 µM vs. 100 pg/mL	39.33	No	ns
Butyrate vs. Combo	75 µM vs. 150 pg/mL	17.17	No	ns
Butyrate vs. Combo	75 µM vs. 200 pg/mL	9.167	No	ns
Butyrate vs. Combo	75 µM vs. 250 pg/mL	-1.333	No	ns
Butyrate vs. Combo	75 µM vs. 500 pg/mL	-10.67	No	ns
Butyrate vs. Combo	75 µM vs. 1 ng/mL	3.667	No	ns
Butyrate vs. Combo	75 µM vs. 2.5 ng/mL	14.33	No	ns
Butyrate vs. Combo	75 µM vs. 5 ng/mL	27.33	No	ns
Butyrate vs. Butyrate	100 µM vs. 250 µM	23	No	ns
Butyrate vs. Butyrate	100 µM vs. 500 µM	15.67	No	ns
Butyrate vs. Butyrate	100 µM vs. 750 µM	5.667	No	ns
Butyrate vs. Butyrate	100 µM vs. 1 mM	0.6667	No	ns
Butyrate vs. Butyrate	100 µM vs. 2.5 mM	-5.333	No	ns
Butyrate vs. Butyrate	100 µM vs. 5 mM	23.33	No	ns
Butyrate vs. Butyrate	100 µM vs. 7.5 mM	22.67	No	ns
Butyrate vs. Combo	100 µM vs. 50 pg/mL	-10	No	ns
Butyrate vs. Combo	100 µM vs. 100 pg/mL	30.33	No	ns
Butyrate vs. Combo	100 µM vs. 150 pg/mL	8.167	No	ns
Butyrate vs. Combo	100 µM vs. 200 pg/mL	0.1667	No	ns
Butyrate vs. Combo	100 µM vs. 250 pg/mL	-10.33	No	ns
Butyrate vs. Combo	100 µM vs. 500 pg/mL	-19.67	No	ns
Butyrate vs. Combo	100 µM vs. 1 ng/mL	-5.333	No	ns
Butyrate vs. Combo	100 µM vs. 2.5 ng/mL	5.333	No	ns
Butyrate vs. Combo	100 µM vs. 5 ng/mL	18.33	No	ns
Butyrate vs. Butyrate	250 µM vs. 500 µM	-7.333	No	ns
Butyrate vs. Butyrate	250 µM vs. 750 µM	-17.33	No	ns
Butyrate vs. Butyrate	250 µM vs. 1 mM	-22.33	No	ns
Butyrate vs. Butyrate	250 µM vs. 2.5 mM	-28.33	No	ns
Butyrate vs. Butyrate	250 µM vs. 5 mM	0.3333	No	ns
Butyrate vs. Butyrate	250 µM vs. 7.5 mM	-0.3333	No	ns
Butyrate vs. Combo	250 µM vs. 50 pg/mL	-33	No	ns
Butyrate vs. Combo	250 µM vs. 100 pg/mL	7.333	No	ns
Butyrate vs. Combo	250 µM vs. 150 pg/mL	-14.83	No	ns
Butyrate vs. Combo	250 µM vs. 200 pg/mL	-22.83	No	ns
Butyrate vs. Combo	250 µM vs. 250 pg/mL	-33.33	No	ns
Butyrate vs. Combo	250 µM vs. 500 pg/mL	-42.67	No	ns
Butyrate vs. Combo	250 µM vs. 1 ng/mL	-28.33	No	ns

Butyrate vs. Combo	250 µM vs. 2.5 ng/mL	-17.67	No	ns
Butyrate vs. Combo	250 µM vs. 5 ng/mL	-4.667	No	ns
Butyrate vs. Butyrate	500 µM vs. 750 µM	-10	No	ns
Butyrate vs. Butyrate	500 µM vs. 1 mM	-15	No	ns
Butyrate vs. Butyrate	500 µM vs. 2.5 mM	-21	No	ns
Butyrate vs. Butyrate	500 µM vs. 5 mM	7.667	No	ns
Butyrate vs. Butyrate	500 µM vs. 7.5 mM	7	No	ns
Butyrate vs. Combo	500 µM vs. 50 pg/mL	-25.67	No	ns
Butyrate vs. Combo	500 µM vs. 100 pg/mL	14.67	No	ns
Butyrate vs. Combo	500 µM vs. 150 pg/mL	-7.5	No	ns
Butyrate vs. Combo	500 µM vs. 200 pg/mL	-15.5	No	ns
Butyrate vs. Combo	500 µM vs. 250 pg/mL	-26	No	ns
Butyrate vs. Combo	500 µM vs. 500 pg/mL	-35.33	No	ns
Butyrate vs. Combo	500 µM vs. 1 ng/mL	-21	No	ns
Butyrate vs. Combo	500 µM vs. 2.5 ng/mL	-10.33	No	ns
Butyrate vs. Combo	500 µM vs. 5 ng/mL	2.667	No	ns
Butyrate vs. Butyrate	750 µM vs. 1 mM	-5	No	ns
Butyrate vs. Butyrate	750 µM vs. 2.5 mM	-11	No	ns
Butyrate vs. Butyrate	750 µM vs. 5 mM	17.67	No	ns
Butyrate vs. Butyrate	750 µM vs. 7.5 mM	17	No	ns
Butyrate vs. Combo	750 µM vs. 50 pg/mL	-15.67	No	ns
Butyrate vs. Combo	750 µM vs. 100 pg/mL	24.67	No	ns
Butyrate vs. Combo	750 µM vs. 150 pg/mL	2.5	No	ns
Butyrate vs. Combo	750 µM vs. 200 pg/mL	-5.5	No	ns
Butyrate vs. Combo	750 µM vs. 250 pg/mL	-16	No	ns
Butyrate vs. Combo	750 µM vs. 500 pg/mL	-25.33	No	ns
Butyrate vs. Combo	750 µM vs. 1 ng/mL	-11	No	ns
Butyrate vs. Combo	750 µM vs. 2.5 ng/mL	-0.3333	No	ns
Butyrate vs. Combo	750 µM vs. 5 ng/mL	12.67	No	ns
Butyrate vs. Butyrate	1 mM vs. 2.5 mM	-6	No	ns
Butyrate vs. Butyrate	1 mM vs. 5 mM	22.67	No	ns
Butyrate vs. Butyrate	1 mM vs. 7.5 mM	22	No	ns
Butyrate vs. Combo	1 mM vs. 50 pg/mL	-10.67	No	ns
Butyrate vs. Combo	1 mM vs. 100 pg/mL	29.67	No	ns
Butyrate vs. Combo	1 mM vs. 150 pg/mL	7.5	No	ns
Butyrate vs. Combo	1 mM vs. 200 pg/mL	-0.5	No	ns
Butyrate vs. Combo	1 mM vs. 250 pg/mL	-11	No	ns
Butyrate vs. Combo	1 mM vs. 500 pg/mL	-20.33	No	ns
Butyrate vs. Combo	1 mM vs. 1 ng/mL	-6	No	ns
Butyrate vs. Combo	1 mM vs. 2.5 ng/mL	4.667	No	ns
Butyrate vs. Combo	1 mM vs. 5 ng/mL	17.67	No	ns
Butyrate vs. Butyrate	2.5 mM vs. 5 mM	28.67	No	ns
Butyrate vs. Butyrate	2.5 mM vs. 7.5 mM	28	No	ns
Butyrate vs. Combo	2.5 mM vs. 50 pg/mL	-4.667	No	ns
Butyrate vs. Combo	2.5 mM vs. 100 pg/mL	35.67	No	ns
Butyrate vs. Combo	2.5 mM vs. 150 pg/mL	13.5	No	ns
Butyrate vs. Combo	2.5 mM vs. 200 pg/mL	5.5	No	ns

Butyrate vs. Combo	2.5 mM vs. 250 pg/mL	-5	No	ns
Butyrate vs. Combo	2.5 mM vs. 500 pg/mL	-14.33	No	ns
Butyrate vs. Combo	2.5 mM vs. 1 ng/mL	0	No	ns
Butyrate vs. Combo	2.5 mM vs. 2.5 ng/mL	10.67	No	ns
Butyrate vs. Combo	2.5 mM vs. 5 ng/mL	23.67	No	ns
Butyrate vs. Butyrate	5 mM vs. 7.5 mM	-0.6667	No	ns
Butyrate vs. Combo	5 mM vs. 50 pg/mL	-33.33	No	ns
Butyrate vs. Combo	5 mM vs. 100 pg/mL	7	No	ns
Butyrate vs. Combo	5 mM vs. 150 pg/mL	-15.17	No	ns
Butyrate vs. Combo	5 mM vs. 200 pg/mL	-23.17	No	ns
Butyrate vs. Combo	5 mM vs. 250 pg/mL	-33.67	No	ns
Butyrate vs. Combo	5 mM vs. 500 pg/mL	-43	No	ns
Butyrate vs. Combo	5 mM vs. 1 ng/mL	-28.67	No	ns
Butyrate vs. Combo	5 mM vs. 2.5 ng/mL	-18	No	ns
Butyrate vs. Combo	5 mM vs. 5 ng/mL	-5	No	ns
Butyrate vs. Combo	7.5 mM vs. 50 pg/mL	-32.67	No	ns
Butyrate vs. Combo	7.5 mM vs. 100 pg/mL	7.667	No	ns
Butyrate vs. Combo	7.5 mM vs. 150 pg/mL	-14.5	No	ns
Butyrate vs. Combo	7.5 mM vs. 200 pg/mL	-22.5	No	ns
Butyrate vs. Combo	7.5 mM vs. 250 pg/mL	-33	No	ns
Butyrate vs. Combo	7.5 mM vs. 500 pg/mL	-42.33	No	ns
Butyrate vs. Combo	7.5 mM vs. 1 ng/mL	-28	No	ns
Butyrate vs. Combo	7.5 mM vs. 2.5 ng/mL	-17.33	No	ns
Butyrate vs. Combo	7.5 mM vs. 5 ng/mL	-4.333	No	ns
Combo vs. Combo	50 pg/mL vs. 100 pg/mL	40.33	No	ns
Combo vs. Combo	50 pg/mL vs. 150 pg/mL	18.17	No	ns
Combo vs. Combo	50 pg/mL vs. 200 pg/mL	10.17	No	ns
Combo vs. Combo	50 pg/mL vs. 250 pg/mL	-0.3333	No	ns
Combo vs. Combo	50 pg/mL vs. 500 pg/mL	-9.667	No	ns
Combo vs. Combo	50 pg/mL vs. 1 ng/mL	4.667	No	ns
Combo vs. Combo	50 pg/mL vs. 2.5 ng/mL	15.33	No	ns
Combo vs. Combo	50 pg/mL vs. 5 ng/mL	28.33	No	ns
Combo vs. Combo	100 pg/mL vs. 150 pg/mL	-22.17	No	ns
Combo vs. Combo	100 pg/mL vs. 200 pg/mL	-30.17	No	ns
Combo vs. Combo	100 pg/mL vs. 250 pg/mL	-40.67	No	ns
Combo vs. Combo	100 pg/mL vs. 500 pg/mL	-50	No	ns
Combo vs. Combo	100 pg/mL vs. 1 ng/mL	-35.67	No	ns
Combo vs. Combo	100 pg/mL vs. 2.5 ng/mL	-25	No	ns
Combo vs. Combo	100 pg/mL vs. 5 ng/mL	-12	No	ns
Combo vs. Combo	150 pg/mL vs. 200 pg/mL	-8	No	ns
Combo vs. Combo	150 pg/mL vs. 250 pg/mL	-18.5	No	ns
Combo vs. Combo	150 pg/mL vs. 500 pg/mL	-27.83	No	ns
Combo vs. Combo	150 pg/mL vs. 1 ng/mL	-13.5	No	ns
Combo vs. Combo	150 pg/mL vs. 2.5 ng/mL	-2.833	No	ns
Combo vs. Combo	150 pg/mL vs. 5 ng/mL	10.17	No	ns
Combo vs. Combo	200 pg/mL vs. 250 pg/mL	-10.5	No	ns
Combo vs. Combo	200 pg/mL vs. 500 pg/mL	-19.83	No	ns

Combo vs. Combo	200 pg/mL vs. 1 ng/mL	-5.5	No	ns
Combo vs. Combo	200 pg/mL vs. 2.5 ng/mL	5.167	No	ns
Combo vs. Combo	200 pg/mL vs. 5 ng/mL	18.17	No	ns
Combo vs. Combo	250 pg/mL vs. 500 pg/mL	-9.333	No	ns
Combo vs. Combo	250 pg/mL vs. 1 ng/mL	5	No	ns
Combo vs. Combo	250 pg/mL vs. 2.5 ng/mL	15.67	No	ns
Combo vs. Combo	250 pg/mL vs. 5 ng/mL	28.67	No	ns
Combo vs. Combo	500 pg/mL vs. 1 ng/mL	14.33	No	ns
Combo vs. Combo	500 pg/mL vs. 2.5 ng/mL	25	No	ns
Combo vs. Combo	500 pg/mL vs. 5 ng/mL	38	No	ns
Combo vs. Combo	1 ng/mL vs. 2.5 ng/mL	10.67	No	ns
Combo vs. Combo	1 ng/mL vs. 5 ng/mL	23.67	No	ns
Combo vs. Combo	2.5 ng/mL vs. 5 ng/mL			

A-B
A-C
A-D
A-E
A-F
A-G
A-H
A-I
A-J
A-K
A-L
A-M
A-N
A-O
A-P
A-Q
A-R
A-S
A-T
A-U
A-V
A-W
A-X
A-Y
A-Z
A-AA
A-AB
A-AC
A-AD
B-C
B-D
B-E
B-F
B-G
B-H
B-I
B-J
B-K
B-L
B-M
B-N
B-O
B-P
B-Q
B-R
B-S

B-T
B-U
B-V
B-W
B-X
B-Y
B-Z
B-AA
B-AB
B-AC
B-AD
C-D
C-E
C-F
C-G
C-H
C-I
C-J
C-K
C-L
C-M
C-N
C-O
C-P
C-Q
C-R
C-S
C-T
C-U
C-V
C-W
C-X
C-Y
C-Z
C-AA
C-AB
C-AC
C-AD
D-E
D-F
D-G
D-H
D-I
D-J
D-K
D-L
D-M

D-N
D-O
D-P
D-Q
D-R
D-S
D-T
D-U
D-V
D-W
D-X
D-Y
D-Z
D-AA
D-AB
D-AC
D-AD
E-F
E-G
E-H
E-I
E-J
E-K
E-L
E-M
E-N
E-O
E-P
E-Q
E-R
E-S
E-T
E-U
E-V
E-W
E-X
E-Y
E-Z
E-AA
E-AB
E-AC
E-AD
F-G
F-H
F-I
F-J
F-K

F-L
F-M
F-N
F-O
F-P
F-Q
F-R
F-S
F-T
F-U
F-V
F-W
F-X
F-Y
F-Z
F-AA
F-AB
F-AC
F-AD
G-H
G-I
G-J
G-K
G-L
G-M
G-N
G-O
G-P
G-Q
G-R
G-S
G-T
G-U
G-V
G-W
G-X
G-Y
G-Z
G-AA
G-AB
G-AC
G-AD
H-I
H-J
H-K
H-L
H-M

H-N
H-O
H-P
H-Q
H-R
H-S
H-T
H-U
H-V
H-W
H-X
H-Y
H-Z
H-AA
H-AB
H-AC
H-AD
I-J
I-K
I-L
I-M
I-N
I-O
I-P
I-Q
I-R
I-S
I-T
I-U
I-V
I-W
I-X
I-Y
I-Z
I-AA
I-AB
I-AC
I-AD
J-K
J-L
J-M
J-N
J-O
J-P
J-Q
J-R
J-S

J-T
J-U
J-V
J-W
J-X
J-Y
J-Z
J-AA
J-AB
J-AC
J-AD
K-L
K-M
K-N
K-O
K-P
K-Q
K-R
K-S
K-T
K-U
K-V
K-W
K-X
K-Y
K-Z
K-AA
K-AB
K-AC
K-AD
L-M
L-N
L-O
L-P
L-Q
L-R
L-S
L-T
L-U
L-V
L-W
L-X
L-Y
L-Z
L-AA
L-AB
L-AC

L-AD
M-N
M-O
M-P
M-Q
M-R
M-S
M-T
M-U
M-V
M-W
M-X
M-Y
M-Z
M-AA
M-AB
M-AC
M-AD
N-O
N-P
N-Q
N-R
N-S
N-T
N-U
N-V
N-W
N-X
N-Y
N-Z
N-AA
N-AB
N-AC
N-AD
O-P
O-Q
O-R
O-S
O-T
O-U
O-V
O-W
O-X
O-Y
O-Z
O-AA
O-AB

O-AC
O-AD
P-Q
P-R
P-S
P-T
P-U
P-V
P-W
P-X
P-Y
P-Z
P-AA
P-AB
P-AC
P-AD
Q-R
Q-S
Q-T
Q-U
Q-V
Q-W
Q-X
Q-Y
Q-Z
Q-AA
Q-AB
Q-AC
Q-AD
R-S
R-T
R-U
R-V
R-W
R-X
R-Y
R-Z
R-AA
R-AB
R-AC
R-AD
S-T
S-U
S-V
S-W
S-X
S-Y

S-Z
S-AA
S-AB
S-AC
S-AD
T-U
T-V
T-W
T-X
T-Y
T-Z
T-AA
T-AB
T-AC
T-AD
U-V
U-W
U-X
U-Y
U-Z
U-AA
U-AB
U-AC
U-AD
V-W
V-X
V-Y
V-Z
V-AA
V-AB
V-AC
V-AD
W-X
W-Y
W-Z
W-AA
W-AB
W-AC
W-AD
X-Y
X-Z
X-AA
X-AB
X-AC
X-AD
Y-Z
Y-AA

Y-AB
Y-AC
Y-AD
Z-AA
Z-AB
Z-AC
Z-AD
AA-AB
AA-AC
AA-AD
AB-AC
AB-AD

Treatment	Dunn's multiple comparisons test	Mean rank	Significant?	Summary	Adjusted P
Media vs. FCCP	Media vs. 8µM FCCP	35.83	No	ns	>0.9999
Media Vs. Butyrate	Media vs. 10µM	35.5	No	ns	>0.9999
Media Vs. Butyrate	Media vs. 25µM	-7.5	No	ns	>0.9999
Media Vs. Butyrate	Media vs. 50µM	-26.5	No	ns	>0.9999
Media Vs. Butyrate	Media vs. 100µM	-52.33	No	ns	>0.9999
Media Vs. Butyrate	Media vs. 250µM	-71.67	No	ns	0.4464
Media Vs. Butyrate	Media vs. 500µM	-38.17	No	ns	>0.9999
Media vs. TNFa	Media vs. 10pg/mL	6	No	ns	>0.9999
Media vs. TNFa	Media vs. 25pg/mL	30.83	No	ns	>0.9999
Media vs. TNFa	Media vs. 50pg/mL	-10.5	No	ns	>0.9999
Media vs. TNFa	Media vs. 100pg/mL	1.5	No	ns	>0.9999
Media vs. TNFa	Media vs. 250pg/mL	-13.33	No	ns	>0.9999
Media vs. Combo	Media vs. 10pg/mL	16.17	No	ns	>0.9999
Media vs. Combo	Media vs. 25pg/mL	29.33	No	ns	>0.9999
Media vs. Combo	Media vs. 50pg/mL	-4.5	No	ns	>0.9999
Media vs. Combo	Media vs. 100pg/mL	-35.33	No	ns	>0.9999
Media vs. Combo	Media vs. 250pg/mL	-3.833	No	ns	>0.9999
Media vs. Combo	Media vs. 10pg/mL	-65	No	ns	>0.9999
Media vs. Combo	Media vs. 25pg/mL	-64.33	No	ns	>0.9999
Media vs. Combo	Media vs. 50pg/mL	-44.83	No	ns	>0.9999
Media vs. Combo	Media vs. 100pg/mL	-42.73	No	ns	>0.9999
Media vs. Combo	Media vs. 250pg/mL	-9	No	ns	>0.9999
FCCP vs. FCCP	8µM FCCP vs. 10µM	-0.3333	No	ns	>0.9999
FCCP Vs. Butyrate	8µM FCCP vs. 25µM	-43.33	No	ns	>0.9999
FCCP Vs. Butyrate	8µM FCCP vs. 50µM	-62.33	No	ns	>0.9999
FCCP Vs. Butyrate	8µM FCCP vs. 100µM	-88.17	Yes	*	0.0302
FCCP Vs. Butyrate	8µM FCCP vs. 250µM	-107.5	Yes	***	0.0007
FCCP Vs. Butyrate	8µM FCCP vs. 500µM	-74	No	ns	0.3142
FCCP Vs. Butyrate	8µM FCCP vs. 10pg/mL	-29.83	No	ns	>0.9999
FCCP vs. TNFa	8µM FCCP vs. 25pg/mL	-5	No	ns	>0.9999
FCCP vs. TNFa	8µM FCCP vs. 50pg/mL	-46.33	No	ns	>0.9999
FCCP vs. TNFa	8µM FCCP vs. 100pg/mL	-34.33	No	ns	>0.9999
FCCP vs. TNFa	8µM FCCP vs. 250pg/mL	-49.17	No	ns	>0.9999
FCCP vs. TNFa	8µM FCCP vs. 10pg/mL	-19.67	No	ns	>0.9999
FCCP vs. Combo	8µM FCCP vs. 25pg/mL	-6.5	No	ns	>0.9999
FCCP vs. Combo	8µM FCCP vs. 50pg/mL	-40.33	No	ns	>0.9999
FCCP vs. Combo	8µM FCCP vs. 100pg/mL	-71.17	No	ns	0.4806
FCCP vs. Combo	8µM FCCP vs. 250pg/mL	-39.67	No	ns	>0.9999
FCCP vs. Combo	8µM FCCP vs. 10pg/mL	-100.8	Yes	**	0.0027
FCCP vs. Combo	8µM FCCP vs. 25pg/mL	-100.2	Yes	**	0.0031
FCCP vs. Combo	8µM FCCP vs. 50pg/mL	-80.67	No	ns	0.1092
FCCP vs. Combo	8µM FCCP vs. 100pg/mL	-78.57	No	ns	0.2732
FCCP vs. Combo	8µM FCCP vs. 250pg/mL	-44.83	No	ns	>0.9999
Butyrate vs. Butyrate	10µM vs. 25µM	-43	No	ns	>0.9999
Butyrate vs. Butyrate	10µM vs. 50µM	-62	No	ns	>0.9999
Butyrate vs. Butyrate	10µM vs. 100µM	-87.83	Yes	*	0.0321

Butyrate vs. Butyrate	10µM vs. 250µM	-107.2	Yes	***	0.0007
Butyrate vs. Butyrate	10µM vs. 500µM	-73.67	No	ns	0.3305
Butyrate vs. TNFa	10µM vs. 10pg/mL	-29.5	No	ns	>0.9999
Butyrate vs. TNFa	10µM vs. 25pg/mL	-4.667	No	ns	>0.9999
Butyrate vs. TNFa	10µM vs. 50pg/mL	-46	No	ns	>0.9999
Butyrate vs. TNFa	10µM vs. 100pg/mL	-34	No	ns	>0.9999
Butyrate vs. TNFa	10µM vs. 250pg/mL	-48.83	No	ns	>0.9999
Butyrate vs. Combo	10µM vs. 10pg/mL	-19.33	No	ns	>0.9999
Butyrate vs. Combo	10µM vs. 25pg/mL	-6.167	No	ns	>0.9999
Butyrate vs. Combo	10µM vs. 50pg/mL	-40	No	ns	>0.9999
Butyrate vs. Combo	10µM vs. 100pg/mL	-70.83	No	ns	0.5048
Butyrate vs. Combo	10µM vs. 250pg/mL	-39.33	No	ns	>0.9999
Butyrate vs. Combo	10µM vs. 10pg/mL	-100.5	Yes	**	0.0029
Butyrate vs. Combo	10µM vs. 25pg/mL	-99.83	Yes	**	0.0033
Butyrate vs. Combo	10µM vs. 50pg/mL	-80.33	No	ns	0.1153
Butyrate vs. Combo	10µM vs. 100pg/mL	-78.23	No	ns	0.2869
Butyrate vs. Combo	10µM vs. 250pg/mL	-44.5	No	ns	>0.9999
Butyrate vs. Butyrate	25µM vs. 50µM	-19	No	ns	>0.9999
Butyrate vs. Butyrate	25µM vs. 100µM	-44.83	No	ns	>0.9999
Butyrate vs. Butyrate	25µM vs. 250µM	-64.17	No	ns	>0.9999
Butyrate vs. Butyrate	25µM vs. 500µM	-30.67	No	ns	>0.9999
Butyrate vs. TNFa	25µM vs. 10pg/mL	13.5	No	ns	>0.9999
Butyrate vs. TNFa	25µM vs. 25pg/mL	38.33	No	ns	>0.9999
Butyrate vs. TNFa	25µM vs. 50pg/mL	-3	No	ns	>0.9999
Butyrate vs. TNFa	25µM vs. 100pg/mL	9	No	ns	>0.9999
Butyrate vs. TNFa	25µM vs. 250pg/mL	-5.833	No	ns	>0.9999
Butyrate vs. Combo	25µM vs. 10pg/mL	23.67	No	ns	>0.9999
Butyrate vs. Combo	25µM vs. 25pg/mL	36.83	No	ns	>0.9999
Butyrate vs. Combo	25µM vs. 50pg/mL	3	No	ns	>0.9999
Butyrate vs. Combo	25µM vs. 100pg/mL	-27.83	No	ns	>0.9999
Butyrate vs. Combo	25µM vs. 250pg/mL	3.667	No	ns	>0.9999
Butyrate vs. Combo	25µM vs. 10pg/mL	-57.5	No	ns	>0.9999
Butyrate vs. Combo	25µM vs. 25pg/mL	-56.83	No	ns	>0.9999
Butyrate vs. Combo	25µM vs. 50pg/mL	-37.33	No	ns	>0.9999
Butyrate vs. Combo	25µM vs. 100pg/mL	-35.23	No	ns	>0.9999
Butyrate vs. Combo	25µM vs. 250pg/mL	-1.5	No	ns	>0.9999
Butyrate vs. Butyrate	50µM vs. 100µM	-25.83	No	ns	>0.9999
Butyrate vs. Butyrate	50µM vs. 250µM	-45.17	No	ns	>0.9999
Butyrate vs. Butyrate	50µM vs. 500µM	-11.67	No	ns	>0.9999
Butyrate vs. TNFa	50µM vs. 10pg/mL	32.5	No	ns	>0.9999
Butyrate vs. TNFa	50µM vs. 25pg/mL	57.33	No	ns	>0.9999
Butyrate vs. TNFa	50µM vs. 50pg/mL	16	No	ns	>0.9999
Butyrate vs. TNFa	50µM vs. 100pg/mL	28	No	ns	>0.9999
Butyrate vs. TNFa	50µM vs. 250pg/mL	13.17	No	ns	>0.9999
Butyrate vs. Combo	50µM vs. 10pg/mL	42.67	No	ns	>0.9999
Butyrate vs. Combo	50µM vs. 25pg/mL	55.83	No	ns	>0.9999
Butyrate vs. Combo	50µM vs. 50pg/mL	22	No	ns	>0.9999

Butyrate vs. Combo	50µM vs. 100pg/mL	-8.833	No	ns	>0.9999
Butyrate vs. Combo	50µM vs. 250pg/mL	22.67	No	ns	>0.9999
Butyrate vs. Combo	50µM vs. 10pg/mL	-38.5	No	ns	>0.9999
Butyrate vs. Combo	50µM vs. 25pg/mL	-37.83	No	ns	>0.9999
Butyrate vs. Combo	50µM vs. 50pg/mL	-18.33	No	ns	>0.9999
Butyrate vs. Combo	50µM vs. 100pg/mL	-16.23	No	ns	>0.9999
Butyrate vs. Combo	50µM vs. 250pg/mL	17.5	No	ns	>0.9999
Butyrate vs. Butyrate	100µM vs. 250µM	-19.33	No	ns	>0.9999
Butyrate vs. Butyrate	100µM vs. 500µM	14.17	No	ns	>0.9999
Butyrate vs. TNFa	100µM vs. 10pg/mL	58.33	No	ns	>0.9999
Butyrate vs. TNFa	100µM vs. 25pg/mL	83.17	No	ns	0.072
Butyrate vs. TNFa	100µM vs. 50pg/mL	41.83	No	ns	>0.9999
Butyrate vs. TNFa	100µM vs. 100pg/mL	53.83	No	ns	>0.9999
Butyrate vs. TNFa	100µM vs. 250pg/mL	39	No	ns	>0.9999
Butyrate vs. Combo	100µM vs. 10pg/mL	68.5	No	ns	0.7079
Butyrate vs. Combo	100µM vs. 25pg/mL	81.67	No	ns	0.0925
Butyrate vs. Combo	100µM vs. 50pg/mL	47.83	No	ns	>0.9999
Butyrate vs. Combo	100µM vs. 100pg/mL	17	No	ns	>0.9999
Butyrate vs. Combo	100µM vs. 250pg/mL	48.5	No	ns	>0.9999
Butyrate vs. Combo	100µM vs. 10pg/mL	-12.67	No	ns	>0.9999
Butyrate vs. Combo	100µM vs. 25pg/mL	-12	No	ns	>0.9999
Butyrate vs. Combo	100µM vs. 50pg/mL	7.5	No	ns	>0.9999
Butyrate vs. Combo	100µM vs. 100pg/mL	9.6	No	ns	>0.9999
Butyrate vs. Combo	100µM vs. 250pg/mL	43.33	No	ns	>0.9999
Butyrate vs. Butyrate	250µM vs. 500µM	33.5	No	ns	>0.9999
Butyrate vs. TNFa	250µM vs. 10pg/mL	77.67	No	ns	0.1774
Butyrate vs. TNFa	250µM vs. 25pg/mL	102.5	Yes	**	0.002
Butyrate vs. TNFa	250µM vs. 50pg/mL	61.17	No	ns	>0.9999
Butyrate vs. TNFa	250µM vs. 100pg/mL	73.17	No	ns	0.3565
Butyrate vs. TNFa	250µM vs. 250pg/mL	58.33	No	ns	>0.9999
Butyrate vs. Combo	250µM vs. 10pg/mL	87.83	Yes	*	0.0321
Butyrate vs. Combo	250µM vs. 25pg/mL	101	Yes	**	0.0026
Butyrate vs. Combo	250µM vs. 50pg/mL	67.17	No	ns	0.855
Butyrate vs. Combo	250µM vs. 100pg/mL	36.33	No	ns	>0.9999
Butyrate vs. Combo	250µM vs. 250pg/mL	67.83	No	ns	0.7783
Butyrate vs. Combo	250µM vs. 10pg/mL	6.667	No	ns	>0.9999
Butyrate vs. Combo	250µM vs. 25pg/mL	7.333	No	ns	>0.9999
Butyrate vs. Combo	250µM vs. 50pg/mL	26.83	No	ns	>0.9999
Butyrate vs. Combo	250µM vs. 100pg/mL	28.93	No	ns	>0.9999
Butyrate vs. Combo	250µM vs. 250pg/mL	62.67	No	ns	>0.9999
Butyrate vs. TNFa	500µM vs. 10pg/mL	44.17	No	ns	>0.9999
Butyrate vs. TNFa	500µM vs. 25pg/mL	69	No	ns	0.6589
Butyrate vs. TNFa	500µM vs. 50pg/mL	27.67	No	ns	>0.9999
Butyrate vs. TNFa	500µM vs. 100pg/mL	39.67	No	ns	>0.9999
Butyrate vs. TNFa	500µM vs. 250pg/mL	24.83	No	ns	>0.9999
Butyrate vs. Combo	500µM vs. 10pg/mL	54.33	No	ns	>0.9999
Butyrate vs. Combo	500µM vs. 25pg/mL	67.5	No	ns	0.8158

Butyrate vs. Combo	500µM vs. 50pg/mL	33.67	No	ns	>0.9999
Butyrate vs. Combo	500µM vs. 100pg/mL	2.833	No	ns	>0.9999
Butyrate vs. Combo	500µM vs. 250pg/mL	34.33	No	ns	>0.9999
Butyrate vs. Combo	500µM vs. 10pg/mL	-26.83	No	ns	>0.9999
Butyrate vs. Combo	500µM vs. 25pg/mL	-26.17	No	ns	>0.9999
Butyrate vs. Combo	500µM vs. 50pg/mL	-6.667	No	ns	>0.9999
Butyrate vs. Combo	500µM vs. 100pg/mL	-4.567	No	ns	>0.9999
Butyrate vs. Combo	500µM vs. 250pg/mL	29.17	No	ns	>0.9999
TNFa vs. TNFa	10pg/mL vs. 25pg/mL	24.83	No	ns	>0.9999
TNFa vs. TNFa	10pg/mL vs. 50pg/mL	-16.5	No	ns	>0.9999
TNFa vs. TNFa	10pg/mL vs. 100pg/mL	-4.5	No	ns	>0.9999
TNFa vs. TNFa	10pg/mL vs. 250pg/mL	-19.33	No	ns	>0.9999
TNFa vs. Combo	10pg/mL vs. 10pg/mL	10.17	No	ns	>0.9999
TNFa vs. Combo	10pg/mL vs. 25pg/mL	23.33	No	ns	>0.9999
TNFa vs. Combo	10pg/mL vs. 50pg/mL	-10.5	No	ns	>0.9999
TNFa vs. Combo	10pg/mL vs. 100pg/mL	-41.33	No	ns	>0.9999
TNFa vs. Combo	10pg/mL vs. 250pg/mL	-9.833	No	ns	>0.9999
TNFa vs. Combo	10pg/mL vs. 10pg/mL	-71	No	ns	0.4926
TNFa vs. Combo	10pg/mL vs. 25pg/mL	-70.33	No	ns	0.5432
TNFa vs. Combo	10pg/mL vs. 50pg/mL	-50.83	No	ns	>0.9999
TNFa vs. Combo	10pg/mL vs. 100pg/mL	-48.73	No	ns	>0.9999
TNFa vs. Combo	10pg/mL vs. 250pg/mL	-15	No	ns	>0.9999
TNFa vs. TNFa	25pg/mL vs. 50pg/mL	-41.33	No	ns	>0.9999
TNFa vs. TNFa	25pg/mL vs. 100pg/mL	-29.33	No	ns	>0.9999
TNFa vs. TNFa	25pg/mL vs. 250pg/mL	-44.17	No	ns	>0.9999
TNFa vs. Combo	25pg/mL vs. 10pg/mL	-14.67	No	ns	>0.9999
TNFa vs. Combo	25pg/mL vs. 25pg/mL	-1.5	No	ns	>0.9999
TNFa vs. Combo	25pg/mL vs. 50pg/mL	-35.33	No	ns	>0.9999
TNFa vs. Combo	25pg/mL vs. 100pg/mL	-66.17	No	ns	0.9831
TNFa vs. Combo	25pg/mL vs. 250pg/mL	-34.67	No	ns	>0.9999
TNFa vs. Combo	25pg/mL vs. 10pg/mL	-95.83	Yes	**	0.0073
TNFa vs. Combo	25pg/mL vs. 25pg/mL	-95.17	Yes	**	0.0083
TNFa vs. Combo	25pg/mL vs. 50pg/mL	-75.67	No	ns	0.243
TNFa vs. Combo	25pg/mL vs. 100pg/mL	-73.57	No	ns	0.5585
TNFa vs. Combo	25pg/mL vs. 250pg/mL	-39.83	No	ns	>0.9999
TNFa vs. TNFa	50pg/mL vs. 100pg/mL	12	No	ns	>0.9999
TNFa vs. TNFa	50pg/mL vs. 250pg/mL	-2.833	No	ns	>0.9999
TNFa vs. Combo	50pg/mL vs. 10pg/mL	26.67	No	ns	>0.9999
TNFa vs. Combo	50pg/mL vs. 25pg/mL	39.83	No	ns	>0.9999
TNFa vs. Combo	50pg/mL vs. 50pg/mL	6	No	ns	>0.9999
TNFa vs. Combo	50pg/mL vs. 100pg/mL	-24.83	No	ns	>0.9999
TNFa vs. Combo	50pg/mL vs. 250pg/mL	6.667	No	ns	>0.9999
TNFa vs. Combo	50pg/mL vs. 10pg/mL	-54.5	No	ns	>0.9999
TNFa vs. Combo	50pg/mL vs. 25pg/mL	-53.83	No	ns	>0.9999
TNFa vs. Combo	50pg/mL vs. 50pg/mL	-34.33	No	ns	>0.9999
TNFa vs. Combo	50pg/mL vs. 100pg/mL	-32.23	No	ns	>0.9999
TNFa vs. Combo	50pg/mL vs. 250pg/mL	1.5	No	ns	>0.9999

TNFa vs. TNFa	100pg/mL vs. 250pg/mL	-14.83	No	ns	>0.9999
TNFa vs. Combo	100pg/mL vs. 10pg/mL	14.67	No	ns	>0.9999
TNFa vs. Combo	100pg/mL vs. 25pg/mL	27.83	No	ns	>0.9999
TNFa vs. Combo	100pg/mL vs. 50pg/mL	-6	No	ns	>0.9999
TNFa vs. Combo	100pg/mL vs. 100pg/mL	-36.83	No	ns	>0.9999
TNFa vs. Combo	100pg/mL vs. 250pg/mL	-5.333	No	ns	>0.9999
TNFa vs. Combo	100pg/mL vs. 10pg/mL	-66.5	No	ns	0.9386
TNFa vs. Combo	100pg/mL vs. 25pg/mL	-65.83	No	ns	>0.9999
TNFa vs. Combo	100pg/mL vs. 50pg/mL	-46.33	No	ns	>0.9999
TNFa vs. Combo	100pg/mL vs. 100pg/mL	-44.23	No	ns	>0.9999
TNFa vs. Combo	100pg/mL vs. 250pg/mL	-10.5	No	ns	>0.9999
TNFa vs. Combo	250pg/mL vs. 10pg/mL	29.5	No	ns	>0.9999
TNFa vs. Combo	250pg/mL vs. 25pg/mL	42.67	No	ns	>0.9999
TNFa vs. Combo	250pg/mL vs. 50pg/mL	8.833	No	ns	>0.9999
TNFa vs. Combo	250pg/mL vs. 100pg/mL	-22	No	ns	>0.9999
TNFa vs. Combo	250pg/mL vs. 250pg/mL	9.5	No	ns	>0.9999
TNFa vs. Combo	250pg/mL vs. 10pg/mL	-51.67	No	ns	>0.9999
TNFa vs. Combo	250pg/mL vs. 25pg/mL	-51	No	ns	>0.9999
TNFa vs. Combo	250pg/mL vs. 50pg/mL	-31.5	No	ns	>0.9999
TNFa vs. Combo	250pg/mL vs. 100pg/mL	-29.4	No	ns	>0.9999
TNFa vs. Combo	250pg/mL vs. 250pg/mL	4.333	No	ns	>0.9999
Combo vs. Combo	10pg/mL vs. 25pg/mL	13.17	No	ns	>0.9999
Combo vs. Combo	10pg/mL vs. 50pg/mL	-20.67	No	ns	>0.9999
Combo vs. Combo	10pg/mL vs. 100pg/mL	-51.5	No	ns	>0.9999
Combo vs. Combo	10pg/mL vs. 250pg/mL	-20	No	ns	>0.9999
Combo vs. Combo	10pg/mL vs. 10pg/mL	-81.17	No	ns	0.1005
Combo vs. Combo	10pg/mL vs. 25pg/mL	-80.5	No	ns	0.1122
Combo vs. Combo	10pg/mL vs. 50pg/mL	-61	No	ns	>0.9999
Combo vs. Combo	10pg/mL vs. 100pg/mL	-58.9	No	ns	>0.9999
Combo vs. Combo	10pg/mL vs. 250pg/mL	-25.17	No	ns	>0.9999
Combo vs. Combo	25pg/mL vs. 50pg/mL	-33.83	No	ns	>0.9999
Combo vs. Combo	25pg/mL vs. 100pg/mL	-64.67	No	ns	>0.9999
Combo vs. Combo	25pg/mL vs. 250pg/mL	-33.17	No	ns	>0.9999
Combo vs. Combo	25pg/mL vs. 10pg/mL	-94.33	Yes	**	0.0097
Combo vs. Combo	25pg/mL vs. 25pg/mL	-93.67	Yes	*	0.011
Combo vs. Combo	25pg/mL vs. 50pg/mL	-74.17	No	ns	0.3063
Combo vs. Combo	25pg/mL vs. 100pg/mL	-72.07	No	ns	0.6866
Combo vs. Combo	25pg/mL vs. 250pg/mL	-38.33	No	ns	>0.9999
Combo vs. Combo	50pg/mL vs. 100pg/mL	-30.83	No	ns	>0.9999
Combo vs. Combo	50pg/mL vs. 250pg/mL	0.6667	No	ns	>0.9999
Combo vs. Combo	50pg/mL vs. 10pg/mL	-60.5	No	ns	>0.9999
Combo vs. Combo	50pg/mL vs. 25pg/mL	-59.83	No	ns	>0.9999
Combo vs. Combo	50pg/mL vs. 50pg/mL	-40.33	No	ns	>0.9999
Combo vs. Combo	50pg/mL vs. 100pg/mL	-38.23	No	ns	>0.9999
Combo vs. Combo	50pg/mL vs. 250pg/mL	-4.5	No	ns	>0.9999
Combo vs. Combo	100pg/mL vs. 250pg/mL	31.5	No	ns	>0.9999
Combo vs. Combo	100pg/mL vs. 10pg/mL	-29.67	No	ns	>0.9999

Combo vs. Combo	100pg/mL vs. 25pg/mL	-29	No	ns	>0.9999
Combo vs. Combo	100pg/mL vs. 50pg/mL	-9.5	No	ns	>0.9999
Combo vs. Combo	100pg/mL vs. 100pg/mL	-7.4	No	ns	>0.9999
Combo vs. Combo	100pg/mL vs. 250pg/mL	26.33	No	ns	>0.9999
Combo vs. Combo	250pg/mL vs. 10pg/mL	-61.17	No	ns	>0.9999
Combo vs. Combo	250pg/mL vs. 25pg/mL	-60.5	No	ns	>0.9999
Combo vs. Combo	250pg/mL vs. 50pg/mL	-41	No	ns	>0.9999
Combo vs. Combo	250pg/mL vs. 100pg/mL	-38.9	No	ns	>0.9999
Combo vs. Combo	250pg/mL vs. 250pg/mL	-5.167	No	ns	>0.9999
Combo vs. Combo	10pg/mL vs. 25pg/mL	0.6667	No	ns	>0.9999
Combo vs. Combo	10pg/mL vs. 50pg/mL	20.17	No	ns	>0.9999
Combo vs. Combo	10pg/mL vs. 100pg/mL	22.27	No	ns	>0.9999
Combo vs. Combo	10pg/mL vs. 250pg/mL	56	No	ns	>0.9999
Combo vs. Combo	25pg/mL vs. 50pg/mL	19.5	No	ns	>0.9999
Combo vs. Combo	25pg/mL vs. 100pg/mL	21.6	No	ns	>0.9999
Combo vs. Combo	25pg/mL vs. 250pg/mL	55.33	No	ns	>0.9999
Combo vs. Combo	50pg/mL vs. 100pg/mL	2.1	No	ns	>0.9999
Combo vs. Combo	50pg/mL vs. 250pg/mL	35.83	No	ns	>0.9999
Combo vs. Combo	100pg/mL vs. 250pg/mL	33.73	No		

Value
A-B
A-C
A-D
A-E
A-F
A-G
A-H
A-I
A-J
A-K
A-L
A-M
A-N
A-O
A-P
A-Q
A-R
A-S
A-T
A-U
A-V
A-W
B-C
B-D
B-E
B-F
B-G
B-H
B-I
B-J
B-K
B-L
B-M
B-N
B-O
B-P
B-Q
B-R
B-S
B-T
B-U
B-V
B-W
C-D
C-E
C-F

C-G
C-H
C-I
C-J
C-K
C-L
C-M
C-N
C-O
C-P
C-Q
C-R
C-S
C-T
C-U
C-V
C-W
D-E
D-F
D-G
D-H
D-I
D-J
D-K
D-L
D-M
D-N
D-O
D-P
D-Q
D-R
D-S
D-T
D-U
D-V
D-W
E-F
E-G
E-H
E-I
E-J
E-K
E-L
E-M
E-N
E-O
E-P

E-Q
E-R
E-S
E-T
E-U
E-V
E-W
F-G
F-H
F-I
F-J
F-K
F-L
F-M
F-N
F-O
F-P
F-Q
F-R
F-S
F-T
F-U
F-V
F-W
G-H
G-I
G-J
G-K
G-L
G-M
G-N
G-O
G-P
G-Q
G-R
G-S
G-T
G-U
G-V
G-W
H-I
H-J
H-K
H-L
H-M
H-N
H-O

H-P
H-Q
H-R
H-S
H-T
H-U
H-V
H-W
I-J
I-K
I-L
I-M
I-N
I-O
I-P
I-Q
I-R
I-S
I-T
I-U
I-V
I-W
J-K
J-L
J-M
J-N
J-O
J-P
J-Q
J-R
J-S
J-T
J-U
J-V
J-W
K-L
K-M
K-N
K-O
K-P
K-Q
K-R
K-S
K-T
K-U
K-V
K-W

L-M
L-N
L-O
L-P
L-Q
L-R
L-S
L-T
L-U
L-V
L-W
M-N
M-O
M-P
M-Q
M-R
M-S
M-T
M-U
M-V
M-W
N-O
N-P
N-Q
N-R
N-S
N-T
N-U
N-V
N-W
O-P
O-Q
O-R
O-S
O-T
O-U
O-V
O-W
P-Q
P-R
P-S
P-T
P-U
P-V
P-W
Q-R
Q-S

Q-T
Q-U
Q-V
Q-W
R-S
R-T
R-U
R-V
R-W
S-T
S-U
S-V
S-W
T-U
T-V
T-W
U-V
U-W

Treatment	Dunn's multiple comparisons test	Mean rank	Significant'	Summary
Media vs. FCCP	Media vs. 8uM FCCP	63.83	No	ns
Media vs. TNFa	Media vs. 50pg/mL	-17.33	No	ns
Media vs. TNFa	Media vs. 100pg/mL	20.67	No	ns
Media vs. TNFa	Media vs. 150pg/mL	23	No	ns
Media vs. TNFa	Media vs. 200pg/mL	44.83	No	ns
Media vs. TNFa	Media vs. 250pg/mL	31	No	ns
Media vs. TNFa	Media vs. 500pg/mL	22	No	ns
Media vs. TNFa	Media vs. 1000pg/mL	38.67	No	ns
Media vs. TNFa	Media vs. 2500pg/mL	37.33	No	ns
Media vs. TNFa	Media vs. 5000pg/mL	42.17	No	ns
Medea vs. Butyrate	Media vs. 50uM	-24.17	No	ns
Medea vs. Butyrate	Media vs. 75um	-33.5	No	ns
Medea vs. Butyrate	Media vs. 100uM	-62.33	No	ns
Medea vs. Butyrate	Media vs. 250µM	-72.6	No	ns
Medea vs. Butyrate	Media vs. 500uM	-91	No	ns
Medea vs. Butyrate	Media vs. 750uM	-93.67	No	ns
Medea vs. Butyrate	Media vs. 1000uM	-90.6	No	ns
Medea vs. Butyrate	Media vs. 2500uM	-59	No	ns
Medea vs. Butyrate	Media vs. 5000uM	-37.17	No	ns
Medea vs. Butyrate	Media vs. 7500uM	-16.8	No	ns
Media vs. Combo	Media vs. 50pg/mL	-78.5	No	ns
Media vs. Combo	Media vs. 100pg/mL	-62.83	No	ns
Media vs. Combo	Media vs. 150pg/mL	-71.33	No	ns
Media vs. Combo	Media vs. 200pg/mL	-69.5	No	ns
Media vs. Combo	Media vs. 250pg/mL	-61.17	No	ns
Media vs. Combo	Media vs. 500pg/mL	-44.17	No	ns
Media vs. Combo	Media vs. 1000pg/mL	-20	No	ns
Media vs. Combo	Media vs. 2500pg/mL	7.5	No	ns
Media vs. Combo	Media vs. 5000pg/mL	32.33	No	ns
FCCP vs. TNFa	8uM FCCP vs. 50pg/mL	-81.17	No	ns
FCCP vs. TNFa	8uM FCCP vs. 100pg/mL	-43.17	No	ns
FCCP vs. TNFa	8uM FCCP vs. 150pg/mL	-40.83	No	ns
FCCP vs. TNFa	8uM FCCP vs. 200pg/mL	-19	No	ns
FCCP vs. TNFa	8uM FCCP vs. 250pg/mL	-32.83	No	ns
FCCP vs. TNFa	8uM FCCP vs. 500pg/mL	-41.83	No	ns
FCCP vs. TNFa	8uM FCCP vs. 1000pg/mL	-25.17	No	ns
FCCP vs. TNFa	8uM FCCP vs. 2500pg/mL	-26.5	No	ns
FCCP vs. TNFa	8uM FCCP vs. 5000pg/mL	-21.67	No	ns
Media vs. Butyrate	8uM FCCP vs. 50uM	-88	No	ns
Media vs. Butyrate	8uM FCCP vs. 75um	-97.33	No	ns
Media vs. Butyrate	8uM FCCP vs. 100uM	-126.2	Yes	**
Media vs. Butyrate	8uM FCCP vs. 250µM	-136.4	Yes	**
Media vs. Butyrate	8uM FCCP vs. 500uM	-154.8	Yes	****
Media vs. Butyrate	8uM FCCP vs. 750uM	-157.5	Yes	**
Media vs. Butyrate	8uM FCCP vs. 1000uM	-154.4	Yes	***
Media vs. Butyrate	8uM FCCP vs. 2500uM	-122.8	Yes	*

Media vs. Butyrate	8uM FCCP vs. 5000uM	-101	No	ns
Media vs. Butyrate	8uM FCCP vs. 7500uM	-80.63	No	ns
FCCP vs. Combo	8uM FCCP vs. 50pg/mL	-142.3	Yes	***
FCCP vs. Combo	8uM FCCP vs. 100pg/mL	-126.7	Yes	**
FCCP vs. Combo	8uM FCCP vs. 150pg/mL	-135.2	Yes	**
FCCP vs. Combo	8uM FCCP vs. 200pg/mL	-133.3	Yes	**
FCCP vs. Combo	8uM FCCP vs. 250pg/mL	-125	Yes	**
FCCP vs. Combo	8uM FCCP vs. 500pg/mL	-108	No	ns
FCCP vs. Combo	8uM FCCP vs. 1000pg/mL	-83.83	No	ns
FCCP vs. Combo	8uM FCCP vs. 2500pg/mL	-56.33	No	ns
FCCP vs. Combo	8uM FCCP vs. 5000pg/mL	-31.5	No	ns
TNFa vs. TNFa	50pg/mL vs. 100pg/mL	38	No	ns
TNFa vs. TNFa	50pg/mL vs. 150pg/mL	40.33	No	ns
TNFa vs. TNFa	50pg/mL vs. 200pg/mL	62.17	No	ns
TNFa vs. TNFa	50pg/mL vs. 250pg/mL	48.33	No	ns
TNFa vs. TNFa	50pg/mL vs. 500pg/mL	39.33	No	ns
TNFa vs. TNFa	50pg/mL vs. 1000pg/mL	56	No	ns
TNFa vs. TNFa	50pg/mL vs. 2500pg/mL	54.67	No	ns
TNFa vs. TNFa	50pg/mL vs. 5000pg/mL	59.5	No	ns
TNFa vs. Butyrate	50pg/mL vs. 50uM	-6.833	No	ns
TNFa vs. Butyrate	50pg/mL vs. 75um	-16.17	No	ns
TNFa vs. Butyrate	50pg/mL vs. 100uM	-45	No	ns
TNFa vs. Butyrate	50pg/mL vs. 250µM	-55.27	No	ns
TNFa vs. Butyrate	50pg/mL vs. 500uM	-73.67	No	ns
TNFa vs. Butyrate	50pg/mL vs. 750uM	-76.33	No	ns
TNFa vs. Butyrate	50pg/mL vs. 1000uM	-73.27	No	ns
TNFa vs. Butyrate	50pg/mL vs. 2500uM	-41.67	No	ns
TNFa vs. Butyrate	50pg/mL vs. 5000uM	-19.83	No	ns
TNFa vs. Butyrate	50pg/mL vs. 7500uM	0.5333	No	ns
TNFa vs. Combo	50pg/mL vs. 50pg/mL	-61.17	No	ns
TNFa vs. Combo	50pg/mL vs. 100pg/mL	-45.5	No	ns
TNFa vs. Combo	50pg/mL vs. 150pg/mL	-54	No	ns
TNFa vs. Combo	50pg/mL vs. 200pg/mL	-52.17	No	ns
TNFa vs. Combo	50pg/mL vs. 250pg/mL	-43.83	No	ns
TNFa vs. Combo	50pg/mL vs. 500pg/mL	-26.83	No	ns
TNFa vs. Combo	50pg/mL vs. 1000pg/mL	-2.667	No	ns
TNFa vs. Combo	50pg/mL vs. 2500pg/mL	24.83	No	ns
TNFa vs. Combo	50pg/mL vs. 5000pg/mL	49.67	No	ns
TNFa vs. TNFa	100pg/mL vs. 150pg/mL	2.333	No	ns
TNFa vs. TNFa	100pg/mL vs. 200pg/mL	24.17	No	ns
TNFa vs. TNFa	100pg/mL vs. 250pg/mL	10.33	No	ns
TNFa vs. TNFa	100pg/mL vs. 500pg/mL	1.333	No	ns
TNFa vs. TNFa	100pg/mL vs. 1000pg/mL	18	No	ns
TNFa vs. TNFa	100pg/mL vs. 2500pg/mL	16.67	No	ns
TNFa vs. TNFa	100pg/mL vs. 5000pg/mL	21.5	No	ns
TNFa vs. Butyrate	100pg/mL vs. 50uM	-44.83	No	ns
TNFa vs. Butyrate	100pg/mL vs. 75um	-54.17	No	ns

TNFa vs. Butyrate	100pg/mL vs. 100uM	-83	No	ns
TNFa vs. Butyrate	100pg/mL vs. 250µM	-93.27	No	ns
TNFa vs. Butyrate	100pg/mL vs. 500uM	-111.7	No	ns
TNFa vs. Butyrate	100pg/mL vs. 750uM	-114.3	No	ns
TNFa vs. Butyrate	100pg/mL vs. 1000uM	-111.3	No	ns
TNFa vs. Butyrate	100pg/mL vs. 2500uM	-79.67	No	ns
TNFa vs. Butyrate	100pg/mL vs. 5000uM	-57.83	No	ns
TNFa vs. Butyrate	100pg/mL vs. 7500uM	-37.47	No	ns
TNFa vs. Combo	100pg/mL vs. 50pg/mL	-99.17	No	ns
TNFa vs. Combo	100pg/mL vs. 100pg/mL	-83.5	No	ns
TNFa vs. Combo	100pg/mL vs. 150pg/mL	-92	No	ns
TNFa vs. Combo	100pg/mL vs. 200pg/mL	-90.17	No	ns
TNFa vs. Combo	100pg/mL vs. 250pg/mL	-81.83	No	ns
TNFa vs. Combo	100pg/mL vs. 500pg/mL	-64.83	No	ns
TNFa vs. Combo	100pg/mL vs. 1000pg/mL	-40.67	No	ns
TNFa vs. Combo	100pg/mL vs. 2500pg/mL	-13.17	No	ns
TNFa vs. Combo	100pg/mL vs. 5000pg/mL	11.67	No	ns
TNFa vs. TNFa	150pg/mL vs. 200pg/mL	21.83	No	ns
TNFa vs. TNFa	150pg/mL vs. 250pg/mL	8	No	ns
TNFa vs. TNFa	150pg/mL vs. 500pg/mL	-1	No	ns
TNFa vs. TNFa	150pg/mL vs. 1000pg/mL	15.67	No	ns
TNFa vs. TNFa	150pg/mL vs. 2500pg/mL	14.33	No	ns
TNFa vs. TNFa	150pg/mL vs. 5000pg/mL	19.17	No	ns
TNFa vs. Butyrate	150pg/mL vs. 50uM	-47.17	No	ns
TNFa vs. Butyrate	150pg/mL vs. 75um	-56.5	No	ns
TNFa vs. Butyrate	150pg/mL vs. 100uM	-85.33	No	ns
TNFa vs. Butyrate	150pg/mL vs. 250µM	-95.6	No	ns
TNFa vs. Butyrate	150pg/mL vs. 500uM	-114	Yes	*
TNFa vs. Butyrate	150pg/mL vs. 750uM	-116.7	No	ns
TNFa vs. Butyrate	150pg/mL vs. 1000uM	-113.6	No	ns
TNFa vs. Butyrate	150pg/mL vs. 2500uM	-82	No	ns
TNFa vs. Butyrate	150pg/mL vs. 5000uM	-60.17	No	ns
TNFa vs. Butyrate	150pg/mL vs. 7500uM	-39.8	No	ns
TNFa vs. Combo	150pg/mL vs. 50pg/mL	-101.5	No	ns
TNFa vs. Combo	150pg/mL vs. 100pg/mL	-85.83	No	ns
TNFa vs. Combo	150pg/mL vs. 150pg/mL	-94.33	No	ns
TNFa vs. Combo	150pg/mL vs. 200pg/mL	-92.5	No	ns
TNFa vs. Combo	150pg/mL vs. 250pg/mL	-84.17	No	ns
TNFa vs. Combo	150pg/mL vs. 500pg/mL	-67.17	No	ns
TNFa vs. Combo	150pg/mL vs. 1000pg/mL	-43	No	ns
TNFa vs. Combo	150pg/mL vs. 2500pg/mL	-15.5	No	ns
TNFa vs. Combo	150pg/mL vs. 5000pg/mL	9.333	No	ns
TNFa vs. TNFa	200pg/mL vs. 250pg/mL	-13.83	No	ns
TNFa vs. TNFa	200pg/mL vs. 500pg/mL	-22.83	No	ns
TNFa vs. TNFa	200pg/mL vs. 1000pg/mL	-6.167	No	ns
TNFa vs. TNFa	200pg/mL vs. 2500pg/mL	-7.5	No	ns
TNFa vs. TNFa	200pg/mL vs. 5000pg/mL	-2.667	No	ns

TNFa vs. Butyrate	200pg/mL vs. 50uM	-69	No	ns
TNFa vs. Butyrate	200pg/mL vs. 75um	-78.33	No	ns
TNFa vs. Butyrate	200pg/mL vs. 100uM	-107.2	No	ns
TNFa vs. Butyrate	200pg/mL vs. 250µM	-117.4	No	ns
TNFa vs. Butyrate	200pg/mL vs. 500uM	-135.8	Yes	**
TNFa vs. Butyrate	200pg/mL vs. 750uM	-138.5	Yes	*
TNFa vs. Butyrate	200pg/mL vs. 1000uM	-135.4	Yes	**
TNFa vs. Butyrate	200pg/mL vs. 2500uM	-103.8	No	ns
TNFa vs. Butyrate	200pg/mL vs. 5000uM	-82	No	ns
TNFa vs. Butyrate	200pg/mL vs. 7500uM	-61.63	No	ns
TNFa vs. Combo	200pg/mL vs. 50pg/mL	-123.3	Yes	**
TNFa vs. Combo	200pg/mL vs. 100pg/mL	-107.7	No	ns
TNFa vs. Combo	200pg/mL vs. 150pg/mL	-116.2	Yes	*
TNFa vs. Combo	200pg/mL vs. 200pg/mL	-114.3	Yes	*
TNFa vs. Combo	200pg/mL vs. 250pg/mL	-106	No	ns
TNFa vs. Combo	200pg/mL vs. 500pg/mL	-89	No	ns
TNFa vs. Combo	200pg/mL vs. 1000pg/mL	-64.83	No	ns
TNFa vs. Combo	200pg/mL vs. 2500pg/mL	-37.33	No	ns
TNFa vs. Combo	200pg/mL vs. 5000pg/mL	-12.5	No	ns
TNFa vs. TNFa	250pg/mL vs. 500pg/mL	-9	No	ns
TNFa vs. TNFa	250pg/mL vs. 1000pg/mL	7.667	No	ns
TNFa vs. TNFa	250pg/mL vs. 2500pg/mL	6.333	No	ns
TNFa vs. TNFa	250pg/mL vs. 5000pg/mL	11.17	No	ns
TNFa vs. Butyrate	250pg/mL vs. 50uM	-55.17	No	ns
TNFa vs. Butyrate	250pg/mL vs. 75um	-64.5	No	ns
TNFa vs. Butyrate	250pg/mL vs. 100uM	-93.33	No	ns
TNFa vs. Butyrate	250pg/mL vs. 250µM	-103.6	No	ns
TNFa vs. Butyrate	250pg/mL vs. 500uM	-122	Yes	*
TNFa vs. Butyrate	250pg/mL vs. 750uM	-124.7	No	ns
TNFa vs. Butyrate	250pg/mL vs. 1000uM	-121.6	Yes	*
TNFa vs. Butyrate	250pg/mL vs. 2500uM	-90	No	ns
TNFa vs. Butyrate	250pg/mL vs. 5000uM	-68.17	No	ns
TNFa vs. Butyrate	250pg/mL vs. 7500uM	-47.8	No	ns
TNFa vs. Combo	250pg/mL vs. 50pg/mL	-109.5	No	ns
TNFa vs. Combo	250pg/mL vs. 100pg/mL	-93.83	No	ns
TNFa vs. Combo	250pg/mL vs. 150pg/mL	-102.3	No	ns
TNFa vs. Combo	250pg/mL vs. 200pg/mL	-100.5	No	ns
TNFa vs. Combo	250pg/mL vs. 250pg/mL	-92.17	No	ns
TNFa vs. Combo	250pg/mL vs. 500pg/mL	-75.17	No	ns
TNFa vs. Combo	250pg/mL vs. 1000pg/mL	-51	No	ns
TNFa vs. Combo	250pg/mL vs. 2500pg/mL	-23.5	No	ns
TNFa vs. Combo	250pg/mL vs. 5000pg/mL	1.333	No	ns
TNFa vs. TNFa	500pg/mL vs. 1000pg/mL	16.67	No	ns
TNFa vs. TNFa	500pg/mL vs. 2500pg/mL	15.33	No	ns
TNFa vs. TNFa	500pg/mL vs. 5000pg/mL	20.17	No	ns
TNFa vs. Butyrate	500pg/mL vs. 50uM	-46.17	No	ns
TNFa vs. Butyrate	500pg/mL vs. 75um	-55.5	No	ns

TNFa vs. Butyrate	500pg/mL vs. 100uM	-84.33	No	ns
TNFa vs. Butyrate	500pg/mL vs. 250μM	-94.6	No	ns
TNFa vs. Butyrate	500pg/mL vs. 500uM	-113	Yes	*
TNFa vs. Butyrate	500pg/mL vs. 750uM	-115.7	No	ns
TNFa vs. Butyrate	500pg/mL vs. 1000uM	-112.6	No	ns
TNFa vs. Butyrate	500pg/mL vs. 2500uM	-81	No	ns
TNFa vs. Butyrate	500pg/mL vs. 5000uM	-59.17	No	ns
TNFa vs. Butyrate	500pg/mL vs. 7500uM	-38.8	No	ns
TNFa vs. Combo	500pg/mL vs. 50pg/mL	-100.5	No	ns
TNFa vs. Combo	500pg/mL vs. 100pg/mL	-84.83	No	ns
TNFa vs. Combo	500pg/mL vs. 150pg/mL	-93.33	No	ns
TNFa vs. Combo	500pg/mL vs. 200pg/mL	-91.5	No	ns
TNFa vs. Combo	500pg/mL vs. 250pg/mL	-83.17	No	ns
TNFa vs. Combo	500pg/mL vs. 500pg/mL	-66.17	No	ns
TNFa vs. Combo	500pg/mL vs. 1000pg/mL	-42	No	ns
TNFa vs. Combo	500pg/mL vs. 2500pg/mL	-14.5	No	ns
TNFa vs. Combo	500pg/mL vs. 5000pg/mL	10.33	No	ns
TNFa vs. TNFa	1000pg/mL vs. 2500pg/mL	-1.333	No	ns
TNFa vs. TNFa	1000pg/mL vs. 5000pg/mL	3.5	No	ns
TNFa vs. Butyrate	1000pg/mL vs. 50uM	-62.83	No	ns
TNFa vs. Butyrate	1000pg/mL vs. 75um	-72.17	No	ns
TNFa vs. Butyrate	1000pg/mL vs. 100uM	-101	No	ns
TNFa vs. Butyrate	1000pg/mL vs. 250μM	-111.3	No	ns
TNFa vs. Butyrate	1000pg/mL vs. 500uM	-129.7	Yes	**
TNFa vs. Butyrate	1000pg/mL vs. 750uM	-132.3	No	ns
TNFa vs. Butyrate	1000pg/mL vs. 1000uM	-129.3	Yes	**
TNFa vs. Butyrate	1000pg/mL vs. 2500uM	-97.67	No	ns
TNFa vs. Butyrate	1000pg/mL vs. 5000uM	-75.83	No	ns
TNFa vs. Butyrate	1000pg/mL vs. 7500uM	-55.47	No	ns
TNFa vs. Combo	1000pg/mL vs. 50pg/mL	-117.2	Yes	*
TNFa vs. Combo	1000pg/mL vs. 100pg/mL	-101.5	No	ns
TNFa vs. Combo	1000pg/mL vs. 150pg/mL	-110	No	ns
TNFa vs. Combo	1000pg/mL vs. 200pg/mL	-108.2	No	ns
TNFa vs. Combo	1000pg/mL vs. 250pg/mL	-99.83	No	ns
TNFa vs. Combo	1000pg/mL vs. 500pg/mL	-82.83	No	ns
TNFa vs. Combo	1000pg/mL vs. 1000pg/mL	-58.67	No	ns
TNFa vs. Combo	1000pg/mL vs. 2500pg/mL	-31.17	No	ns
TNFa vs. Combo	1000pg/mL vs. 5000pg/mL	-6.333	No	ns
TNFa vs. TNFa	2500pg/mL vs. 5000pg/mL	4.833	No	ns
TNFa vs. Butyrate	2500pg/mL vs. 50uM	-61.5	No	ns
TNFa vs. Butyrate	2500pg/mL vs. 75um	-70.83	No	ns
TNFa vs. Butyrate	2500pg/mL vs. 100uM	-99.67	No	ns
TNFa vs. Butyrate	2500pg/mL vs. 250μM	-109.9	No	ns
TNFa vs. Butyrate	2500pg/mL vs. 500uM	-128.3	Yes	**
TNFa vs. Butyrate	2500pg/mL vs. 750uM	-131	No	ns
TNFa vs. Butyrate	2500pg/mL vs. 1000uM	-127.9	Yes	*
TNFa vs. Butyrate	2500pg/mL vs. 2500uM	-96.33	No	ns

TNFa vs. Butyrate	2500pg/mL vs. 5000uM	-74.5	No	ns
TNFa vs. Butyrate	2500pg/mL vs. 7500uM	-54.13	No	ns
TNFa vs. Combo	2500pg/mL vs. 50pg/mL	-115.8	Yes	*
TNFa vs. Combo	2500pg/mL vs. 100pg/mL	-100.2	No	ns
TNFa vs. Combo	2500pg/mL vs. 150pg/mL	-108.7	No	ns
TNFa vs. Combo	2500pg/mL vs. 200pg/mL	-106.8	No	ns
TNFa vs. Combo	2500pg/mL vs. 250pg/mL	-98.5	No	ns
TNFa vs. Combo	2500pg/mL vs. 500pg/mL	-81.5	No	ns
TNFa vs. Combo	2500pg/mL vs. 1000pg/mL	-57.33	No	ns
TNFa vs. Combo	2500pg/mL vs. 2500pg/mL	-29.83	No	ns
TNFa vs. Combo	2500pg/mL vs. 5000pg/mL	-5	No	ns
TNFa vs. Butyrate	5000pg/mL vs. 50uM	-66.33	No	ns
TNFa vs. Butyrate	5000pg/mL vs. 75um	-75.67	No	ns
TNFa vs. Butyrate	5000pg/mL vs. 100uM	-104.5	No	ns
TNFa vs. Butyrate	5000pg/mL vs. 250μM	-114.8	No	ns
TNFa vs. Butyrate	5000pg/mL vs. 500uM	-133.2	Yes	**
TNFa vs. Butyrate	5000pg/mL vs. 750uM	-135.8	No	ns
TNFa vs. Butyrate	5000pg/mL vs. 1000uM	-132.8	Yes	**
TNFa vs. Butyrate	5000pg/mL vs. 2500uM	-101.2	No	ns
TNFa vs. Butyrate	5000pg/mL vs. 5000uM	-79.33	No	ns
TNFa vs. Butyrate	5000pg/mL vs. 7500uM	-58.97	No	ns
TNFa vs. Combo	5000pg/mL vs. 50pg/mL	-120.7	Yes	*
TNFa vs. Combo	5000pg/mL vs. 100pg/mL	-105	No	ns
TNFa vs. Combo	5000pg/mL vs. 150pg/mL	-113.5	Yes	*
TNFa vs. Combo	5000pg/mL vs. 200pg/mL	-111.7	No	ns
TNFa vs. Combo	5000pg/mL vs. 250pg/mL	-103.3	No	ns
TNFa vs. Combo	5000pg/mL vs. 500pg/mL	-86.33	No	ns
TNFa vs. Combo	5000pg/mL vs. 1000pg/mL	-62.17	No	ns
TNFa vs. Combo	5000pg/mL vs. 2500pg/mL	-34.67	No	ns
TNFa vs. Combo	5000pg/mL vs. 5000pg/mL	-9.833	No	ns
Butyrate vs. Butyrate	50uM vs. 75um	-9.333	No	ns
Butyrate vs. Butyrate	50uM vs. 100uM	-38.17	No	ns
Butyrate vs. Butyrate	50uM vs. 250μM	-48.43	No	ns
Butyrate vs. Butyrate	50uM vs. 500uM	-66.83	No	ns
Butyrate vs. Butyrate	50uM vs. 750uM	-69.5	No	ns
Butyrate vs. Butyrate	50uM vs. 1000uM	-66.43	No	ns
Butyrate vs. Butyrate	50uM vs. 2500uM	-34.83	No	ns
Butyrate vs. Butyrate	50uM vs. 5000uM	-13	No	ns
Butyrate vs. Butyrate	50uM vs. 7500uM	7.367	No	ns
Butyrate vs. Combo	50uM vs. 50pg/mL	-54.33	No	ns
Butyrate vs. Combo	50uM vs. 100pg/mL	-38.67	No	ns
Butyrate vs. Combo	50uM vs. 150pg/mL	-47.17	No	ns
Butyrate vs. Combo	50uM vs. 200pg/mL	-45.33	No	ns
Butyrate vs. Combo	50uM vs. 250pg/mL	-37	No	ns
Butyrate vs. Combo	50uM vs. 500pg/mL	-20	No	ns
Butyrate vs. Combo	50uM vs. 1000pg/mL	4.167	No	ns
Butyrate vs. Combo	50uM vs. 2500pg/mL	31.67	No	ns

Butyrate vs. Combo	50uM vs. 5000pg/mL	56.5	No	ns
Butyrate vs. Butyrate	75um vs. 100uM	-28.83	No	ns
Butyrate vs. Butyrate	75um vs. 250µM	-39.1	No	ns
Butyrate vs. Butyrate	75um vs. 500uM	-57.5	No	ns
Butyrate vs. Butyrate	75um vs. 750uM	-60.17	No	ns
Butyrate vs. Butyrate	75um vs. 1000uM	-57.1	No	ns
Butyrate vs. Butyrate	75um vs. 2500uM	-25.5	No	ns
Butyrate vs. Butyrate	75um vs. 5000uM	-3.667	No	ns
Butyrate vs. Butyrate	75um vs. 7500uM	16.7	No	ns
Butyrate vs. Combo	75um vs. 50pg/mL	-45	No	ns
Butyrate vs. Combo	75um vs. 100pg/mL	-29.33	No	ns
Butyrate vs. Combo	75um vs. 150pg/mL	-37.83	No	ns
Butyrate vs. Combo	75um vs. 200pg/mL	-36	No	ns
Butyrate vs. Combo	75um vs. 250pg/mL	-27.67	No	ns
Butyrate vs. Combo	75um vs. 500pg/mL	-10.67	No	ns
Butyrate vs. Combo	75um vs. 1000pg/mL	13.5	No	ns
Butyrate vs. Combo	75um vs. 2500pg/mL	41	No	ns
Butyrate vs. Combo	75um vs. 5000pg/mL	65.83	No	ns
Butyrate vs. Butyrate	100uM vs. 250µM	-10.27	No	ns
Butyrate vs. Butyrate	100uM vs. 500uM	-28.67	No	ns
Butyrate vs. Butyrate	100uM vs. 750uM	-31.33	No	ns
Butyrate vs. Butyrate	100uM vs. 1000uM	-28.27	No	ns
Butyrate vs. Butyrate	100uM vs. 2500uM	3.333	No	ns
Butyrate vs. Butyrate	100uM vs. 5000uM	25.17	No	ns
Butyrate vs. Butyrate	100uM vs. 7500uM	45.53	No	ns
Butyrate vs. Combo	100uM vs. 50pg/mL	-16.17	No	ns
Butyrate vs. Combo	100uM vs. 100pg/mL	-0.5	No	ns
Butyrate vs. Combo	100uM vs. 150pg/mL	-9	No	ns
Butyrate vs. Combo	100uM vs. 200pg/mL	-7.167	No	ns
Butyrate vs. Combo	100uM vs. 250pg/mL	1.167	No	ns
Butyrate vs. Combo	100uM vs. 500pg/mL	18.17	No	ns
Butyrate vs. Combo	100uM vs. 1000pg/mL	42.33	No	ns
Butyrate vs. Combo	100uM vs. 2500pg/mL	69.83	No	ns
Butyrate vs. Combo	100uM vs. 5000pg/mL	94.67	No	ns
Butyrate vs. Butyrate	250µM vs. 500uM	-18.4	No	ns
Butyrate vs. Butyrate	250µM vs. 750uM	-21.07	No	ns
Butyrate vs. Butyrate	250µM vs. 1000uM	-18	No	ns
Butyrate vs. Butyrate	250µM vs. 2500uM	13.6	No	ns
Butyrate vs. Butyrate	250µM vs. 5000uM	35.43	No	ns
Butyrate vs. Butyrate	250µM vs. 7500uM	55.8	No	ns
Butyrate vs. Combo	250µM vs. 50pg/mL	-5.9	No	ns
Butyrate vs. Combo	250µM vs. 100pg/mL	9.767	No	ns
Butyrate vs. Combo	250µM vs. 150pg/mL	1.267	No	ns
Butyrate vs. Combo	250µM vs. 200pg/mL	3.1	No	ns
Butyrate vs. Combo	250µM vs. 250pg/mL	11.43	No	ns
Butyrate vs. Combo	250µM vs. 500pg/mL	28.43	No	ns
Butyrate vs. Combo	250µM vs. 1000pg/mL	52.6	No	ns

Butyrate vs. Combo	250µM vs. 2500pg/mL	80.1	No	ns
Butyrate vs. Combo	250µM vs. 5000pg/mL	104.9	No	ns
Butyrate vs. Butyrate	500uM vs. 750uM	-2.667	No	ns
Butyrate vs. Butyrate	500uM vs. 1000uM	0.4	No	ns
Butyrate vs. Butyrate	500uM vs. 2500uM	32	No	ns
Butyrate vs. Butyrate	500uM vs. 5000uM	53.83	No	ns
Butyrate vs. Butyrate	500uM vs. 7500uM	74.2	No	ns
Butyrate vs. Combo	500uM vs. 50pg/mL	12.5	No	ns
Butyrate vs. Combo	500uM vs. 100pg/mL	28.17	No	ns
Butyrate vs. Combo	500uM vs. 150pg/mL	19.67	No	ns
Butyrate vs. Combo	500uM vs. 200pg/mL	21.5	No	ns
Butyrate vs. Combo	500uM vs. 250pg/mL	29.83	No	ns
Butyrate vs. Combo	500uM vs. 500pg/mL	46.83	No	ns
Butyrate vs. Combo	500uM vs. 1000pg/mL	71	No	ns
Butyrate vs. Combo	500uM vs. 2500pg/mL	98.5	No	ns
Butyrate vs. Combo	500uM vs. 5000pg/mL	123.3	Yes	**
Butyrate vs. Butyrate	750uM vs. 1000uM	3.067	No	ns
Butyrate vs. Butyrate	750uM vs. 2500uM	34.67	No	ns
Butyrate vs. Butyrate	750uM vs. 5000uM	56.5	No	ns
Butyrate vs. Butyrate	750uM vs. 7500uM	76.87	No	ns
Butyrate vs. Combo	750uM vs. 50pg/mL	15.17	No	ns
Butyrate vs. Combo	750uM vs. 100pg/mL	30.83	No	ns
Butyrate vs. Combo	750uM vs. 150pg/mL	22.33	No	ns
Butyrate vs. Combo	750uM vs. 200pg/mL	24.17	No	ns
Butyrate vs. Combo	750uM vs. 250pg/mL	32.5	No	ns
Butyrate vs. Combo	750uM vs. 500pg/mL	49.5	No	ns
Butyrate vs. Combo	750uM vs. 1000pg/mL	73.67	No	ns
Butyrate vs. Combo	750uM vs. 2500pg/mL	101.2	No	ns
Butyrate vs. Combo	750uM vs. 5000pg/mL	126	No	ns
Butyrate vs. Butyrate	1000uM vs. 2500uM	31.6	No	ns
Butyrate vs. Butyrate	1000uM vs. 5000uM	53.43	No	ns
Butyrate vs. Butyrate	1000uM vs. 7500uM	73.8	No	ns
Butyrate vs. Combo	1000uM vs. 50pg/mL	12.1	No	ns
Butyrate vs. Combo	1000uM vs. 100pg/mL	27.77	No	ns
Butyrate vs. Combo	1000uM vs. 150pg/mL	19.27	No	ns
Butyrate vs. Combo	1000uM vs. 200pg/mL	21.1	No	ns
Butyrate vs. Combo	1000uM vs. 250pg/mL	29.43	No	ns
Butyrate vs. Combo	1000uM vs. 500pg/mL	46.43	No	ns
Butyrate vs. Combo	1000uM vs. 1000pg/mL	70.6	No	ns
Butyrate vs. Combo	1000uM vs. 2500pg/mL	98.1	No	ns
Butyrate vs. Combo	1000uM vs. 5000pg/mL	122.9	Yes	*
Butyrate vs. Butyrate	2500uM vs. 5000uM	21.83	No	ns
Butyrate vs. Butyrate	2500uM vs. 7500uM	42.2	No	ns
Butyrate vs. Combo	2500uM vs. 50pg/mL	-19.5	No	ns
Butyrate vs. Combo	2500uM vs. 100pg/mL	-3.833	No	ns
Butyrate vs. Combo	2500uM vs. 150pg/mL	-12.33	No	ns
Butyrate vs. Combo	2500uM vs. 200pg/mL	-10.5	No	ns

Butyrate vs. Combo	2500uM vs. 250pg/mL	-2.167	No	ns
Butyrate vs. Combo	2500uM vs. 500pg/mL	14.83	No	ns
Butyrate vs. Combo	2500uM vs. 1000pg/mL	39	No	ns
Butyrate vs. Combo	2500uM vs. 2500pg/mL	66.5	No	ns
Butyrate vs. Combo	2500uM vs. 5000pg/mL	91.33	No	ns
Butyrate vs. Butyrate	5000uM vs. 7500uM	20.37	No	ns
Butyrate vs. Combo	5000uM vs. 50pg/mL	-41.33	No	ns
Butyrate vs. Combo	5000uM vs. 100pg/mL	-25.67	No	ns
Butyrate vs. Combo	5000uM vs. 150pg/mL	-34.17	No	ns
Butyrate vs. Combo	5000uM vs. 200pg/mL	-32.33	No	ns
Butyrate vs. Combo	5000uM vs. 250pg/mL	-24	No	ns
Butyrate vs. Combo	5000uM vs. 500pg/mL	-7	No	ns
Butyrate vs. Combo	5000uM vs. 1000pg/mL	17.17	No	ns
Butyrate vs. Combo	5000uM vs. 2500pg/mL	44.67	No	ns
Butyrate vs. Combo	5000uM vs. 5000pg/mL	69.5	No	ns
Butyrate vs. Combo	7500uM vs. 50pg/mL	-61.7	No	ns
Butyrate vs. Combo	7500uM vs. 100pg/mL	-46.03	No	ns
Butyrate vs. Combo	7500uM vs. 150pg/mL	-54.53	No	ns
Butyrate vs. Combo	7500uM vs. 200pg/mL	-52.7	No	ns
Butyrate vs. Combo	7500uM vs. 250pg/mL	-44.37	No	ns
Butyrate vs. Combo	7500uM vs. 500pg/mL	-27.37	No	ns
Butyrate vs. Combo	7500uM vs. 1000pg/mL	-3.2	No	ns
Butyrate vs. Combo	7500uM vs. 2500pg/mL	24.3	No	ns
Butyrate vs. Combo	7500uM vs. 5000pg/mL	49.13	No	ns
Combo vs. Combo	50pg/mL vs. 100pg/mL	15.67	No	ns
Combo vs. Combo	50pg/mL vs. 150pg/mL	7.167	No	ns
Combo vs. Combo	50pg/mL vs. 200pg/mL	9	No	ns
Combo vs. Combo	50pg/mL vs. 250pg/mL	17.33	No	ns
Combo vs. Combo	50pg/mL vs. 500pg/mL	34.33	No	ns
Combo vs. Combo	50pg/mL vs. 1000pg/mL	58.5	No	ns
Combo vs. Combo	50pg/mL vs. 2500pg/mL	86	No	ns
Combo vs. Combo	50pg/mL vs. 5000pg/mL	110.8	No	ns
Combo vs. Combo	100pg/mL vs. 150pg/mL	-8.5	No	ns
Combo vs. Combo	100pg/mL vs. 200pg/mL	-6.667	No	ns
Combo vs. Combo	100pg/mL vs. 250pg/mL	1.667	No	ns
Combo vs. Combo	100pg/mL vs. 500pg/mL	18.67	No	ns
Combo vs. Combo	100pg/mL vs. 1000pg/mL	42.83	No	ns
Combo vs. Combo	100pg/mL vs. 2500pg/mL	70.33	No	ns
Combo vs. Combo	100pg/mL vs. 5000pg/mL	95.17	No	ns
Combo vs. Combo	150pg/mL vs. 200pg/mL	1.833	No	ns
Combo vs. Combo	150pg/mL vs. 250pg/mL	10.17	No	ns
Combo vs. Combo	150pg/mL vs. 500pg/mL	27.17	No	ns
Combo vs. Combo	150pg/mL vs. 1000pg/mL	51.33	No	ns
Combo vs. Combo	150pg/mL vs. 2500pg/mL	78.83	No	ns
Combo vs. Combo	150pg/mL vs. 5000pg/mL	103.7	No	ns
Combo vs. Combo	200pg/mL vs. 250pg/mL	8.333	No	ns
Combo vs. Combo	200pg/mL vs. 500pg/mL	25.33	No	ns

Combo vs. Combo	200pg/mL vs. 1000pg/mL	49.5	No	ns
Combo vs. Combo	200pg/mL vs. 2500pg/mL	77	No	ns
Combo vs. Combo	200pg/mL vs. 5000pg/mL	101.8	No	ns
Combo vs. Combo	250pg/mL vs. 500pg/mL	17	No	ns
Combo vs. Combo	250pg/mL vs. 1000pg/mL	41.17	No	ns
Combo vs. Combo	250pg/mL vs. 2500pg/mL	68.67	No	ns
Combo vs. Combo	250pg/mL vs. 5000pg/mL	93.5	No	ns
Combo vs. Combo	500pg/mL vs. 1000pg/mL	24.17	No	ns
Combo vs. Combo	500pg/mL vs. 2500pg/mL	51.67	No	ns
Combo vs. Combo	500pg/mL vs. 5000pg/mL	76.5	No	ns
Combo vs. Combo	1000pg/mL vs. 2500pg/mL	27.5	No	ns
Combo vs. Combo	1000pg/mL vs. 5000pg/mL	52.33	No	ns
Combo vs. Combo	2500pg/mL vs. 5000pg/mL	24.83	No	ns

A-B
A-C
A-D
A-E
A-F
A-G
A-H
A-I
A-J
A-K
A-L
A-M
A-N
A-O
A-P
A-Q
A-R
A-S
A-T
A-U
A-V
A-W
A-X
A-Y
A-Z
A-AA
A-AB
A-AC
A-AD
B-C
B-D
B-E
B-F
B-G
B-H
B-I
B-J
B-K
B-L
B-M
B-N
B-O
B-P
B-Q
B-R
B-S

B-T
B-U
B-V
B-W
B-X
B-Y
B-Z
B-AA
B-AB
B-AC
B-AD
C-D
C-E
C-F
C-G
C-H
C-I
C-J
C-K
C-L
C-M
C-N
C-O
C-P
C-Q
C-R
C-S
C-T
C-U
C-V
C-W
C-X
C-Y
C-Z
C-AA
C-AB
C-AC
C-AD
D-E
D-F
D-G
D-H
D-I
D-J
D-K
D-L
D-M

D-N
D-O
D-P
D-Q
D-R
D-S
D-T
D-U
D-V
D-W
D-X
D-Y
D-Z
D-AA
D-AB
D-AC
D-AD
E-F
E-G
E-H
E-I
E-J
E-K
E-L
E-M
E-N
E-O
E-P
E-Q
E-R
E-S
E-T
E-U
E-V
E-W
E-X
E-Y
E-Z
E-AA
E-AB
E-AC
E-AD
F-G
F-H
F-I
F-J
F-K

F-L
F-M
F-N
F-O
F-P
F-Q
F-R
F-S
F-T
F-U
F-V
F-W
F-X
F-Y
F-Z
F-AA
F-AB
F-AC
F-AD
G-H
G-I
G-J
G-K
G-L
G-M
G-N
G-O
G-P
G-Q
G-R
G-S
G-T
G-U
G-V
G-W
G-X
G-Y
G-Z
G-AA
G-AB
G-AC
G-AD
H-I
H-J
H-K
H-L
H-M

H-N
H-O
H-P
H-Q
H-R
H-S
H-T
H-U
H-V
H-W
H-X
H-Y
H-Z
H-AA
H-AB
H-AC
H-AD
I-J
I-K
I-L
I-M
I-N
I-O
I-P
I-Q
I-R
I-S
I-T
I-U
I-V
I-W
I-X
I-Y
I-Z
I-AA
I-AB
I-AC
I-AD
J-K
J-L
J-M
J-N
J-O
J-P
J-Q
J-R
J-S

J-T
J-U
J-V
J-W
J-X
J-Y
J-Z
J-AA
J-AB
J-AC
J-AD
K-L
K-M
K-N
K-O
K-P
K-Q
K-R
K-S
K-T
K-U
K-V
K-W
K-X
K-Y
K-Z
K-AA
K-AB
K-AC
K-AD
L-M
L-N
L-O
L-P
L-Q
L-R
L-S
L-T
L-U
L-V
L-W
L-X
L-Y
L-Z
L-AA
L-AB
L-AC

L-AD
M-N
M-O
M-P
M-Q
M-R
M-S
M-T
M-U
M-V
M-W
M-X
M-Y
M-Z
M-AA
M-AB
M-AC
M-AD
N-O
N-P
N-Q
N-R
N-S
N-T
N-U
N-V
N-W
N-X
N-Y
N-Z
N-AA
N-AB
N-AC
N-AD
O-P
O-Q
O-R
O-S
O-T
O-U
O-V
O-W
O-X
O-Y
O-Z
O-AA
O-AB

O-AC
O-AD
P-Q
P-R
P-S
P-T
P-U
P-V
P-W
P-X
P-Y
P-Z
P-AA
P-AB
P-AC
P-AD
Q-R
Q-S
Q-T
Q-U
Q-V
Q-W
Q-X
Q-Y
Q-Z
Q-AA
Q-AB
Q-AC
Q-AD
R-S
R-T
R-U
R-V
R-W
R-X
R-Y
R-Z
R-AA
R-AB
R-AC
R-AD
S-T
S-U
S-V
S-W
S-X
S-Y

S-Z
S-AA
S-AB
S-AC
S-AD
T-U
T-V
T-W
T-X
T-Y
T-Z
T-AA
T-AB
T-AC
T-AD
U-V
U-W
U-X
U-Y
U-Z
U-AA
U-AB
U-AC
U-AD
V-W
V-X
V-Y
V-Z
V-AA
V-AB
V-AC
V-AD
W-X
W-Y
W-Z
W-AA
W-AB
W-AC
W-AD
X-Y
X-Z
X-AA
X-AB
X-AC
X-AD
Y-Z
Y-AA

Y-AB
Y-AC
Y-AD
Z-AA
Z-AB
Z-AC
Z-AD
AA-AB
AA-AC
AA-AD
AB-AC
AB-AD
AC-AD

Treatment	Dunn's multiple comparisons test	Mean rank	Significant'	Summary
Media vs. TNFa	Media vs. 250 pg/mL	-29.67	No	ns
Media vs. TNFa	Media vs. 500 pg/mL	-41.17	No	ns
Media vs. TNFa	Media vs. 750 pg/mL	-32	No	ns
Media vs. TNFa	Media vs. 1000 pg/mL	-26.17	No	ns
Media vs. Butyrate	Media vs. 500 µM	28.83	No	ns
Media vs. Butyrate	Media vs. 1000 µM	22.67	No	ns
Media vs. Butyrate	Media vs. 2500 µM	-3.833	No	ns
Media vs. Butyrate	Media vs. 5000 µM	-6.5	No	ns
Media vs. Combo	Media vs. 250 pg/mL	-12.33	No	ns
Media vs. Combo	Media vs. 500 pg/mL	-22	No	ns
Media vs. Combo	Media vs. 750 pg/mL	-5.667	No	ns
Media vs. Combo	Media vs. 1000 pg/mL	10.83	No	ns
TNFa vs. TNFa	250 pg/mL vs. 500 pg/mL	-11.5	No	ns
TNFa vs. TNFa	250 pg/mL vs. 750 pg/mL	-2.333	No	ns
TNFa vs. TNFa	250 pg/mL vs. 1000 pg/mL	3.5	No	ns
TNFa vs. Butyrate	250 pg/mL vs. 500 µM	58.5	Yes	**
TNFa vs. Butyrate	250 pg/mL vs. 1000 µM	52.33	Yes	**
TNFa vs. Butyrate	250 pg/mL vs. 2500 µM	25.83	No	ns
TNFa vs. Butyrate	250 pg/mL vs. 5000 µM	23.17	No	ns
TNFa vs. Combo	250 pg/mL vs. 250 pg/mL	17.33	No	ns
TNFa vs. Combo	250 pg/mL vs. 500 pg/mL	7.667	No	ns
TNFa vs. Combo	250 pg/mL vs. 750 pg/mL	24	No	ns
TNFa vs. Combo	250 pg/mL vs. 1000 pg/mL	40.5	No	ns
TNFa vs. TNFa	500 pg/mL vs. 750 pg/mL	9.167	No	ns
TNFa vs. TNFa	500 pg/mL vs. 1000 pg/mL	15	No	ns
TNFa vs. Butyrate	500 pg/mL vs. 500 µM	70	Yes	****
TNFa vs. Butyrate	500 pg/mL vs. 1000 µM	63.83	Yes	***
TNFa vs. Butyrate	500 pg/mL vs. 2500 µM	37.33	No	ns
TNFa vs. Butyrate	500 pg/mL vs. 5000 µM	34.67	No	ns
TNFa vs. Combo	500 pg/mL vs. 250 pg/mL	28.83	No	ns
TNFa vs. Combo	500 pg/mL vs. 500 pg/mL	19.17	No	ns
TNFa vs. Combo	500 pg/mL vs. 750 pg/mL	35.5	No	ns
TNFa vs. Combo	500 pg/mL vs. 1000 pg/mL	52	Yes	*
TNFa vs. TNFa	750 pg/mL vs. 1000 pg/mL	5.833	No	ns
TNFa vs. Butyrate	750 pg/mL vs. 500 µM	60.83	Yes	***
TNFa vs. Butyrate	750 pg/mL vs. 1000 µM	54.67	Yes	**
TNFa vs. Butyrate	750 pg/mL vs. 2500 µM	28.17	No	ns
TNFa vs. Butyrate	750 pg/mL vs. 5000 µM	25.5	No	ns
TNFa vs. Combo	750 pg/mL vs. 250 pg/mL	19.67	No	ns
TNFa vs. Combo	750 pg/mL vs. 500 pg/mL	10	No	ns
TNFa vs. Combo	750 pg/mL vs. 750 pg/mL	26.33	No	ns
TNFa vs. Combo	750 pg/mL vs. 1000 pg/mL	42.83	No	ns
TNFa vs. Butyrate	1000 pg/mL vs. 500 µM	55	Yes	**
TNFa vs. Butyrate	1000 pg/mL vs. 1000 µM	48.83	Yes	*
TNFa vs. Butyrate	1000 pg/mL vs. 2500 µM	22.33	No	ns
TNFa vs. Butyrate	1000 pg/mL vs. 5000 µM	19.67	No	ns

TNFa vs. Combo	1000 pg/mL vs. 250 pg/mL	13.83	No	ns
TNFa vs. Combo	1000 pg/mL vs. 500 pg/mL	4.167	No	ns
TNFa vs. Combo	1000 pg/mL vs. 750 pg/mL	20.5	No	ns
TNFa vs. Combo	1000 pg/mL vs. 1000 pg/mL	37	No	ns
Butyrate vs. Butyrate	500 µM vs. 1000 µM	-6.167	No	ns
Butyrate vs. Butyrate	500 µM vs. 2500 µM	-32.67	No	ns
Butyrate vs. Butyrate	500 µM vs. 5000 µM	-35.33	No	ns
Butyrate vs. Combo	500 µM vs. 250 pg/mL	-41.17	No	ns
Butyrate vs. Combo	500 µM vs. 500 pg/mL	-50.83	Yes	*
Butyrate vs. Combo	500 µM vs. 750 pg/mL	-34.5	No	ns
Butyrate vs. Combo	500 µM vs. 1000 pg/mL	-18	No	ns
Butyrate vs. Butyrate	1000 µM vs. 2500 µM	-26.5	No	ns
Butyrate vs. Butyrate	1000 µM vs. 5000 µM	-29.17	No	ns
Butyrate vs. Combo	1000 µM vs. 250 pg/mL	-35	No	ns
Butyrate vs. Combo	1000 µM vs. 500 pg/mL	-44.67	No	ns
Butyrate vs. Combo	1000 µM vs. 750 pg/mL	-28.33	No	ns
Butyrate vs. Combo	1000 µM vs. 1000 pg/mL	-11.83	No	ns
Butyrate vs. Butyrate	2500 µM vs. 5000 µM	-2.667	No	ns
Butyrate vs. Combo	2500 µM vs. 250 pg/mL	-8.5	No	ns
Butyrate vs. Combo	2500 µM vs. 500 pg/mL	-18.17	No	ns
Butyrate vs. Combo	2500 µM vs. 750 pg/mL	-1.833	No	ns
Butyrate vs. Combo	2500 µM vs. 1000 pg/mL	14.67	No	ns
Butyrate vs. Combo	5000 µM vs. 250 pg/mL	-5.833	No	ns
Butyrate vs. Combo	5000 µM vs. 500 pg/mL	-15.5	No	ns
Butyrate vs. Combo	5000 µM vs. 750 pg/mL	0.8333	No	ns
Butyrate vs. Combo	5000 µM vs. 1000 pg/mL	17.33	No	ns
Combo vs. Combo	250 pg/mL vs. 500 pg/mL	-9.667	No	ns
Combo vs. Combo	250 pg/mL vs. 750 pg/mL	6.667	No	ns
Combo vs. Combo	250 pg/mL vs. 1000 pg/mL	23.17	No	ns
Combo vs. Combo	500 pg/mL vs. 750 pg/mL	16.33	No	ns
Combo vs. Combo	500 pg/mL vs. 1000 pg/mL	32.83	No	ns
Combo vs. Combo	750 pg/mL vs. 1000 pg/mL	16.5	No	ns

A-C
A-D
A-E
A-F
A-G
A-H
A-I
A-J
A-K
A-L
A-M
A-N
C-D
C-E
C-F
C-G
C-H
C-I
C-J
C-K
C-L
C-M
C-N
D-E
D-F
D-G
D-H
D-I
D-J
D-K
D-L
D-M
D-N
E-F
E-G
E-H
E-I
E-J
E-K
E-L
E-M
E-N
F-G
F-H
F-I
F-J

F-K
F-L
F-M
F-N
G-H
G-I
G-J
G-K
G-L
G-M
G-N
H-I
H-J
H-K
H-L
H-M
H-N
I-J
I-K
I-L
I-M
I-N
J-K
J-L
J-M
J-N
K-L
K-M
K-N
L-M
L-N
M-N

Treatment	Dunn's multiple comparisons test	Mean rank	Significant?	Summary	Adjusted P
Media vs. Men	Media vs. 50µM MEN	-101.8	No	ns	0.3096
Media vs. TNFa	Media vs. 50pg/mL	4.667	No	ns	>0.9999
Media vs. TNFa	Media vs. 100pg/mL	39	No	ns	>0.9999
Media vs. TNFa	Media vs. 150pg/mL	45.92	No	ns	>0.9999
Media vs. TNFa	Media vs. 200pg/mL	30.17	No	ns	>0.9999
Media vs. TNFa	Media vs. 250pg/mL	58.17	No	ns	>0.9999
Media vs. TNFa	Media vs. 500pg/mL	44.67	No	ns	>0.9999
Media vs. TNFa	Media vs. 1000pg/mL	-63	No	ns	>0.9999
Media vs. TNFa	Media vs. 2500pg/mL	-25	No	ns	>0.9999
Media vs. TNFa	Media vs. 5000pg/mL	10.33	No	ns	>0.9999
Media vs. Butyrate	Media vs. 50µM	-11.83	No	ns	>0.9999
Media vs. Butyrate	Media vs. 75µM	12.5	No	ns	>0.9999
Media vs. Butyrate	Media vs. 100µM	20.83	No	ns	>0.9999
Media vs. Butyrate	Media vs. 250µM	56.33	No	ns	>0.9999
Media vs. Butyrate	Media vs. 500µM	1.5	No	ns	>0.9999
Media vs. Butyrate	Media vs. 750µM	-74.83	No	ns	>0.9999
Media vs. Butyrate	Media vs. 1000µM	-69.17	No	ns	>0.9999
Media vs. Butyrate	Media vs. 2500µM	-56.58	No	ns	>0.9999
Media vs. Butyrate	Media vs. 5000µM	-52.67	No	ns	>0.9999
Media vs. Butyrate	Media vs. 7500µM	-24.83	No	ns	>0.9999
Media vs. Combo	Media vs. 50pg/mL	-12.33	No	ns	>0.9999
Media vs. Combo	Media vs. 100pg/mL	41.58	No	ns	>0.9999
Media vs. Combo	Media vs. 150pg/mL	45.83	No	ns	>0.9999
Media vs. Combo	Media vs. 200pg/mL	-78	No	ns	>0.9999
Media vs. Combo	Media vs. 250pg/mL	-76.17	No	ns	>0.9999
Media vs. Combo	Media vs. 500pg/mL	-74.5	No	ns	>0.9999
Media vs. Combo	Media vs. 1000pg/mL	-53.25	No	ns	>0.9999
Media vs. Combo	Media vs. 2500pg/mL	-47.33	No	ns	>0.9999
Media vs. Combo	Media vs. 5000pg/mL	-40.17	No	ns	>0.9999
MEN vs. TNFa	50µM MEN vs. 50pg/mL	106.5	No	ns	0.1739
MEN vs. TNFa	50µM MEN vs. 100pg/mL	140.8	Yes	**	0.0012
MEN vs. TNFa	50µM MEN vs. 150pg/mL	147.8	Yes	***	0.0004
MEN vs. TNFa	50µM MEN vs. 200pg/mL	132	Yes	**	0.005
MEN vs. TNFa	50µM MEN vs. 250pg/mL	160	Yes	****	<0.0001
MEN vs. TNFa	50µM MEN vs. 500pg/mL	146.5	Yes	***	0.0005
MEN vs. TNFa	50µM MEN vs. 1000pg/mL	38.83	No	ns	>0.9999
MEN vs. TNFa	50µM MEN vs. 2500pg/mL	76.83	No	ns	>0.9999
MEN vs. TNFa	50µM MEN vs. 5000pg/mL	112.2	No	ns	0.0838
MEN vs. Butyrate	50µM MEN vs. 50µM	90	No	ns	>0.9999
MEN vs. Butyrate	50µM MEN vs. 75µM	114.3	No	ns	0.0628
MEN vs. Butyrate	50µM MEN vs. 100µM	122.7	Yes	*	0.0198
MEN vs. Butyrate	50µM MEN vs. 250µM	158.2	Yes	****	<0.0001
MEN vs. Butyrate	50µM MEN vs. 500µM	103.3	No	ns	0.2578
MEN vs. Butyrate	50µM MEN vs. 750µM	27	No	ns	>0.9999
MEN vs. Butyrate	50µM MEN vs. 1000µM	32.67	No	ns	>0.9999
MEN vs. Butyrate	50µM MEN vs. 2500µM	45.25	No	ns	>0.9999

MEN vs. Butyrate	50µM MEN vs. 5000µM	49.17	No	ns	>0.9999
MEN vs. Butyrate	50µM MEN vs. 7500µM	77	No	ns	>0.9999
MEN vs. Combo	50µM MEN vs. 50pg/mL	89.5	No	ns	>0.9999
MEN vs. Combo	50µM MEN vs. 100pg/mL	143.4	Yes	***	0.0008
MEN vs. Combo	50µM MEN vs. 150pg/mL	147.7	Yes	***	0.0004
MEN vs. Combo	50µM MEN vs. 200pg/mL	23.83	No	ns	>0.9999
MEN vs. Combo	50µM MEN vs. 250pg/mL	25.67	No	ns	>0.9999
MEN vs. Combo	50µM MEN vs. 500pg/mL	27.33	No	ns	>0.9999
MEN vs. Combo	50µM MEN vs. 1000pg/mL	48.58	No	ns	>0.9999
MEN vs. Combo	50µM MEN vs. 2500pg/mL	54.5	No	ns	>0.9999
MEN vs. Combo	50µM MEN vs. 5000pg/mL	61.67	No	ns	>0.9999
TNFa vs. TNFa	50pg/mL vs. 100pg/mL	34.33	No	ns	>0.9999
TNFa vs. TNFa	50pg/mL vs. 150pg/mL	41.25	No	ns	>0.9999
TNFa vs. TNFa	50pg/mL vs. 200pg/mL	25.5	No	ns	>0.9999
TNFa vs. TNFa	50pg/mL vs. 250pg/mL	53.5	No	ns	>0.9999
TNFa vs. TNFa	50pg/mL vs. 500pg/mL	40	No	ns	>0.9999
TNFa vs. TNFa	50pg/mL vs. 1000pg/mL	-67.67	No	ns	>0.9999
TNFa vs. TNFa	50pg/mL vs. 2500pg/mL	-29.67	No	ns	>0.9999
TNFa vs. TNFa	50pg/mL vs. 5000pg/mL	5.667	No	ns	>0.9999
TNFa vs. Butyrate	50pg/mL vs. 50µM	-16.5	No	ns	>0.9999
TNFa vs. Butyrate	50pg/mL vs. 75µM	7.833	No	ns	>0.9999
TNFa vs. Butyrate	50pg/mL vs. 100µM	16.17	No	ns	>0.9999
TNFa vs. Butyrate	50pg/mL vs. 250µM	51.67	No	ns	>0.9999
TNFa vs. Butyrate	50pg/mL vs. 500µM	-3.167	No	ns	>0.9999
TNFa vs. Butyrate	50pg/mL vs. 750µM	-79.5	No	ns	>0.9999
TNFa vs. Butyrate	50pg/mL vs. 1000µM	-73.83	No	ns	>0.9999
TNFa vs. Butyrate	50pg/mL vs. 2500µM	-61.25	No	ns	>0.9999
TNFa vs. Butyrate	50pg/mL vs. 5000µM	-57.33	No	ns	>0.9999
TNFa vs. Butyrate	50pg/mL vs. 7500µM	-29.5	No	ns	>0.9999
TNFa vs. Combo	50pg/mL vs. 50pg/mL	-17	No	ns	>0.9999
TNFa vs. Combo	50pg/mL vs. 100pg/mL	36.92	No	ns	>0.9999
TNFa vs. Combo	50pg/mL vs. 150pg/mL	41.17	No	ns	>0.9999
TNFa vs. Combo	50pg/mL vs. 200pg/mL	-82.67	No	ns	>0.9999
TNFa vs. Combo	50pg/mL vs. 250pg/mL	-80.83	No	ns	>0.9999
TNFa vs. Combo	50pg/mL vs. 500pg/mL	-79.17	No	ns	>0.9999
TNFa vs. Combo	50pg/mL vs. 1000pg/mL	-57.92	No	ns	>0.9999
TNFa vs. Combo	50pg/mL vs. 2500pg/mL	-52	No	ns	>0.9999
TNFa vs. Combo	50pg/mL vs. 5000pg/mL	-44.83	No	ns	>0.9999
TNFa vs. TNFa	100pg/mL vs. 150pg/mL	6.917	No	ns	>0.9999
TNFa vs. TNFa	100pg/mL vs. 200pg/mL	-8.833	No	ns	>0.9999
TNFa vs. TNFa	100pg/mL vs. 250pg/mL	19.17	No	ns	>0.9999
TNFa vs. TNFa	100pg/mL vs. 500pg/mL	5.667	No	ns	>0.9999
TNFa vs. TNFa	100pg/mL vs. 1000pg/mL	-102	No	ns	0.3034
TNFa vs. TNFa	100pg/mL vs. 2500pg/mL	-64	No	ns	>0.9999
TNFa vs. TNFa	100pg/mL vs. 5000pg/mL	-28.67	No	ns	>0.9999
TNFa vs. Butyrate	100pg/mL vs. 50µM	-50.83	No	ns	>0.9999
TNFa vs. Butyrate	100pg/mL vs. 75µM	-26.5	No	ns	>0.9999

TNFa vs. Butyrate	100pg/mL vs. 100µM	-18.17	No	ns	>0.9999
TNFa vs. Butyrate	100pg/mL vs. 250µM	17.33	No	ns	>0.9999
TNFa vs. Butyrate	100pg/mL vs. 500µM	-37.5	No	ns	>0.9999
TNFa vs. Butyrate	100pg/mL vs. 750µM	-113.8	No	ns	0.0671
TNFa vs. Butyrate	100pg/mL vs. 1000µM	-108.2	No	ns	0.1408
TNFa vs. Butyrate	100pg/mL vs. 2500µM	-95.58	No	ns	0.6466
TNFa vs. Butyrate	100pg/mL vs. 5000µM	-91.67	No	ns	>0.9999
TNFa vs. Butyrate	100pg/mL vs. 7500µM	-63.83	No	ns	>0.9999
TNFa vs. Combo	100pg/mL vs. 50pg/mL	-51.33	No	ns	>0.9999
TNFa vs. Combo	100pg/mL vs. 100pg/mL	2.583	No	ns	>0.9999
TNFa vs. Combo	100pg/mL vs. 150pg/mL	6.833	No	ns	>0.9999
TNFa vs. Combo	100pg/mL vs. 200pg/mL	-117	Yes	*	0.0437
TNFa vs. Combo	100pg/mL vs. 250pg/mL	-115.2	No	ns	0.0561
TNFa vs. Combo	100pg/mL vs. 500pg/mL	-113.5	No	ns	0.0702
TNFa vs. Combo	100pg/mL vs. 1000pg/mL	-92.25	No	ns	0.9421
TNFa vs. Combo	100pg/mL vs. 2500pg/mL	-86.33	No	ns	>0.9999
TNFa vs. Combo	100pg/mL vs. 5000pg/mL	-79.17	No	ns	>0.9999
TNFa vs. TNFa	150pg/mL vs. 200pg/mL	-15.75	No	ns	>0.9999
TNFa vs. TNFa	150pg/mL vs. 250pg/mL	12.25	No	ns	>0.9999
TNFa vs. TNFa	150pg/mL vs. 500pg/mL	-1.25	No	ns	>0.9999
TNFa vs. TNFa	150pg/mL vs. 1000pg/mL	-108.9	No	ns	0.1279
TNFa vs. TNFa	150pg/mL vs. 2500pg/mL	-70.92	No	ns	>0.9999
TNFa vs. TNFa	150pg/mL vs. 5000pg/mL	-35.58	No	ns	>0.9999
TNFa vs. Butyrate	150pg/mL vs. 50µM	-57.75	No	ns	>0.9999
TNFa vs. Butyrate	150pg/mL vs. 75µM	-33.42	No	ns	>0.9999
TNFa vs. Butyrate	150pg/mL vs. 100µM	-25.08	No	ns	>0.9999
TNFa vs. Butyrate	150pg/mL vs. 250µM	10.42	No	ns	>0.9999
TNFa vs. Butyrate	150pg/mL vs. 500µM	-44.42	No	ns	>0.9999
TNFa vs. Butyrate	150pg/mL vs. 750µM	-120.8	Yes	*	0.026
TNFa vs. Butyrate	150pg/mL vs. 1000µM	-115.1	No	ns	0.0568
TNFa vs. Butyrate	150pg/mL vs. 2500µM	-102.5	No	ns	0.2855
TNFa vs. Butyrate	150pg/mL vs. 5000µM	-98.58	No	ns	0.4563
TNFa vs. Butyrate	150pg/mL vs. 7500µM	-70.75	No	ns	>0.9999
TNFa vs. Combo	150pg/mL vs. 50pg/mL	-58.25	No	ns	>0.9999
TNFa vs. Combo	150pg/mL vs. 100pg/mL	-4.333	No	ns	>0.9999
TNFa vs. Combo	150pg/mL vs. 150pg/mL	-0.08333	No	ns	>0.9999
TNFa vs. Combo	150pg/mL vs. 200pg/mL	-123.9	Yes	*	0.0165
TNFa vs. Combo	150pg/mL vs. 250pg/mL	-122.1	Yes	*	0.0215
TNFa vs. Combo	150pg/mL vs. 500pg/mL	-120.4	Yes	*	0.0272
TNFa vs. Combo	150pg/mL vs. 1000pg/mL	-99.17	No	ns	0.426
TNFa vs. Combo	150pg/mL vs. 2500pg/mL	-93.25	No	ns	0.8426
TNFa vs. Combo	150pg/mL vs. 5000pg/mL	-86.08	No	ns	>0.9999
TNFa vs. TNFa	200pg/mL vs. 250pg/mL	28	No	ns	>0.9999
TNFa vs. TNFa	200pg/mL vs. 500pg/mL	14.5	No	ns	>0.9999
TNFa vs. TNFa	200pg/mL vs. 1000pg/mL	-93.17	No	ns	0.8505
TNFa vs. TNFa	200pg/mL vs. 2500pg/mL	-55.17	No	ns	>0.9999
TNFa vs. TNFa	200pg/mL vs. 5000pg/mL	-19.83	No	ns	>0.9999

TNFa vs. Butyrate	200pg/mL vs. 50µM	-42	No	ns	>0.9999
TNFa vs. Butyrate	200pg/mL vs. 75µM	-17.67	No	ns	>0.9999
TNFa vs. Butyrate	200pg/mL vs. 100µM	-9.333	No	ns	>0.9999
TNFa vs. Butyrate	200pg/mL vs. 250µM	26.17	No	ns	>0.9999
TNFa vs. Butyrate	200pg/mL vs. 500µM	-28.67	No	ns	>0.9999
TNFa vs. Butyrate	200pg/mL vs. 750µM	-105	No	ns	0.2099
TNFa vs. Butyrate	200pg/mL vs. 1000µM	-99.33	No	ns	0.4177
TNFa vs. Butyrate	200pg/mL vs. 2500µM	-86.75	No	ns	>0.9999
TNFa vs. Butyrate	200pg/mL vs. 5000µM	-82.83	No	ns	>0.9999
TNFa vs. Butyrate	200pg/mL vs. 7500µM	-55	No	ns	>0.9999
TNFa vs. Combo	200pg/mL vs. 50pg/mL	-42.5	No	ns	>0.9999
TNFa vs. Combo	200pg/mL vs. 100pg/mL	11.42	No	ns	>0.9999
TNFa vs. Combo	200pg/mL vs. 150pg/mL	15.67	No	ns	>0.9999
TNFa vs. Combo	200pg/mL vs. 200pg/mL	-108.2	No	ns	0.1408
TNFa vs. Combo	200pg/mL vs. 250pg/mL	-106.3	No	ns	0.1776
TNFa vs. Combo	200pg/mL vs. 500pg/mL	-104.7	No	ns	0.2187
TNFa vs. Combo	200pg/mL vs. 1000pg/mL	-83.42	No	ns	>0.9999
TNFa vs. Combo	200pg/mL vs. 2500pg/mL	-77.5	No	ns	>0.9999
TNFa vs. Combo	200pg/mL vs. 5000pg/mL	-70.33	No	ns	>0.9999
TNFa vs. TNFa	250pg/mL vs. 500pg/mL	-13.5	No	ns	>0.9999
TNFa vs. TNFa	250pg/mL vs. 1000pg/mL	-121.2	Yes	*	0.0245
TNFa vs. TNFa	250pg/mL vs. 2500pg/mL	-83.17	No	ns	>0.9999
TNFa vs. TNFa	250pg/mL vs. 5000pg/mL	-47.83	No	ns	>0.9999
TNFa vs. Butyrate	250pg/mL vs. 50µM	-70	No	ns	>0.9999
TNFa vs. Butyrate	250pg/mL vs. 75µM	-45.67	No	ns	>0.9999
TNFa vs. Butyrate	250pg/mL vs. 100µM	-37.33	No	ns	>0.9999
TNFa vs. Butyrate	250pg/mL vs. 250µM	-1.833	No	ns	>0.9999
TNFa vs. Butyrate	250pg/mL vs. 500µM	-56.67	No	ns	>0.9999
TNFa vs. Butyrate	250pg/mL vs. 750µM	-133	Yes	**	0.0043
TNFa vs. Butyrate	250pg/mL vs. 1000µM	-127.3	Yes	*	0.01
TNFa vs. Butyrate	250pg/mL vs. 2500µM	-114.8	No	ns	0.0594
TNFa vs. Butyrate	250pg/mL vs. 5000µM	-110.8	No	ns	0.0998
TNFa vs. Butyrate	250pg/mL vs. 7500µM	-83	No	ns	>0.9999
TNFa vs. Combo	250pg/mL vs. 50pg/mL	-70.5	No	ns	>0.9999
TNFa vs. Combo	250pg/mL vs. 100pg/mL	-16.58	No	ns	>0.9999
TNFa vs. Combo	250pg/mL vs. 150pg/mL	-12.33	No	ns	>0.9999
TNFa vs. Combo	250pg/mL vs. 200pg/mL	-136.2	Yes	**	0.0026
TNFa vs. Combo	250pg/mL vs. 250pg/mL	-134.3	Yes	**	0.0035
TNFa vs. Combo	250pg/mL vs. 500pg/mL	-132.7	Yes	**	0.0045
TNFa vs. Combo	250pg/mL vs. 1000pg/mL	-111.4	No	ns	0.0925
TNFa vs. Combo	250pg/mL vs. 2500pg/mL	-105.5	No	ns	0.1972
TNFa vs. Combo	250pg/mL vs. 5000pg/mL	-98.33	No	ns	0.47
TNFa vs. TNFa	500pg/mL vs. 1000pg/mL	-107.7	No	ns	0.1501
TNFa vs. TNFa	500pg/mL vs. 2500pg/mL	-69.67	No	ns	>0.9999
TNFa vs. TNFa	500pg/mL vs. 5000pg/mL	-34.33	No	ns	>0.9999
TNFa vs. Butyrate	500pg/mL vs. 50µM	-56.5	No	ns	>0.9999
TNFa vs. Butyrate	500pg/mL vs. 75µM	-32.17	No	ns	>0.9999

TNFa vs. Butyrate	500pg/mL vs. 100µM	-23.83	No	ns	>0.9999
TNFa vs. Butyrate	500pg/mL vs. 250µM	11.67	No	ns	>0.9999
TNFa vs. Butyrate	500pg/mL vs. 500µM	-43.17	No	ns	>0.9999
TNFa vs. Butyrate	500pg/mL vs. 750µM	-119.5	Yes	*	0.031
TNFa vs. Butyrate	500pg/mL vs. 1000µM	-113.8	No	ns	0.0671
TNFa vs. Butyrate	500pg/mL vs. 2500µM	-101.3	No	ns	0.3322
TNFa vs. Butyrate	500pg/mL vs. 5000µM	-97.33	No	ns	0.5283
TNFa vs. Butyrate	500pg/mL vs. 7500µM	-69.5	No	ns	>0.9999
TNFa vs. Combo	500pg/mL vs. 50pg/mL	-57	No	ns	>0.9999
TNFa vs. Combo	500pg/mL vs. 100pg/mL	-3.083	No	ns	>0.9999
TNFa vs. Combo	500pg/mL vs. 150pg/mL	1.167	No	ns	>0.9999
TNFa vs. Combo	500pg/mL vs. 200pg/mL	-122.7	Yes	*	0.0198
TNFa vs. Combo	500pg/mL vs. 250pg/mL	-120.8	Yes	*	0.0257
TNFa vs. Combo	500pg/mL vs. 500pg/mL	-119.2	Yes	*	0.0324
TNFa vs. Combo	500pg/mL vs. 1000pg/mL	-97.92	No	ns	0.4935
TNFa vs. Combo	500pg/mL vs. 2500pg/mL	-92	No	ns	0.9687
TNFa vs. Combo	500pg/mL vs. 5000pg/mL	-84.83	No	ns	>0.9999
TNFa vs. TNFa	1000pg/mL vs. 2500pg/mL	38	No	ns	>0.9999
TNFa vs. TNFa	1000pg/mL vs. 5000pg/mL	73.33	No	ns	>0.9999
TNFa vs. Butyrate	1000pg/mL vs. 50µM	51.17	No	ns	>0.9999
TNFa vs. Butyrate	1000pg/mL vs. 75µM	75.5	No	ns	>0.9999
TNFa vs. Butyrate	1000pg/mL vs. 100µM	83.83	No	ns	>0.9999
TNFa vs. Butyrate	1000pg/mL vs. 250µM	119.3	Yes	*	0.0317
TNFa vs. Butyrate	1000pg/mL vs. 500µM	64.5	No	ns	>0.9999
TNFa vs. Butyrate	1000pg/mL vs. 750µM	-11.83	No	ns	>0.9999
TNFa vs. Butyrate	1000pg/mL vs. 1000µM	-6.167	No	ns	>0.9999
TNFa vs. Butyrate	1000pg/mL vs. 2500µM	6.417	No	ns	>0.9999
TNFa vs. Butyrate	1000pg/mL vs. 5000µM	10.33	No	ns	>0.9999
TNFa vs. Butyrate	1000pg/mL vs. 7500µM	38.17	No	ns	>0.9999
TNFa vs. Combo	1000pg/mL vs. 50pg/mL	50.67	No	ns	>0.9999
TNFa vs. Combo	1000pg/mL vs. 100pg/mL	104.6	No	ns	0.221
TNFa vs. Combo	1000pg/mL vs. 150pg/mL	108.8	No	ns	0.1293
TNFa vs. Combo	1000pg/mL vs. 200pg/mL	-15	No	ns	>0.9999
TNFa vs. Combo	1000pg/mL vs. 250pg/mL	-13.17	No	ns	>0.9999
TNFa vs. Combo	1000pg/mL vs. 500pg/mL	-11.5	No	ns	>0.9999
TNFa vs. Combo	1000pg/mL vs. 1000pg/mL	9.75	No	ns	>0.9999
TNFa vs. Combo	1000pg/mL vs. 2500pg/mL	15.67	No	ns	>0.9999
TNFa vs. Combo	1000pg/mL vs. 5000pg/mL	22.83	No	ns	>0.9999
TNFa vs. TNFa	2500pg/mL vs. 5000pg/mL	35.33	No	ns	>0.9999
TNFa vs. Butyrate	2500pg/mL vs. 50µM	13.17	No	ns	>0.9999
TNFa vs. Butyrate	2500pg/mL vs. 75µM	37.5	No	ns	>0.9999
TNFa vs. Butyrate	2500pg/mL vs. 100µM	45.83	No	ns	>0.9999
TNFa vs. Butyrate	2500pg/mL vs. 250µM	81.33	No	ns	>0.9999
TNFa vs. Butyrate	2500pg/mL vs. 500µM	26.5	No	ns	>0.9999
TNFa vs. Butyrate	2500pg/mL vs. 750µM	-49.83	No	ns	>0.9999
TNFa vs. Butyrate	2500pg/mL vs. 1000µM	-44.17	No	ns	>0.9999
TNFa vs. Butyrate	2500pg/mL vs. 2500µM	-31.58	No	ns	>0.9999

TNFa vs. Butyrate	2500pg/mL vs. 5000µM	-27.67	No	ns	>0.9999
TNFa vs. Butyrate	2500pg/mL vs. 7500µM	0.1667	No	ns	>0.9999
TNFa vs. Combo	2500pg/mL vs. 50pg/mL	12.67	No	ns	>0.9999
TNFa vs. Combo	2500pg/mL vs. 100pg/mL	66.58	No	ns	>0.9999
TNFa vs. Combo	2500pg/mL vs. 150pg/mL	70.83	No	ns	>0.9999
TNFa vs. Combo	2500pg/mL vs. 200pg/mL	-53	No	ns	>0.9999
TNFa vs. Combo	2500pg/mL vs. 250pg/mL	-51.17	No	ns	>0.9999
TNFa vs. Combo	2500pg/mL vs. 500pg/mL	-49.5	No	ns	>0.9999
TNFa vs. Combo	2500pg/mL vs. 1000pg/mL	-28.25	No	ns	>0.9999
TNFa vs. Combo	2500pg/mL vs. 2500pg/mL	-22.33	No	ns	>0.9999
TNFa vs. Combo	2500pg/mL vs. 5000pg/mL	-15.17	No	ns	>0.9999
TNFa vs. Butyrate	5000pg/mL vs. 50µM	-22.17	No	ns	>0.9999
TNFa vs. Butyrate	5000pg/mL vs. 75µM	2.167	No	ns	>0.9999
TNFa vs. Butyrate	5000pg/mL vs. 100µM	10.5	No	ns	>0.9999
TNFa vs. Butyrate	5000pg/mL vs. 250µM	46	No	ns	>0.9999
TNFa vs. Butyrate	5000pg/mL vs. 500µM	-8.833	No	ns	>0.9999
TNFa vs. Butyrate	5000pg/mL vs. 750µM	-85.17	No	ns	>0.9999
TNFa vs. Butyrate	5000pg/mL vs. 1000µM	-79.5	No	ns	>0.9999
TNFa vs. Butyrate	5000pg/mL vs. 2500µM	-66.92	No	ns	>0.9999
TNFa vs. Butyrate	5000pg/mL vs. 5000µM	-63	No	ns	>0.9999
TNFa vs. Butyrate	5000pg/mL vs. 7500µM	-35.17	No	ns	>0.9999
TNFa vs. Combo	5000pg/mL vs. 50pg/mL	-22.67	No	ns	>0.9999
TNFa vs. Combo	5000pg/mL vs. 100pg/mL	31.25	No	ns	>0.9999
TNFa vs. Combo	5000pg/mL vs. 150pg/mL	35.5	No	ns	>0.9999
TNFa vs. Combo	5000pg/mL vs. 200pg/mL	-88.33	No	ns	>0.9999
TNFa vs. Combo	5000pg/mL vs. 250pg/mL	-86.5	No	ns	>0.9999
TNFa vs. Combo	5000pg/mL vs. 500pg/mL	-84.83	No	ns	>0.9999
TNFa vs. Combo	5000pg/mL vs. 1000pg/mL	-63.58	No	ns	>0.9999
TNFa vs. Combo	5000pg/mL vs. 2500pg/mL	-57.67	No	ns	>0.9999
TNFa vs. Combo	5000pg/mL vs. 5000pg/mL	-50.5	No	ns	>0.9999
Butyrate vs. Butyrate	50µM vs. 75µM	24.33	No	ns	>0.9999
Butyrate vs. Butyrate	50µM vs. 100µM	32.67	No	ns	>0.9999
Butyrate vs. Butyrate	50µM vs. 250µM	68.17	No	ns	>0.9999
Butyrate vs. Butyrate	50µM vs. 500µM	13.33	No	ns	>0.9999
Butyrate vs. Butyrate	50µM vs. 750µM	-63	No	ns	>0.9999
Butyrate vs. Butyrate	50µM vs. 1000µM	-57.33	No	ns	>0.9999
Butyrate vs. Butyrate	50µM vs. 2500µM	-44.75	No	ns	>0.9999
Butyrate vs. Butyrate	50µM vs. 5000µM	-40.83	No	ns	>0.9999
Butyrate vs. Butyrate	50µM vs. 7500µM	-13	No	ns	>0.9999
Butyrate + Combo	50µM vs. 50pg/mL	-0.5	No	ns	>0.9999
Butyrate + Combo	50µM vs. 100pg/mL	53.42	No	ns	>0.9999
Butyrate + Combo	50µM vs. 150pg/mL	57.67	No	ns	>0.9999
Butyrate + Combo	50µM vs. 200pg/mL	-66.17	No	ns	>0.9999
Butyrate + Combo	50µM vs. 250pg/mL	-64.33	No	ns	>0.9999
Butyrate + Combo	50µM vs. 500pg/mL	-62.67	No	ns	>0.9999
Butyrate + Combo	50µM vs. 1000pg/mL	-41.42	No	ns	>0.9999
Butyrate + Combo	50µM vs. 2500pg/mL	-35.5	No	ns	>0.9999

Butyrate + Combo	50μM vs. 5000pg/mL	-28.33	No	ns	>0.9999
Butyrate vs. Butyrate	75μM vs. 100μM	8.333	No	ns	>0.9999
Butyrate vs. Butyrate	75μM vs. 250μM	43.83	No	ns	>0.9999
Butyrate vs. Butyrate	75μM vs. 500μM	-11	No	ns	>0.9999
Butyrate vs. Butyrate	75μM vs. 750μM	-87.33	No	ns	>0.9999
Butyrate vs. Butyrate	75μM vs. 1000μM	-81.67	No	ns	>0.9999
Butyrate vs. Butyrate	75μM vs. 2500μM	-69.08	No	ns	>0.9999
Butyrate vs. Butyrate	75μM vs. 5000μM	-65.17	No	ns	>0.9999
Butyrate vs. Butyrate	75μM vs. 7500μM	-37.33	No	ns	>0.9999
Butyrate + Combo	75μM vs. 50pg/mL	-24.83	No	ns	>0.9999
Butyrate + Combo	75μM vs. 100pg/mL	29.08	No	ns	>0.9999
Butyrate + Combo	75μM vs. 150pg/mL	33.33	No	ns	>0.9999
Butyrate + Combo	75μM vs. 200pg/mL	-90.5	No	ns	>0.9999
Butyrate + Combo	75μM vs. 250pg/mL	-88.67	No	ns	>0.9999
Butyrate + Combo	75μM vs. 500pg/mL	-87	No	ns	>0.9999
Butyrate + Combo	75μM vs. 1000pg/mL	-65.75	No	ns	>0.9999
Butyrate + Combo	75μM vs. 2500pg/mL	-59.83	No	ns	>0.9999
Butyrate + Combo	75μM vs. 5000pg/mL	-52.67	No	ns	>0.9999
Butyrate vs. Butyrate	100μM vs. 250μM	35.5	No	ns	>0.9999
Butyrate vs. Butyrate	100μM vs. 500μM	-19.33	No	ns	>0.9999
Butyrate vs. Butyrate	100μM vs. 750μM	-95.67	No	ns	0.6405
Butyrate vs. Butyrate	100μM vs. 1000μM	-90	No	ns	>0.9999
Butyrate vs. Butyrate	100μM vs. 2500μM	-77.42	No	ns	>0.9999
Butyrate vs. Butyrate	100μM vs. 5000μM	-73.5	No	ns	>0.9999
Butyrate vs. Butyrate	100μM vs. 7500μM	-45.67	No	ns	>0.9999
Butyrate + Combo	100μM vs. 50pg/mL	-33.17	No	ns	>0.9999
Butyrate + Combo	100μM vs. 100pg/mL	20.75	No	ns	>0.9999
Butyrate + Combo	100μM vs. 150pg/mL	25	No	ns	>0.9999
Butyrate + Combo	100μM vs. 200pg/mL	-98.83	No	ns	0.4431
Butyrate + Combo	100μM vs. 250pg/mL	-97	No	ns	0.5491
Butyrate + Combo	100μM vs. 500pg/mL	-95.33	No	ns	0.6654
Butyrate + Combo	100μM vs. 1000pg/mL	-74.08	No	ns	>0.9999
Butyrate + Combo	100μM vs. 2500pg/mL	-68.17	No	ns	>0.9999
Butyrate + Combo	100μM vs. 5000pg/mL	-61	No	ns	>0.9999
Butyrate vs. Butyrate	250μM vs. 500μM	-54.83	No	ns	>0.9999
Butyrate vs. Butyrate	250μM vs. 750μM	-131.2	Yes	**	0.0057
Butyrate vs. Butyrate	250μM vs. 1000μM	-125.5	Yes	*	0.0131
Butyrate vs. Butyrate	250μM vs. 2500μM	-112.9	No	ns	0.0759
Butyrate vs. Butyrate	250μM vs. 5000μM	-109	No	ns	0.1265
Butyrate vs. Butyrate	250μM vs. 7500μM	-81.17	No	ns	>0.9999
Butyrate + Combo	250μM vs. 50pg/mL	-68.67	No	ns	>0.9999
Butyrate + Combo	250μM vs. 100pg/mL	-14.75	No	ns	>0.9999
Butyrate + Combo	250μM vs. 150pg/mL	-10.5	No	ns	>0.9999
Butyrate + Combo	250μM vs. 200pg/mL	-134.3	Yes	**	0.0035
Butyrate + Combo	250μM vs. 250pg/mL	-132.5	Yes	**	0.0046
Butyrate + Combo	250μM vs. 500pg/mL	-130.8	Yes	**	0.0059
Butyrate + Combo	250μM vs. 1000pg/mL	-109.6	No	ns	0.1174

Butyrate + Combo	250µM vs. 2500pg/mL	-103.7	No	ns	0.2475
Butyrate + Combo	250µM vs. 5000pg/mL	-96.5	No	ns	0.5819
Butyrate vs. Butyrate	500µM vs. 750µM	-76.33	No	ns	>0.9999
Butyrate vs. Butyrate	500µM vs. 1000µM	-70.67	No	ns	>0.9999
Butyrate vs. Butyrate	500µM vs. 2500µM	-58.08	No	ns	>0.9999
Butyrate vs. Butyrate	500µM vs. 5000µM	-54.17	No	ns	>0.9999
Butyrate vs. Butyrate	500µM vs. 7500µM	-26.33	No	ns	>0.9999
Butyrate + Combo	500µM vs. 50pg/mL	-13.83	No	ns	>0.9999
Butyrate + Combo	500µM vs. 100pg/mL	40.08	No	ns	>0.9999
Butyrate + Combo	500µM vs. 150pg/mL	44.33	No	ns	>0.9999
Butyrate + Combo	500µM vs. 200pg/mL	-79.5	No	ns	>0.9999
Butyrate + Combo	500µM vs. 250pg/mL	-77.67	No	ns	>0.9999
Butyrate + Combo	500µM vs. 500pg/mL	-76	No	ns	>0.9999
Butyrate + Combo	500µM vs. 1000pg/mL	-54.75	No	ns	>0.9999
Butyrate + Combo	500µM vs. 2500pg/mL	-48.83	No	ns	>0.9999
Butyrate + Combo	500µM vs. 5000pg/mL	-41.67	No	ns	>0.9999
Butyrate vs. Butyrate	750µM vs. 1000µM	5.667	No	ns	>0.9999
Butyrate vs. Butyrate	750µM vs. 2500µM	18.25	No	ns	>0.9999
Butyrate vs. Butyrate	750µM vs. 5000µM	22.17	No	ns	>0.9999
Butyrate vs. Butyrate	750µM vs. 7500µM	50	No	ns	>0.9999
Butyrate + Combo	750µM vs. 50pg/mL	62.5	No	ns	>0.9999
Butyrate + Combo	750µM vs. 100pg/mL	116.4	Yes	*	0.0474
Butyrate + Combo	750µM vs. 150pg/mL	120.7	Yes	*	0.0263
Butyrate + Combo	750µM vs. 200pg/mL	-3.167	No	ns	>0.9999
Butyrate + Combo	750µM vs. 250pg/mL	-1.333	No	ns	>0.9999
Butyrate + Combo	750µM vs. 500pg/mL	0.3333	No	ns	>0.9999
Butyrate + Combo	750µM vs. 1000pg/mL	21.58	No	ns	>0.9999
Butyrate + Combo	750µM vs. 2500pg/mL	27.5	No	ns	>0.9999
Butyrate + Combo	750µM vs. 5000pg/mL	34.67	No	ns	>0.9999
Butyrate vs. Butyrate	1000µM vs. 2500µM	12.58	No	ns	>0.9999
Butyrate vs. Butyrate	1000µM vs. 5000µM	16.5	No	ns	>0.9999
Butyrate vs. Butyrate	1000µM vs. 7500µM	44.33	No	ns	>0.9999
Butyrate + Combo	1000µM vs. 50pg/mL	56.83	No	ns	>0.9999
Butyrate + Combo	1000µM vs. 100pg/mL	110.8	No	ns	0.1009
Butyrate + Combo	1000µM vs. 150pg/mL	115	No	ns	0.0574
Butyrate + Combo	1000µM vs. 200pg/mL	-8.833	No	ns	>0.9999
Butyrate + Combo	1000µM vs. 250pg/mL	-7	No	ns	>0.9999
Butyrate + Combo	1000µM vs. 500pg/mL	-5.333	No	ns	>0.9999
Butyrate + Combo	1000µM vs. 1000pg/mL	15.92	No	ns	>0.9999
Butyrate + Combo	1000µM vs. 2500pg/mL	21.83	No	ns	>0.9999
Butyrate + Combo	1000µM vs. 5000pg/mL	29	No	ns	>0.9999
Butyrate vs. Butyrate	2500µM vs. 5000µM	3.917	No	ns	>0.9999
Butyrate vs. Butyrate	2500µM vs. 7500µM	31.75	No	ns	>0.9999
Butyrate + Combo	2500µM vs. 50pg/mL	44.25	No	ns	>0.9999
Butyrate + Combo	2500µM vs. 100pg/mL	98.17	No	ns	0.4792
Butyrate + Combo	2500µM vs. 150pg/mL	102.4	No	ns	0.2884
Butyrate + Combo	2500µM vs. 200pg/mL	-21.42	No	ns	>0.9999

Butyrate + Combo	2500µM vs. 250pg/mL	-19.58	No	ns	>0.9999
Butyrate + Combo	2500µM vs. 500pg/mL	-17.92	No	ns	>0.9999
Butyrate + Combo	2500µM vs. 1000pg/mL	3.333	No	ns	>0.9999
Butyrate + Combo	2500µM vs. 2500pg/mL	9.25	No	ns	>0.9999
Butyrate + Combo	2500µM vs. 5000pg/mL	16.42	No	ns	>0.9999
Butyrate vs. Butyrate	5000µM vs. 7500µM	27.83	No	ns	>0.9999
Butyrate + Combo	5000µM vs. 50pg/mL	40.33	No	ns	>0.9999
Butyrate + Combo	5000µM vs. 100pg/mL	94.25	No	ns	0.7527
Butyrate + Combo	5000µM vs. 150pg/mL	98.5	No	ns	0.4608
Butyrate + Combo	5000µM vs. 200pg/mL	-25.33	No	ns	>0.9999
Butyrate + Combo	5000µM vs. 250pg/mL	-23.5	No	ns	>0.9999
Butyrate + Combo	5000µM vs. 500pg/mL	-21.83	No	ns	>0.9999
Butyrate + Combo	5000µM vs. 1000pg/mL	-0.5833	No	ns	>0.9999
Butyrate + Combo	5000µM vs. 2500pg/mL	5.333	No	ns	>0.9999
Butyrate + Combo	5000µM vs. 5000pg/mL	12.5	No	ns	>0.9999
Butyrate + Combo	7500µM vs. 50pg/mL	12.5	No	ns	>0.9999
Butyrate + Combo	7500µM vs. 100pg/mL	66.42	No	ns	>0.9999
Butyrate + Combo	7500µM vs. 150pg/mL	70.67	No	ns	>0.9999
Butyrate + Combo	7500µM vs. 200pg/mL	-53.17	No	ns	>0.9999
Butyrate + Combo	7500µM vs. 250pg/mL	-51.33	No	ns	>0.9999
Butyrate + Combo	7500µM vs. 500pg/mL	-49.67	No	ns	>0.9999
Butyrate + Combo	7500µM vs. 1000pg/mL	-28.42	No	ns	>0.9999
Butyrate + Combo	7500µM vs. 2500pg/mL	-22.5	No	ns	>0.9999
Butyrate + Combo	7500µM vs. 5000pg/mL	-15.33	No	ns	>0.9999
Combo vs. Combo	50pg/mL vs. 100pg/mL	53.92	No	ns	>0.9999
Combo vs. Combo	50pg/mL vs. 150pg/mL	58.17	No	ns	>0.9999
Combo vs. Combo	50pg/mL vs. 200pg/mL	-65.67	No	ns	>0.9999
Combo vs. Combo	50pg/mL vs. 250pg/mL	-63.83	No	ns	>0.9999
Combo vs. Combo	50pg/mL vs. 500pg/mL	-62.17	No	ns	>0.9999
Combo vs. Combo	50pg/mL vs. 1000pg/mL	-40.92	No	ns	>0.9999
Combo vs. Combo	50pg/mL vs. 2500pg/mL	-35	No	ns	>0.9999
Combo vs. Combo	50pg/mL vs. 5000pg/mL	-27.83	No	ns	>0.9999
Combo vs. Combo	100pg/mL vs. 150pg/mL	4.25	No	ns	>0.9999
Combo vs. Combo	100pg/mL vs. 200pg/mL	-119.6	Yes	*	0.0306
Combo vs. Combo	100pg/mL vs. 250pg/mL	-117.8	Yes	*	0.0395
Combo vs. Combo	100pg/mL vs. 500pg/mL	-116.1	Yes	*	0.0496
Combo vs. Combo	100pg/mL vs. 1000pg/mL	-94.83	No	ns	0.7045
Combo vs. Combo	100pg/mL vs. 2500pg/mL	-88.92	No	ns	>0.9999
Combo vs. Combo	100pg/mL vs. 5000pg/mL	-81.75	No	ns	>0.9999
Combo vs. Combo	150pg/mL vs. 200pg/mL	-123.8	Yes	*	0.0167
Combo vs. Combo	150pg/mL vs. 250pg/mL	-122	Yes	*	0.0218
Combo vs. Combo	150pg/mL vs. 500pg/mL	-120.3	Yes	*	0.0276
Combo vs. Combo	150pg/mL vs. 1000pg/mL	-99.08	No	ns	0.4302
Combo vs. Combo	150pg/mL vs. 2500pg/mL	-93.17	No	ns	0.8505
Combo vs. Combo	150pg/mL vs. 5000pg/mL	-86	No	ns	>0.9999
Combo vs. Combo	200pg/mL vs. 250pg/mL	1.833	No	ns	>0.9999
Combo vs. Combo	200pg/mL vs. 500pg/mL	3.5	No	ns	>0.9999

Combo vs. Combo	200pg/mL vs. 1000pg/mL	24.75	No	ns	>0.9999
Combo vs. Combo	200pg/mL vs. 2500pg/mL	30.67	No	ns	>0.9999
Combo vs. Combo	200pg/mL vs. 5000pg/mL	37.83	No	ns	>0.9999
Combo vs. Combo	250pg/mL vs. 500pg/mL	1.667	No	ns	>0.9999
Combo vs. Combo	250pg/mL vs. 1000pg/mL	22.92	No	ns	>0.9999
Combo vs. Combo	250pg/mL vs. 2500pg/mL	28.83	No	ns	>0.9999
Combo vs. Combo	250pg/mL vs. 5000pg/mL	36	No	ns	>0.9999
Combo vs. Combo	500pg/mL vs. 1000pg/mL	21.25	No	ns	>0.9999
Combo vs. Combo	500pg/mL vs. 2500pg/mL	27.17	No	ns	>0.9999
Combo vs. Combo	500pg/mL vs. 5000pg/mL	34.33	No	ns	>0.9999
Combo vs. Combo	1000pg/mL vs. 2500pg/mL	5.917	No	ns	>0.9999
Combo vs. Combo	1000pg/mL vs. 5000pg/mL	13.08	No	ns	>0.9999
Combo vs. Combo	2500pg/mL vs. 5000pg/mL	7.167	No	ns	

Value
A-B
A-C
A-D
A-E
A-F
A-G
A-H
A-I
A-J
A-K
A-L
A-M
A-N
A-O
A-P
A-Q
A-R
A-S
A-T
A-U
A-V
A-W
A-X
A-Y
A-Z
A-AA
A-AB
A-AC
A-AD
B-C
B-D
B-E
B-F
B-G
B-H
B-I
B-J
B-K
B-L
B-M
B-N
B-O
B-P
B-Q
B-R
B-S

B-T
B-U
B-V
B-W
B-X
B-Y
B-Z
B-AA
B-AB
B-AC
B-AD
C-D
C-E
C-F
C-G
C-H
C-I
C-J
C-K
C-L
C-M
C-N
C-O
C-P
C-Q
C-R
C-S
C-T
C-U
C-V
C-W
C-X
C-Y
C-Z
C-AA
C-AB
C-AC
C-AD
D-E
D-F
D-G
D-H
D-I
D-J
D-K
D-L
D-M

D-N
D-O
D-P
D-Q
D-R
D-S
D-T
D-U
D-V
D-W
D-X
D-Y
D-Z
D-AA
D-AB
D-AC
D-AD
E-F
E-G
E-H
E-I
E-J
E-K
E-L
E-M
E-N
E-O
E-P
E-Q
E-R
E-S
E-T
E-U
E-V
E-W
E-X
E-Y
E-Z
E-AA
E-AB
E-AC
E-AD
F-G
F-H
F-I
F-J
F-K

F-L
F-M
F-N
F-O
F-P
F-Q
F-R
F-S
F-T
F-U
F-V
F-W
F-X
F-Y
F-Z
F-AA
F-AB
F-AC
F-AD
G-H
G-I
G-J
G-K
G-L
G-M
G-N
G-O
G-P
G-Q
G-R
G-S
G-T
G-U
G-V
G-W
G-X
G-Y
G-Z
G-AA
G-AB
G-AC
G-AD
H-I
H-J
H-K
H-L
H-M

H-N
H-O
H-P
H-Q
H-R
H-S
H-T
H-U
H-V
H-W
H-X
H-Y
H-Z
H-AA
H-AB
H-AC
H-AD
I-J
I-K
I-L
I-M
I-N
I-O
I-P
I-Q
I-R
I-S
I-T
I-U
I-V
I-W
I-X
I-Y
I-Z
I-AA
I-AB
I-AC
I-AD
J-K
J-L
J-M
J-N
J-O
J-P
J-Q
J-R
J-S

J-T
J-U
J-V
J-W
J-X
J-Y
J-Z
J-AA
J-AB
J-AC
J-AD
K-L
K-M
K-N
K-O
K-P
K-Q
K-R
K-S
K-T
K-U
K-V
K-W
K-X
K-Y
K-Z
K-AA
K-AB
K-AC
K-AD
L-M
L-N
L-O
L-P
L-Q
L-R
L-S
L-T
L-U
L-V
L-W
L-X
L-Y
L-Z
L-AA
L-AB
L-AC

L-AD
M-N
M-O
M-P
M-Q
M-R
M-S
M-T
M-U
M-V
M-W
M-X
M-Y
M-Z
M-AA
M-AB
M-AC
M-AD
N-O
N-P
N-Q
N-R
N-S
N-T
N-U
N-V
N-W
N-X
N-Y
N-Z
N-AA
N-AB
N-AC
N-AD
O-P
O-Q
O-R
O-S
O-T
O-U
O-V
O-W
O-X
O-Y
O-Z
O-AA
O-AB

O-AC
O-AD
P-Q
P-R
P-S
P-T
P-U
P-V
P-W
P-X
P-Y
P-Z
P-AA
P-AB
P-AC
P-AD
Q-R
Q-S
Q-T
Q-U
Q-V
Q-W
Q-X
Q-Y
Q-Z
Q-AA
Q-AB
Q-AC
Q-AD
R-S
R-T
R-U
R-V
R-W
R-X
R-Y
R-Z
R-AA
R-AB
R-AC
R-AD
S-T
S-U
S-V
S-W
S-X
S-Y

S-Z
S-AA
S-AB
S-AC
S-AD
T-U
T-V
T-W
T-X
T-Y
T-Z
T-AA
T-AB
T-AC
T-AD
U-V
U-W
U-X
U-Y
U-Z
U-AA
U-AB
U-AC
U-AD
V-W
V-X
V-Y
V-Z
V-AA
V-AB
V-AC
V-AD
W-X
W-Y
W-Z
W-AA
W-AB
W-AC
W-AD
X-Y
X-Z
X-AA
X-AB
X-AC
X-AD
Y-Z
Y-AA

Y-AB
Y-AC
Y-AD
Z-AA
Z-AB
Z-AC
Z-AD
AA-AB
AA-AC
AA-AD
AB-AC
AB-AD

Treatment	Holm-Šídák's multiple comparisons	Mean Diff.	Below thre:	Summary
Media vs. FCCP	Media vs. 5 µM FCCP	193.3	Yes	****
Media vs. TNFa	Media vs. 250 pg/mL	-32.29	No	ns
Media vs. TNFa	Media vs. 500 pg/mL	-41.08	Yes	**
Media vs. TNFa	Media vs. 750 pg/mL	-16.04	No	ns
Media vs. TNFa	Media vs. 1000 pg/mL	-9.316	No	ns
Media vs. Butyrate	Media vs. 500 µM	46.02	Yes	**
Media vs. Butyrate	Media vs. 1000 µM	21.26	No	ns
Media vs. Butyrate	Media vs. 2500 µM	-12.4	No	ns
Media vs. Butyrate	Media vs. 5000 µM	-12.96	No	ns
Media vs. Combo	Media vs. 250 pg/mL	-10.9	No	ns
Media vs. Combo	Media vs. 500 pg/mL	-25.49	No	ns
Media vs. Combo	Media vs. 750 pg/mL	11.89	No	ns
Media vs. Combo	Media vs. 1000 pg/mL	19.27	No	ns
FCCP vs. TNFa	5 µM FCCP vs. 250 pg/mL	-225.6	Yes	****
FCCP vs. TNFa	5 µM FCCP vs. 500 pg/mL	-234.4	Yes	****
FCCP vs. TNFa	5 µM FCCP vs. 750 pg/mL	-209.3	Yes	****
FCCP vs. TNFa	5 µM FCCP vs. 1000 pg/mL	-202.6	Yes	****
FCCP vs. Butyrate	5 µM FCCP vs. 500 µM	-147.3	Yes	****
FCCP vs. Butyrate	5 µM FCCP vs. 1000 µM	-172	Yes	****
FCCP vs. Butyrate	5 µM FCCP vs. 2500 µM	-205.7	Yes	****
FCCP vs. Butyrate	5 µM FCCP vs. 5000 µM	-206.2	Yes	****
FCCP vs. Combo	5 µM FCCP vs. 250 pg/mL	-204.2	Yes	****
FCCP vs. Combo	5 µM FCCP vs. 500 pg/mL	-218.8	Yes	****
FCCP vs. Combo	5 µM FCCP vs. 750 pg/mL	-181.4	Yes	****
FCCP vs. Combo	5 µM FCCP vs. 1000 pg/mL	-174	Yes	****
TNFa vs. TNFa	250 pg/mL vs. 500 pg/mL	-8.793	No	ns
TNFa vs. TNFa	250 pg/mL vs. 750 pg/mL	16.25	No	ns
TNFa vs. TNFa	250 pg/mL vs. 1000 pg/mL	22.97	No	ns
TNFa vs. butyrate	250 pg/mL vs. 500 µM	78.31	Yes	****
TNFa vs. butyrate	250 pg/mL vs. 1000 µM	53.55	Yes	***
TNFa vs. butyrate	250 pg/mL vs. 2500 µM	19.89	No	ns
TNFa vs. butyrate	250 pg/mL vs. 5000 µM	19.33	No	ns
TNFa vs. Combo	250 pg/mL vs. 250 pg/mL	21.39	No	ns
TNFa vs. Combo	250 pg/mL vs. 500 pg/mL	6.804	No	ns
TNFa vs. Combo	250 pg/mL vs. 750 pg/mL	44.18	Yes	**
TNFa vs. Combo	250 pg/mL vs. 1000 pg/mL	51.56	Yes	***
TNFa vs. TNFa	500 pg/mL vs. 750 pg/mL	25.04	No	ns
TNFa vs. TNFa	500 pg/mL vs. 1000 pg/mL	31.77	No	ns
TNFa vs. butyrate	500 pg/mL vs. 500 µM	87.1	Yes	****
TNFa vs. butyrate	500 pg/mL vs. 1000 µM	62.34	Yes	****
TNFa vs. butyrate	500 pg/mL vs. 2500 µM	28.69	No	ns
TNFa vs. butyrate	500 pg/mL vs. 5000 µM	28.12	No	ns
TNFa vs. Combo	500 pg/mL vs. 250 pg/mL	30.18	No	ns
TNFa vs. Combo	500 pg/mL vs. 500 pg/mL	15.6	No	ns
TNFa vs. Combo	500 pg/mL vs. 750 pg/mL	52.97	Yes	***
TNFa vs. Combo	500 pg/mL vs. 1000 pg/mL	60.36	Yes	****

TNFa vs. TNFa	750 pg/mL vs. 1000 pg/mL	6.723	No	ns
TNFa vs. butyrate	750 pg/mL vs. 500 µM	62.06	Yes	****
TNFa vs. butyrate	750 pg/mL vs. 1000 µM	37.3	No	ns
TNFa vs. butyrate	750 pg/mL vs. 2500 µM	3.644	No	ns
TNFa vs. butyrate	750 pg/mL vs. 5000 µM	3.079	No	ns
TNFa vs. Combo	750 pg/mL vs. 250 pg/mL	5.139	No	ns
TNFa vs. Combo	750 pg/mL vs. 500 pg/mL	-9.446	No	ns
TNFa vs. Combo	750 pg/mL vs. 750 pg/mL	27.93	No	ns
TNFa vs. Combo	750 pg/mL vs. 1000 pg/mL	35.31	No	ns
TNFa vs. butyrate	1000 pg/mL vs. 500 µM	55.33	Yes	***
TNFa vs. butyrate	1000 pg/mL vs. 1000 µM	30.58	No	ns
TNFa vs. butyrate	1000 pg/mL vs. 2500 µM	-3.079	No	ns
TNFa vs. butyrate	1000 pg/mL vs. 5000 µM	-3.644	No	ns
TNFa vs. Combo	1000 pg/mL vs. 250 pg/mL	-1.585	No	ns
TNFa vs. Combo	1000 pg/mL vs. 500 pg/mL	-16.17	No	ns
TNFa vs. Combo	1000 pg/mL vs. 750 pg/mL	21.2	No	ns
TNFa vs. Combo	1000 pg/mL vs. 1000 pg/mL	28.59	No	ns
Butyrate vs. Butyrate	500 µM vs. 1000 µM	-24.76	No	ns
Butyrate vs. Butyrate	500 µM vs. 2500 µM	-58.41	Yes	****
Butyrate vs. Butyrate	500 µM vs. 5000 µM	-58.98	Yes	****
Butyrate vs. Combo	500 µM vs. 250 pg/mL	-56.92	Yes	***
Butyrate vs. Combo	500 µM vs. 500 pg/mL	-71.5	Yes	****
Butyrate vs. Combo	500 µM vs. 750 pg/mL	-34.13	No	ns
Butyrate vs. Combo	500 µM vs. 1000 pg/mL	-26.74	No	ns
Butyrate vs. Butyrate	1000 µM vs. 2500 µM	-33.66	No	ns
Butyrate vs. Butyrate	1000 µM vs. 5000 µM	-34.22	No	ns
Butyrate vs. Combo	1000 µM vs. 250 pg/mL	-32.16	No	ns
Butyrate vs. Combo	1000 µM vs. 500 pg/mL	-46.75	Yes	**
Butyrate vs. Combo	1000 µM vs. 750 pg/mL	-9.375	No	ns
Butyrate vs. Combo	1000 µM vs. 1000 pg/mL	-1.987	No	ns
Butyrate vs. Butyrate	2500 µM vs. 5000 µM	-0.5652	No	ns
Butyrate vs. Combo	2500 µM vs. 250 pg/mL	1.495	No	ns
Butyrate vs. Combo	2500 µM vs. 500 pg/mL	-13.09	No	ns
Butyrate vs. Combo	2500 µM vs. 750 pg/mL	24.28	No	ns
Butyrate vs. Combo	2500 µM vs. 1000 pg/mL	31.67	No	ns
Butyrate vs. Combo	5000 µM vs. 250 pg/mL	2.06	No	ns
Butyrate vs. Combo	5000 µM vs. 500 pg/mL	-12.53	No	ns
Butyrate vs. Combo	5000 µM vs. 750 pg/mL	24.85	No	ns
Butyrate vs. Combo	5000 µM vs. 1000 pg/mL	32.24	No	ns
Combo vs. Combo	250 pg/mL vs. 500 pg/mL	-14.59	No	ns
Combo vs. Combo	250 pg/mL vs. 750 pg/mL	22.79	No	ns
Combo vs. Combo	250 pg/mL vs. 1000 pg/mL	30.18	No	ns
Combo vs. Combo	500 pg/mL vs. 750 pg/mL	37.37	No	ns
Combo vs. Combo	500 pg/mL vs. 1000 pg/mL	44.76	Yes	**
Combo vs. Combo	750 pg/mL vs. 1000 pg/mL	7.388	No	ns

A-B
A-C
A-D
A-E
A-F
A-G
A-H
A-I
A-J
A-K
A-L
A-M
A-N
B-C
B-D
B-E
B-F
B-G
B-H
B-I
B-J
B-K
B-L
B-M
B-N
C-D
C-E
C-F
C-G
C-H
C-I
C-J
C-K
C-L
C-M
C-N
D-E
D-F
D-G
D-H
D-I
D-J
D-K
D-L
D-M
D-N

E-F
E-G
E-H
E-I
E-J
E-K
E-L
E-M
E-N
F-G
F-H
F-I
F-J
F-K
F-L
F-M
F-N
G-H
G-I
G-J
G-K
G-L
G-M
G-N
H-I
H-J
H-K
H-L
H-M
H-N
I-J
I-K
I-L
I-M
I-N
J-K
J-L
J-M
J-N
K-L
K-M
K-N
L-M
L-N

	Holm-Šídák's multiple comparisons test	Mean Diff.	Below thre:	Summary
Media vs. FCCP	Media vs. 5 µM FCCP	18.54	Yes	**
Media vs. TNFa	Media vs. 250 pg/mL	10.25	No	ns
Media vs. TNFa	Media vs. 500 pg/mL	14.34	No	ns
Media vs. TNFa	Media vs. 750 pg/mL	18.79	Yes	**
Media vs. TNFa	Media vs. 1000 pg/mL	19.35	Yes	**
Media vs. Butyrate	Media vs. 500 µM	-3.184	No	ns
Media vs. Butyrate	Media vs. 1000 µM	-9.599	No	ns
Media vs. Butyrate	Media vs. 2500 µM	-9.378	No	ns
Media vs. Butyrate	Media vs. 5000 µM	-8	No	ns
Media vs. Combo	Media vs. 250 pg/mL	-2.184	No	ns
Media vs. Combo	Media vs. 500 pg/mL	-4.317	No	ns
Media vs. Combo	Media vs. 750 pg/mL	15.55	Yes	*
Media vs. Combo	Media vs. 1000 pg/mL	11.59	No	ns
FCCP vs. TNFa	5 µM FCCP vs. 250 pg/mL	-8.288	No	ns
FCCP vs. TNFa	5 µM FCCP vs. 500 pg/mL	-4.194	No	ns
FCCP vs. TNFa	5 µM FCCP vs. 750 pg/mL	0.2541	No	ns
FCCP vs. TNFa	5 µM FCCP vs. 1000 pg/mL	0.8151	No	ns
FCCP vs. Butyrate	5 µM FCCP vs. 500 µM	-21.72	Yes	**
FCCP vs. Butyrate	5 µM FCCP vs. 1000 µM	-28.14	Yes	****
FCCP vs. Butyrate	5 µM FCCP vs. 2500 µM	-27.92	Yes	****
FCCP vs. Butyrate	5 µM FCCP vs. 5000 µM	-26.54	Yes	****
FCCP vs. Combo	5 µM FCCP vs. 250 pg/mL	-20.72	Yes	**
FCCP vs. Combo	5 µM FCCP vs. 500 pg/mL	-22.85	Yes	**
FCCP vs. Combo	5 µM FCCP vs. 750 pg/mL	-2.99	No	ns
FCCP vs. Combo	5 µM FCCP vs. 1000 pg/mL	-6.949	No	ns
TNFa vs. TNFa	250 pg/mL vs. 500 pg/mL	4.095	No	ns
TNFa vs. TNFa	250 pg/mL vs. 750 pg/mL	8.542	No	ns
TNFa vs. TNFa	250 pg/mL vs. 1000 pg/mL	9.103	No	ns
TNFa vs. butyrate	250 pg/mL vs. 500 µM	-13.43	No	ns
TNFa vs. butyrate	250 pg/mL vs. 1000 µM	-19.85	Yes	**
TNFa vs. butyrate	250 pg/mL vs. 2500 µM	-19.63	Yes	**
TNFa vs. butyrate	250 pg/mL vs. 5000 µM	-18.25	Yes	*
TNFa vs. Combo	250 pg/mL vs. 250 pg/mL	-12.43	No	ns
TNFa vs. Combo	250 pg/mL vs. 500 pg/mL	-14.57	No	ns
TNFa vs. Combo	250 pg/mL vs. 750 pg/mL	5.299	No	ns
TNFa vs. Combo	250 pg/mL vs. 1000 pg/mL	1.34	No	ns
TNFa vs. TNFa	500 pg/mL vs. 750 pg/mL	4.448	No	ns
TNFa vs. TNFa	500 pg/mL vs. 1000 pg/mL	5.009	No	ns
TNFa vs. butyrate	500 pg/mL vs. 500 µM	-17.53	Yes	*
TNFa vs. butyrate	500 pg/mL vs. 1000 µM	-23.94	Yes	***
TNFa vs. butyrate	500 pg/mL vs. 2500 µM	-23.72	Yes	***
TNFa vs. butyrate	500 pg/mL vs. 5000 µM	-22.34	Yes	**
TNFa vs. Combo	500 pg/mL vs. 250 pg/mL	-16.53	No	ns
TNFa vs. Combo	500 pg/mL vs. 500 pg/mL	-18.66	Yes	*
TNFa vs. Combo	500 pg/mL vs. 750 pg/mL	1.204	No	ns
TNFa vs. Combo	500 pg/mL vs. 1000 pg/mL	-2.755	No	ns

TNFa vs. TNFa	750 pg/mL vs. 1000 pg/mL	0.561	No	ns
TNFa vs. butyrate	750 pg/mL vs. 500 µM	-21.98	Yes	**
TNFa vs. butyrate	750 pg/mL vs. 1000 µM	-28.39	Yes	****
TNFa vs. butyrate	750 pg/mL vs. 2500 µM	-28.17	Yes	****
TNFa vs. butyrate	750 pg/mL vs. 5000 µM	-26.79	Yes	****
TNFa vs. Combo	750 pg/mL vs. 250 pg/mL	-20.98	Yes	**
TNFa vs. Combo	750 pg/mL vs. 500 pg/mL	-23.11	Yes	***
TNFa vs. Combo	750 pg/mL vs. 750 pg/mL	-3.244	No	ns
TNFa vs. Combo	750 pg/mL vs. 1000 pg/mL	-7.203	No	ns
TNFa vs. butyrate	1000 pg/mL vs. 500 µM	-22.54	Yes	**
TNFa vs. butyrate	1000 pg/mL vs. 1000 µM	-28.95	Yes	****
TNFa vs. butyrate	1000 pg/mL vs. 2500 µM	-28.73	Yes	****
TNFa vs. butyrate	1000 pg/mL vs. 5000 µM	-27.35	Yes	****
TNFa vs. Combo	1000 pg/mL vs. 250 pg/mL	-21.54	Yes	**
TNFa vs. Combo	1000 pg/mL vs. 500 pg/mL	-23.67	Yes	***
TNFa vs. Combo	1000 pg/mL vs. 750 pg/mL	-3.805	No	ns
TNFa vs. Combo	1000 pg/mL vs. 1000 pg/mL	-7.764	No	ns
Butyrate vs. Butyrate	500 µM vs. 1000 µM	-6.415	No	ns
Butyrate vs. Butyrate	500 µM vs. 2500 µM	-6.194	No	ns
Butyrate vs. Butyrate	500 µM vs. 5000 µM	-4.816	No	ns
Butyrate vs. Combo	500 µM vs. 250 pg/mL	0.9997	No	ns
Butyrate vs. Combo	500 µM vs. 500 pg/mL	-1.133	No	ns
Butyrate vs. Combo	500 µM vs. 750 pg/mL	18.73	Yes	*
Butyrate vs. Combo	500 µM vs. 1000 pg/mL	14.77	No	ns
Butyrate vs. Butyrate	1000 µM vs. 2500 µM	0.2209	No	ns
Butyrate vs. Butyrate	1000 µM vs. 5000 µM	1.599	No	ns
Butyrate vs. Combo	1000 µM vs. 250 pg/mL	7.415	No	ns
Butyrate vs. Combo	1000 µM vs. 500 pg/mL	5.282	No	ns
Butyrate vs. Combo	1000 µM vs. 750 pg/mL	25.15	Yes	***
Butyrate vs. Combo	1000 µM vs. 1000 pg/mL	21.19	Yes	**
Butyrate vs. Butyrate	2500 µM vs. 5000 µM	1.378	No	ns
Butyrate vs. Combo	2500 µM vs. 250 pg/mL	7.194	No	ns
Butyrate vs. Combo	2500 µM vs. 500 pg/mL	5.061	No	ns
Butyrate vs. Combo	2500 µM vs. 750 pg/mL	24.93	Yes	***
Butyrate vs. Combo	2500 µM vs. 1000 pg/mL	20.97	Yes	**
Butyrate vs. Combo	5000 µM vs. 250 pg/mL	5.816	No	ns
Butyrate vs. Combo	5000 µM vs. 500 pg/mL	3.683	No	ns
Butyrate vs. Combo	5000 µM vs. 750 pg/mL	23.55	Yes	***
Butyrate vs. Combo	5000 µM vs. 1000 pg/mL	19.59	Yes	**
Combo vs. Combo	250 pg/mL vs. 500 pg/mL	-2.132	No	ns
Combo vs. Combo	250 pg/mL vs. 750 pg/mL	17.73	Yes	*
Combo vs. Combo	250 pg/mL vs. 1000 pg/mL	13.77	No	ns
Combo vs. Combo	500 pg/mL vs. 750 pg/mL	19.86	Yes	**
Combo vs. Combo	500 pg/mL vs. 1000 pg/mL	15.91	No	ns
Combo vs. Combo	750 pg/mL vs. 1000 pg/mL	-3.959	No	ns

A-B
A-C
A-D
A-E
A-F
A-G
A-H
A-I
A-J
A-K
A-L
A-M
A-N
B-C
B-D
B-E
B-F
B-G
B-H
B-I
B-J
B-K
B-L
B-M
B-N
C-D
C-E
C-F
C-G
C-H
C-I
C-J
C-K
C-L
C-M
C-N
D-E
D-F
D-G
D-H
D-I
D-J
D-K
D-L
D-M
D-N

E-F
E-G
E-H
E-I
E-J
E-K
E-L
E-M
E-N
F-G
F-H
F-I
F-J
F-K
F-L
F-M
F-N
G-H
G-I
G-J
G-K
G-L
G-M
G-N
H-I
H-J
H-K
H-L
H-M
H-N
I-J
I-K
I-L
I-M
I-N
J-K
J-L
J-M
J-N
K-L
K-M
K-N
L-M
L-N

Treatment	Dunn's multiple comparisons test	Mean rank	Significant'	Summary
Media vs. MEN	Media vs. 50µM MEN	-21.83	No	ns
Media vs. TNFa	Media vs. 50pg/mL	-10.13	No	ns
Media vs. TNFa	Media vs. 100pg/mL	2.267	No	ns
Media vs. TNFa	Media vs. 150pg/mL	32.58	No	ns
Media vs. TNFa	Media vs. 200pg/mL	30.67	No	ns
Media vs. TNFa	Media vs. 250pg/mL	55.25	No	ns
Media vs. TNFa	Media vs. 500pg/mL	62.83	No	ns
Media vs. TNFa	Media vs. 1000pg/mL	58.67	No	ns
Media vs. TNFa	Media vs. 2500pg/mL	111.5	No	ns
Media vs. TNFa	Media vs. 5000pg/mL	138	Yes	**
Media vs. Butyrate	Media vs. 50µM	-2	No	ns
Media vs. Butyrate	Media vs. 75µM	30.58	No	ns
Media vs. Butyrate	Media vs. 100µM	20.17	No	ns
Media vs. Butyrate	Media vs. 250µM	38.17	No	ns
Media vs. Butyrate	Media vs. 500µM	51.67	No	ns
Media vs. Butyrate	Media vs. 750µM	38.17	No	ns
Media vs. Butyrate	Media vs. 1000µM	48.5	No	ns
Media vs. Butyrate	Media vs. 2500µM	75.67	No	ns
Media vs. Butyrate	Media vs. 5000µM	84.17	No	ns
Media vs. Butyrate	Media vs. 7500µM	92.5	No	ns
Media vs. Combo	Media vs. 50pg/mL	61.5	No	ns
Media vs. Combo	Media vs. 100pg/mL	55.17	No	ns
Media vs. Combo	Media vs. 150pg/mL	88.17	No	ns
Media vs. Combo	Media vs. 200pg/mL	110.5	No	ns
Media vs. Combo	Media vs. 250pg/mL	99.5	No	ns
Media vs. Combo	Media vs. 500pg/mL	124.9	Yes	*
Media vs. Combo	Media vs. 1000pg/mL	133.2	Yes	**
Media vs. Combo	Media vs. 2500pg/mL	143.3	Yes	***
Media vs. Combo	Media vs. 5000pg/mL	148.7	Yes	***
MEN vs. TNFa	50µM MEN vs. 50pg/mL	11.7	No	ns
MEN vs. TNFa	50µM MEN vs. 100pg/mL	24.1	No	ns
MEN vs. TNFa	50µM MEN vs. 150pg/mL	54.42	No	ns
MEN vs. TNFa	50µM MEN vs. 200pg/mL	52.5	No	ns
MEN vs. TNFa	50µM MEN vs. 250pg/mL	77.08	No	ns
MEN vs. TNFa	50µM MEN vs. 500pg/mL	84.67	No	ns
MEN vs. TNFa	50µM MEN vs. 1000pg/mL	80.5	No	ns
MEN vs. TNFa	50µM MEN vs. 2500pg/mL	133.3	Yes	**
MEN vs. TNFa	50µM MEN vs. 5000pg/mL	159.8	Yes	****
MEN vs. Butyrate	50µM MEN vs. 50µM	19.83	No	ns
MEN vs. Butyrate	50µM MEN vs. 75µM	52.42	No	ns
MEN vs. Butyrate	50µM MEN vs. 100µM	42	No	ns
MEN vs. Butyrate	50µM MEN vs. 250µM	60	No	ns
MEN vs. Butyrate	50µM MEN vs. 500µM	73.5	No	ns
MEN vs. Butyrate	50µM MEN vs. 750µM	60	No	ns
MEN vs. Butyrate	50µM MEN vs. 1000µM	70.33	No	ns
MEN vs. Butyrate	50µM MEN vs. 2500µM	97.5	No	ns

MEN vs. Butyrate	50µM MEN vs. 5000µM	106	No	ns
MEN vs. Butyrate	50µM MEN vs. 7500µM	114.3	No	ns
MEN vs. Combo	50µM MEN vs. 50pg/mL	83.33	No	ns
MEN vs. Combo	50µM MEN vs. 100pg/mL	77	No	ns
MEN vs. Combo	50µM MEN vs. 150pg/mL	110	No	ns
MEN vs. Combo	50µM MEN vs. 200pg/mL	132.3	Yes	**
MEN vs. Combo	50µM MEN vs. 250pg/mL	121.3	Yes	*
MEN vs. Combo	50µM MEN vs. 500pg/mL	146.8	Yes	***
MEN vs. Combo	50µM MEN vs. 1000pg/mL	155	Yes	****
MEN vs. Combo	50µM MEN vs. 2500pg/mL	165.2	Yes	****
MEN vs. Combo	50µM MEN vs. 5000pg/mL	170.5	Yes	****
TNFa vs. TNFa	50pg/mL vs. 100pg/mL	12.4	No	ns
TNFa vs. TNFa	50pg/mL vs. 150pg/mL	42.72	No	ns
TNFa vs. TNFa	50pg/mL vs. 200pg/mL	40.8	No	ns
TNFa vs. TNFa	50pg/mL vs. 250pg/mL	65.38	No	ns
TNFa vs. TNFa	50pg/mL vs. 500pg/mL	72.97	No	ns
TNFa vs. TNFa	50pg/mL vs. 1000pg/mL	68.8	No	ns
TNFa vs. TNFa	50pg/mL vs. 2500pg/mL	121.6	Yes	*
TNFa vs. TNFa	50pg/mL vs. 5000pg/mL	148.1	Yes	***
TNFa vs. Butyrate	50pg/mL vs. 50µM	8.133	No	ns
TNFa vs. Butyrate	50pg/mL vs. 75µM	40.72	No	ns
TNFa vs. Butyrate	50pg/mL vs. 100µM	30.3	No	ns
TNFa vs. Butyrate	50pg/mL vs. 250µM	48.3	No	ns
TNFa vs. Butyrate	50pg/mL vs. 500µM	61.8	No	ns
TNFa vs. Butyrate	50pg/mL vs. 750µM	48.3	No	ns
TNFa vs. Butyrate	50pg/mL vs. 1000µM	58.63	No	ns
TNFa vs. Butyrate	50pg/mL vs. 2500µM	85.8	No	ns
TNFa vs. Butyrate	50pg/mL vs. 5000µM	94.3	No	ns
TNFa vs. Butyrate	50pg/mL vs. 7500µM	102.6	No	ns
TNFa vs Combo	50pg/mL vs. 50pg/mL	71.63	No	ns
TNFa vs Combo	50pg/mL vs. 100pg/mL	65.3	No	ns
TNFa vs Combo	50pg/mL vs. 150pg/mL	98.3	No	ns
TNFa vs Combo	50pg/mL vs. 200pg/mL	120.6	Yes	*
TNFa vs Combo	50pg/mL vs. 250pg/mL	109.6	No	ns
TNFa vs Combo	50pg/mL vs. 500pg/mL	135.1	Yes	**
TNFa vs Combo	50pg/mL vs. 1000pg/mL	143.3	Yes	**
TNFa vs Combo	50pg/mL vs. 2500pg/mL	153.5	Yes	***
TNFa vs Combo	50pg/mL vs. 5000pg/mL	158.8	Yes	***
TNFa vs. TNFa	100pg/mL vs. 150pg/mL	30.32	No	ns
TNFa vs. TNFa	100pg/mL vs. 200pg/mL	28.4	No	ns
TNFa vs. TNFa	100pg/mL vs. 250pg/mL	52.98	No	ns
TNFa vs. TNFa	100pg/mL vs. 500pg/mL	60.57	No	ns
TNFa vs. TNFa	100pg/mL vs. 1000pg/mL	56.4	No	ns
TNFa vs. TNFa	100pg/mL vs. 2500pg/mL	109.2	No	ns
TNFa vs. TNFa	100pg/mL vs. 5000pg/mL	135.7	Yes	**
TNFa vs. Butyrate	100pg/mL vs. 50µM	-4.267	No	ns
TNFa vs. Butyrate	100pg/mL vs. 75µM	28.32	No	ns

TNFa vs. Butyrate	100pg/mL vs. 100µM	17.9	No	ns
TNFa vs. Butyrate	100pg/mL vs. 250µM	35.9	No	ns
TNFa vs. Butyrate	100pg/mL vs. 500µM	49.4	No	ns
TNFa vs. Butyrate	100pg/mL vs. 750µM	35.9	No	ns
TNFa vs. Butyrate	100pg/mL vs. 1000µM	46.23	No	ns
TNFa vs. Butyrate	100pg/mL vs. 2500µM	73.4	No	ns
TNFa vs. Butyrate	100pg/mL vs. 5000µM	81.9	No	ns
TNFa vs. Butyrate	100pg/mL vs. 7500µM	90.23	No	ns
TNFa vs Combo	100pg/mL vs. 50pg/mL	59.23	No	ns
TNFa vs Combo	100pg/mL vs. 100pg/mL	52.9	No	ns
TNFa vs Combo	100pg/mL vs. 150pg/mL	85.9	No	ns
TNFa vs Combo	100pg/mL vs. 200pg/mL	108.2	No	ns
TNFa vs Combo	100pg/mL vs. 250pg/mL	97.23	No	ns
TNFa vs Combo	100pg/mL vs. 500pg/mL	122.7	Yes	*
TNFa vs Combo	100pg/mL vs. 1000pg/mL	130.9	Yes	*
TNFa vs Combo	100pg/mL vs. 2500pg/mL	141.1	Yes	**
TNFa vs Combo	100pg/mL vs. 5000pg/mL	146.4	Yes	**
TNFa vs. TNFa	150pg/mL vs. 200pg/mL	-1.917	No	ns
TNFa vs. TNFa	150pg/mL vs. 250pg/mL	22.67	No	ns
TNFa vs. TNFa	150pg/mL vs. 500pg/mL	30.25	No	ns
TNFa vs. TNFa	150pg/mL vs. 1000pg/mL	26.08	No	ns
TNFa vs. TNFa	150pg/mL vs. 2500pg/mL	78.92	No	ns
TNFa vs. TNFa	150pg/mL vs. 5000pg/mL	105.4	No	ns
TNFa vs. Butyrate	150pg/mL vs. 50µM	-34.58	No	ns
TNFa vs. Butyrate	150pg/mL vs. 75µM	-2	No	ns
TNFa vs. Butyrate	150pg/mL vs. 100µM	-12.42	No	ns
TNFa vs. Butyrate	150pg/mL vs. 250µM	5.583	No	ns
TNFa vs. Butyrate	150pg/mL vs. 500µM	19.08	No	ns
TNFa vs. Butyrate	150pg/mL vs. 750µM	5.583	No	ns
TNFa vs. Butyrate	150pg/mL vs. 1000µM	15.92	No	ns
TNFa vs. Butyrate	150pg/mL vs. 2500µM	43.08	No	ns
TNFa vs. Butyrate	150pg/mL vs. 5000µM	51.58	No	ns
TNFa vs. Butyrate	150pg/mL vs. 7500µM	59.92	No	ns
TNFa vs Combo	150pg/mL vs. 50pg/mL	28.92	No	ns
TNFa vs Combo	150pg/mL vs. 100pg/mL	22.58	No	ns
TNFa vs Combo	150pg/mL vs. 150pg/mL	55.58	No	ns
TNFa vs Combo	150pg/mL vs. 200pg/mL	77.92	No	ns
TNFa vs Combo	150pg/mL vs. 250pg/mL	66.92	No	ns
TNFa vs Combo	150pg/mL vs. 500pg/mL	92.33	No	ns
TNFa vs Combo	150pg/mL vs. 1000pg/mL	100.6	No	ns
TNFa vs Combo	150pg/mL vs. 2500pg/mL	110.8	No	ns
TNFa vs Combo	150pg/mL vs. 5000pg/mL	116.1	Yes	*
TNFa vs. TNFa	200pg/mL vs. 250pg/mL	24.58	No	ns
TNFa vs. TNFa	200pg/mL vs. 500pg/mL	32.17	No	ns
TNFa vs. TNFa	200pg/mL vs. 1000pg/mL	28	No	ns
TNFa vs. TNFa	200pg/mL vs. 2500pg/mL	80.83	No	ns
TNFa vs. TNFa	200pg/mL vs. 5000pg/mL	107.3	No	ns

TNFa vs. Butyrate	200pg/mL vs. 50µM	-32.67	No	ns
TNFa vs. Butyrate	200pg/mL vs. 75µM	-0.08333	No	ns
TNFa vs. Butyrate	200pg/mL vs. 100µM	-10.5	No	ns
TNFa vs. Butyrate	200pg/mL vs. 250µM	7.5	No	ns
TNFa vs. Butyrate	200pg/mL vs. 500µM	21	No	ns
TNFa vs. Butyrate	200pg/mL vs. 750µM	7.5	No	ns
TNFa vs. Butyrate	200pg/mL vs. 1000µM	17.83	No	ns
TNFa vs. Butyrate	200pg/mL vs. 2500µM	45	No	ns
TNFa vs. Butyrate	200pg/mL vs. 5000µM	53.5	No	ns
TNFa vs. Butyrate	200pg/mL vs. 7500µM	61.83	No	ns
TNFa vs Combo	200pg/mL vs. 50pg/mL	30.83	No	ns
TNFa vs Combo	200pg/mL vs. 100pg/mL	24.5	No	ns
TNFa vs Combo	200pg/mL vs. 150pg/mL	57.5	No	ns
TNFa vs Combo	200pg/mL vs. 200pg/mL	79.83	No	ns
TNFa vs Combo	200pg/mL vs. 250pg/mL	68.83	No	ns
TNFa vs Combo	200pg/mL vs. 500pg/mL	94.25	No	ns
TNFa vs Combo	200pg/mL vs. 1000pg/mL	102.5	No	ns
TNFa vs Combo	200pg/mL vs. 2500pg/mL	112.7	No	ns
TNFa vs Combo	200pg/mL vs. 5000pg/mL	118	Yes	*
TNFa vs. TNFa	250pg/mL vs. 500pg/mL	7.583	No	ns
TNFa vs. TNFa	250pg/mL vs. 1000pg/mL	3.417	No	ns
TNFa vs. TNFa	250pg/mL vs. 2500pg/mL	56.25	No	ns
TNFa vs. TNFa	250pg/mL vs. 5000pg/mL	82.75	No	ns
TNFa vs. Butyrate	250pg/mL vs. 50µM	-57.25	No	ns
TNFa vs. Butyrate	250pg/mL vs. 75µM	-24.67	No	ns
TNFa vs. Butyrate	250pg/mL vs. 100µM	-35.08	No	ns
TNFa vs. Butyrate	250pg/mL vs. 250µM	-17.08	No	ns
TNFa vs. Butyrate	250pg/mL vs. 500µM	-3.583	No	ns
TNFa vs. Butyrate	250pg/mL vs. 750µM	-17.08	No	ns
TNFa vs. Butyrate	250pg/mL vs. 1000µM	-6.75	No	ns
TNFa vs. Butyrate	250pg/mL vs. 2500µM	20.42	No	ns
TNFa vs. Butyrate	250pg/mL vs. 5000µM	28.92	No	ns
TNFa vs. Butyrate	250pg/mL vs. 7500µM	37.25	No	ns
TNFa vs Combo	250pg/mL vs. 50pg/mL	6.25	No	ns
TNFa vs Combo	250pg/mL vs. 100pg/mL	-0.08333	No	ns
TNFa vs Combo	250pg/mL vs. 150pg/mL	32.92	No	ns
TNFa vs Combo	250pg/mL vs. 200pg/mL	55.25	No	ns
TNFa vs Combo	250pg/mL vs. 250pg/mL	44.25	No	ns
TNFa vs Combo	250pg/mL vs. 500pg/mL	69.67	No	ns
TNFa vs Combo	250pg/mL vs. 1000pg/mL	77.92	No	ns
TNFa vs Combo	250pg/mL vs. 2500pg/mL	88.08	No	ns
TNFa vs Combo	250pg/mL vs. 5000pg/mL	93.42	No	ns
TNFa vs. TNFa	500pg/mL vs. 1000pg/mL	-4.167	No	ns
TNFa vs. TNFa	500pg/mL vs. 2500pg/mL	48.67	No	ns
TNFa vs. TNFa	500pg/mL vs. 5000pg/mL	75.17	No	ns
TNFa vs. Butyrate	500pg/mL vs. 50µM	-64.83	No	ns
TNFa vs. Butyrate	500pg/mL vs. 75µM	-32.25	No	ns

TNFa vs. Butyrate	500pg/mL vs. 100µM	-42.67	No	ns
TNFa vs. Butyrate	500pg/mL vs. 250µM	-24.67	No	ns
TNFa vs. Butyrate	500pg/mL vs. 500µM	-11.17	No	ns
TNFa vs. Butyrate	500pg/mL vs. 750µM	-24.67	No	ns
TNFa vs. Butyrate	500pg/mL vs. 1000µM	-14.33	No	ns
TNFa vs. Butyrate	500pg/mL vs. 2500µM	12.83	No	ns
TNFa vs. Butyrate	500pg/mL vs. 5000µM	21.33	No	ns
TNFa vs. Butyrate	500pg/mL vs. 7500µM	29.67	No	ns
TNFa vs Combo	500pg/mL vs. 50pg/mL	-1.333	No	ns
TNFa vs Combo	500pg/mL vs. 100pg/mL	-7.667	No	ns
TNFa vs Combo	500pg/mL vs. 150pg/mL	25.33	No	ns
TNFa vs Combo	500pg/mL vs. 200pg/mL	47.67	No	ns
TNFa vs Combo	500pg/mL vs. 250pg/mL	36.67	No	ns
TNFa vs Combo	500pg/mL vs. 500pg/mL	62.08	No	ns
TNFa vs Combo	500pg/mL vs. 1000pg/mL	70.33	No	ns
TNFa vs Combo	500pg/mL vs. 2500pg/mL	80.5	No	ns
TNFa vs Combo	500pg/mL vs. 5000pg/mL	85.83	No	ns
TNFa vs. TNFa	1000pg/mL vs. 2500pg/mL	52.83	No	ns
TNFa vs. TNFa	1000pg/mL vs. 5000pg/mL	79.33	No	ns
TNFa vs. Butyrate	1000pg/mL vs. 50µM	-60.67	No	ns
TNFa vs. Butyrate	1000pg/mL vs. 75µM	-28.08	No	ns
TNFa vs. Butyrate	1000pg/mL vs. 100µM	-38.5	No	ns
TNFa vs. Butyrate	1000pg/mL vs. 250µM	-20.5	No	ns
TNFa vs. Butyrate	1000pg/mL vs. 500µM	-7	No	ns
TNFa vs. Butyrate	1000pg/mL vs. 750µM	-20.5	No	ns
TNFa vs. Butyrate	1000pg/mL vs. 1000µM	-10.17	No	ns
TNFa vs. Butyrate	1000pg/mL vs. 2500µM	17	No	ns
TNFa vs. Butyrate	1000pg/mL vs. 5000µM	25.5	No	ns
TNFa vs. Butyrate	1000pg/mL vs. 7500µM	33.83	No	ns
TNFa vs Combo	1000pg/mL vs. 50pg/mL	2.833	No	ns
TNFa vs Combo	1000pg/mL vs. 100pg/mL	-3.5	No	ns
TNFa vs Combo	1000pg/mL vs. 150pg/mL	29.5	No	ns
TNFa vs Combo	1000pg/mL vs. 200pg/mL	51.83	No	ns
TNFa vs Combo	1000pg/mL vs. 250pg/mL	40.83	No	ns
TNFa vs Combo	1000pg/mL vs. 500pg/mL	66.25	No	ns
TNFa vs Combo	1000pg/mL vs. 1000pg/mL	74.5	No	ns
TNFa vs Combo	1000pg/mL vs. 2500pg/mL	84.67	No	ns
TNFa vs Combo	1000pg/mL vs. 5000pg/mL	90	No	ns
TNFa vs. TNFa	2500pg/mL vs. 5000pg/mL	26.5	No	ns
TNFa vs. Butyrate	2500pg/mL vs. 50µM	-113.5	No	ns
TNFa vs. Butyrate	2500pg/mL vs. 75µM	-80.92	No	ns
TNFa vs. Butyrate	2500pg/mL vs. 100µM	-91.33	No	ns
TNFa vs. Butyrate	2500pg/mL vs. 250µM	-73.33	No	ns
TNFa vs. Butyrate	2500pg/mL vs. 500µM	-59.83	No	ns
TNFa vs. Butyrate	2500pg/mL vs. 750µM	-73.33	No	ns
TNFa vs. Butyrate	2500pg/mL vs. 1000µM	-63	No	ns
TNFa vs. Butyrate	2500pg/mL vs. 2500µM	-35.83	No	ns

TNFa vs. Butyrate	2500pg/mL vs. 5000µM	-27.33	No	ns
TNFa vs. Butyrate	2500pg/mL vs. 7500µM	-19	No	ns
TNFa vs Combo	2500pg/mL vs. 50pg/mL	-50	No	ns
TNFa vs Combo	2500pg/mL vs. 100pg/mL	-56.33	No	ns
TNFa vs Combo	2500pg/mL vs. 150pg/mL	-23.33	No	ns
TNFa vs Combo	2500pg/mL vs. 200pg/mL	-1	No	ns
TNFa vs Combo	2500pg/mL vs. 250pg/mL	-12	No	ns
TNFa vs Combo	2500pg/mL vs. 500pg/mL	13.42	No	ns
TNFa vs Combo	2500pg/mL vs. 1000pg/mL	21.67	No	ns
TNFa vs Combo	2500pg/mL vs. 2500pg/mL	31.83	No	ns
TNFa vs Combo	2500pg/mL vs. 5000pg/mL	37.17	No	ns
TNFa vs. Butyrate	5000pg/mL vs. 50µM	-140	Yes	**
TNFa vs. Butyrate	5000pg/mL vs. 75µM	-107.4	No	ns
TNFa vs. Butyrate	5000pg/mL vs. 100µM	-117.8	Yes	*
TNFa vs. Butyrate	5000pg/mL vs. 250µM	-99.83	No	ns
TNFa vs. Butyrate	5000pg/mL vs. 500µM	-86.33	No	ns
TNFa vs. Butyrate	5000pg/mL vs. 750µM	-99.83	No	ns
TNFa vs. Butyrate	5000pg/mL vs. 1000µM	-89.5	No	ns
TNFa vs. Butyrate	5000pg/mL vs. 2500µM	-62.33	No	ns
TNFa vs. Butyrate	5000pg/mL vs. 5000µM	-53.83	No	ns
TNFa vs. Butyrate	5000pg/mL vs. 7500µM	-45.5	No	ns
TNFa vs Combo	5000pg/mL vs. 50pg/mL	-76.5	No	ns
TNFa vs Combo	5000pg/mL vs. 100pg/mL	-82.83	No	ns
TNFa vs Combo	5000pg/mL vs. 150pg/mL	-49.83	No	ns
TNFa vs Combo	5000pg/mL vs. 200pg/mL	-27.5	No	ns
TNFa vs Combo	5000pg/mL vs. 250pg/mL	-38.5	No	ns
TNFa vs Combo	5000pg/mL vs. 500pg/mL	-13.08	No	ns
TNFa vs Combo	5000pg/mL vs. 1000pg/mL	-4.833	No	ns
TNFa vs Combo	5000pg/mL vs. 2500pg/mL	5.333	No	ns
TNFa vs Combo	5000pg/mL vs. 5000pg/mL	10.67	No	ns
Butyrate vs. Butyrate	50µM vs. 75µM	32.58	No	ns
Butyrate vs. Butyrate	50µM vs. 100µM	22.17	No	ns
Butyrate vs. Butyrate	50µM vs. 250µM	40.17	No	ns
Butyrate vs. Butyrate	50µM vs. 500µM	53.67	No	ns
Butyrate vs. Butyrate	50µM vs. 750µM	40.17	No	ns
Butyrate vs. Butyrate	50µM vs. 1000µM	50.5	No	ns
Butyrate vs. Butyrate	50µM vs. 2500µM	77.67	No	ns
Butyrate vs. Butyrate	50µM vs. 5000µM	86.17	No	ns
Butyrate vs. Butyrate	50µM vs. 7500µM	94.5	No	ns
Butyrate vs. Combo	50µM vs. 50pg/mL	63.5	No	ns
Butyrate vs. Combo	50µM vs. 100pg/mL	57.17	No	ns
Butyrate vs. Combo	50µM vs. 150pg/mL	90.17	No	ns
Butyrate vs. Combo	50µM vs. 200pg/mL	112.5	No	ns
Butyrate vs. Combo	50µM vs. 250pg/mL	101.5	No	ns
Butyrate vs. Combo	50µM vs. 500pg/mL	126.9	Yes	**
Butyrate vs. Combo	50µM vs. 1000pg/mL	135.2	Yes	**
Butyrate vs. Combo	50µM vs. 2500pg/mL	145.3	Yes	***

Butyrate vs. Combo	50µM vs. 5000pg/mL	150.7	Yes	***
Butyrate vs. Butyrate	75µM vs. 100µM	-10.42	No	ns
Butyrate vs. Butyrate	75µM vs. 250µM	7.583	No	ns
Butyrate vs. Butyrate	75µM vs. 500µM	21.08	No	ns
Butyrate vs. Butyrate	75µM vs. 750µM	7.583	No	ns
Butyrate vs. Butyrate	75µM vs. 1000µM	17.92	No	ns
Butyrate vs. Butyrate	75µM vs. 2500µM	45.08	No	ns
Butyrate vs. Butyrate	75µM vs. 5000µM	53.58	No	ns
Butyrate vs. Butyrate	75µM vs. 7500µM	61.92	No	ns
Butyrate vs. Combo	75µM vs. 50pg/mL	30.92	No	ns
Butyrate vs. Combo	75µM vs. 100pg/mL	24.58	No	ns
Butyrate vs. Combo	75µM vs. 150pg/mL	57.58	No	ns
Butyrate vs. Combo	75µM vs. 200pg/mL	79.92	No	ns
Butyrate vs. Combo	75µM vs. 250pg/mL	68.92	No	ns
Butyrate vs. Combo	75µM vs. 500pg/mL	94.33	No	ns
Butyrate vs. Combo	75µM vs. 1000pg/mL	102.6	No	ns
Butyrate vs. Combo	75µM vs. 2500pg/mL	112.8	No	ns
Butyrate vs. Combo	75µM vs. 5000pg/mL	118.1	Yes	*
Butyrate vs. Butyrate	100µM vs. 250µM	18	No	ns
Butyrate vs. Butyrate	100µM vs. 500µM	31.5	No	ns
Butyrate vs. Butyrate	100µM vs. 750µM	18	No	ns
Butyrate vs. Butyrate	100µM vs. 1000µM	28.33	No	ns
Butyrate vs. Butyrate	100µM vs. 2500µM	55.5	No	ns
Butyrate vs. Butyrate	100µM vs. 5000µM	64	No	ns
Butyrate vs. Butyrate	100µM vs. 7500µM	72.33	No	ns
Butyrate vs. Combo	100µM vs. 50pg/mL	41.33	No	ns
Butyrate vs. Combo	100µM vs. 100pg/mL	35	No	ns
Butyrate vs. Combo	100µM vs. 150pg/mL	68	No	ns
Butyrate vs. Combo	100µM vs. 200pg/mL	90.33	No	ns
Butyrate vs. Combo	100µM vs. 250pg/mL	79.33	No	ns
Butyrate vs. Combo	100µM vs. 500pg/mL	104.8	No	ns
Butyrate vs. Combo	100µM vs. 1000pg/mL	113	No	ns
Butyrate vs. Combo	100µM vs. 2500pg/mL	123.2	Yes	*
Butyrate vs. Combo	100µM vs. 5000pg/mL	128.5	Yes	**
Butyrate vs. Butyrate	250µM vs. 500µM	13.5	No	ns
Butyrate vs. Butyrate	250µM vs. 750µM	0	No	ns
Butyrate vs. Butyrate	250µM vs. 1000µM	10.33	No	ns
Butyrate vs. Butyrate	250µM vs. 2500µM	37.5	No	ns
Butyrate vs. Butyrate	250µM vs. 5000µM	46	No	ns
Butyrate vs. Butyrate	250µM vs. 7500µM	54.33	No	ns
Butyrate vs. Combo	250µM vs. 50pg/mL	23.33	No	ns
Butyrate vs. Combo	250µM vs. 100pg/mL	17	No	ns
Butyrate vs. Combo	250µM vs. 150pg/mL	50	No	ns
Butyrate vs. Combo	250µM vs. 200pg/mL	72.33	No	ns
Butyrate vs. Combo	250µM vs. 250pg/mL	61.33	No	ns
Butyrate vs. Combo	250µM vs. 500pg/mL	86.75	No	ns
Butyrate vs. Combo	250µM vs. 1000pg/mL	95	No	ns

Butyrate vs. Combo	250µM vs. 2500pg/mL	105.2	No	ns
Butyrate vs. Combo	250µM vs. 5000pg/mL	110.5	No	ns
Butyrate vs. Butyrate	500µM vs. 750µM	-13.5	No	ns
Butyrate vs. Butyrate	500µM vs. 1000µM	-3.167	No	ns
Butyrate vs. Butyrate	500µM vs. 2500µM	24	No	ns
Butyrate vs. Butyrate	500µM vs. 5000µM	32.5	No	ns
Butyrate vs. Butyrate	500µM vs. 7500µM	40.83	No	ns
Butyrate vs. Combo	500µM vs. 50pg/mL	9.833	No	ns
Butyrate vs. Combo	500µM vs. 100pg/mL	3.5	No	ns
Butyrate vs. Combo	500µM vs. 150pg/mL	36.5	No	ns
Butyrate vs. Combo	500µM vs. 200pg/mL	58.83	No	ns
Butyrate vs. Combo	500µM vs. 250pg/mL	47.83	No	ns
Butyrate vs. Combo	500µM vs. 500pg/mL	73.25	No	ns
Butyrate vs. Combo	500µM vs. 1000pg/mL	81.5	No	ns
Butyrate vs. Combo	500µM vs. 2500pg/mL	91.67	No	ns
Butyrate vs. Combo	500µM vs. 5000pg/mL	97	No	ns
Butyrate vs. Butyrate	750µM vs. 1000µM	10.33	No	ns
Butyrate vs. Butyrate	750µM vs. 2500µM	37.5	No	ns
Butyrate vs. Butyrate	750µM vs. 5000µM	46	No	ns
Butyrate vs. Butyrate	750µM vs. 7500µM	54.33	No	ns
Butyrate vs. Combo	750µM vs. 50pg/mL	23.33	No	ns
Butyrate vs. Combo	750µM vs. 100pg/mL	17	No	ns
Butyrate vs. Combo	750µM vs. 150pg/mL	50	No	ns
Butyrate vs. Combo	750µM vs. 200pg/mL	72.33	No	ns
Butyrate vs. Combo	750µM vs. 250pg/mL	61.33	No	ns
Butyrate vs. Combo	750µM vs. 500pg/mL	86.75	No	ns
Butyrate vs. Combo	750µM vs. 1000pg/mL	95	No	ns
Butyrate vs. Combo	750µM vs. 2500pg/mL	105.2	No	ns
Butyrate vs. Combo	750µM vs. 5000pg/mL	110.5	No	ns
Butyrate vs. Butyrate	1000µM vs. 2500µM	27.17	No	ns
Butyrate vs. Butyrate	1000µM vs. 5000µM	35.67	No	ns
Butyrate vs. Butyrate	1000µM vs. 7500µM	44	No	ns
Butyrate vs. Combo	1000µM vs. 50pg/mL	13	No	ns
Butyrate vs. Combo	1000µM vs. 100pg/mL	6.667	No	ns
Butyrate vs. Combo	1000µM vs. 150pg/mL	39.67	No	ns
Butyrate vs. Combo	1000µM vs. 200pg/mL	62	No	ns
Butyrate vs. Combo	1000µM vs. 250pg/mL	51	No	ns
Butyrate vs. Combo	1000µM vs. 500pg/mL	76.42	No	ns
Butyrate vs. Combo	1000µM vs. 1000pg/mL	84.67	No	ns
Butyrate vs. Combo	1000µM vs. 2500pg/mL	94.83	No	ns
Butyrate vs. Combo	1000µM vs. 5000pg/mL	100.2	No	ns
Butyrate vs. Butyrate	2500µM vs. 5000µM	8.5	No	ns
Butyrate vs. Butyrate	2500µM vs. 7500µM	16.83	No	ns
Butyrate vs. Combo	2500µM vs. 50pg/mL	-14.17	No	ns
Butyrate vs. Combo	2500µM vs. 100pg/mL	-20.5	No	ns
Butyrate vs. Combo	2500µM vs. 150pg/mL	12.5	No	ns
Butyrate vs. Combo	2500µM vs. 200pg/mL	34.83	No	ns

Butyrate vs. Combo	2500µM vs. 250pg/mL	23.83	No	ns
Butyrate vs. Combo	2500µM vs. 500pg/mL	49.25	No	ns
Butyrate vs. Combo	2500µM vs. 1000pg/mL	57.5	No	ns
Butyrate vs. Combo	2500µM vs. 2500pg/mL	67.67	No	ns
Butyrate vs. Combo	2500µM vs. 5000pg/mL	73	No	ns
Butyrate vs. Butyrate	5000µM vs. 7500µM	8.333	No	ns
Butyrate vs. Combo	5000µM vs. 50pg/mL	-22.67	No	ns
Butyrate vs. Combo	5000µM vs. 100pg/mL	-29	No	ns
Butyrate vs. Combo	5000µM vs. 150pg/mL	4	No	ns
Butyrate vs. Combo	5000µM vs. 200pg/mL	26.33	No	ns
Butyrate vs. Combo	5000µM vs. 250pg/mL	15.33	No	ns
Butyrate vs. Combo	5000µM vs. 500pg/mL	40.75	No	ns
Butyrate vs. Combo	5000µM vs. 1000pg/mL	49	No	ns
Butyrate vs. Combo	5000µM vs. 2500pg/mL	59.17	No	ns
Butyrate vs. Combo	5000µM vs. 5000pg/mL	64.5	No	ns
Butyrate vs. Combo	7500µM vs. 50pg/mL	-31	No	ns
Butyrate vs. Combo	7500µM vs. 100pg/mL	-37.33	No	ns
Butyrate vs. Combo	7500µM vs. 150pg/mL	-4.333	No	ns
Butyrate vs. Combo	7500µM vs. 200pg/mL	18	No	ns
Butyrate vs. Combo	7500µM vs. 250pg/mL	7	No	ns
Butyrate vs. Combo	7500µM vs. 500pg/mL	32.42	No	ns
Butyrate vs. Combo	7500µM vs. 1000pg/mL	40.67	No	ns
Butyrate vs. Combo	7500µM vs. 2500pg/mL	50.83	No	ns
Butyrate vs. Combo	7500µM vs. 5000pg/mL	56.17	No	ns
Combo vs. Combo	50pg/mL vs. 100pg/mL	-6.333	No	ns
Combo vs. Combo	50pg/mL vs. 150pg/mL	26.67	No	ns
Combo vs. Combo	50pg/mL vs. 200pg/mL	49	No	ns
Combo vs. Combo	50pg/mL vs. 250pg/mL	38	No	ns
Combo vs. Combo	50pg/mL vs. 500pg/mL	63.42	No	ns
Combo vs. Combo	50pg/mL vs. 1000pg/mL	71.67	No	ns
Combo vs. Combo	50pg/mL vs. 2500pg/mL	81.83	No	ns
Combo vs. Combo	50pg/mL vs. 5000pg/mL	87.17	No	ns
Combo vs. Combo	100pg/mL vs. 150pg/mL	33	No	ns
Combo vs. Combo	100pg/mL vs. 200pg/mL	55.33	No	ns
Combo vs. Combo	100pg/mL vs. 250pg/mL	44.33	No	ns
Combo vs. Combo	100pg/mL vs. 500pg/mL	69.75	No	ns
Combo vs. Combo	100pg/mL vs. 1000pg/mL	78	No	ns
Combo vs. Combo	100pg/mL vs. 2500pg/mL	88.17	No	ns
Combo vs. Combo	100pg/mL vs. 5000pg/mL	93.5	No	ns
Combo vs. Combo	150pg/mL vs. 200pg/mL	22.33	No	ns
Combo vs. Combo	150pg/mL vs. 250pg/mL	11.33	No	ns
Combo vs. Combo	150pg/mL vs. 500pg/mL	36.75	No	ns
Combo vs. Combo	150pg/mL vs. 1000pg/mL	45	No	ns
Combo vs. Combo	150pg/mL vs. 2500pg/mL	55.17	No	ns
Combo vs. Combo	150pg/mL vs. 5000pg/mL	60.5	No	ns
Combo vs. Combo	200pg/mL vs. 250pg/mL	-11	No	ns
Combo vs. Combo	200pg/mL vs. 500pg/mL	14.42	No	ns

Combo vs. Combo	200pg/mL vs. 1000pg/mL	22.67	No	ns
Combo vs. Combo	200pg/mL vs. 2500pg/mL	32.83	No	ns
Combo vs. Combo	200pg/mL vs. 5000pg/mL	38.17	No	ns
Combo vs. Combo	250pg/mL vs. 500pg/mL	25.42	No	ns
Combo vs. Combo	250pg/mL vs. 1000pg/mL	33.67	No	ns
Combo vs. Combo	250pg/mL vs. 2500pg/mL	43.83	No	ns
Combo vs. Combo	250pg/mL vs. 5000pg/mL	49.17	No	ns
Combo vs. Combo	500pg/mL vs. 1000pg/mL	8.25	No	ns
Combo vs. Combo	500pg/mL vs. 2500pg/mL	18.42	No	ns
Combo vs. Combo	500pg/mL vs. 5000pg/mL	23.75	No	ns
Combo vs. Combo	1000pg/mL vs. 2500pg/mL	10.17	No	ns
Combo vs. Combo	1000pg/mL vs. 5000pg/mL	15.5	No	ns

A-B
A-C
A-D
A-E
A-F
A-G
A-H
A-I
A-J
A-K
A-L
A-M
A-N
A-O
A-P
A-Q
A-R
A-S
A-T
A-U
A-V
A-W
A-X
A-Y
A-Z
A-AA
A-AB
A-AC
A-AD
B-C
B-D
B-E
B-F
B-G
B-H
B-I
B-J
B-K
B-L
B-M
B-N
B-O
B-P
B-Q
B-R
B-S

B-T
B-U
B-V
B-W
B-X
B-Y
B-Z
B-AA
B-AB
B-AC
B-AD
C-D
C-E
C-F
C-G
C-H
C-I
C-J
C-K
C-L
C-M
C-N
C-O
C-P
C-Q
C-R
C-S
C-T
C-U
C-V
C-W
C-X
C-Y
C-Z
C-AA
C-AB
C-AC
C-AD
D-E
D-F
D-G
D-H
D-I
D-J
D-K
D-L
D-M

D-N
D-O
D-P
D-Q
D-R
D-S
D-T
D-U
D-V
D-W
D-X
D-Y
D-Z
D-AA
D-AB
D-AC
D-AD
E-F
E-G
E-H
E-I
E-J
E-K
E-L
E-M
E-N
E-O
E-P
E-Q
E-R
E-S
E-T
E-U
E-V
E-W
E-X
E-Y
E-Z
E-AA
E-AB
E-AC
E-AD
F-G
F-H
F-I
F-J
F-K

F-L
F-M
F-N
F-O
F-P
F-Q
F-R
F-S
F-T
F-U
F-V
F-W
F-X
F-Y
F-Z
F-AA
F-AB
F-AC
F-AD
G-H
G-I
G-J
G-K
G-L
G-M
G-N
G-O
G-P
G-Q
G-R
G-S
G-T
G-U
G-V
G-W
G-X
G-Y
G-Z
G-AA
G-AB
G-AC
G-AD
H-I
H-J
H-K
H-L
H-M

H-N
H-O
H-P
H-Q
H-R
H-S
H-T
H-U
H-V
H-W
H-X
H-Y
H-Z
H-AA
H-AB
H-AC
H-AD
I-J
I-K
I-L
I-M
I-N
I-O
I-P
I-Q
I-R
I-S
I-T
I-U
I-V
I-W
I-X
I-Y
I-Z
I-AA
I-AB
I-AC
I-AD
J-K
J-L
J-M
J-N
J-O
J-P
J-Q
J-R
J-S

J-T
J-U
J-V
J-W
J-X
J-Y
J-Z
J-AA
J-AB
J-AC
J-AD
K-L
K-M
K-N
K-O
K-P
K-Q
K-R
K-S
K-T
K-U
K-V
K-W
K-X
K-Y
K-Z
K-AA
K-AB
K-AC
K-AD
L-M
L-N
L-O
L-P
L-Q
L-R
L-S
L-T
L-U
L-V
L-W
L-X
L-Y
L-Z
L-AA
L-AB
L-AC

L-AD
M-N
M-O
M-P
M-Q
M-R
M-S
M-T
M-U
M-V
M-W
M-X
M-Y
M-Z
M-AA
M-AB
M-AC
M-AD
N-O
N-P
N-Q
N-R
N-S
N-T
N-U
N-V
N-W
N-X
N-Y
N-Z
N-AA
N-AB
N-AC
N-AD
O-P
O-Q
O-R
O-S
O-T
O-U
O-V
O-W
O-X
O-Y
O-Z
O-AA
O-AB

O-AC
O-AD
P-Q
P-R
P-S
P-T
P-U
P-V
P-W
P-X
P-Y
P-Z
P-AA
P-AB
P-AC
P-AD
Q-R
Q-S
Q-T
Q-U
Q-V
Q-W
Q-X
Q-Y
Q-Z
Q-AA
Q-AB
Q-AC
Q-AD
R-S
R-T
R-U
R-V
R-W
R-X
R-Y
R-Z
R-AA
R-AB
R-AC
R-AD
S-T
S-U
S-V
S-W
S-X
S-Y

S-Z
S-AA
S-AB
S-AC
S-AD
T-U
T-V
T-W
T-X
T-Y
T-Z
T-AA
T-AB
T-AC
T-AD
U-V
U-W
U-X
U-Y
U-Z
U-AA
U-AB
U-AC
U-AD
V-W
V-X
V-Y
V-Z
V-AA
V-AB
V-AC
V-AD
W-X
W-Y
W-Z
W-AA
W-AB
W-AC
W-AD
X-Y
X-Z
X-AA
X-AB
X-AC
X-AD
Y-Z
Y-AA

Y-AB
Y-AC
Y-AD
Z-AA
Z-AB
Z-AC
Z-AD
AA-AB
AA-AC
AA-AD
AB-AC
AB-AD
